# A prospective cohort study of dietary intake and clinical–nutritional biomarkers in patients with periampullary malignancies undergoing pancreatoduodenectomy in Southeast Brazil

**DOI:** 10.1007/s00520-026-11357-8

**Published:** 2026-10-21

**Authors:** Marco Aurélio Ribeiro, José Sebastião Dos Santos, Gleici da Silva Castro Perdoná, Anderson Marliere Navarro

**Affiliations:** 1 Division of Clinical Nutrition, Department of Internal Medicine, Ribeirão Preto Medical School, University of São Paulo (FMRP-USP), Ribeirão Preto, Brazil; 2 Division of Digestive Surgery, Department of Surgery and Anatomy, Ribeirão Preto Medical School, University of São Paulo (FMRP-USP), Ribeirão Preto, Brazil; 3 Division of Epidemiology and Statistics, Department of Social Medicine, Ribeirão Preto Medical School, University of São Paulo (FMRP-USP), Ribeirão Preto, Brazil; 4 Division of Nutrition and Metabolism, Department of Health Sciences, Ribeirão Preto Medical School, University of São Paulo (FMRP-USP), Ribeirão Preto, Brazil

**Keywords:** Pancreatoduodenectomy, Dietary intake, Clinical–nutritional biomarkers, Inflammatory–nutritional indices, Periampullary neoplasms

## Abstract

**Purpose:**

Patients undergoing pancreatoduodenectomy for periampullary malignancies are at high nutritional risk. The aim was to characterize longitudinal changes in reported dietary intake, clinical–nutritional biomarkers, and inflammatory–nutritional indices after surgery and contextualize them through cross-sectional comparisons with age- and sex-similar controls.

**Methods:**

This prospective longitudinal cohort included 24 patients and 20 contextual controls. Patients were assessed preoperatively and at 3 and 6 months after discharge; controls were assessed once. Dietary intake was estimated using an adapted food frequency questionnaire (FFQ). FFQ-reported energy surplus was intake minus the 25 kcal/kg/day requirement based on the European Society for Clinical Nutrition and Metabolism (ESPEN). Longitudinal analyses, contextual comparisons, and exploratory adjusted diet–biomarker regressions were performed.

**Results:**

Of 24 patients assessed preoperatively, 18 and 16 were reassessed at 3 and 6 months. Median reported energy intake decreased from 2625 to 2011 and 1948 kcal/day, respectively. FFQ-reported energy surplus and intakes of macronutrients, fiber, calcium, iron, and selenium decreased, whereas zinc and vitamins A and E showed no detected longitudinal change. C-reactive protein, ferritin, liver enzymes, and serum vitamin E decreased. Glasgow Prognostic Score (GPS) and C-reactive protein-to-albumin ratio (CAR) decreased, the C-reactive protein–albumin–lymphocyte (CALLY) index increased, and the Prognostic Nutritional Index (PNI) showed no detected longitudinal change. Diet–biomarker associations were limited and inconsistent, although iron intake was positively associated with hemoglobin preoperatively in both adjusted models.

**Conclusion:**

During the first 6 months after pancreatoduodenectomy, reduced reported dietary intake persisted despite attenuation of systemic inflammation, while biochemical and inflammatory–nutritional indices followed divergent trajectories. These trajectories describe patients alive and available for follow-up.

**Supplementary Information:**

The online version contains supplementary material available at https://doi.org/10.1007/s00520-026-11357-8.

## Introduction

Periampullary malignancies involving the pancreatic head, ampulla, distal bile duct, and duodenum may disrupt digestive and metabolic homeostasis [1]. Pancreatic ductal adenocarcinoma (PDAC), the most aggressive entity, has a five-year survival of 13% [2]. Obstructive jaundice affects up to 71% of pancreatic head tumors [3], whereas distal lesions may present with back pain and weight loss. Established risk factors include older age, smoking, obesity, type 2 diabetes, chronic pancreatitis, and genetic susceptibility [1–4]. Pancreatoduodenectomy (PD) remains the main curative-intent treatment for resectable disease [2].

Nutritional impairment after PD reflects oncological burden and postoperative morbidity. Delayed gastric emptying (DGE) [5], postoperative pancreatic fistula (POPF) [6], and pancreatic exocrine insufficiency (PEI) [7] may compromise intake, digestion, and absorption amid systemic inflammation [8, 9]. Fearon et al. [10] showed that cancer-associated wasting, including sarcopenia/sarcopenic obesity, can coexist with normal or elevated body mass index (BMI) and is associated with complications, impaired treatment tolerance, poorer quality of life, and reduced survival [11]. Furthermore, Eckart et al. [12] emphasized that inflammation limits the interpretation of nutritional markers.

This combined burden supports structured nutritional assessment and support when indicated [8, 13, 14]. Nutritional Risk Screening 2002 (NRS-2002) [13, 14] and Patient-Generated Subjective Global Assessment (PG-SGA) [8, 11] are recommended, while Enhanced Recovery After Surgery (ERAS®), prehabilitation, and preoperative immunonutrition inform perioperative care [15, 16]. When oral intake is inadequate, enteral nutrition is preferred and parenteral nutrition reserved for infeasible or insufficient enteral feeding [8, 14]. Phillips et al. [17, 18] described persistent iron, zinc, calcium, and fat-soluble vitamin abnormalities after PD, particularly with untreated PEI [7]. Ganz [19] demonstrated the inflammatory dependence of iron metabolism, whereas Freedman et al. [20] documented systematic error in self-reported dietary intake. Appetite and gastrointestinal symptoms may further affect postoperative intake [8].

These overlapping determinants argue against isolated nutritional measures. Eckart et al. [12] and Evans et al. [21] emphasized that visceral proteins are influenced by inflammation and disease severity and lack specificity as standalone nutritional markers. Composite inflammatory–nutritional indices may provide complementary information, although the available evidence remains predominantly prognostic. For example, Liu et al. [22] evaluated the prognostic nutritional index (PNI), Sahakyan et al. [23] the Glasgow Prognostic Score (GPS), Liu et al. [24] the C-reactive protein-to-albumin ratio (CAR), and Kawahara et al. [25] the C-reactive protein–albumin–lymphocyte (CALLY) index. However, their longitudinal behavior after PD remains insufficiently characterized.

Accordingly, prospective studies integrating dietary intake, biochemical markers, and composite indices during follow-up remain scarce, particularly within Brazil’s Unified Health System (SUS), where longitudinal nutritional data after PD are limited. The primary descriptive objective was to characterize reported dietary intake trajectories from preoperative baseline (T0) to 3 (T1) and 6 months (T2) post-discharge. Secondary objectives were to characterize concurrent clinical–nutritional biomarker and inflammatory–nutritional index trajectories and contextualize them through cross-sectional comparisons with an age- and sex-similar control group assessed once. Diet–biomarker associations and postoperative clinical subgroup differences were exploratory and hypothesis-generating given the modest sample size and attrition.

## Methods

### Ethical aspects

The study was approved by the local Research Ethics Committee (CAAE 63993522.3.0000.5440 and 64695422.6.0000.5440), and written informed consent was obtained from all participants before enrollment. The study was conducted in accordance with the Declaration of Helsinki and registered on ClinicalTrials.gov on August 28, 2025 (NCT07159672).

### Study design

This prospective longitudinal cohort with contextual controls was conducted at a high-volume public academic center. In the Brazilian public health system, higher institutional PD volume has been associated with better short-term outcomes [26]. Consecutive recruitment occurred from February 1, 2023, to December 31, 2024. This secondary analysis of a broader prospective protocol evaluating resting energy expenditure and clinical–nutritional parameters focused on reported dietary intake, clinical–nutritional biomarkers, composite inflammatory–nutritional indices, and exploratory associations. Body composition, Global Leadership Initiative on Malnutrition (GLIM)-based malnutrition, and longitudinal cachexia will be reported separately. Patients were assessed preoperatively and 3 and 6 months post-discharge. Contextual controls underwent a single cross-sectional assessment. Reporting followed the Strengthening the Reporting of Observational Studies in Epidemiology (STROBE) Statement [27].

### Participants

**Fig. 1:**
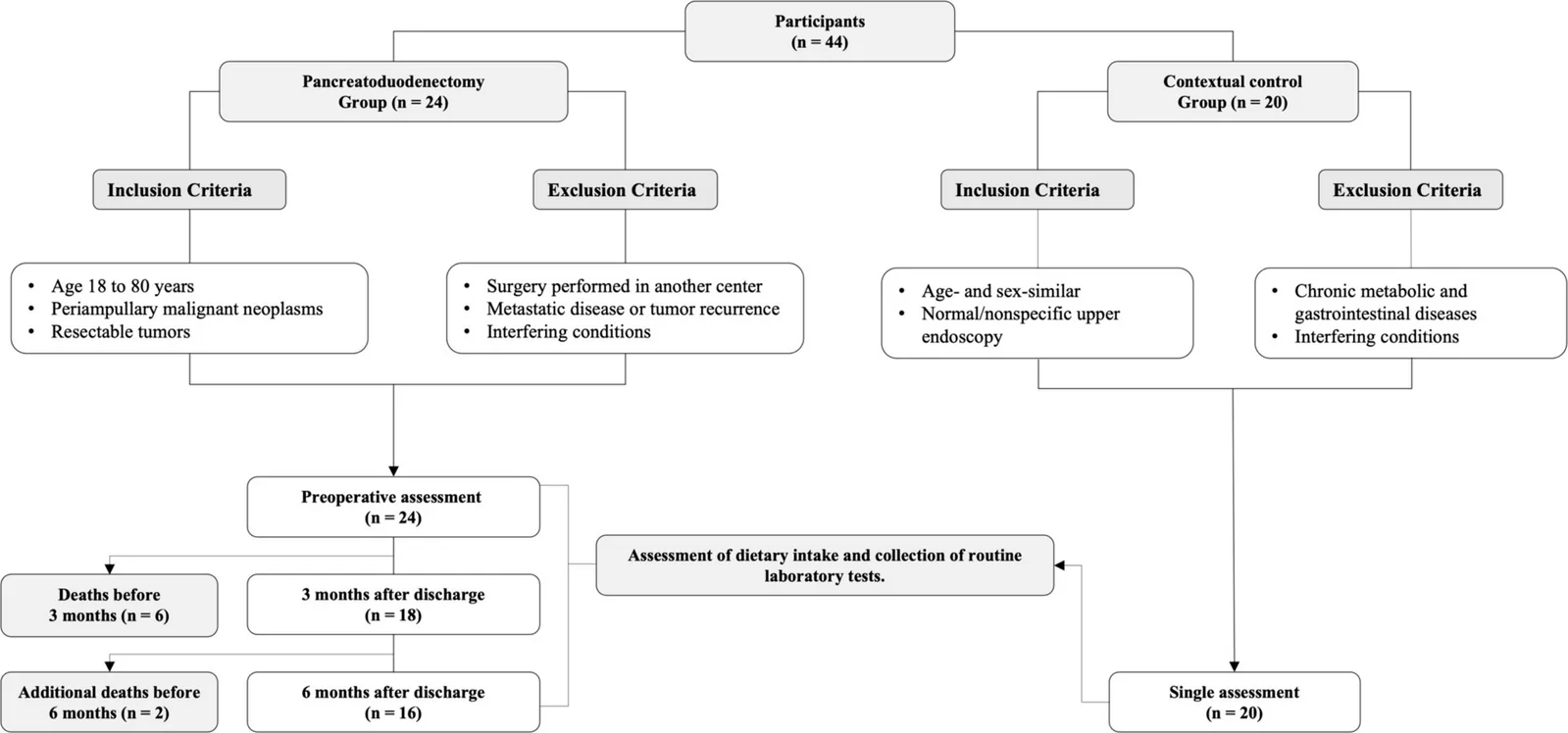
Study flowchart showing participants included at baseline, eligibility criteria, follow-up assessments, and deaths in the pancreatoduodenectomy group, as well as the single-time-point assessment in the control group

Eligible patients aged 18–80 years underwent classic or pylorus-preserving PD for periampullary malignancies, with histopathological staging according to the American Joint Committee on Cancer 8th edition [28]. Exclusion criteria were surgery performed outside the institution, unresectable disease, recurrence at enrollment, or distant metastasis. Contextual controls were age- and sex-similar individuals undergoing upper endoscopy with normal or nonspecific findings and no active or previous malignancy, active infection, or chronic gastrointestinal, inflammatory, autoimmune, metabolic, hepatic, or renal disease. Controls were used exclusively for contextual cross-sectional comparisons. Participant flow is shown in Fig. 1.

### Sample size

Sample size was based on the parent cohort primary outcome, longitudinal change in resting energy expenditure (REE) measured by indirect calorimetry. A one-group repeated-measures analysis of variance (ANOVA) with three assessments was specified in G*Power v3.1.9.7, using effect size *f* = 0.36 from Sasaki et al. [29], *α* = 0.05, 80% power, repeated-measures correlation = 0.50, and *ε* = 1.0. The minimum required sample was 14 evaluable patients. Allowing for postoperative attrition, 20 patients and a similar number of controls were initially planned; the final sample comprised 24 patients and 20 contextual controls. Because dietary, biochemical, inflammatory, and subgroup analyses were secondary to the parent outcome, they were considered descriptive and hypothesis-generating.

### Variables, anthropometry, and dietary assessment

The outcomes included dietary variables, clinical–nutritional biomarkers, and inflammatory–nutritional indices. Baseline anthropometry included weight, height, BMI, fat-free mass index (FFMI), and involuntary weight loss. Weight and height were measured to the nearest 0.1 kg and 0.5 cm, respectively, using calibrated Filizola scales and stadiometers. Usual weight was defined as stable weight or weight six months before baseline, and weight loss was calculated as 100 × (usual weight − baseline weight)/usual weight. Fat-free mass (FFM) was measured using multifrequency octapolar bioelectrical impedance (seca mBCA 525, Hamburg, Germany), and FFMI was calculated as FFM/height^2^. According to Fearon et al. [10], cancer cachexia is defined as weight loss of more than 5%, or more than 2% with a BMI of less than 20 kg/m^2^, or low muscle mass as defined by the European Society for Clinical Nutrition and Metabolism (ESPEN) with FFMI cutoffs of less than 17 kg/m^2^ for men and less than 15 kg/m^2^ for women [13]. Dietary variables included energy, macronutrients, fiber, micronutrients, estimated energy requirements, and food frequency questionnaire (FFQ)-reported energy surplus. The requirement was the actual body weight multiplied by 25 kcal/kg/day, which is the lower bound for cancer patients according to ESPEN guidelines [30] and was used as a clinical reference rather than the physiological energy expenditure. The FFQ-reported energy surplus was the reported intake minus this estimate. The 114-item Brazilian Longitudinal Study of Adult Health (ELSA-Brasil) FFQ, which was validated for habitual intake [31], was adapted to the preceding 30 days. Frequencies were converted to daily intake, and nutrients were derived from the Brazilian Food Composition Table (TACO; NEPA-UNICAMP, 4th edition). No standardized nutritional intervention was part of the research protocol. When clinically indicated, the Gastrosurgery Service provided nutritional follow-up, pancreatic enzyme replacement therapy (PERT) titration, and micronutrient supplementation as routine care.

### Clinical–nutritional biomarkers and indices

Biomarkers comprised visceral protein, inflammatory, glycemic, hematologic, iron metabolism, renal, hepatic, and micronutrient markers measured by standardized assays in certified institutional laboratories. Derived indices used same-assessment laboratory values. The PNI was 10 × albumin (g/dL) + 5 × lymphocytes (10^3^/µL) [22]. The GPS was 0 when neither C-reactive protein (CRP) > 10 mg/L nor albumin < 3.5 g/dL was present, 1 for either criterion, and 2 for both criteria [23]. The CAR was CRP (mg/L)/albumin (g/dL) [24]. Albumin/CRP, CRP/lymphocyte, ferritin/albumin, and ferritin/lymphocyte ratios were calculated directly. The CALLY index was albumin (g/dL) × lymphocytes (10^3^/µL)/CRP (mg/L) [25]. Clinical variables comprised biliary drainage by endoscopic retrograde cholangiopancreatography (ERCP), DGE, postoperative pancreatic fistula (POPF), Clavien–Dindo grade, PEI, and pancreatic enzyme replacement therapy (PERT) exposure [32–35]. DGE and POPF followed International Study Group of Pancreatic Surgery (ISGPS) criteria [5, 6]. Biochemical leak was not clinically relevant POPF, whereas grades B–C were. Clavien–Dindo grades III–V defined major and I–II minor morbidity [32–34]. Without systematic fecal elastase-1, PEI was clinically classified as suspected or documented, with severity based on symptoms and PERT requirements [7, 16]. PERT exposure was descriptive [7, 17]. Exploratory subgroup analyses used these variables, with DGE additionally classified as primary or secondary according to Marchegiani et al. [36].

### Bias control and missing data

Consecutive recruitment and standardized data collection, trained staff, the adapted ELSA-Brasil FFQ, and centralized assays addressed selection and measurement bias. Mortality-related attrition was potentially informative. Observed data were not imputed because post-death dietary and biochemical outcomes are undefined. Mixed-effects models therefore describe patients alive and available at each postoperative assessment.

### Statistical analysis

The primary descriptive outcomes were longitudinal dietary changes, while the secondary descriptive outcomes were clinical-nutritional biomarkers and inflammatory-nutritional indices. Diet-biomarker regressions were exploratory. The analyses were performed using RStudio v2026.04.0 + 526 (R v4.3.0). Continuous variables were summarized as the mean ± standard deviation (SD) or median (interquartile range [IQR]), according to Shapiro–Wilk tests and quantile–quantile (*Q*–*Q*) plots. Categorical variables were summarized as *n* (%). Levene’s test was used to assess homoscedasticity, and Welch’s correction was applied when necessary. Preoperative (T0), three-month (T1), and six-month (T2) changes were analyzed using linear mixed models (LMMs, Satterthwaite degrees of freedom) or generalized linear mixed models (GLMMs) with random intercepts (lme4 and lmerTest). Pairwise contrasts used emmeans. Contextual control comparisons used a Student’s *t*-test, a Welch’s *t*-test, or a Mann–Whitney *U*-test. Six planned longitudinal and control contrasts per variable were adjusted using the Benjamini–Hochberg false discovery rate (FDR). Separate multiple linear regressions examined the associations between diet and biomarkers, adjusting for age, sex, and either CRP or ferritin, except when ferritin was the outcome [12, 19, 21]. Regression *p*-values were not multiplicity-adjusted because the analyses were exploratory. Interpretation emphasized effect estimates, 95% confidence intervals, and consistency. Exploratory subgroups (Supplementary Tables S5–S11) used observed data without imputation or covariate adjustment. Post hoc, mortality-related sensitivity analyses (Supplementary Table S12) included inverse-probability weighting, pattern-mixture models under adverse-value scenarios, and joint-parameter models.

## Results

### Clinical characteristics of the sample

Twenty-four patients in the PD group were assessed preoperatively, 18 at three months, and 16 at six months. Twenty contextual controls were assessed once. Cancer cachexia affected 87.5% of patients. The PD group was predominantly male, and pancreatic adenocarcinoma was the most common diagnosis. The clinical, nutritional, histopathological, treatment (Table 1), postoperative complication, PEI, and PERT characteristics are summarized (Table 2).

**Table 1:** Demographic and baseline nutritional characteristics of patients undergoing pancreatoduodenectomy (PD) and contextual controls, and histopathological diagnoses, TNM staging characteristics, and follow-up characteristics of the PD group

Variable	*Pancreatoduodenectomy group (n* = *24)*	*Contextual control group (n* = *20)*
Age (years)	63.0 ± 11.9	61.9 ± 12.5
Male sex, *n* (%)	16 (66.7)	12 (60.0)
Female sex, *n* (%)	8 (33.3)	8 (40.0)
Height (m)	1.66 ± 0.10	1.66 ± 0.12
Baseline body weight (kg)	75.8 ± 17.0	77.9 ± 20.0
Baseline body mass index (kg/m^2^)	27.09 ± 4.50	28.00 ± 5.52
Baseline BIA-derived fat-free mass index (kg/m^2^)	18.23 ± 3.39	17.61 ± 3.53
**Preoperative involuntary weight loss from usual body weight in the PD group, *n* (%)**		
> 10%	2 (8.3)	
> 5 to ≤ 10%	18 (75.0)	
≤ 5%	4 (16.7)	
**Cancer cachexia in the PD group according to the Fearon consensus framework, *n* (%)**		
Present	21 (87.5)	
Absent	3 (12.5)	
**Histopathological diagnosis of the PD group, *n* (%)**		
Pancreatic adenocarcinoma	14 (58.3)	
Ampullary adenocarcinoma	8 (33.3)	
Distal bile duct carcinoma	2 (8.3)	
**Primary tumor extent (pT), *n* (%)**		
pT1	5 (20.8)	
pT2	7 (29.2)	
pT3–pT4	6 (25.0)	
Not available	6 (25.0)	
**Regional lymph node involvement (pN), *n* (%)**		
pN0	8 (33.3)	
pN1–pN2	10 (41.7)	
Not available	6 (25.0)	
**Distant metastasis (M), *n* (%)**		
M0	18 (75.0)	
Not available	6 (25.0)	
**Postoperative systemic treatment, *n* (%)**		
Adjuvant − intent chemotherapy	12 (50.0)	
Palliative − intent chemotherapy	7 (29.2)	
No chemotherapy documented/not available	5 (20.8)	
**Cumulative deaths before the 6-month nutritional assessment, *n* (%)**	8 (33.3)	

Continuous variables are presented as mean ± standard deviation and categorical variables as *n* (%). Cancer cachexia was defined according to the Fearon consensus framework as weight loss > 5%, or > 2% with body mass index < 20 kg/m^2^ or low muscle mass; low muscle mass was operationalized as BIA-derived FFMI < 17 kg/m^2^ in men and < 15 kg/m^2^ in women. Tumors were staged according to the UICC/AJCC TNM classification, 8th edition; pT3–pT4 and pN1–pN2 were grouped descriptively

*AJCC* American Joint Committee on Cancer, *BIA* bioelectrical impedance analysis, *FFMI* fat-free mass index, *PD* pancreatoduodenectomy, *TNM* tumor–node–metastasis, *UICC* Union for International Cancer Control

**Table 2:** Preoperative biliary drainage, postoperative complications, exocrine pancreatic insufficiency, and pancreatic enzyme replacement therapy among patients undergoing pancreatoduodenectomy

Variable	*n (%)*
**Preoperative biliary drainage by ERCP**	
Yes	16 (66.7)
No	8 (33.3)
**Type of preoperative biliary drainage among ERCP patients**	
ERCP with a plastic biliary stent	15 (93.8)
ERCP with a fully covered self-expandable metal biliary stent	1 (6.3)
**DGE classification according to ISGPS**	
No DGE	2 (8.3)
Grade A	14 (58.3)
Grade B	5 (20.8)
Grade C	3 (12.5)
**POPF classification according to ISGPS**	
Biochemical leak	7 (29.2)
Grade B	14 (58.3)
Grade C	3 (12.5)
**Clavien–Dindo classification**	
I	7 (29.2)
II	3 (12.5)
IIIa	11 (45.8)
IIIb	0 (0.0)
IVa	0 (0.0)
IVb	0 (0.0)
V	3 (12.5)
**Clinically suspected or documented exocrine pancreatic insufficiency**	
Yes	19 (79.2)
No	5 (20.8)
**EPI grade**	
Normal	5 (20.8)
Mild	0 (0.0)
Moderate	11 (45.8)
Severe	8 (33.3)
**Pancreatic enzyme replacement therapy (PERT)**	
Yes	10 (41.7)
No	14 (58.3)

Values are presented as *n* (%). Percentages were calculated using the total cohort (*n* = 24), except for biliary stent type, calculated among patients undergoing ERCP (*n* = 16). DGE and POPF were classified according to ISGPS criteria; biochemical leak was not considered clinically relevant POPF, whereas grades B–C were. Clavien–Dindo grade represents the highest postoperative complication grade; grade V included only deaths attributable to postoperative complications. PEI was clinically classified because fecal elastase-1 was not systematically available; severity was assigned from medical-record documentation based on symptoms, evidence of fat malabsorption when available, nutritional impact, and PERT requirement

*DGE* delayed gastric emptying, *ERCP* endoscopic retrograde cholangiopancreatography, *ISGPS* International Study Group of Pancreatic Surgery, *EPI* exocrine pancreatic insufficiency, *PERT* pancreatic enzyme replacement therapy, *POPF* postoperative pancreatic fistula

### Food intake analysis

**Fig. 2:**
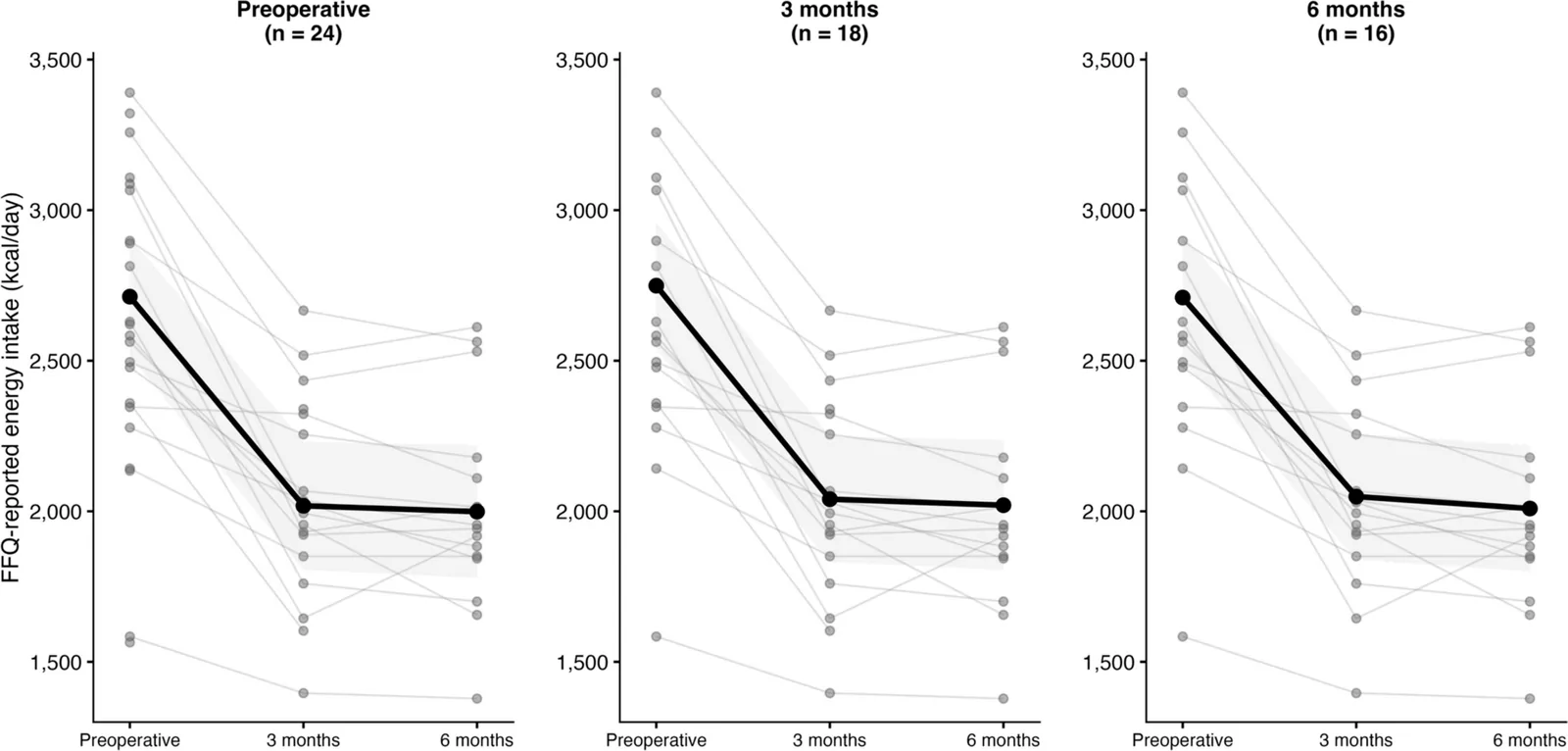
Individual trajectories of FFQ-reported energy intake after pancreatoduodenectomy. Each panel shows longitudinal trajectories across the preoperative, 3-month, and 6-month assessments for patients with observed FFQ-reported energy intake at the assessment indicated in the panel title. Thin gray lines represent individual patients. The black line represents the model-based marginal mean, and the shaded grey band indicates the 95% confidence interval. Postoperative estimates represent patients who survived and remained available for follow-up. FFQ, food frequency questionnaire

Reported intake decreased primarily from baseline to three months and remained reduced at six months. The median energy intake was 2625, 2011, and 1948 kcal/day, respectively (Fig. 2). Meanwhile, the FFQ-reported energy surplus decreased from 1034 kcal/day to 262 and 289 kcal/day. Intakes of macronutrients, fiber, calcium, iron, and selenium decreased, while zinc and vitamins A and E showed no longitudinal change (Table 3 and Supplementary Table S1). Post-discharge energy, energy surplus, lipid, and selenium intakes were lower than those of contextual controls.

**Table 3:** Reported dietary intake and ESPEN-based energy adequacy variables in PD patients at baseline, 3 months, and 6 months after hospital discharge and in the control group

Variable	Preoperative (*n* = 24) Mean ± SD or median (P25–P75)	3 months (*n* = 18) Mean ± SD or median (P25–P75)	6 months (*n* = 16) Mean ± SD or median (P25–P75)	*p*^*A*^	*p*^*B*^	*p*^*C*^	Control group (*n* = 20) Mean ± SD or median (P25–P75)	*p*^*D*^	*p*^*E*^	*p*^*F*^
Energy (kcal)	2625.20 (2355.82–3092.25)	2011.49 (1868.48–2306.26)	1948.38 (1848.74–2127.03)	< 0.001	0.765	< 0.001	2294.69 (2042.03–2711.35)	0.002	0.002	0.007
Protein (g)	117.25 ± 23.13	96.59 ± 14.33	101.05 ± 15.61	< 0.001	0.240	< 0.001	98.18 ± 20.97	0.004	0.166	0.166
ESPEN-based estimated energy requirement (kcal/day)	1876.35 ± 417.67	1819.86 ± 368.34	1756.56 ± 366.72	0.001	0.536	< 0.001	1948.56 ± 499.63	0.604	0.536	0.416
FFQ-reported energy surplus (kcal/day)	1033.60 (491.48–1180.03)	261.90 (56.96–413.51)	288.85 (53.01–517.03)	0.003	0.691	0.003	405.26 (221.82–603.67)	0.003	0.152	0.447
Carbohydrate (g)	383.65 (319.45–431.04)	265.69 (239.20–280.49)	261.88 (234.10–279.17)	< 0.001	0.992	< 0.001	284.77 (251.84–339.31)	< 0.001	< 0.001	< 0.001
Lipid (g)	81.46 (66.44–89.04)	63.96 (56.67–77.55)	58.38 (50.32–77.20)	< 0.001	0.235	< 0.001	85.82 (71.05–100.25)	< 0.001	< 0.001	< 0.001
Fiber (g)	46.03 ± 11.30	38.14 ± 9.20	36.96 ± 8.76	0.002	0.877	0.002	24.91 ± 9.45	< 0.001	< 0.001	< 0.001
Calcium (mg)	1475.32 ± 312.03	1310.28 ± 384.84	1255.37 ± 349.57	0.034	0.668	0.029	656.79 ± 387.69	< 0.001	< 0.001	< 0.001
Iron (mg)	26.49 (22.85–28.97)	21.72 (17.75–27.47)	21.02 (18.33–25.69)	0.002	0.813	0.002	12.51 (10.49–14.63)	< 0.001	< 0.001	< 0.001
Potassium (mg)	51.46 (46.77–68.88)	48.10 ± 20.80	47.31 ± 19.77	0.005	0.626	0.004	170.37 (71.00–282.27)	< 0.001	0.004	0.008
Selenium (µg)	17.39 (15.26–19.34)	13.56 (11.36–16.45)	13.14 (11.55–15.44)	< 0.001	0.694	< 0.001	11.98 (9.52–13.60)	< 0.001	< 0.001	< 0.001
Zinc (mg)	5.04 (3.50–8.27)	4.32 (3.07–7.24)	4.56 (2.84–6.66)	0.509	0.832	0.509	1.08 (0.93–1.66)	< 0.001	< 0.001	< 0.001
Sodium (mg)	4.42 (3.06–5.99)	3.75 (2.78–6.00)	3.48 (2.37–5.07)	0.305	0.305	0.108	10.36 (8.58–11.73)	0.004	0.166	0.166

Data are presented as mean ± SD or median (P25–P75), as appropriate. FFQ-reported energy surplus was calculated as FFQ-reported daily energy intake minus the ESPEN-based estimated energy requirement (actual body weight × 25 kcal/kg/day); positive values indicate reported intake above this clinical reference. Longitudinal comparisons were performed using linear mixed-effects models or generalized linear mixed models, as appropriate. Contextual control comparisons used Student’s t-test, Welch’s *t*-test, or the Mann–Whitney *U* test, according to distributional assumptions and variance homogeneity. *p*^*A*^ = *preoperative vs. 3 months; p*^*B*^ = *3 months vs. 6 months; p*^*C*^ = *preoperative vs. 6 months; p*^*D*^ = *control group vs. preoperative; p*^*E*^ = *control group vs. 3 months; p*^*F*^ = *control group vs. 6 months.* Benjamini–Hochberg correction was applied within each variable across *p*^*A*^*, p*^*B*^*, p*^*C*^*, p*^*D*^*, p*^*E*^*,* and *p*^*F*^

*P25*–*P75*, 25th–75th percentiles; *SD*, standard deviation

### Clinical–nutritional laboratory analyses

Six months later, the median CRP level had decreased from 6.19 to 0.40 mg/L, while aspartate aminotransferase (AST) and alanine aminotransferase (ALT) decreased (Table 4 and Supplementary Table S2). Ferritin levels decreased but remained higher than those of the control group. Hemoglobin showed no detected longitudinal change, whereas vitamin A showed no longitudinal change but remained lower than in contextual controls. Vitamin E decreased from 7.46 to 5.54 mg/L and remained lower postoperatively. GPS and CAR decreased, while CALLY increased. PNI showed no longitudinal change (Table 4 and Supplementary Table S3). No exploratory subgroup difference remained after Benjamini–Hochberg correction, while sensitivity analyses broadly preserved the inferential pattern (Supplementary Tables S5–S12).

**Table 4:** Clinical laboratory biomarkers and inflammatory–nutritional indices in patients undergoing pancreatoduodenectomy over follow-up and in the control group

Variable	Preoperative (*n* = 24) Mean ± SD or median (P25–P75)	3 months (*n* = 18) Mean ± SD or median (P25–P75)	6 months (*n* = 16) Mean ± SD or median (P25–P75)	*p*^*A*^	*p*^*B*^	*p*^*C*^	Control group (*n* = 20) Mean ± SD or median (P25–P75)	*p*^*D*^	*p*^*E*^	*p*^*F*^
**Laboratory biomarkers**										
Albumin (g/dL)	4.04 (3.45–4.46)	4.22 (3.98–4.37)	4.25 (3.94–4.58)	0.650	0.730	0.650	4.51 (4.39–4.58)	0.009	0.032	0.130
C-reactive protein (mg/L)	6.19 (2.64–17.03)	0.77 (0.40–3.93)	0.40 (0.40–1.69)	< 0.001	0.497	< 0.001	0.40 (0.40–0.41)	< 0.001	0.019	0.111
Blood glucose (mg/dL)	121.54 (97.36–154.50)	119.81 (84.59–134.69)	110.81 (79.81–119.47)	0.289	0.377	0.098	96.15 (85.56–109.36)	0.319	0.853	1.000
Hemoglobin (g/dL)	12.62 ± 2.25	12.56 ± 1.61	12.27 ± 2.17	0.897	0.897	0.897	13.74 ± 1.82	0.081	0.064	0.064
Ferritin (ng/mL)	336.00 (93.67–828.80)	244.45 (175.72–470.33)	206.15 (125.85–408.50)	0.023	0.336	0.009	97.75 (49.42–179.80)	0.013	0.015	0.029
Transferrin saturation (%)	23.07 ± 8.09	22.92 ± 9.11	21.98 ± 8.19	0.974	0.880	0.880	20.51 ± 7.16	0.553	0.553	0.569
Urea (mg/dL)	34.72 (25.59–41.40)	34.30 (26.75–48.57)	31.25 (22.65–40.44)	0.981	0.981	0.981	34.16 (28.65–40.00)	0.934	0.934	0.934
Creatinine (mg/dL)	0.92 (0.80–1.16)	0.84 (0.74–0.95)	0.87 (0.78–1.09)	0.548	0.548	0.991	1.12 (0.93–1.26)	0.238	0.106	0.189
Aspartate aminotransferase (AST) (U/L)	147.88 (81.54–267.31)	28.45 (23.88–36.96)	30.47 (26.89–39.06)	< 0.001	0.984	< 0.001	24.01 (18.96–28.54)	< 0.001	0.118	0.016
Alanine aminotransferase (ALT) (U/L)	146.68 (71.69–253.93)	34.27 (28.38–44.12)	34.82 (25.84–43.71)	< 0.001	0.833	< 0.001	24.48 (15.97–29.56)	< 0.001	0.015	0.012
Vitamin B12 (pg/mL)	478.00 (410.00–688.00)	491.00 (375.50–720.00)	385.50 (315.44–544.50)	0.783	0.151	0.137	460.50 (417.00–566.75)	0.775	0.815	0.775
Vitamin A (mg/L)	0.30 (0.19–0.35)	0.33 (0.24–0.39)	0.30 (0.28–0.36)	0.599	0.711	0.599	0.55 (0.45–0.71)	< 0.001	< 0.001	< 0.001
Vitamin E (mg/L)	7.46 (6.11–8.60)	6.59 (4.86–7.67)	5.54 (4.83–6.57)	0.002	0.333	< 0.001	9.30 (6.53–10.58)	0.283	0.019	0.007
Copper (µg/dL)	139.00 (105.35–152.45)	103.00 (91.10–115.25)	94.90 (88.76–113.73)	< 0.001	0.553	< 0.001	109.00 (91.95–113.80)	0.027	0.672	0.572
Zinc (µg/dL)	87.50 (58.25–96.00)	78.00 (75.00–87.88)	80.25 (69.88–88.00)	0.188	0.188	0.837	83.50 (74.57–87.12)	0.930	0.930	0.930
**Derived inflammatory–nutritional indices**										
Prognostic nutritional index	50.68 ± 9.65	53.50 ± 7.46	53.79 ± 9.16	0.461	0.926	0.461	54.42 ± 3.64	0.268	0.797	0.797
Glasgow prognostic score (GPS)	1.00 (0.00–1.00)	0.00 (0.00–0.00)	0.00 (0.00–0.00)	0.026	0.405	0.026	0.00 (0.00–0.00)	< 0.001	0.097	0.288
CRP/albumin ratio	1.66 (0.62–4.09)	0.17 (0.09–1.09)	0.11 (0.09–0.40)	0.002	0.461	0.002	0.09 (0.09–0.10)	< 0.001	0.012	0.030
Albumin-lymphocytes-CRP index	1.36 (0.42–3.45)	12.12 (1.95–28.45)	17.40 (7.21–31.52)	0.014	0.521	0.014	20.30 (15.92–26.09)	< 0.001	0.409	0.787

Data are presented as mean ± SD or median (P25–P75), as appropriate. Longitudinal comparisons were performed using linear mixed-effects models or generalized linear mixed models, as appropriate. Contextual control comparisons used Student’s *t*-test, Welch’s *t*-test, or the Mann–Whitney *U* test, according to distributional assumptions and variance homogeneity. *p*^*A*^ = *preoperative vs. 3 months; p*^*B*^ = *3 months vs. 6 months; p*^*C*^ = *preoperative vs. 6 months; p*^*D*^ = *control group vs. preoperative; p*^*E*^ = *control group vs. 3 months; p*^*F*^ = *control group vs. 6 months.* Benjamini–Hochberg correction was applied within each variable across *p*^*A*^*, p*^*B*^*, p*^*C*^*, p*^*D*^*, p*^*E*^*, and p*^*F*^

*P25*–*P75*, 25th–75th percentiles; *SD*, standard deviation

### Association between food intake and clinical–nutritional biomarkers

Exploratory regressions revealed limited and inconsistent associations (Supplementary Table S4). Iron intake was positively associated with hemoglobin preoperatively in both the CRP- and ferritin-adjusted models. Protein intake was only associated with albumin at six months in the ferritin-adjusted model. No consistent postoperative associations were observed for zinc, fat-soluble vitamins, or protein. In contextual controls, vitamin E intake was inversely associated with serum vitamin E in the ferritin-adjusted model only.

## Discussion

In this prospective cohort, postoperative survivor trajectories combined sustained reductions in reported intake with attenuated systemic inflammation and liver enzyme abnormalities, selective fat-soluble vitamin changes, and limited diet–biomarker correspondence. Their divergence supports multiparametric nutritional interpretation rather than reliance on isolated markers.

### Sample characteristics and perioperative clinical profile

Older age, male predominance, and pancreatic adenocarcinoma characterized the cohort, consistent with pancreatic and periampullary malignancy patterns [1, 2]. Cancer cachexia affected 87.5% despite mean BMI in the overweight range, consistent with Fearon et al. and ESPEN criteria [10, 13] showing that weight loss or depleted muscle mass may coexist with preserved or elevated BMI. Preoperative ERCP drainage was frequent, although van der Gaag et al. [3] and American College of Gastroenterology guidance [35] support selective decompression. Postoperative morbidity was substantial, with frequent DGE and clinically relevant POPF [5, 6, 32–34]. Marchegiani et al. [36] distinguished primary and secondary DGE phenotypes, and Dai et al. [37] confirmed its multifactorial risk profile, underscoring clinical heterogeneity. Expert recommendations likewise emphasize POPF prevention, mitigation, and management [38]. The 12.5% Clavien–Dindo grade V rate exceeded the 8% in-hospital mortality in high-volume SUS institutions but remained below the 17% in low-volume centers [26]. Comparability is limited because our estimate reflects complication-attributable mortality in a small prospective cohort, whereas Brazilian estimates derive from retrospective administrative data. Grade V was restricted to postoperative-complication deaths, whereas other follow-up deaths reflected oncological progression or nonsurgical deterioration. Suspected or documented PEI was frequent, whereas PERT exposure was limited. This contrasts with the recognized post-PD PEI burden [7] and ISGPS nutritional recommendations [16], suggesting a potential implementation gap, although undertreatment cannot be inferred because indication, dose adequacy, access, and adherence were not protocolized.

### Food intake

Reported dietary intake decreased mainly during the first 3 postoperative months and remained reduced at 6 months. Energy intake fell from 2625 kcal/day preoperatively to 2011 and 1948 kcal/day at 3 and 6 months, alongside reductions in FFQ-reported energy surplus and most macronutrients. This differed from Wijma et al. [8], who reported increased postoperative caloric intake despite reductions in protein intake, body weight, and muscle thickness, underscoring heterogeneous nutritional recovery after pancreatic surgery. The ESPEN lower bound of 25 kcal/kg/day [30] served only as a clinical reference, whereas Sasaki et al. [29] measured early postoperative resting energy expenditure of approximately 25.5 kcal/kg/day and proposed 30 kcal/kg/day after applying an activity factor of 1.2–1.3. Although self-reported intake is subject to measurement error [20], concordant reductions across dietary variables support the observed trajectory. Calcium, iron, and selenium intakes decreased, whereas no longitudinal change was detected for zinc, consistent with broader post-PD micronutrient vulnerability [7, 17, 18] but not equivalent to biochemical depletion. Reported vitamin E intake also showed no detected longitudinal change despite lower postoperative serum vitamin E, suggesting that intake alone may not reflect circulating fat-soluble vitamin status in the presence of PEI, maldigestion, and variable PERT exposure [7, 17].

### Clinical–nutritional biomarkers and inflammatory–nutritional indices

Postoperatively, CRP, AST, and ALT decreased, indicating attenuation of baseline inflammatory and liver enzyme abnormalities. Ferritin also decreased but remained higher than in contextual controls. This agrees with Ganz [19], who described the inflammatory dependence of ferritin, and with Eckart et al. [12] and Evans et al. [21], who emphasized the limited specificity of visceral proteins as nutritional markers. However, ferritin reduction was not accompanied by detected longitudinal changes in hemoglobin or most other iron-related parameters, indicating that attenuation of inflammation did not parallel the entire iron profile. Ferritin, albumin, and related markers should therefore be interpreted within the inflammatory context rather than as direct surrogates of nutritional recovery [12, 19, 21]. Fat-soluble vitamins followed a different trajectory. Vitamin A showed no detected longitudinal change but remained lower than in contextual controls, whereas vitamin E decreased despite unchanged reported intake [39]. This discordance is consistent with Kroon et al. [7] and Phillips et al. [17, 18], who described pancreatic exocrine dysfunction and adequacy of enzyme replacement as relevant to fat-soluble vitamin status after PD. Reported intake alone therefore did not reflect circulating vitamin E [39]. Serum copper also decreased, although postoperative trace-element concentrations may reflect absorption and inflammatory changes as well as intake [17]. Composite indices were likewise nonparallel. GPS and CAR decreased, CALLY increased, and no longitudinal change was detected in PNI. Liu et al. [22], Sahakyan et al. [23], Liu et al. [24], and Kawahara et al. [25] have primarily evaluated these indices as prognostic markers rather than longitudinal measures of postoperative recovery. In this cohort, GPS and CAR paralleled CRP reduction, consistent with their direct inflammatory components, whereas PNI followed a different trajectory and cannot be interpreted as evidence of preserved nutritional or immune status. The increase in CALLY further shows that these indices capture different combinations of inflammatory, protein, and hematologic variables and should be interpreted as complementary rather than interchangeable.

### Association between food intake and clinical–nutritional biomarkers

Reported dietary intake showed limited and inconsistent correspondence with circulating biomarkers after PD. Eckart et al. [12] and Evans et al. [21] emphasized that visceral proteins are strongly influenced by inflammation and disease severity, while Ganz [19] and Kosuge et al. [40] described inflammatory regulation of iron availability. Measurement error inherent to self-reported dietary assessment, as discussed by Freedman et al. [20], may have further weakened these associations. Protein intake was associated with albumin only at 6 months in the ferritin-adjusted model, supporting the limited value of albumin as a direct marker of protein intake [12, 21]. Conversely, iron intake was positively associated with hemoglobin preoperatively in both CRP- and ferritin-adjusted models but not after PD, consistent with the inflammatory disturbances in iron metabolism described by Ganz [19], although causality cannot be established. Vitamin E showed a similar dissociation. No consistent postoperative association was detected between reported intake and serum vitamin E [39]. Kroon et al. [7] and Phillips et al. [17, 18] described PEI, fat maldigestion, and adequacy of enzyme replacement as relevant determinants of fat-soluble vitamin status after PD. An inverse association in contextual controls occurred only in the ferritin-adjusted model and was not reproduced across other analyses, arguing against a consistent intake–biomarker relationship. Overall, FFQ-reported intake and circulating biomarkers represented related but non-equivalent dimensions of nutritional assessment. Given the modest sample size and inconsistency across assessments and adjustment strategies, these exploratory associations should be considered hypothesis-generating.

### Study limitations and strengths

This study has limitations. Sample size was determined for the parent cohort outcome, limiting power for adjusted diet–biomarker regressions and subgroup analyses, which remain exploratory. Attrition was predominantly mortality-related and potentially informative. Analyses used observed data without imputation, and mixed-effects models cannot fully address death-related missingness. Postoperative estimates therefore represent patients alive and available at each assessment rather than the entire baseline cohort. Sensitivity analyses remained constrained by the modest sample size and few deaths. FFQ assessment is susceptible to recall error, portion-size misestimation, and exposure misclassification. Freedman et al. [20] showed systematic misreporting of self-reported energy and protein intake in relation to adiposity, relevant to this cohort’s BMI distribution. Baseline BMI and cancer cachexia were therefore reported (Table 1). FFQ-reported energy surplus was based on actual body weight × 25 kcal/kg/day and should be interpreted relative to an ESPEN-based clinical reference, not as physiological energy balance. Formal GLIM classification and longitudinal body-composition analyses were outside the study scope. Subgroup analyses were further constrained by small strata, multiple comparisons, and limited capacity for multivariable adjustment. Postoperative systemic treatment was reported descriptively but not modeled because of sample size. Pancreatic texture and duct diameter were not systematically available. Without routine fecal elastase-1, PEI was clinically classified, while PERT exposure did not establish indication, dose adequacy, or adherence. Contextual controls were assessed once and provided cross-sectional context rather than a longitudinal counterfactual. Strengths include the prospective longitudinal design, predefined assessments, standardized data collection, an FFQ adapted from a validated Brazilian instrument, centralized laboratory measurements, multiplicity adjustment for planned contrasts, and STROBE-guided reporting [27]. Integration of dietary intake, clinical–nutritional biomarkers, and inflammatory–nutritional indices enabled assessment across complementary domains.

## Conclusion

Among patients with periampullary malignancies undergoing PD, reported energy, macronutrient, and selected micronutrient intakes decreased primarily during the first three months and remained reduced at six months. These dietary trajectories were accompanied by a reduction in systemic inflammation and liver enzyme abnormalities, while changes in fat-soluble vitamins were selective. Composite inflammatory-nutritional indices exhibited divergent trajectories: decreases in GPS and CAR, an increase in CALLY, and no longitudinal change in PNI. Contextual cross-sectional comparisons revealed persistent differences in certain biomarkers compared to controls. Reported dietary intake showed limited and inconsistent correspondence with circulating biomarkers. No exploratory clinical subgroup differences remained after multiplicity correction. FFQ-reported energy surplus should be interpreted relative to a clinical reference based on ESPEN guidelines rather than as a physiological energy balance. Overall, these findings characterize complementary but non-equivalent dietary, biochemical, and inflammatory trajectories during the first six months after PD. Diet-biomarker and subgroup findings remain hypothesis-generating.

## Supplementary Information

Below is the link to the electronic supplementary material.

ESM1 (DOCX 263 KB)

## Data Availability

The datasets generated and/or analyzed during the current study are not publicly available due to patient confidentiality and ethical restrictions but may be made available by the corresponding author upon reasonable request and with approval from the Research Ethics Committee.

## Declarations

### Declaration of artificial intelligence and digital tools use

STORM supported conceptual exploration. OpenEvidence, Consensus, ResearchRabbit, and LeapSpace supported literature identification. Gemini Notebook supported document organization and summaries and Grammarly supported English editing. These tools did not generate data, perform analyses, determine methods, interpret findings, or make authorship decisions. The authors verified all references and outputs and retain full responsibility for the manuscript.
