# Supplementary material for: A prospective cohort study of dietary intake and clinical–nutritional biomarkers in patients with periampullary malignancies undergoing pancreatoduodenectomy in Southeast Brazil
Source: Support Care Cancer. 2026 Oct 21;34(11):1130. doi: 10.1007/s00520-026-11357-8 (PMC13687123; doi:10.1007/s00520-026-11357-8)
Supplement: Supplementary file 1 [file 520_2026_11357_MOESM1_ESM.docx]

| **Supplementary Table S1.** Additional reported dietary intake and ESPEN-based energy adequacy variables in PD patients at baseline, 3 months, and 6 months after hospital discharge and in the control group. | | | | | | | | | | |
| --- | --- | --- | --- | --- | --- | --- | --- | --- | --- | --- |
| **Variable** | **Preoperative**  **(n=24)**  **Mean ± SD or Median**  **(P25–P75)** | **3 months**  **(n=18)**  **Mean ± SD or Median**  **(P25–P75)** | **6 months**  **(n=16)**  **Mean ± SD or Median**  **(P25–P75)** | **Statistical test**  **95% CI**  ***p^A^*** | **Statistical test**  **95% CI**  ***p^B^*** | **Statistical test**  **95% CI**  ***p^C^*** | **Control Group**  **(n=20)**  **Mean ± SD or Median**  **(P25–P75)** | **Statistical test**  **95% CI**  ***p^D^*** | **Statistical test**  **95% CI**  ***p^E^*** | **Statistical test**  **95% CI**  ***p^F^*** |
| Energy (kcal) | 2625.20 (2355.82–3092.25) | 2011.49 (1868.48–2306.26) | 1948.38 (1848.74–2127.03) | z = 7.968; Δ = 673.420; 95% CI [507.771, 839.068]; p < 0.001 | z = 0.299; Δ = 22.454; 95% CI [-124.792, 169.700]; p = 0.765 | z = 7.989; Δ = 695.873; 95% CI [525.153, 866.593]; p < 0.001 | 2294.69 (2042.03–2711.35) | U = -105.0; Δ = -333.784; 95% CI [-469.755, -161.267]; p = 0.002 | U = 83.0; Δ = 307.748; 95% CI [158.911, 431.292]; p = 0.002 | U = 35.0; Δ = 199.489; 95% CI [71.889, 307.593]; p = 0.007 |
| Protein (g) | 117.25 ± 23.13 | 96.59 ± 14.33 | 101.05 ± 15.61 | t(34.6) = 6.230; Δ = 23.001; 95% CI [15.503, 30.500]; p < 0.001 | t(32.6) = -1.196; Δ = -4.687; 95% CI [-12.664, 3.289]; p = 0.240 | t(34.8) = 4.746; Δ = 18.314; 95% CI [10.479, 26.150]; p < 0.001 | 98.18 ± 20.97 | U = -108.0; Δ = -83.711; 95% CI [-131.660, -37.420]; p = 0.004 | U = 19.0; Δ = 25.712; 95% CI [-8.540, 69.390]; p = 0.166 | U = -6.0; Δ = 28.210; 95% CI [-8.750, 73.020]; p = 0.166 |
| ESPEN-based estimated energy requirement (kcal/day) | 1876.35 ± 417.67 (n=24) | 1819.86 ± 368.34 (n=18) | 1756.56 ± 366.72 (n=16) | t(32.6) = 3.922; Δ = 138.873; 95% CI [66.801, 210.946]; p =0.001 | t(32.1) = 0.771; Δ = 28.631; 95% CI [-47.008, 104.269]; p =0.536 | t(32.6) = 4.524; Δ = 167.504; 95% CI [92.138, 242.870]; pBH < 0.001 | 1948.56 ± 499.63 (n=20) | t(42.0) = -0.522; Δ = -72.208; 95% CI [-351.176, 206.759]; p =0.604 | t(36.0) = -0.895; Δ = -128.701; 95% CI [-420.278, 162.875]; p =0.536 | t(34.0) = -1.284; Δ = -192.000; 95% CI [-495.944, 111.944]; p =0.416 |
| FFQ-reported energy surplus (kcal/day) | 1033.60 (491.48–1180.03) (n=24) | 261.90 (56.96–413.51) (n=18) | 288.85 (53.01–517.03) (n=16) | z = 3.462; Δ = 568.910; 95% CI [320.250, 819.735]; p =0.003 | z = -0.398; Δ = -8.673; 95% CI [-47.110, 70.565]; p =0.691 | z = 3.154; Δ = 512.370; 95% CI [249.425, 801.920]; p =0.003 | 405.26 (221.82–603.67) (n=20) | U = 380.0; Δ = 564.079; 95% CI [274.440, 699.390]; p =0.003 | U = 124.0; Δ = -173.162; 95% CI [-363.650, 33.290]; p =0.152 | U = 132.0; Δ = -120.561; 95% CI [-335.590, 86.170]; p =0.447 |
| Carbohydrate (g) | 383.65 (319.45–431.04) | 265.69 (239.20–280.49) | 261.88 (234.10–279.17) | z = 9.963; Δ = 110.723; 95% CI [88.942, 132.504]; p < 0.001 | z = 0.010; Δ = 0.099; 95% CI [-18.396, 18.593]; p = 0.992 | z = 9.678; Δ = 110.822; 95% CI [88.379, 133.265]; p < 0.001 | 284.77 (251.84–339.31) | t(42.0) = -6.641; Δ = -21.123; 95% CI [-27.542, -14.703]; p < 0.001 | t(36.0) = -4.365; Δ = -13.234; 95% CI [-19.383, -7.084]; p < 0.001 | t(34.0) = -3.927; Δ = -12.057; 95% CI [-18.295, -5.818]; p < 0.001 |
| Lipid (g) | 81.46 (66.44–89.04) | 63.96 (56.67–77.55) | 58.38 (50.32–77.20) | z = 3.819; Δ = 15.676; 95% CI [7.630, 23.721]; p < 0.001 | z = 1.188; Δ = 4.488; 95% CI [-2.915, 11.892]; p = 0.235 | z = 4.858; Δ = 20.164; 95% CI [12.029, 28.299]; p < 0.001 | 85.82 (71.05–100.25) | t(42.0) = -7.762; Δ = -818.535; 95% CI [-1031.347, -605.722]; p < 0.001 | t(36.0) = -5.206; Δ = -653.496; 95% CI [-908.066, -398.926]; p < 0.001 | t(34.0) = -4.806; Δ = -598.583; 95% CI [-851.713, -345.452]; p < 0.001 |
| Fiber (g) | 46.03 ± 11.30 | 38.14 ± 9.20 | 36.96 ± 8.76 | t(35.5) = 3.557; Δ = 7.945; 95% CI [3.413, 12.477]; p = 0.002 | t(33.0) = 0.156; Δ = 0.372; 95% CI [-4.482, 5.225]; p = 0.877 | t(35.8) = 3.564; Δ = 8.316; 95% CI [3.583, 13.050]; p = 0.002 | 24.91 ± 9.45 | U = -206.0; Δ = -13.814; 95% CI [-16.650, -10.660]; p < 0.001 | U = -196.0; Δ = -9.607; 95% CI [-13.700, -6.290]; p < 0.001 | U = -200.0; Δ = -8.808; 95% CI [-12.600, -5.720]; p < 0.001 |
| Calcium (mg) | 1475.32 ± 312.03 | 1310.28 ± 384.84 | 1255.37 ± 349.57 | t(35.6) = 2.385; Δ = 180.828; 95% CI [27.017, 334.640]; p = 0.034 | t(33.1) = 0.433; Δ = 35.103; 95% CI [-129.789, 199.995]; p = 0.668 | t(36.0) = 2.727; Δ = 215.932; 95% CI [55.308, 376.555]; p = 0.029 | 656.79 ± 387.69 | t(38.3) = -11.481; Δ = -3675.206; 95% CI [-4323.102, -3027.311]; p < 0.001 | t(27.7) = -7.828; Δ = -2707.328; 95% CI [-3416.122, -1998.533]; p < 0.001 | t(34.0) = -7.829; Δ = -2535.649; 95% CI [-3193.843, -1877.456]; p < 0.001 |
| Iron (mg) | 26.49 (22.85–28.97) | 21.72 (17.75–27.47) | 21.02 (18.33–25.69) | z = 3.191; Δ = 3.902; 95% CI [1.505, 6.299]; p = 0.002 | z = 0.236; Δ = 0.281; 95% CI [-2.044, 2.605]; p = 0.813 | z = 3.304; Δ = 4.183; 95% CI [1.702, 6.664]; p = 0.002 | 12.51 (10.49–14.63) | U = 186.0; Δ = 116.093; 95% CI [46.110, 201.880]; p < 0.001 | U = 104.0; Δ = 126.257; 95% CI [47.110, 217.400]; p < 0.001 | U = 67.0; Δ = 125.190; 95% CI [47.520, 222.990]; p < 0.001 |
| Potassium (mg) | 6769.97 (5984.62–7452.64) | 5295.36 (4777.38–6888.53) | 5288.48 (4558.52–6435.36) | z = 4.312; Δ = 1029.033; 95% CI [561.315, 1496.752]; p < 0.001 | z = 0.402; Δ = 93.230; 95% CI [-360.848, 547.307]; p = 0.687 | z = 4.559; Δ = 1122.263; 95% CI [639.835, 1604.692]; p < 0.001 | 3022.22 ± 779.62 | U = -141.0; Δ = -5.580; 95% CI [-8.170, -3.480]; p < 0.001 | U = -94.0; Δ = -2.284; 95% CI [-5.260, 0.110]; p = 0.095 | U = -99.0; Δ = -1.815; 95% CI [-4.300, 0.360]; p = 0.123 |
| Selenium (µg) | 57.49 ± 19.41 | 48.10 ± 20.80 | 47.31 ± 19.77 | t(34.3) = 3.142; Δ = 10.836; 95% CI [3.829, 17.843]; p = 0.005 | t(32.5) = 0.492; Δ = 1.796; 95% CI [-5.640, 9.233]; p = 0.626 | t(34.4) = 3.504; Δ = 12.632; 95% CI [5.309, 19.956]; p = 0.004 | 170.37 (71.00–282.27) | U = -154.0; Δ = -861.197; 95% CI [-1259.950, -518.440]; p < 0.001 | U = -134.0; Δ = -483.248; 95% CI [-763.680, -214.850]; p = 0.004 | U = -134.0; Δ = -408.448; 95% CI [-701.700, -140.760]; p = 0.008 |
| Zinc (mg) | 17.39 (15.26–19.34) | 13.56 (11.36–16.45) | 13.14 (11.55–15.44) | z = 5.714; Δ = 3.547; 95% CI [2.330, 4.763]; p < 0.001 | z = 0.393; Δ = 0.219; 95% CI [-0.874, 1.312]; p = 0.694 | z = 5.870; Δ = 3.766; 95% CI [2.509, 5.023]; p < 0.001 | 11.98 (9.52–13.60) | U = -197.0; Δ = -3.493; 95% CI [-5.100, -2.490]; p < 0.001 | U = -203.0; Δ = -3.063; 95% CI [-4.990, -1.970]; p < 0.001 | U = -202.5; Δ = -3.180; 95% CI [-4.120, -1.850]; p < 0.001 |
| Sodium (mg) | 1656.51 (1267.89–2440.80) | 1258.16 (1127.98–1648.89) | 1159.76 (994.86–1599.22) | z = 5.655; Δ = 476.457; 95% CI [311.322, 641.591]; p < 0.001 | z = 0.504; Δ = 35.077; 95% CI [-101.460, 171.614]; p = 0.615 | z = 5.905; Δ = 511.533; 95% CI [341.746, 681.321]; p < 0.001 | 761.34 (616.51–1131.48) | U = -84.0; Δ = -136.793; 95% CI [-262.600, -19.250]; p = 0.022 | U = -95.0; Δ = -115.293; 95% CI [-247.280, 7.370]; p = 0.089 | U = -103.0; Δ = -96.781; 95% CI [-227.780, 17.840]; p = 0.095 |
| Vitamin A (mg) | 5.04 (3.50–8.27) | 4.32 (3.07–7.24) | 4.56 (2.84–6.66) | z = 0.956; Δ = 0.409; 95% CI [-0.429, 1.247]; p = 0.509 | z = 0.212; Δ = 0.091; 95% CI [-0.748, 0.929]; p = 0.832 | z = 1.133; Δ = 0.500; 95% CI [-0.364, 1.364]; p = 0.509 | 1.08 (0.93–1.66) | U = 250.0; Δ = 5.827; 95% CI [4.160, 7.380]; p < 0.001 | U = 129.0; Δ = 5.872; 95% CI [3.960, 7.870]; p < 0.001 | U = 95.0; Δ = 6.524; 95% CI [4.360, 8.260]; p < 0.001 |
| Vitamin C (mg) | 343.70 (226.44–541.27) | 325.13 (210.74–465.86) | 314.27 (211.08–419.62) | z = 1.663; Δ = 37.174; 95% CI [-6.642, 80.991]; p = 0.145 | z = 0.267; Δ = 5.841; 95% CI [-37.093, 48.775]; p = 0.790 | z = 1.862; Δ = 43.016; 95% CI [-2.256, 88.287]; p = 0.145 | 257.48 (109.08–337.16) | U = -105.0; Δ = -333.784; 95% CI [-469.755, -161.267]; p = 0.002 | U = 83.0; Δ = 307.748; 95% CI [158.911, 431.292]; p = 0.002 | U = 35.0; Δ = 199.489; 95% CI [71.889, 307.593]; p = 0.007 |
| Vitamin E (mg) | 4.42 (3.06–5.99) | 3.75 (2.78–6.00) | 3.48 (2.37–5.07) | z = 1.081; Δ = 0.400; 95% CI [-0.326, 1.127]; p = 0.305 | z = 1.026; Δ = 0.370; 95% CI [-0.337, 1.077]; p = 0.305 | z = 2.098; Δ = 0.770; 95% CI [0.051, 1.490]; p = 0.108 | 10.36 (8.58–11.73) | U = -108.0; Δ = -83.711; 95% CI [-131.660, -37.420]; p = 0.004 | U = 19.0; Δ = 25.712; 95% CI [-8.540, 69.390]; p = 0.166 | U = -6.0; Δ = 28.210; 95% CI [-8.750, 73.020]; p = 0.166 |
| Notes: Data are presented as mean ± SD or median (P25–P75), as appropriate. The sample sizes shown in the column headers correspond to the preoperative assessment (n = 24), 3 months after hospital discharge (n = 18), 6 months after hospital discharge (n = 16), and the control group (n = 20). FFQ-reported energy surplus was calculated as FFQ-reported daily energy intake minus the ESPEN-based estimated energy requirement, defined as actual body weight × 25 kcal/kg/day, and expressed in kcal/day. Because all observed values were positive, this variable represents FFQ-reported intake above the estimated requirement. Longitudinal comparisons within the pancreatoduodenectomy group were performed using linear mixed-effects models for approximately normally distributed variables, with degrees of freedom estimated by Satterthwaite’s method, or generalized linear mixed models for non-normally distributed variables. Between-group comparisons with the control group were performed using Student’s t-test, Welch’s t-test, or the Mann–Whitney U test, according to distributional assumptions and variance homogeneity. *p^A^* = preoperative vs. 3 months; *p^B^* = 3 months vs. 6 months; *p^C^* = preoperative vs. 6 months; *p^D^* = control group vs. preoperative; pE = control group vs. 3 months; pF = control group vs. 6 months. Benjamini–Hochberg correction was applied within each variable across *p^A^, p^B^, pC, p^D^, p^E^, and p^F^.* | | | | | | | | | | |

|  | | | | | | | | | | |
| --- | --- | --- | --- | --- | --- | --- | --- | --- | --- | --- |
| **Supplementary Table S2.** Measures of centrality and dispersion of clinical–nutritional laboratory analyses data in PD patients at preoperative. 3. and 6 months after discharge and in the control group. | | | | | | | | | | |
| **Variable** | **Preoperative**  **(n=24)**  **Mean ± SD or Median**  **(P25–P75)** | **3 months**  **(n=18)**  **Mean ± SD or Median**  **(P25–P75)** | **6 months**  **(n=16)**  **Mean ± SD or Median**  **(P25–P75)** | **Statistical test**  **95% CI**  ***p^A^*** | **Statistical test**  **95% CI**  ***p^B^*** | **Statistical test**  **95% CI**  ***p^C^*** | **Control Group**  **(n=20)**  **Mean ± SD or Median**  **(P25–P75)** | **Statistical test**  **95% CI**  ***p^D^*** | **Statistical test**  **95% CI**  ***p^E^*** | **Statistical test**  **95% CI**  ***p^F^*** |
| Total Proteins (g/dL) | 6.91 ± 0.56 | 6.88 ± 0.48 | 6.65 ± 0.51 | t(36.8) = 0.078; Δ = 0.011; 95% CI [-0.265, 0.286]; p = 0.939 | t(33.9) = 1.711; Δ = 0.251; 95% CI [-0.047, 0.550]; p = 0.144 | t(37.5) = 1.846; Δ = 0.262; 95% CI [-0.025, 0.549]; p = 0.144 | 6.99 ± 0.60 | t(42.0) = 0.471; Δ = 0.082; 95% CI [-0.271, 0.436]; p = 0.640 | t(36.0) = 0.632; Δ = 0.112; 95% CI [-0.247, 0.471]; p = 0.640 | t(34.0) = 1.796; Δ = 0.339; 95% CI [-0.045, 0.722]; p = 0.244 |
| Albumin (g/dL) | 4.04 (3.45–4.46) | 4.22 (3.98–4.37) | 4.25 (3.94–4.58) | z = -0.784; Δ = -0.149; 95% CI [-0.521, 0.223]; p = 0.650 | z = -0.345; Δ = -0.074; 95% CI [-0.493, 0.345]; p = 0.730 | z = -1.118; Δ = -0.223; 95% CI [-0.613, 0.168]; p = 0.650 | 4.51 (4.39–4.58) | U = 156.5; Δ = 0.460; 95% CI [0.120, 0.810]; p = 0.009 | U = 49.0; Δ = 0.293; 95% CI [0.070, 0.560]; p = 0.032 | U = -2.0; Δ = 0.206; 95% CI [-0.100, 0.490]; p = 0.130 |
| C-Reactive Protein (mg/L) | 6.19 (2.64–17.03) | 0.77 (0.40–3.93) | 0.40 (0.40–1.69) | z = 3.527; Δ = 8.434; 95% CI [3.747, 13.120]; p < 0.001 | z = 0.680; Δ = 0.449; 95% CI [-0.846, 1.744]; p = 0.497 | z = 3.706; Δ = 8.883; 95% CI [4.185, 13.581]; p < 0.001 | 0.40 (0.40–0.41) | U = -181.0; Δ = -5.151; 95% CI [-10.620, -3.500]; p < 0.001 | U = -106.0; Δ = -0.360; 95% CI [-2.970, -0.000]; p = 0.019 | U = -92.5; Δ = -0.000; 95% CI [-0.330, 0.000]; p = 0.111 |
| Blood Glucose (mg/dL) | 121.54 (97.36–154.50) | 119.81 (84.59–134.69) | 110.81 (79.81–119.47) | z = 1.303; Δ = 11.729; 95% CI [-5.919, 29.378]; p = 0.289 | z = 0.884; Δ = 7.833; 95% CI [-9.531, 25.197]; p = 0.377 | z = 2.138; Δ = 19.562; 95% CI [1.626, 37.499]; p = 0.098 | 96.15 (85.56–109.36) | U = -39.0; Δ = -15.519; 95% CI [-42.080, 3.430]; p = 0.319 | U = -50.0; Δ = -7.222; 95% CI [-34.530, 12.130]; p = 0.853 | U = -50.0; Δ = -0.202; 95% CI [-22.220, 18.490]; p = 1.000 |
| Hemoglobin (g/dL) | 12.62 ± 2.25 | 12.56 ± 1.61 | 12.27 ± 2.17 | t(35.5) = 0.130; Δ = 0.060; 95% CI [-0.878, 0.998]; p = 0.897 | t(33.0) = 0.530; Δ = 0.262; 95% CI [-0.743, 1.267]; p = 0.897 | t(35.9) = 0.667; Δ = 0.322; 95% CI [-0.657, 1.301]; p = 0.897 | 13.74 ± 1.82 | t(42.0) = 1.790; Δ = 1.121; 95% CI [-0.143, 2.385]; p = 0.081 | t(36.0) = 2.101; Δ = 1.178; 95% CI [0.041, 2.314]; p = 0.064 | t(34.0) = 2.212; Δ = 1.473; 95% CI [0.120, 2.825]; p = 0.064 |
| Lymphocytes (x10³/mm³) | 2.23 ± 1.24 | 2.49 ± 0.93 | 2.41 ± 1.03 | t(38.1) = -0.803; Δ = -0.264; 95% CI [-0.929, 0.401]; p = 0.785 | t(35.5) = 0.275; Δ = 0.099; 95% CI [-0.631, 0.829]; p = 0.785 | t(39.4) = -0.483; Δ = -0.165; 95% CI [-0.856, 0.526]; p = 0.785 | 1.80 (1.67–2.42) | U = 14.5; Δ = -0.000; 95% CI [-0.600, 0.500]; p = 0.721 | U = -103.5; Δ = -0.500; 95% CI [-1.000, -0.100]; p = 0.097 | U = -97.0; Δ = -0.400; 95% CI [-0.912, 0.300]; p = 0.206 |
| Serum Iron (µg/dL) | 87.64 (52.29–103.39) | 95.20 (50.22–121.90) | 88.28 (61.43–98.20) | z = -0.449; Δ = -5.775; 95% CI [-31.002, 19.452]; p = 0.900 | z = 0.547; Δ = 7.368; 95% CI [-19.047, 33.782]; p = 0.900 | z = 0.126; Δ = 1.593; 95% CI [-23.254, 26.440]; p = 0.900 | 85.31 (64.51–103.01) | U = 13.0; Δ = -3.240; 95% CI [-23.680, 20.090]; p = 0.886 | U = -47.0; Δ = -4.565; 95% CI [-36.620, 25.400]; p = 0.886 | U = -55.0; Δ = -2.755; 95% CI [-28.020, 22.180]; p = 0.886 |
| Ferritin (ng/mL) | 336.00 (93.67–828.80) | 244.45 (175.72–470.33) | 206.15 (125.85–408.50) | z = 2.429; Δ = 222.123; 95% CI [42.906, 401.340]; p = 0.023 | z = 0.961; Δ = 51.544; 95% CI [-53.539, 156.626]; p = 0.336 | z = 2.973; Δ = 273.667; 95% CI [93.246, 454.087]; p = 0.009 | 97.75 (49.42–179.80) | U = -92.0; Δ = -220.129; 95% CI [-607.900, -52.500]; p = 0.013 | U = -119.0; Δ = -150.853; 95% CI [-316.200, -22.900]; p = 0.015 | U = -119.0; Δ = -100.985; 95% CI [-233.100, -13.680]; p = 0.029 |
| UIBC (µg/dL) | 284.25 (251.39–311.06) | 300.86 (280.74–308.16) | 296.85 (282.52–314.73) | z = -1.283; Δ = -17.892; 95% CI [-45.219, 9.436]; p = 0.299 | z = -0.298; Δ = -4.565; 95% CI [-34.543, 25.413]; p = 0.765 | z = -1.524; Δ = -22.456; 95% CI [-51.328, 6.415]; p = 0.299 | 318.29 ± 39.91 | t(37.3) = 1.802; Δ = 30.595; 95% CI [-3.801, 64.992]; p = 0.212 | t(36.0) = 1.301; Δ = 19.699; 95% CI [-11.003, 50.402]; p = 0.212 | t(34.0) = 1.271; Δ = 16.952; 95% CI [-10.155, 44.060]; p = 0.212 |
| TIBC (µg/dL) | 375.09 ± 85.31 | 394.56 ± 92.23 | 389.97 ± 61.38 | t(36.3) = -1.288; Δ = -26.080; 95% CI [-67.117, 14.957]; p = 0.542 | t(33.5) = 0.300; Δ = 6.534; 95% CI [-37.740, 50.807]; p = 0.766 | t(36.9) = -0.925; Δ = -19.546; 95% CI [-62.369, 23.276]; p = 0.542 | 402.39 ± 52.61 | t(39.0) = 1.299; Δ = 27.292; 95% CI [-15.217, 69.801]; p = 0.605 | t(26.4) = 0.317; Δ = 7.827; 95% CI [-42.944, 58.597]; p = 0.754 | t(34.0) = 0.654; Δ = 12.419; 95% CI [-26.192, 51.031]; p = 0.754 |
| Transferrin (mg/dL) | 227.40 (201.11–248.85) | 240.68 (224.59–246.53) | 237.48 (226.02–251.78) | z = -1.283; Δ = -14.314; 95% CI [-36.176, 7.549]; p = 0.299 | z = -0.298; Δ = -3.652; 95% CI [-27.634, 20.331]; p = 0.765 | z = -1.524; Δ = -17.965; 95% CI [-41.062, 5.132]; p = 0.299 | 254.63 ± 31.93 | t(37.3) = 1.802; Δ = 24.476; 95% CI [-3.041, 51.994]; p = 0.212 | t(36.0) = 1.301; Δ = 15.759; 95% CI [-8.803, 40.321]; p = 0.212 | t(34.0) = 1.271; Δ = 13.562; 95% CI [-8.124, 35.248]; p = 0.212 |
| Transferrin Saturation (%) | 23.07 ± 8.09 | 22.92 ± 9.11 | 21.98 ± 8.19 | t(36.9) = -0.033; Δ = -0.074; 95% CI [-4.588, 4.441]; p = 0.974 | t(34.0) = 0.560; Δ = 1.349; 95% CI [-3.547, 6.245]; p = 0.880 | t(37.6) = 0.549; Δ = 1.275; 95% CI [-3.432, 5.982]; p = 0.880 | 20.51 ± 7.16 | t(42.0) = -1.102; Δ = -2.564; 95% CI [-7.259, 2.130]; p = 0.553 | t(36.0) = -0.911; Δ = -2.410; 95% CI [-7.774, 2.955]; p = 0.553 | t(34.0) = -0.576; Δ = -1.474; 95% CI [-6.677, 3.729]; p = 0.569 |
| Urea (mg/dL) | 34.72 (25.59–41.40) | 34.30 (26.75–48.57) | 31.25 (22.65–40.44) | z = -0.163; Δ = -0.806; 95% CI [-10.480, 8.868]; p = 0.981 | z = 0.173; Δ = 0.926; 95% CI [-9.578, 11.429]; p = 0.981 | z = 0.023; Δ = 0.120; 95% CI [-9.916, 10.156]; p = 0.981 | 34.16 (28.65–40.00) | U = 34.0; Δ = 0.342; 95% CI [-6.770, 6.210]; p = 0.934 | U = -35.0; Δ = -0.294; 95% CI [-11.770, 5.690]; p = 0.934 | U = -28.0; Δ = 2.277; 95% CI [-5.750, 9.520]; p = 0.934 |
| Creatinine (mg/dL) | 0.92 (0.80–1.16) | 0.84 (0.74–0.95) | 0.87 (0.78–1.09) | z = 0.968; Δ = 0.077; 95% CI [-0.079, 0.232]; p = 0.548 | z = -0.905; Δ = -0.078; 95% CI [-0.246, 0.091]; p = 0.548 | z = -0.011; Δ = -0.001; 95% CI [-0.170, 0.169]; p = 0.991 | 1.12 (0.93–1.26) | U = 80.5; Δ = 0.120; 95% CI [-0.080, 0.290]; p = 0.238 | U = 42.5; Δ = 0.200; 95% CI [0.010, 0.365]; p = 0.106 | U = -1.5; Δ = 0.151; 95% CI [-0.060, 0.330]; p = 0.189 |
| Aspartate Aminotransferase (AST) (U/L) | 147.88 (81.54–267.31) | 28.45 (23.88–36.96) | 30.47 (26.89–39.06) | z = 5.547; Δ = 127.934; 95% CI [82.733, 173.136]; p < 0.001 | z = -0.020; Δ = -0.137; 95% CI [-13.780, 13.505]; p = 0.984 | z = 5.494; Δ = 127.797; 95% CI [82.206, 173.387]; p < 0.001 | 24.01 (18.96–28.54) | U = -161.0; Δ = -122.897; 95% CI [-152.770, -74.580]; p < 0.001 | U = -84.0; Δ = -5.100; 95% CI [-10.930, 1.470]; p = 0.118 | U = -131.0; Δ = -7.207; 95% CI [-13.070, -1.970]; p = 0.016 |
| Alanine Aminotransferase (ALT) (U/L) | 146.68 (71.69–253.93) | 34.27 (28.38–44.12) | 34.82 (25.84–43.71) | z = 4.517; Δ = 176.498; 95% CI [99.915, 253.082]; p < 0.001 | z = -0.211; Δ = -1.754; 95% CI [-18.086, 14.578]; p = 0.833 | z = 4.409; Δ = 174.744; 95% CI [97.064, 252.424]; p < 0.001 | 24.48 (15.97–29.56) | U = -185.0; Δ = -122.850; 95% CI [-186.750, -65.040]; p < 0.001 | U = -114.0; Δ = -11.007; 95% CI [-19.630, -2.700]; p = 0.015 | U = -134.0; Δ = -11.054; 95% CI [-21.150, -3.440]; p = 0.012 |
| Folic Acid (ng/mL) | 13.85 (9.91–18.99) | 16.91 (10.68–24.00) | 21.52 (13.80–24.00) | z = -1.545; Δ = -2.687; 95% CI [-6.095, 0.721]; p = 0.183 | z = -0.820; Δ = -1.726; 95% CI [-5.852, 2.399]; p = 0.412 | z = -2.278; Δ = -4.413; 95% CI [-8.210, -0.616]; p = 0.068 | 19.34 (16.79–22.66) | U = 128.0; Δ = 5.010; 95% CI [0.710, 8.430]; p = 0.063 | U = -15.0; Δ = 0.099; 95% CI [-2.300, 6.840]; p = 0.736 | U = -61.0; Δ = -0.178; 95% CI [-4.300, 2.590]; p = 0.736 |
| Vitamin B12 (pg/mL) | 478.00 (410.00–688.00) | 491.00 (375.50–720.00) | 385.50 (315.44–544.50) | z = 0.275; Δ = 19.590; 95% CI [-120.064, 159.244]; p = 0.783 | z = 1.641; Δ = 111.680; 95% CI [-21.741, 245.101]; p = 0.151 | z = 1.999; Δ = 131.270; 95% CI [2.538, 260.001]; p = 0.137 | 460.50 (417.00–566.75) | U = 2.0; Δ = -36.347; 95% CI [-172.000, 65.000]; p = 0.775 | U = -38.5; Δ = -16.749; 95% CI [-171.000, 94.000]; p = 0.815 | U = -17.0; Δ = 71.184; 95% CI [-84.000, 139.060]; p = 0.775 |
| Vitamin A (mg/L) | 0.30 (0.19–0.35) | 0.33 (0.24–0.39) | 0.30 (0.28–0.36) | z = -0.843; Δ = -0.027; 95% CI [-0.089, 0.036]; p = 0.599 | z = -0.370; Δ = -0.014; 95% CI [-0.086, 0.058]; p = 0.711 | z = -1.194; Δ = -0.040; 95% CI [-0.107, 0.026]; p = 0.599 | 0.55 (0.45–0.71) | U = 242.0; Δ = 0.270; 95% CI [0.180, 0.390]; p < 0.001 | U = 119.0; Δ = 0.250; 95% CI [0.140, 0.360]; p < 0.001 | U = 82.0; Δ = 0.220; 95% CI [0.140, 0.350]; p < 0.001 |
| Vitamin E (mg/L) | 7.46 (6.11–8.60) | 6.59 (4.86–7.67) | 5.54 (4.83–6.57) | z = 3.270; Δ = 1.829; 95% CI [0.733, 2.925]; p = 0.002 | z = 0.968; Δ = 0.491; 95% CI [-0.503, 1.485]; p = 0.333 | z = 4.117; Δ = 2.320; 95% CI [1.215, 3.424]; p < 0.001 | 9.30 (6.53–10.58) | U = 76.0; Δ = 0.896; 95% CI [-0.760, 2.660]; p = 0.283 | U = 55.5; Δ = 2.401; 95% CI [0.680, 4.100]; p = 0.019 | U = 45.5; Δ = 3.110; 95% CI [0.930, 4.890]; p = 0.007 |
| Copper (µg/dL) | 139.00 (105.35–152.45) | 103.00 (91.10–115.25) | 94.90 (88.76–113.73) | z = 4.146; Δ = 35.408; 95% CI [18.668, 52.148]; p < 0.001 | z = 0.594; Δ = 4.512; 95% CI [-10.387, 19.412]; p = 0.553 | z = 4.602; Δ = 39.920; 95% CI [22.917, 56.923]; p < 0.001 | 109.00 (91.95–113.80) | U = -81.0; Δ = -30.800; 95% CI [-47.600, -10.600]; p = 0.027 | U = -15.0; Δ = 2.225; 95% CI [-12.800, 19.800]; p = 0.672 | U = -22.0; Δ = 8.262; 95% CI [-10.200, 22.190]; p = 0.572 |
| Zinc (µg/dL) | 87.50 (58.25–96.00) | 78.00 (75.00–87.88) | 80.25 (69.88–88.00) | z = -1.532; Δ = -6.294; 95% CI [-14.347, 1.760]; p = 0.188 | z = 1.645; Δ = 7.130; 95% CI [-1.364, 15.623]; p = 0.188 | z = 0.206; Δ = 0.836; 95% CI [-7.135, 8.807]; p = 0.837 | 83.50 (74.57–87.12) | U = 22.0; Δ = -1.341; 95% CI [-10.500, 15.500]; p = 0.930 | U = -26.5; Δ = 0.100; 95% CI [-7.000, 8.000]; p = 0.930 | U = -28.5; Δ = 2.700; 95% CI [-5.350, 11.000]; p = 0.930 |
| Notes: Data are presented as mean ± SD or median (P25–P75). The n shown in the headers corresponds to T1=24. T2=18. T3=16 and Control Group = 20. Variables with an approximately normal distribution were analyzed using linear mixed-effects models. with t-statistics and degrees of freedom estimated by Satterthwaite's method. Variables with a non-normal distribution were analyzed using generalized linear mixed models. with z-statistics. Pairwise contrasts are reported as estimated differences (Δ) with 95% confidence intervals. pA = preoperative vs. 3 months; pB = 3 months vs. 6 months; pC = preoperative vs. 6 months. The variables with normal distribution and homogeneity of variance were analyzed using Student's t-test; in cases of heterogeneity of variance. Welch's t-test was applied. with the respective degrees of freedom indicated in the table. For variables with nonparametric distribution. the Mann-Whitney U test was used. Comparisons report Δ represents the Hodges–Lehmann estimator of the between-group difference and 95% confidence intervals when estimable. pD = Control vs. preoperative; pE = Control vs. 3 months; pF = Control vs. 6 months. Benjamini-Hochberg correction was applied within each variable across pA. pB. pC. pD. pE and pF. | | | | | | | | | | |

| **Supplementary Table S3.** Exploratory composite inflammatory-nutritional indices. ratios and scores comparing PD patients and the Control group. | | | | | | | | | | |
| --- | --- | --- | --- | --- | --- | --- | --- | --- | --- | --- |
| **Variable** | **Preoperative**  **(n=24)**  **Mean ± SD or Median**  **(P25–P75)** | **3 months**  **(n=18)**  **Mean ± SD or Median**  **(P25–P75)** | **6 months**  **(n=16)**  **Mean ± SD or Median**  **(P25–P75)** | **Statistical test**  **95% CI**  ***p^A^*** | **Statistical test**  **95% CI**  ***p^B^*** | **Statistical test**  **95% CI**  ***p^C^*** | **Control Group**  **(n=20)**  **Mean ± SD or Median**  **(P25–P75)** | **Statistical test**  **95% CI**  ***p^D^*** | **Statistical test**  **95% CI**  ***p^E^*** | **Statistical test**  **95% CI**  ***p^F^*** |
| Prognostic Nutritional Index | 50.68 ± 9.65 | 53.50 ± 7.46 | 53.79 ± 9.16 | t(38.2) = -1.035; Δ = -2.787;  95% CI [-8.236. 2.662];  p = 0.461 | t(35.7) = -0.094; Δ = -0.278; 95% CI [-6.270. 5.715];  p = 0.926 | t(39.5) = -1.095; Δ = -3.065; 95% CI [-8.723. 2.594];  p = 0.461 | 54.42 ± 3.64 | t(30.4) = 1.756; Δ = 3.742;  95% CI [-0.608. 8.091];  p = 0.268 | t(24.1) = 0.478; Δ = 0.925;  95% CI [-3.072. 4.922];  p = 0.797 | t(18.8) = 0.261; Δ = 0.634;  95% CI [-4.457. 5.725];  p = 0.797 |
| Glasgow Prognostic Score (GPS) | 1.00  (0.00–1.00) | 0.00  (0.00–0.00) | 0.00  (0.00–0.00) | z = 2.385; Δ = 1.776;  95% CI [0.317. 3.235];  p = 0.026 | z = 0.833; Δ = 1.008;  95% CI [-1.365. 3.382];  p = 0.405 | z = 2.510; Δ = 2.784;  95% CI [0.610. 4.958];  p = 0.026 | 0.00  (0.00–0.00) | U = -100.0; Δ = -1.000;  95% CI [-1.000. -0.000];  p < 0.001 | U = -60.0; Δ = 0.000;  95% CI [-0.000. 0.000];  p = 0.097 | U = -60.0; Δ = 0.000;  95% CI [0.000. 0.000];  p = 0.288 |
| Albumin/CRP ratio | 0.64  (0.24–1.64) | 5.80  (0.95–10.70) | 9.38  (2.53–10.77) | z = -2.812; Δ = -4.400;  95% CI [-7.466. -1.333];  p = 0.007 | z = -0.716; Δ = -1.706;  95% CI [-6.378. 2.966];  p = 0.474 | z = -2.861; Δ = -6.106; 95% CI [-10.289. -1.922];  p = 0.007 | 11.15  (10.01–11.36) | U = 245.0; Δ = 9.860;  95% CI [8.873. 10.679];  p < 0.001 | U = 61.0; Δ = 2.928;  95% CI [0.500. 9.089];  p = 0.012 | U = 19.0; Δ = 1.267;  95% CI [0.125. 5.712];  p = 0.029 |
| CRP/Albumin ratio | 1.66  (0.62–4.09) | 0.17  (0.09–1.09) | 0.11  (0.09–0.40) | z = 3.203; Δ = 2.245;  95% CI [0.871. 3.618];  p = 0.002 | z = 0.738; Δ = 0.135;  95% CI [-0.223. 0.492];  p = 0.461 | z = 3.376; Δ = 2.380; 95% CI [0.998. 3.761];  p = 0.002 | 0.09  (0.09–0.10) | U = -185.0; Δ = -1.454;  95% CI [-3.404. -1.044];  p < 0.001 | U = -121.0; Δ = -0.069;  95% CI [-0.655. -0.004];  p = 0.012 | U = -118.5; Δ = -0.014;  95% CI [-0.109. -0.001];  p = 0.030 |
| CRP/Lymphocytes ratio | 2.78  (1.26–8.90) | 0.37  (0.16–1.97) | 0.25  (0.14–0.67) | z = 2.973; Δ = 5.387;  95% CI [1.836. 8.938];  p = 0.004 | z = 0.313; Δ = 0.122;  95% CI [-0.642. 0.886];  p = 0.754 | z = 3.041; Δ = 5.509;  95% CI [1.958. 9.060];  p = 0.004 | 0.22  (0.17–0.29) | U = -166.0; Δ = -2.485;  95% CI [-6.974. -1.165];  p < 0.001 | U = -67.5; Δ = -0.121;  95% CI [-1.178. 0.039];  p = 0.418 | U = -57.0; Δ = -0.019;  95% CI [-0.233. 0.074];  p = 0.836 |
| Ferritin/Albumin ratio | 89.09  (25.41–214.13) | 58.99  (40.51–122.15) | 49.91  (29.62–106.34) | z = 2.530; Δ = 57.501;  95% CI [12.962. 102.041];  p = 0.017 | z = 0.905; Δ = 11.565;  95% CI [-13.484. 36.615];  p = 0.366 | z = 2.995; Δ = 69.067;  95% CI [23.864. 114.270];  p = 0.008 | 22.91  (10.76–39.41) | U = -111.0; Δ = -56.738;  95% CI [-151.422. -16.825];  p = 0.003 | U = -125.0; Δ = -36.804;  95% CI [-88.023. -11.822];  p = 0.009 | U = -124.0; Δ = -24.591;  95% CI [-54.986. -2.492];  p = 0.019 |
| Ferritin/Lymphocytes ratio | 227.27  (60.82–458.52) | 102.85  (66.60–157.20) | 98.43  (57.83–142.68) | z = 2.587; Δ = 173.580;  95% CI [42.097. 305.063];  p = 0.015 | z = 0.326; Δ = 11.276;  95% CI [-56.538. 79.089];  p = 0.745 | z = 2.727; Δ = 184.856;  95% CI [51.982. 317.730];  p = 0.015 | 53.19  (20.83–109.90) | U = -91.0; Δ = -122.728;  95% CI [-345.584. -26.807];  p = 0.014 | U = -100.0; Δ = -48.612;  95% CI [-98.303. -2.170];  p = 0.063 | U = -103.0; Δ = -37.942;  95% CI [-87.298. 8.608];  p = 0.095 |
| Albumin-Lymphocytes-CRP index | 1.36  (0.42–3.45) | 12.12  (1.95–28.45) | 17.40  (7.21–31.52) | z = -2.600; Δ = -11.327;  95% CI [-19.864. -2.789];  p = 0.014 | z = -0.642; Δ = -4.016;  95% CI [-16.282. 8.251];  p = 0.521 | z = -2.618; Δ = -15.342;  95% CI [-26.828. -3.857];  p = 0.014 | 20.30  (15.92–26.09) | U = 225.0; Δ = 17.319;  95% CI [13.563. 20.089];  p < 0.001 | U = 8.0; Δ = 5.944;  95% CI [-5.540. 15.011];  p = 0.409 | U = -41.0; Δ = 1.440;  95% CI [-8.488. 11.094];  p = 0.787 |
| Notes: Data are presented as mean ± SD or median (P25–P75). The n shown in the headers corresponds to T1=24. T2=18. T3=16 and Control Group = 20. Variables with an approximately normal distribution were analyzed using linear mixed-effects models. with t-statistics and degrees of freedom estimated by Satterthwaite's method. Variables with a non-normal distribution were analyzed using generalized linear mixed models. with z-statistics. Pairwise contrasts are reported as estimated differences (Δ) with 95% confidence intervals. *p^A^* = preoperative vs. 3 months; *p^B^* = 3 months vs. 6 months; *p^C^* = preoperative vs. 6 months. The variables with normal distribution and homogeneity of variance were analyzed using Student's t-test; in cases of heterogeneity of variance. Welch's t-test was applied. with the respective degrees of freedom indicated in the table. For variables with nonparametric distribution. the Mann-Whitney U test was used. Comparisons report Δ represents the Hodges–Lehmann estimator of the between-group difference and 95% confidence intervals when estimable. *p^D^* = Control vs. preoperative; *p^E^* = Control vs. 3 months; *p^F^* = Control vs. 6 months. Benjamini-Hochberg correction was applied within each variable across *p^A^. p^B^. p^C^. p^D^. p^E^* and *p^F^.* | | | | | | | | | | |

| **Supplementary Table S4.** Adjusted associations between food intake and clinical–nutritional biomarkers in the PD group and Control group. | | | | | | | | | | | | | | |
| --- | --- | --- | --- | --- | --- | --- | --- | --- | --- | --- | --- | --- | --- | --- |
|  |  | | | |  | **Preoperative**  **(n=24)** | | **3 months**  **(n=18)** | | **6 months**  **(n=16)** | | **Control Group**  **(n=20)** | | |
| **Intake variable** | | **Corresponding biomarker** | **Adjustment** | | | **β (95% CI).**  ***p*** | | **β (95% CI).**  ***p*** | | **β (95% CI).**  ***p*** | | **β (95% CI).**  ***p*** | | |
| Protein (g) | | Albumin (g/dL) | | CRP | | | 0.001  (-0.014; 0.017).  p = 0.848 | | 0.004  (-0.010; 0.019).  p = 0.549 | | 0.011  (-0.008; 0.031).  p = 0.226 | | -0.006  (-0.014; 0.001).  p = 0.104 |  |
|  |  | Total proteins (g/dL) | | CRP | | | 0.001  (-0.012; 0.013).  p = 0.896 | | 0.008  (-0.008; 0.024).  p = 0.282 | | 0.014  (-0.004; 0.031).  p = 0.115 | | 0.005  (-0.013; 0.023).  p = 0.548 |  |
| Iron (mg) | | Serum Iron (µg/dL) | | CRP | | | 1.178  (-1.681; 4.037).  p = 0.399 | | -0.006  (-3.694; 3.682).  p = 0.997 | | -0.269  (-3.930; 3.392).  p = 0.874 | | -2.508  (-8.423; 3.407).  p = 0.380 |  |
|  |  | Ferritin (ng/mL) | | CRP | | | 41.426  (-2.951; 85.802).  p = 0.066 | | 5.269  (-14.845; 25.384).  p = 0.581 | | 4.627  (-8.091; 17.344).  p = 0.440 | | -10.898  (-33.150; 11.355).  p = 0.313 |  |
|  |  | Hemoglobin (g/dL) | | CRP | | | 0.192  (0.047; 0.338).  p = 0.012 | | 0.053  (-0.051; 0.157).  p = 0.294 | | -0.038  (-0.226; 0.150).  p = 0.662 | | 0.061  (-0.225; 0.346).  p = 0.657 |  |
|  |  | UIBC (µg/dL) | | CRP | | | -2.058  (-7.254; 3.139).  p = 0.417 | | 0.836  (-2.752; 4.423).  p = 0.623 | | -0.747  (-3.459; 1.965).  p = 0.557 | | 1.093  (-6.351; 8.536).  p = 0.759 |  |
|  |  | TIBC (µg/dL) | | CRP | | | -0.880  (-7.346; 5.587).  p = 0.779 | | 0.830  (-4.938; 6.598).  p = 0.761 | | -1.016  (-5.564; 3.531).  p = 0.633 | | -1.416  (-10.655; 7.823).  p = 0.748 |  |
|  |  | Transferrin (mg/dL) | | CRP | | | -1.646  (-5.803; 2.511).  p = 0.417 | | 0.668  (-2.202; 3.539).  p = 0.623 | | -0.598  (-2.768; 1.572).  p = 0.557 | | 0.874  (-5.081; 6.829).  p = 0.759 |  |
|  |  | Transferrin Saturation (%) | | CRP | | | 0.396  (-0.183; 0.976).  p = 0.169 | | -0.038  (-0.677; 0.600).  p = 0.899 | | -0.028  (-0.754; 0.698).  p = 0.935 | | -0.468  (-1.683; 0.747).  p = 0.425 |  |
| Zinc (mg) | | Serum Zinc (µg/dL) | | CRP | | | -0.710  (-2.269; 0.849).  p = 0.353 | | -0.398  (-3.445; 2.649).  p = 0.782 | | -0.670  (-3.725; 2.384).  p = 0.639 | | -0.807  (-2.920; 1.306).  p = 0.428 |  |
| Vitamin A (mg) | | Vitamin A (µg/dL) | | CRP | | | -0.005  (-0.023; 0.013).  p = 0.561 | | -0.007  (-0.025; 0.011).  p = 0.418 | | 0.006  (-0.017; 0.030).  p = 0.567 | | -0.004  (-0.134; 0.126).  p = 0.949 |  |
| Vitamin E (mg) | | Vitamin E (mg/dL) | | CRP | | | 0.240  (-0.552; 1.033).  p = 0.533 | | 0.083  (-0.539; 0.705).  p = 0.778 | | 0.169  (-0.907; 1.246).  p = 0.736 | | -0.262  (-0.585; 0.061).  p = 0.105 |  |
|  | |  | |  | | |  | |  | |  | |  |  |
| Protein (g) | | Albumin (g/dL) | | Ferritin | | | -0.001  (-0.016; 0.015).  p = 0.917 | | 0.005  (-0.015; 0.025).  p = 0.617 | | 0.021  (0.007; 0.036).  p = 0.008 | | -0.007  (-0.015; 0.001).  p = 0.078 |  |
|  |  | Total proteins (g/dL) | | Ferritin | | | 0.002  (-0.011; 0.015).  p = 0.791 | | 0.008  (-0.007; 0.023).  p = 0.287 | | 0.016  (-0.000; 0.032).  p = 0.051 | | 0.006  (-0.011; 0.024).  p = 0.453 |  |
| Iron (mg) | | Serum Iron (µg/dL) | | Ferritin | | | 1.527  (-1.583; 4.637).  p = 0.317 | | 0.051  (-3.873; 3.974).  p = 0.978 | | 0.258  (-3.224; 3.739).  p = 0.874 | | -1.941  (-7.937; 4.054).  p = 0.501 |  |
|  |  | Ferritin (ng/mL) | | Ferritin | | | - | | - | | - | | - |  |
|  |  | Hemoglobin (g/dL) | | Ferritin | | | 0.193  (0.033; 0.353).  p = 0.021 | | 0.056  (-0.059; 0.171).  p = 0.314 | | -0.020  (-0.192; 0.152).  p = 0.807 | | 0.119  (-0.158; 0.397).  p = 0.374 |  |
|  |  | UIBC (µg/dL) | | Ferritin | | | -1.015  (-6.567; 4.537).  p = 0.706 | | 1.380  (-1.524; 4.283).  p = 0.323 | | 0.072  (-1.630; 1.774).  p = 0.928 | | 1.610  (-6.226; 9.447).  p = 0.668 |  |
|  |  | TIBC (µg/dL) | | Ferritin | | | 0.513  (-6.324; 7.349).  p = 0.877 | | 1.430  (-4.078; 6.939).  p = 0.584 | | 0.330  (-4.102; 4.762).  p = 0.873 | | -0.331  (-9.874; 9.213).  p = 0.942 |  |
|  |  | Transferrin (mg/dL) | | Ferritin | | | -0.812  (-5.253; 3.630).  p = 0.706 | | 1.104  (-1.219; 3.427).  p = 0.323 | | 0.058  (-1.304; 1.419).  p = 0.928 | | 1.288  (-4.981; 7.557).  p = 0.668 |  |
|  |  | Transferrin Saturation (%) | | Ferritin | | | 0.414  (-0.219; 1.047).  p = 0.187 | | -0.070  (-0.763; 0.624).  p = 0.832 | | 0.027  (-0.629; 0.682).  p = 0.931 | | -0.354  (-1.582; 0.874).  p = 0.548 |  |
| Zinc (mg) | | Serum Zinc (µg/dL) | | Ferritin | | | -0.137  (-2.115; 1.840).  p = 0.886 | | -1.010  (-4.202; 2.182).  p = 0.506 | | -0.370  (-3.262; 2.522).  p = 0.783 | | -0.929  (-3.052; 1.193).  p = 0.365 |  |
| Vitamin A (mg) | | Vitamin A (µg/dL) | | Ferritin | | | 0.000  (-0.019; 0.019).  p = 0.986 | | -0.002  (-0.018; 0.013).  p = 0.735 | | 0.006  (-0.017; 0.029).  p = 0.568 | | 0.004  (-0.125; 0.133).  p = 0.950 |  |
| Vitamin E (mg) | | Vitamin E (mg/dL) | | Ferritin | | | 0.213  (-0.584; 1.010).  p = 0.582 | | 0.112  (-0.448; 0.671).  p = 0.674 | | 0.079  (-0.955; 1.114).  p = 0.869 | | -0.257  (-0.488; -0.026).  p = 0.032 |  |
| Notes: Each model was adjusted for age. sex. and either CRP or ferritin. as indicated. Unstandardized linear regression coefficients (β) are shown with 95% confidence intervals and *p-values.* | | | | | | | | | | | | | | |

| **Supplementary Table S5.** Exploratory associations between Preoperative biliary drainage by ERCP and dietary intake, clinical-nutritional laboratory variables, and inflammatory-nutritional indices after pancreatoduodenectomy. | | | | | | | | |
| --- | --- | --- | --- | --- | --- | --- | --- | --- |
| **Outcome** | | | | **Preoperative biliary drainage by ERCP** | | | | |
| **Domain** | **Source row** | **Variable** | **Time** | **Yes** | **No** | **Statistical result** | **Raw p-value** | **BH-FDR adjusted p-value** |
|  |  |  |  | **Mean ± SD or Median (P25–P75), n** | **Mean ± SD or Median (P25–P75), n** |  |  |  |
| Dietary intake | 1 | Energy (kcal) | Preoperative | 2640.26 ± 628.58 (n=16) | 2857.40 ± 453.84 (n=8) | t(22)=-0.87; Δ_mean=-217.14; 95% IC [-736.85 ; 302.57] | 0,396 | 1,000 |
| Dietary intake | 2 | Energy (kcal) | 3 months after discharge | 2060.14 ± 337.71 (n=12) | 2000.53 ± 356.62 (n=6) | t(16)=0.35; Δ_mean=59.61; 95% IC [-304.72 ; 423.95] | 0,733 | 1,000 |
| Dietary intake | 3 | Energy (kcal) | 6 months after discharge | 1954.42 (1791.89–2144.14) (n=11) | 1942.33 (1850.60–2014.08) (n=5) | W=27.00; Δ_HL=-0.08; 95% IC [-472.65 ; 327.74] | 1,000 | 1,000 |
| Dietary intake | 4 | Protein (g) | Preoperative | 115.50 ± 26.96 (n=16) | 120.73 ± 13.44 (n=8) | t(22)=-0.51; Δ_mean=-5.22; 95% IC [-26.34 ; 15.89] | 0,613 | 1,000 |
| Dietary intake | 5 | Protein (g) | 3 months after discharge | 98.05 (86.27–106.99) (n=12) | 89.34 (88.50–96.50) (n=6) | W=41.00; Δ_HL=3.52; 95% IC [-11.73 ; 18.32] | 0,673 | 1,000 |
| Dietary intake | 6 | Protein (g) | 6 months after discharge | 101.56 ± 17.35 (n=11) | 99.92 ± 12.61 (n=5) | t(14)=0.19; Δ_mean=1.64; 95% IC [-17.03 ; 20.31] | 0,853 | 1,000 |
| Dietary intake | 7 | Relative energy balance (kcal) | Preoperative | 1168.25 ± 306.91 (n=16) | 1005.15 ± 338.60 (n=8) | t(22)=1.19; Δ_mean=163.11; 95% IC [-121.86 ; 448.08] | 0,248 | 1,000 |
| Dietary intake | 8 | Relative energy balance (kcal) | 3 months after discharge | 610.89 ± 282.55 (n=12) | 448.32 ± 89.70 (n=6) | t(16)=1.36; Δ_mean=162.57; 95% IC [-91.37 ; 416.52] | 0,194 | 1,000 |
| Dietary intake | 9 | Relative energy balance (kcal) | 6 months after discharge | 614.40 (553.98–779.19) (n=11) | 614.48 (483.15–672.48) (n=5) | W=34.00; Δ_HL=78.72; 95% IC [-94.15 ; 475.31] | 0,497 | 1,000 |
| Dietary intake | 10 | Carbohydrate (g) | Preoperative | 364.56 ± 86.48 (n=16) | 397.71 ± 60.81 (n=8) | t(22)=-0.97; Δ_mean=-33.15; 95% IC [-104.29 ; 37.99] | 0,344 | 1,000 |
| Dietary intake | 11 | Carbohydrate (g) | 3 months after discharge | 263.89 ± 40.22 (n=12) | 262.64 ± 43.35 (n=6) | t(16)=0.06; Δ_mean=1.25; 95% IC [-42.44 ; 44.95] | 0,952 | 1,000 |
| Dietary intake | 12 | Carbohydrate (g) | 6 months after discharge | 257.88 ± 43.48 (n=11) | 271.19 ± 29.73 (n=5) | t(14)=-0.62; Δ_mean=-13.32; 95% IC [-59.64 ; 33.00] | 0,547 | 1,000 |
| Dietary intake | 13 | Lipid (g) | Preoperative | 80.00 ± 25.58 (n=16) | 87.07 ± 21.44 (n=8) | t(22)=-0.67; Δ_mean=-7.07; 95% IC [-28.93 ; 14.79] | 0,509 | 1,000 |
| Dietary intake | 14 | Lipid (g) | 3 months after discharge | 67.47 (60.01–78.56) (n=12) | 57.41 (56.67–61.99) (n=6) | W=45.00; Δ_HL=7.59; 95% IC [-14.54 ; 24.31] | 0,426 | 1,000 |
| Dietary intake | 15 | Lipid (g) | 6 months after discharge | 61.81 ± 18.22 (n=11) | 62.02 ± 16.87 (n=5) | t(14)=-0.02; Δ_mean=-0.21; 95% IC [-20.85 ; 20.43] | 0,983 | 1,000 |
| Dietary intake | 16 | Fiber (g) | Preoperative | 45.90 ± 11.37 (n=16) | 46.29 ± 11.95 (n=8) | t(22)=-0.08; Δ_mean=-0.39; 95% IC [-10.77 ; 9.99] | 0,939 | 1,000 |
| Dietary intake | 17 | Fiber (g) | 3 months after discharge | 39.05 ± 9.10 (n=12) | 36.31 ± 9.99 (n=6) | t(16)=0.58; Δ_mean=2.74; 95% IC [-7.21 ; 12.69] | 0,567 | 1,000 |
| Dietary intake | 18 | Fiber (g) | 6 months after discharge | 37.35 ± 8.94 (n=11) | 36.11 ± 9.31 (n=5) | t(14)=0.26; Δ_mean=1.25; 95% IC [-9.22 ; 11.72] | 0,802 | 1,000 |
| Dietary intake | 19 | Calcium (mg) | Preoperative | 1523.35 ± 334.23 (n=16) | 1379.28 ± 254.39 (n=8) | t(22)=1.07; Δ_mean=144.07; 95% IC [-135.26 ; 423.40] | 0,296 | 1,000 |
| Dietary intake | 20 | Calcium (mg) | 3 months after discharge | 1367.79 (1011.59–1534.45) (n=12) | 1165.10 (1055.54–1271.27) (n=6) | W=40.00; Δ_HL=52.40; 95% IC [-412.41 ; 464.15] | 0,743 | 1,000 |
| Dietary intake | 21 | Calcium (mg) | 6 months after discharge | 1258.11 (975.54–1457.99) (n=11) | 1068.66 (1062.21–1272.90) (n=5) | W=24.00; Δ_HL=-42.85; 95% IC [-494.95 ; 408.65] | 0,734 | 1,000 |
| Dietary intake | 22 | Iron (mg) | Preoperative | 25.75 (20.94–29.09) (n=16) | 27.05 (25.32–28.77) (n=8) | W=52.00; Δ_HL=-1.63; 95% IC [-7.48 ; 3.94] | 0,481 | 1,000 |
| Dietary intake | 23 | Iron (mg) | 3 months after discharge | 22.40 ± 5.88 (n=12) | 26.88 ± 12.11 (n=6) | t(16)=-1.07; Δ_mean=-4.47; 95% IC [-13.31 ; 4.37] | 0,299 | 1,000 |
| Dietary intake | 24 | Iron (mg) | 6 months after discharge | 20.74 ± 4.29 (n=11) | 28.39 ± 13.46 (n=5) | t(14)=-1.76; Δ_mean=-7.65; 95% IC [-16.97 ; 1.67] | 0,100 | 1,000 |
| Dietary intake | 25 | Potassium (mg) | Preoperative | 6668.05 ± 1508.38 (n=16) | 6756.17 ± 896.02 (n=8) | t(22)=-0.15; Δ_mean=-88.11; 95% IC [-1295.17 ; 1118.94] | 0,881 | 1,000 |
| Dietary intake | 26 | Potassium (mg) | 3 months after discharge | 5803.93 ± 1370.98 (n=12) | 5580.78 ± 1133.63 (n=6) | t(16)=0.34; Δ_mean=223.14; 95% IC [-1156.35 ; 1602.64] | 0,736 | 1,000 |
| Dietary intake | 27 | Potassium (mg) | 6 months after discharge | 5513.25 ± 1295.59 (n=11) | 5656.03 ± 907.96 (n=5) | t(14)=-0.22; Δ_mean=-142.79; 95% IC [-1528.31 ; 1242.74] | 0,828 | 1,000 |
| Dietary intake | 28 | Selenium (µg) | Preoperative | 57.57 ± 18.38 (n=16) | 57.33 ± 22.67 (n=8) | t(22)=0.03; Δ_mean=0.24; 95% IC [-17.58 ; 18.07] | 0,978 | 1,000 |
| Dietary intake | 29 | Selenium (µg) | 3 months after discharge | 47.06 ± 19.25 (n=12) | 50.18 ± 25.45 (n=6) | t(16)=-0.29; Δ_mean=-3.13; 95% IC [-25.79 ; 19.53] | 0,774 | 1,000 |
| Dietary intake | 30 | Selenium (µg) | 6 months after discharge | 45.33 ± 18.01 (n=11) | 51.66 ± 24.90 (n=5) | t(14)=-0.58; Δ_mean=-6.33; 95% IC [-29.72 ; 17.06] | 0,571 | 1,000 |
| Dietary intake | 31 | Zinc (mg) | Preoperative | 17.24 (12.45–18.26) (n=16) | 17.95 (16.55–19.93) (n=8) | W=50.00; Δ_HL=-1.43; 95% IC [-5.98 ; 2.21] | 0,408 | 1,000 |
| Dietary intake | 32 | Zinc (mg) | 3 months after discharge | 14.77 ± 5.09 (n=12) | 14.43 ± 3.97 (n=6) | t(16)=0.14; Δ_mean=0.34; 95% IC [-4.71 ; 5.39] | 0,889 | 1,000 |
| Dietary intake | 33 | Zinc (mg) | 6 months after discharge | 13.36 ± 3.51 (n=11) | 14.75 ± 3.52 (n=5) | t(14)=-0.73; Δ_mean=-1.39; 95% IC [-5.45 ; 2.67] | 0,475 | 1,000 |
| Dietary intake | 34 | Sodium (mg) | Preoperative | 1611.20 (1224.73–2392.67) (n=16) | 1807.37 (1448.59–2715.27) (n=8) | W=49.00; Δ_HL=-300.19; 95% IC [-1162.39 ; 364.44] | 0,375 | 1,000 |
| Dietary intake | 35 | Sodium (mg) | 3 months after discharge | 1330.87 ± 335.63 (n=12) | 1441.56 ± 591.52 (n=6) | t(16)=-0.51; Δ_mean=-110.69; 95% IC [-568.79 ; 347.41] | 0,615 | 1,000 |
| Dietary intake | 36 | Sodium (mg) | 6 months after discharge | 1264.84 ± 328.23 (n=11) | 1443.70 ± 632.65 (n=5) | t(14)=-0.76; Δ_mean=-178.86; 95% IC [-684.84 ; 327.12] | 0,461 | 1,000 |
| Dietary intake | 37 | Vitamin A (mg) | Preoperative | 6.31 ± 2.77 (n=16) | 4.08 ± 2.49 (n=8) | t(22)=1.92; Δ_mean=2.23; 95% IC [-0.18 ; 4.64] | 0,069 | 1,000 |
| Dietary intake | 38 | Vitamin A (mg) | 3 months after discharge | 4.43 (3.43–8.42) (n=12) | 4.24 (2.90–6.34) (n=6) | W=45.00; Δ_HL=0.61; 95% IC [-2.15 ; 5.38] | 0,426 | 1,000 |
| Dietary intake | 39 | Vitamin A (mg) | 6 months after discharge | 4.54 (3.13–6.72) (n=11) | 4.76 (2.56–6.66) (n=5) | W=29.00; Δ_HL=0.23; 95% IC [-2.72 ; 4.89] | 0,910 | 1,000 |
| Dietary intake | 40 | Vitamin C (mg) | Preoperative | 426.19 ± 200.89 (n=16) | 298.09 ± 162.26 (n=8) | t(22)=1.56; Δ_mean=128.10; 95% IC [-42.04 ; 298.23] | 0,133 | 1,000 |
| Dietary intake | 41 | Vitamin C (mg) | 3 months after discharge | 348.52 (237.38–633.89) (n=12) | 242.85 (210.74–321.44) (n=6) | W=46.00; Δ_HL=77.83; 95% IC [-73.46 ; 388.60] | 0,374 | 1,000 |
| Dietary intake | 42 | Vitamin C (mg) | 6 months after discharge | 367.05 ± 191.29 (n=11) | 296.41 ± 103.52 (n=5) | t(14)=0.77; Δ_mean=70.64; 95% IC [-127.03 ; 268.31] | 0,456 | 1,000 |
| Dietary intake | 43 | Vitamin E (mg) | Preoperative | 4.93 ± 1.68 (n=16) | 3.85 ± 1.54 (n=8) | t(22)=1.53; Δ_mean=1.08; 95% IC [-0.39 ; 2.55] | 0,141 | 1,000 |
| Dietary intake | 44 | Vitamin E (mg) | 3 months after discharge | 4.63 ± 2.41 (n=12) | 4.43 ± 2.17 (n=6) | t(16)=0.17; Δ_mean=0.20; 95% IC [-2.28 ; 2.67] | 0,867 | 1,000 |
| Dietary intake | 45 | Vitamin E (mg) | 6 months after discharge | 4.23 ± 2.10 (n=11) | 3.59 ± 2.21 (n=5) | t(14)=0.56; Δ_mean=0.64; 95% IC [-1.83 ; 3.10] | 0,587 | 1,000 |
| Clinical–Nutritional Laboratory Analyses | 46 | Total Proteins (g/dL) | Preoperative | 6.87 ± 0.62 (n=16) | 7.00 ± 0.44 (n=8) | t(22)=-0.53; Δ_mean=-0.13; 95% IC [-0.64 ; 0.38] | 0,604 | 1,000 |
| Clinical–Nutritional Laboratory Analyses | 47 | Total Proteins (g/dL) | 3 months after discharge after discharge | 6.92 ± 0.52 (n=12) | 6.79 ± 0.39 (n=6) | t(16)=0.53; Δ_mean=0.13; 95% IC [-0.39 ; 0.64] | 0,604 | 1,000 |
| Clinical–Nutritional Laboratory Analyses | 48 | Total Proteins (g/dL) | 6 months after discharge after discharge | 6.66 ± 0.51 (n=11) | 6.63 ± 0.56 (n=5) | t(14)=0.12; Δ_mean=0.03; 95% IC [-0.57 ; 0.64] | 0,905 | 1,000 |
| Clinical–Nutritional Laboratory Analyses | 49 | Albumin (g/dL) | Preoperative | 3.76 ± 0.61 (n=16) | 4.33 ± 0.44 (n=8) | t(22)=-2.32; Δ_mean=-0.56; 95% IC [-1.07 ; -0.06] | 0,030 | 0,927 |
| Clinical–Nutritional Laboratory Analyses | 50 | Albumin (g/dL) | 3 months after discharge after discharge | 4.07 ± 0.52 (n=12) | 4.17 ± 0.56 (n=6) | t(16)=-0.35; Δ_mean=-0.09; 95% IC [-0.66 ; 0.47] | 0,732 | 1,000 |
| Clinical–Nutritional Laboratory Analyses | 51 | Albumin (g/dL) | 6 months after discharge after discharge | 4.32 ± 0.31 (n=11) | 3.86 ± 1.11 (n=5) | t(4.29)=0.91; Δ_mean=0.46; 95% IC [-0.90 ; 1.82] | 0,411 | 1,000 |
| Clinical–Nutritional Laboratory Analyses | 52 | C-Reactive Protein (mg/L) | Preoperative | 7.75 (4.33–17.03) (n=16) | 4.08 (1.76–15.52) (n=8) | W=71.50; Δ_HL=1.81; 95% IC [-7.43 ; 8.22] | 0,668 | 1,000 |
| Clinical–Nutritional Laboratory Analyses | 53 | C-Reactive Protein (mg/L) | 3 months after discharge after discharge | 0.77 (0.40–4.23) (n=12) | 1.08 (0.40–3.02) (n=6) | W=37.50; Δ_HL=0.00; 95% IC [-2.66 ; 2.88] | 0,922 | 1,000 |
| Clinical–Nutritional Laboratory Analyses | 54 | C-Reactive Protein (mg/L) | 6 months after discharge after discharge | 0.40 (0.40–1.19) (n=11) | 0.40 (0.40–3.44) (n=5) | W=25.00; Δ_HL=-0.00; 95% IC [-9.26 ; 0.33] | 0,803 | 1,000 |
| Clinical–Nutritional Laboratory Analyses | 55 | Blood Glucose (mg/dL) | Preoperative | 116.54 (90.62–152.60) (n=16) | 125.50 (99.79–159.00) (n=8) | W=57.00; Δ_HL=-7.37; 95% IC [-47.51 ; 39.00] | 0,691 | 1,000 |
| Clinical–Nutritional Laboratory Analyses | 56 | Blood Glucose (mg/dL) | 3 months after discharge after discharge | 119.81 (84.74–134.19) (n=12) | 109.68 (85.98–149.81) (n=6) | W=34.00; Δ_HL=-6.00; 95% IC [-47.45 ; 42.82] | 0,888 | 1,000 |
| Clinical–Nutritional Laboratory Analyses | 57 | Blood Glucose (mg/dL) | 6 months after discharge after discharge | 106.64 (79.07–119.55) (n=11) | 116.00 (91.79–119.00) (n=5) | W=24.00; Δ_HL=-1.26; 95% IC [-38.45 ; 38.68] | 0,734 | 1,000 |
| Clinical–Nutritional Laboratory Analyses | 58 | Hemoglobin (g/dL) | Preoperative | 12.25 ± 2.30 (n=16) | 13.36 ± 2.11 (n=8) | t(22)=-1.15; Δ_mean=-1.11; 95% IC [-3.12 ; 0.90] | 0,263 | 0,927 |
| Clinical–Nutritional Laboratory Analyses | 59 | Hemoglobin (g/dL) | 3 months after discharge after discharge | 12.15 ± 1.81 (n=12) | 13.38 ± 0.66 (n=6) | t(15.22)=-2.09; Δ_mean=-1.23; 95% IC [-2.48 ; 0.02] | 0,054 | 0,927 |
| Clinical–Nutritional Laboratory Analyses | 60 | Hemoglobin (g/dL) | 6 months after discharge after discharge | 12.22 ± 2.44 (n=11) | 12.38 ± 1.67 (n=5) | t(14)=-0.13; Δ_mean=-0.16; 95% IC [-2.76 ; 2.44] | 0,896 | 1,000 |
| Clinical–Nutritional Laboratory Analyses | 61 | Lymphocytes (x10³/mm³) | Preoperative | 2.39 ± 1.34 (n=16) | 1.91 ± 1.01 (n=8) | t(22)=0.89; Δ_mean=0.48; 95% IC [-0.64 ; 1.60] | 0,382 | 1,000 |
| Clinical–Nutritional Laboratory Analyses | 62 | Lymphocytes (x10³/mm³) | 3 months after discharge after discharge | 2.44 ± 1.10 (n=12) | 2.60 ± 0.52 (n=6) | t(16)=-0.33; Δ_mean=-0.16; 95% IC [-1.17 ; 0.85] | 0,744 | 1,000 |
| Clinical–Nutritional Laboratory Analyses | 63 | Lymphocytes (x10³/mm³) | 6 months after discharge after discharge | 2.37 ± 1.10 (n=11) | 2.48 ± 0.98 (n=5) | t(14)=-0.18; Δ_mean=-0.11; 95% IC [-1.34 ; 1.13] | 0,856 | 1,000 |
| Clinical–Nutritional Laboratory Analyses | 64 | Serum Iron (µg/dL) | Preoperative | 81.72 ± 36.44 (n=16) | 98.76 ± 41.53 (n=8) | t(22)=-1.03; Δ_mean=-17.03; 95% IC [-51.27 ; 17.21] | 0,313 | 1,000 |
| Clinical–Nutritional Laboratory Analyses | 65 | Serum Iron (µg/dL) | 3 months after discharge after discharge | 92.05 ± 58.90 (n=12) | 103.81 ± 50.64 (n=6) | t(16)=-0.42; Δ_mean=-11.75; 95% IC [-71.59 ; 48.09] | 0,683 | 1,000 |
| Clinical–Nutritional Laboratory Analyses | 66 | Serum Iron (µg/dL) | 6 months after discharge after discharge | 88.67 ± 51.53 (n=11) | 88.55 ± 8.24 (n=5) | t(11.07)=0.01; Δ_mean=0.12; 95% IC [-35.00 ; 35.24] | 0,994 | 1,000 |
| Clinical–Nutritional Laboratory Analyses | 67 | Ferritin (ng/mL) | Preoperative | 306.00 (80.25–954.97) (n=16) | 399.25 (250.10–788.67) (n=8) | W=48.50; Δ_HL=-149.37; 95% IC [-458.70 ; 504.70] | 0,358 | 1,000 |
| Clinical–Nutritional Laboratory Analyses | 68 | Ferritin (ng/mL) | 3 months after discharge after discharge | 244.45 (180.97–455.37) (n=12) | 291.75 (69.75–515.62) (n=6) | W=40.00; Δ_HL=39.95; 95% IC [-354.50 ; 373.40] | 0,743 | 1,000 |
| Clinical–Nutritional Laboratory Analyses | 69 | Ferritin (ng/mL) | 6 months after discharge after discharge | 248.18 ± 152.73 (n=11) | 317.00 ± 285.15 (n=5) | t(14)=-0.64; Δ_mean=-68.82; 95% IC [-299.88 ; 162.23] | 0,533 | 1,000 |
| Clinical–Nutritional Laboratory Analyses | 70 | UIBC (µg/dL) | Preoperative | 275.91 ± 71.51 (n=16) | 311.26 ± 67.45 (n=8) | t(22)=-1.16; Δ_mean=-35.36; 95% IC [-98.44 ; 27.72] | 0,257 | 0,927 |
| Clinical–Nutritional Laboratory Analyses | 71 | UIBC (µg/dL) | 3 months after discharge after discharge | 291.17 (276.77–303.73) (n=12) | 315.00 (302.14–377.29) (n=6) | W=17.00; Δ_HL=-31.00; 95% IC [-116.97 ; 5.35] | 0,083 | 0,927 |
| Clinical–Nutritional Laboratory Analyses | 72 | UIBC (µg/dL) | 6 months after discharge after discharge | 293.09 ± 24.26 (n=11) | 319.48 ± 61.70 (n=5) | t(14)=-1.26; Δ_mean=-26.39; 95% IC [-71.31 ; 18.54] | 0,228 | 0,927 |
| Clinical–Nutritional Laboratory Analyses | 73 | TIBC (µg/dL) | Preoperative | 357.63 ± 86.22 (n=16) | 410.02 ± 76.77 (n=8) | t(22)=-1.45; Δ_mean=-52.39; 95% IC [-127.22 ; 22.44] | 0,161 | 0,927 |
| Clinical–Nutritional Laboratory Analyses | 74 | TIBC (µg/dL) | 3 months after discharge after discharge | 373.99 ± 84.86 (n=12) | 435.70 ± 100.16 (n=6) | t(16)=-1.37; Δ_mean=-61.72; 95% IC [-157.03 ; 33.59] | 0,189 | 0,927 |
| Clinical–Nutritional Laboratory Analyses | 75 | TIBC (µg/dL) | 6 months after discharge after discharge | 381.76 ± 60.23 (n=11) | 408.03 ± 66.83 (n=5) | t(14)=-0.78; Δ_mean=-26.27; 95% IC [-98.21 ; 45.67] | 0,447 | 1,000 |
| Clinical–Nutritional Laboratory Analyses | 76 | Transferrin (mg/dL) | Preoperative | 220.72 ± 57.20 (n=16) | 249.01 ± 53.96 (n=8) | t(22)=-1.16; Δ_mean=-28.29; 95% IC [-78.75 ; 22.17] | 0,257 | 0,927 |
| Clinical–Nutritional Laboratory Analyses | 77 | Transferrin (mg/dL) | 3 months after discharge after discharge | 232.94 (221.42–242.99) (n=12) | 252.00 (241.71–301.83) (n=6) | W=17.00; Δ_HL=-24.80; 95% IC [-93.58 ; 4.28] | 0,083 | 0,927 |
| Clinical–Nutritional Laboratory Analyses | 78 | Transferrin (mg/dL) | 6 months after discharge after discharge | 234.47 ± 19.40 (n=11) | 255.58 ± 49.36 (n=5) | t(14)=-1.26; Δ_mean=-21.11; 95% IC [-57.05 ; 14.83] | 0,228 | 0,927 |
| Clinical–Nutritional Laboratory Analyses | 79 | Transferrin Saturation (%) | Preoperative | 22.65 ± 7.67 (n=16) | 23.91 ± 9.36 (n=8) | t(22)=-0.35; Δ_mean=-1.25; 95% IC [-8.66 ; 6.15] | 0,729 | 1,000 |
| Clinical–Nutritional Laboratory Analyses | 80 | Transferrin Saturation (%) | 3 months after discharge after discharge | 22.72 ± 10.34 (n=12) | 23.31 ± 6.82 (n=6) | t(16)=-0.13; Δ_mean=-0.60; 95% IC [-10.54 ; 9.35] | 0,901 | 1,000 |
| Clinical–Nutritional Laboratory Analyses | 81 | Transferrin Saturation (%) | 6 months after discharge after discharge | 21.98 ± 9.89 (n=11) | 21.98 ± 2.60 (n=5) | t(14)=-0.00; Δ_mean=-0.01; 95% IC [-9.81 ; 9.80] | 0,999 | 1,000 |
| Clinical–Nutritional Laboratory Analyses | 82 | Urea (mg/dL) | Preoperative | 35.25 (27.94–44.71) (n=16) | 29.11 (22.64–39.31) (n=8) | W=77.00; Δ_HL=5.13; 95% IC [-5.95 ; 19.78] | 0,444 | 1,000 |
| Clinical–Nutritional Laboratory Analyses | 83 | Urea (mg/dL) | 3 months after discharge after discharge | 38.08 ± 18.43 (n=12) | 36.65 ± 9.01 (n=6) | t(16)=0.18; Δ_mean=1.43; 95% IC [-15.62 ; 18.48] | 0,861 | 1,000 |
| Clinical–Nutritional Laboratory Analyses | 84 | Urea (mg/dL) | 6 months after discharge after discharge | 32.73 (24.13–36.99) (n=11) | 25.14 (22.30–48.54) (n=5) | W=29.00; Δ_HL=0.47; 95% IC [-89.21 ; 18.69] | 0,910 | 1,000 |
| Clinical–Nutritional Laboratory Analyses | 85 | Creatinine (mg/dL) | Preoperative | 0.93 (0.83–1.19) (n=16) | 0.92 (0.75–1.02) (n=8) | W=72.50; Δ_HL=0.08; 95% IC [-0.19 ; 0.42] | 0,624 | 1,000 |
| Clinical–Nutritional Laboratory Analyses | 86 | Creatinine (mg/dL) | 3 months after discharge after discharge | 0.84 (0.75–1.01) (n=12) | 0.84 (0.75–0.91) (n=6) | W=42.00; Δ_HL=0.04; 95% IC [-0.15 ; 0.31] | 0,606 | 1,000 |
| Clinical–Nutritional Laboratory Analyses | 87 | Creatinine (mg/dL) | 6 months after discharge after discharge | 0.95 (0.76–1.10) (n=11) | 0.87 (0.86–0.87) (n=5) | W=27.00; Δ_HL=-0.00; 95% IC [-1.61 ; 0.29] | 1,000 | 1,000 |
| Clinical–Nutritional Laboratory Analyses | 88 | Aspartate Aminotransferase (AST) (U/L) | Preoperative | 148.37 ± 116.77 (n=16) | 224.03 ± 179.59 (n=8) | t(22)=-1.25; Δ_mean=-75.66; 95% IC [-201.25 ; 49.93] | 0,225 | 0,927 |
| Clinical–Nutritional Laboratory Analyses | 89 | Aspartate Aminotransferase (AST) (U/L) | 3 months after discharge after discharge | 26.90 (22.59–38.05) (n=12) | 32.61 (25.25–36.54) (n=6) | W=33.00; Δ_HL=-2.37; 95% IC [-11.95 ; 14.67] | 0,815 | 1,000 |
| Clinical–Nutritional Laboratory Analyses | 90 | Aspartate Aminotransferase (AST) (U/L) | 6 months after discharge after discharge | 30.67 ± 7.60 (n=11) | 40.72 ± 17.08 (n=5) | t(14)=-1.67; Δ_mean=-10.05; 95% IC [-22.97 ; 2.86] | 0,117 | 0,927 |
| Clinical–Nutritional Laboratory Analyses | 91 | Aminotransferase (ALT) (U/L) | Preoperative | 121.31 (54.77–204.35) (n=16) | 252.91 (131.86–497.41) (n=8) | W=38.00; Δ_HL=-116.38; 95% IC [-398.14 ; 13.33] | 0,118 | 0,927 |
| Clinical–Nutritional Laboratory Analyses | 92 | Aminotransferase (ALT) (U/L) | 3 months after discharge after discharge | 33.36 ± 16.09 (n=12) | 40.41 ± 13.18 (n=6) | t(16)=-0.93; Δ_mean=-7.05; 95% IC [-23.21 ; 9.10] | 0,368 | 1,000 |
| Clinical–Nutritional Laboratory Analyses | 93 | Aminotransferase (ALT) (U/L) | 6 months after discharge after discharge | 34.89 ± 12.24 (n=11) | 42.81 ± 26.58 (n=5) | t(14)=-0.84; Δ_mean=-7.93; 95% IC [-28.25 ; 12.40] | 0,417 | 1,000 |
| Clinical–Nutritional Laboratory Analyses | 94 | Folic Acid (ng/mL) | Preoperative | 14.44 ± 6.92 (n=16) | 14.77 ± 4.12 (n=8) | t(22)=-0.12; Δ_mean=-0.33; 95% IC [-5.87 ; 5.21] | 0,902 | 1,000 |
| Clinical–Nutritional Laboratory Analyses | 95 | Folic Acid (ng/mL) | 3 months after discharge after discharge | 14.71 (10.24–22.96) (n=12) | 24.00 (17.05–24.00) (n=6) | W=21.00; Δ_HL=-4.01; 95% IC [-13.38 ; 0.34] | 0,162 | 0,927 |
| Clinical–Nutritional Laboratory Analyses | 96 | Folic Acid (ng/mL) | 6 months after discharge after discharge | 21.56 (14.77–23.83) (n=11) | 20.87 (14.52–24.00) (n=5) | W=28.00; Δ_HL=0.00; 95% IC [-7.49 ; 9.48] | 1,000 | 1,000 |
| Clinical–Nutritional Laboratory Analyses | 97 | Vitamin B12 (pg/mL) | Preoperative | 607.00 (443.75–720.00) (n=16) | 435.00 (387.00–523.00) (n=8) | W=87.00; Δ_HL=137.00; 95% IC [-46.00 ; 370.00] | 0,167 | 0,927 |
| Clinical–Nutritional Laboratory Analyses | 98 | Vitamin B12 (pg/mL) | 3 months after discharge after discharge | 460.00 (382.50–1058.25) (n=12) | 512.50 (356.50–573.25) (n=6) | W=45.00; Δ_HL=127.26; 95% IC [-160.00 ; 779.00] | 0,426 | 1,000 |
| Clinical–Nutritional Laboratory Analyses | 99 | Vitamin B12 (pg/mL) | 6 months after discharge after discharge | 379.00 (325.65–523.00) (n=11) | 510.94 (313.00–585.00) (n=5) | W=28.50; Δ_HL=3.25; 95% IC [-249.94 ; 368.73] | 0,955 | 1,000 |
| Clinical–Nutritional Laboratory Analyses | 100 | Vitamin A (µg/dL) | Preoperative | 0.30 (0.19–0.34) (n=16) | 0.31 (0.22–0.41) (n=8) | W=45.50; Δ_HL=-0.03; 95% IC [-0.20 ; 0.06] | 0,267 | 0,927 |
| Clinical–Nutritional Laboratory Analyses | 101 | Vitamin A (µg/dL) | 3 months after discharge after discharge | 0.29 ± 0.09 (n=12) | 0.38 ± 0.12 (n=6) | t(16)=-1.76; Δ_mean=-0.09; 95% IC [-0.20 ; 0.02] | 0,098 | 0,927 |
| Clinical–Nutritional Laboratory Analyses | 102 | Vitamin A (µg/dL) | 6 months after discharge after discharge | 0.28 (0.27–0.36) (n=11) | 0.34 (0.29–0.36) (n=5) | W=23.00; Δ_HL=-0.02; 95% IC [-0.10 ; 0.18] | 0,649 | 1,000 |
| Clinical–Nutritional Laboratory Analyses | 103 | Vitamin E (mg/dL) | Preoperative | 7.37 (6.07–8.51) (n=16) | 7.79 (6.95–9.03) (n=8) | W=54.50; Δ_HL=-0.22; 95% IC [-2.31 ; 1.49] | 0,580 | 1,000 |
| Clinical–Nutritional Laboratory Analyses | 104 | Vitamin E (mg/dL) | 3 months after discharge after discharge | 6.58 ± 2.38 (n=12) | 6.79 ± 2.06 (n=6) | t(16)=-0.19; Δ_mean=-0.22; 95% IC [-2.64 ; 2.21] | 0,852 | 1,000 |
| Clinical–Nutritional Laboratory Analyses | 105 | Vitamin E (mg/dL) | 6 months after discharge after discharge | 5.43 (4.49–7.86) (n=11) | 5.83 (4.88–6.00) (n=5) | W=27.50; Δ_HL=-0.00; 95% IC [-1.64 ; 4.33] | 1,000 | 1,000 |
| Clinical–Nutritional Laboratory Analyses | 106 | Copper (µg/dL) | Preoperative | 139.00 (102.25–151.40) (n=16) | 144.20 (129.20–157.30) (n=8) | W=49.50; Δ_HL=-11.00; 95% IC [-53.20 ; 18.20] | 0,390 | 1,000 |
| Clinical–Nutritional Laboratory Analyses | 107 | Copper (µg/dL) | 3 months after discharge after discharge | 108.88 ± 36.61 (n=12) | 100.07 ± 26.30 (n=6) | t(16)=0.52; Δ_mean=8.82; 95% IC [-26.93 ; 44.56] | 0,608 | 1,000 |
| Clinical–Nutritional Laboratory Analyses | 108 | Copper (µg/dL) | 6 months after discharge after discharge | 100.45 ± 32.02 (n=11) | 103.82 ± 25.88 (n=5) | t(14)=-0.21; Δ_mean=-3.37; 95% IC [-38.53 ; 31.80] | 0,840 | 1,000 |
| Clinical–Nutritional Laboratory Analyses | 109 | Zinc (µg/dL) | Preoperative | 82.25 (63.50–93.75) (n=16) | 91.50 (57.75–98.62) (n=8) | W=54.00; Δ_HL=-3.59; 95% IC [-24.00 ; 12.00] | 0,559 | 1,000 |
| Clinical–Nutritional Laboratory Analyses | 110 | Zinc (µg/dL) | 3 months after discharge after discharge | 81.50 (76.50–89.38) (n=12) | 72.75 (68.75–83.12) (n=6) | W=50.50; Δ_HL=8.04; 95% IC [-9.50 ; 18.50] | 0,189 | 0,927 |
| Clinical–Nutritional Laboratory Analyses | 111 | Zinc (µg/dL) | 6 months after discharge after discharge | 79.98 ± 12.54 (n=11) | 74.10 ± 18.94 (n=5) | t(14)=0.74; Δ_mean=5.88; 95% IC [-11.07 ; 22.83] | 0,469 | 1,000 |
| Derived inflammatory–nutritional indices | 112 | Prognostic Nutritional Index | Preoperative | 49.61 ± 10.13 (n=16) | 52.84 ± 8.84 (n=8) | t(22)=-0.77; Δ_mean=-3.23; 95% IC [-11.98 ; 5.51] | 0,452 | 0,985 |
| Derived inflammatory–nutritional indices | 113 | Prognostic Nutritional Index | 3 months after discharge | 52.92 ± 8.41 (n=12) | 54.65 ± 5.57 (n=6) | t(16)=-0.45; Δ_mean=-1.73; 95% IC [-9.82 ; 6.37] | 0,658 | 0,985 |
| Derived inflammatory–nutritional indices | 114 | Prognostic Nutritional Index | 6 months after discharge | 55.06 ± 6.30 (n=11) | 51.00 ± 14.19 (n=5) | t(14)=0.81; Δ_mean=4.06; 95% IC [-6.66 ; 14.78] | 0,430 | 0,985 |
| Derived inflammatory–nutritional indices | 115 | Glasgow Prognostic Score (GPS) | Preoperative | 0: 6 (37.5%); 1: 8 (50.0%); 2: 2 (12.5%) | 0: 5 (62.5%); 1: 2 (25.0%); 2: 1 (12.5%) | W=78.00; Δ_HL=0.00; 95% IC [-0.00 ; 1.00] | 0,364 | 0,985 |
| Derived inflammatory–nutritional indices | 116 | Glasgow Prognostic Score (GPS) | 3 months after discharge | 0: 10 (83.3%); 1: 2 (16.7%) | 0: 5 (83.3%); 1: 1 (16.7%) | W=36.00; Δ_HL=-0.00; 95% IC [-0.00 ; 0.00] | 1,000 | 1,000 |
| Derived inflammatory–nutritional indices | 117 | Glasgow Prognostic Score (GPS) | 6 months after discharge | 0: 11 (100.0%) | 0: 4 (80.0%); 2: 1 (20.0%) | W=22.00; Δ_HL=0.00; 95% IC [-0.00 ; 0.00] | 0,178 | 0,985 |
| Derived inflammatory–nutritional indices | 118 | Albumin/CRP ratio | Preoperative | 0.46 (0.24–0.82) (n=16) | 1.17 (0.32–2.54) (n=8) | W=51.00; Δ_HL=-0.37; 95% IC [-2.02 ; 0.41] | 0,444 | 0,985 |
| Derived inflammatory–nutritional indices | 119 | Albumin/CRP ratio | 3 months after discharge | 5.80 (0.82–10.72) (n=12) | 6.36 (1.57–10.64) (n=6) | W=35.50; Δ_HL=-0.01; 95% IC [-8.40 ; 8.39] | 1,000 | 1,000 |
| Derived inflammatory–nutritional indices | 120 | Albumin/CRP ratio | 6 months after discharge | 10.32 (4.01–10.84) (n=11) | 9.12 (1.38–9.62) (n=5) | W=33.00; Δ_HL=1.20; 95% IC [-6.55 ; 9.32] | 0,571 | 0,985 |
| Derived inflammatory–nutritional indices | 121 | CRP/Albumin ratio | Preoperative | 2.16 (1.22–4.09) (n=16) | 0.92 (0.39–3.81) (n=8) | W=77.00; Δ_HL=0.71; 95% IC [-1.44 ; 2.97] | 0,444 | 0,985 |
| Derived inflammatory–nutritional indices | 122 | CRP/Albumin ratio | 3 months after discharge | 1.49 (1.03–914.85) (n=12) | 419.34 (1.03–910.14) (n=6) | W=30.50; Δ_HL=-0.48; 95% IC [-836.66 ; 863.95] | 0,639 | 0,985 |
| Derived inflammatory–nutritional indices | 123 | CRP/Albumin ratio | 6 months after discharge | 847.46 (0.40–922.87) (n=11) | 0.73 (0.11–6.16) (n=5) | W=41.00; Δ_HL=99.80; 95% IC [-5.36 ; 934.47] | 0,141 | 0,985 |
| Derived inflammatory–nutritional indices | 124 | CRP/Lymphocytes ratio | Preoperative | 3.59 (1.26–8.90) (n=16) | 2.61 (1.13–9.59) (n=8) | W=65.50; Δ_HL=0.31; 95% IC [-6.49 ; 5.69] | 0,951 | 1,000 |
| Derived inflammatory–nutritional indices | 125 | CRP/Lymphocytes ratio | 3 months after discharge | 0.89 (0.24–2.50) (n=12) | 0.48 (0.16–1.27) (n=6) | W=46.50; Δ_HL=0.16; 95% IC [-0.64 ; 2.20] | 0,349 | 0,985 |
| Derived inflammatory–nutritional indices | 126 | CRP/Lymphocytes ratio | 6 months after discharge | 0.38 (0.18–1.24) (n=11) | 0.19 (0.17–1.15) (n=5) | W=31.00; Δ_HL=0.04; 95% IC [-7.92 ; 1.86] | 0,734 | 0,985 |
| Derived inflammatory–nutritional indices | 127 | Ferritin/Albumin ratio | Preoperative | 92.89 (23.60–282.74) (n=16) | 89.09 (60.94–192.93) (n=8) | W=51.00; Δ_HL=-29.32; 95% IC [-88.06 ; 124.65] | 0,444 | 0,985 |
| Derived inflammatory–nutritional indices | 128 | Ferritin/Albumin ratio | 3 months after discharge | 58.99 (42.35–106.76) (n=12) | 82.86 (14.99–130.87) (n=6) | W=40.00; Δ_HL=3.96; 95% IC [-90.08 ; 94.39] | 0,743 | 0,985 |
| Derived inflammatory–nutritional indices | 129 | Ferritin/Albumin ratio | 6 months after discharge | 58.13 ± 37.56 (n=11) | 113.29 ± 142.85 (n=5) | t(14)=-1.24; Δ_mean=-55.16; 95% IC [-150.82 ; 40.50] | 0,237 | 0,985 |
| Derived inflammatory–nutritional indices | 130 | Ferritin/Lymphocytes ratio | Preoperative | 187.01 (44.23–413.33) (n=16) | 271.98 (133.25–650.26) (n=8) | W=45.00; Δ_HL=-87.73; 95% IC [-462.99 ; 229.60] | 0,257 | 0,985 |
| Derived inflammatory–nutritional indices | 131 | Ferritin/Lymphocytes ratio | 3 months after discharge | 102.85 (74.39–181.34) (n=12) | 115.16 (31.04–157.20) (n=6) | W=41.00; Δ_HL=26.36; 95% IC [-90.44 ; 196.11] | 0,673 | 0,985 |
| Derived inflammatory–nutritional indices | 132 | Ferritin/Lymphocytes ratio | 6 months after discharge | 103.87 (55.12–153.62) (n=11) | 93.00 (61.30–105.66) (n=5) | W=30.00; Δ_HL=9.81; 95% IC [-355.58 ; 116.95] | 0,821 | 0,985 |
| Derived inflammatory–nutritional indices | 133 | Albumin-Lymphocytes-CRP index | Preoperative | 1.10 (0.42–2.95) (n=16) | 1.64 (0.58–4.74) (n=8) | W=56.00; Δ_HL=-0.30; 95% IC [-2.95 ; 1.13] | 0,646 | 0,985 |
| Derived inflammatory–nutritional indices | 134 | Albumin-Lymphocytes-CRP index | 3 months after discharge | 16.25 ± 16.49 (n=12) | 16.71 ± 15.30 (n=6) | t(16)=-0.06; Δ_mean=-0.46; 95% IC [-17.56 ; 16.64] | 0,955 | 1,000 |
| Derived inflammatory–nutritional indices | 135 | Albumin-Lymphocytes-CRP index | 6 months after discharge | 19.67 ± 15.21 (n=11) | 17.51 ± 14.99 (n=5) | t(14)=0.26; Δ_mean=2.16; 95% IC [-15.37 ; 19.68] | 0,796 | 0,985 |
| Notes: Values are presented as mean +/- standard deviation or median (P25-P75), with the valid n shown for each group and comparison. The analysis used observed available data only; no multiple imputation was performed. The number of patients evaluated was preoperative n = 24, 3 months after discharge n = 18, and 6 months after discharge n = 16. Raw p-values correspond to the statistical test reported in each row. BH-FDR adjusted p-values were calculated with the Benjamini-Hochberg false discovery rate procedure within each Exposure x Domain family: Dietary intake (45 tests), Clinical-nutritional laboratory analyses (66 tests), and Derived inflammatory-nutritional indices (24 tests). The BH-FDR audit found 945 inferential comparisons, 43 raw p-values < 0.05, and 0 BH-FDR adjusted p-values < 0.05. For binary exposures, two-sample t-test/Welch t-test or Mann-Whitney/Wilcoxon rank-sum tests were used as reported. For multicategorical or ordinal exposures, one-way ANOVA or Kruskal-Wallis tests were used; these are omnibus tests and do not establish pairwise differences or dose-response trends without post-hoc or contrast testing. Because this was an exploratory supplementary analysis with multiple comparisons, small subgroup sizes, and no covariate adjustment, the results should be interpreted as hypothesis-generating rather than confirmatory. Abbreviations: BH-FDR, Benjamini-Hochberg false discovery rate; ERCP, endoscopic retrograde cholangiopancreatography; EPI, exocrine pancreatic insufficiency; ISGPS, International Study Group of Pancreatic Surgery; PERT, pancreatic enzyme replacement therapy; POPF, postoperative pancreatic fistula; TIBC, total iron-binding capacity; UIBC, unsaturated iron-binding capacity. | | | | | | | | |

| **Supplementary Table S6.** Exploratory associations between Delayed gastric emptying classification according to ISGPS and dietary intake, clinical-nutritional laboratory variables, and inflammatory-nutritional indices after pancreatoduodenectomy. | | | | | | | | | |
| --- | --- | --- | --- | --- | --- | --- | --- | --- | --- |
| **Outcome** | | | | **Delayed gastric emptying classification according to ISGPS** | | | | | |
| **Domain** | **Source row** | **Variable** | **Time** | **Grade A** | **Grade B** | **Grade C** | **Statistical result** | **Raw p-value** | **BH-FDR adjusted p-value** |
|  |  |  |  | **Mean ± SD or Median (P25–P75), n** | **Mean ± SD or Median (P25–P75), n** | **Mean ± SD or Median (P25–P75), n** |  |  |  |
| Dietary intake | 1 | Energy (kcal) | Preoperative | 2730.04 ± 566.39 (n=14) | 2588.52 ± 790.03 (n=5) | 2906.17 ± 377.61 (n=3) | F(2, 19)=0.26 | 0,772 | 0,921 |
| Dietary intake | 2 | Energy (kcal) | 3 months after discharge | 2035.63 (1955.01–2323.24) (n=13) | 1644.29 (1623.75–1991.86) (n=3) | 1841.09 (1800.58–1881.61) (n=2) | H(2)=3.090.951 | 0,213 | 0,903 |
| Dietary intake | 3 | Energy (kcal) | 6 months after discharge | 2014.00 (1850.60–2178.34) (n=13) | 1917.77 (1917.77–1917.77) (n=1) | 1821.32 (1760.82–1881.83) (n=2) | H(2)=1.08 | 0,582 | 0,903 |
| Dietary intake | 4 | Protein (g) | Preoperative | 122.71 ± 22.11 (n=14) | 108.79 ± 31.54 (n=5) | 113.25 ± 15.52 (n=3) | F(2, 19)=0.70 | 0,507 | 0,903 |
| Dietary intake | 5 | Protein (g) | 3 months after discharge | 100.20 (90.17–107.46) (n=13) | 88.52 (86.48–92.20) (n=3) | 86.79 (86.75–86.83) (n=2) | H(2)=3.520.920 | 0,172 | 0,903 |
| Dietary intake | 6 | Protein (g) | 6 months after discharge | 99.70 (96.22–110.52) (n=13) | 96.33 (96.33–96.33) (n=1) | 86.71 (85.25–88.16) (n=2) | H(2)=3.890.893 | 0,143 | 0,903 |
| Dietary intake | 7 | Relative energy balance (kcal) | Preoperative | 1162.18 (1013.06–1331.19) (n=14) | 1229.60 (790.05–1255.05) (n=5) | 1244.65 (1086.59–1248.81) (n=3) | H(2)=0.07 | 0,968 | 0,982 |
| Dietary intake | 8 | Relative energy balance (kcal) | 3 months after discharge | 514.92 (440.56–693.70) (n=13) | 380.58 (278.69–482.85) (n=3) | 547.59 (458.75–636.42) (n=2) | H(2)=1.30 | 0,522 | 0,903 |
| Dietary intake | 9 | Relative energy balance (kcal) | 6 months after discharge | 634.02 (597.67–693.01) (n=13) | 501.44 (501.44–501.44) (n=1) | 506.39 (494.77–518.01) (n=2) | H(2)=3.300.920 | 0,192 | 0,903 |
| Dietary intake | 10 | Carbohydrate (g) | Preoperative | 373.20 ± 80.17 (n=14) | 366.68 ± 104.95 (n=5) | 403.09 ± 28.90 (n=3) | F(2, 19)=0.20 | 0,819 | 0,921 |
| Dietary intake | 11 | Carbohydrate (g) | 3 months after discharge | 271.36 (249.02–281.31) (n=13) | 227.73 (215.63–271.73) (n=3) | 252.00 (244.89–259.10) (n=2) | H(2)=1.30 | 0,523 | 0,903 |
| Dietary intake | 12 | Carbohydrate (g) | 6 months after discharge | 255.28 (234.52–279.88) (n=13) | 268.49 (268.49–268.49) (n=1) | 252.07 (242.14–262.00) (n=2) | H(2)=0.41 | 0,816 | 0,921 |
| Dietary intake | 13 | Lipid (g) | Preoperative | 82.94 ± 24.76 (n=14) | 76.30 ± 28.64 (n=5) | 93.42 ± 23.86 (n=3) | F(2, 19)=0.42 | 0,662 | 0,910 |
| Dietary intake | 14 | Lipid (g) | 3 months after discharge | 65.56 (62.92–81.03) (n=13) | 48.34 (46.15–62.67) (n=3) | 54.00 (52.63–55.36) (n=2) | H(2)=3.740.893 | 0,154 | 0,903 |
| Dietary intake | 15 | Lipid (g) | 6 months after discharge | 59.66 (56.68–78.45) (n=13) | 50.94 (50.94–50.94) (n=1) | 51.80 (50.14–53.47) (n=2) | H(2)=2.000.989 | 0,369 | 0,903 |
| Dietary intake | 16 | Fiber (g) | Preoperative | 43.01 ± 11.62 (n=14) | 50.72 ± 9.01 (n=5) | 49.77 ± 8.49 (n=3) | F(2, 19)=1.180.989 | 0,328 | 0,903 |
| Dietary intake | 17 | Fiber (g) | 3 months after discharge | 36.38 (31.76–46.11) (n=13) | 37.20 (34.90–46.76) (n=3) | 36.99 (35.87–38.12) (n=2) | H(2)=0.43 | 0,808 | 0,921 |
| Dietary intake | 18 | Fiber (g) | 6 months after discharge | 36.46 (28.17–42.79) (n=13) | 43.46 (43.46–43.46) (n=1) | 36.61 (35.90–37.31) (n=2) | H(2)=0.96 | 0,618 | 0,910 |
| Dietary intake | 19 | Calcium (mg) | Preoperative | 1471.34 ± 329.77 (n=14) | 1556.13 ± 333.23 (n=5) | 1348.38 ± 385.68 (n=3) | F(2, 19)=0.36 | 0,704 | 0,921 |
| Dietary intake | 20 | Calcium (mg) | 3 months after discharge | 1274.51 (1019.91–1437.97) (n=13) | 1261.54 (1035.72–1543.12) (n=3) | 1263.50 (1157.34–1369.66) (n=2) | H(2)=0.04 | 0,978 | 0,982 |
| Dietary intake | 21 | Calcium (mg) | 6 months after discharge | 1258.11 (1015.80–1491.53) (n=13) | 944.45 (944.45–944.45) (n=1) | 1243.33 (1152.77–1333.89) (n=2) | H(2)=1.46 | 0,481 | 0,903 |
| Dietary intake | 22 | Iron (mg) | Preoperative | 26.26 ± 5.69 (n=14) | 28.71 ± 9.02 (n=5) | 30.28 ± 8.09 (n=3) | F(2, 19)=0.56 | 0,581 | 0,903 |
| Dietary intake | 23 | Iron (mg) | 3 months after discharge | 24.65 (19.73–27.88) (n=13) | 17.77 (17.76–26.72) (n=3) | 17.60 (16.44–18.76) (n=2) | H(2)=1.67 | 0,434 | 0,903 |
| Dietary intake | 24 | Iron (mg) | 6 months after discharge | 23.82 (18.72–26.01) (n=13) | 20.71 (20.71–20.71) (n=1) | 17.34 (16.38–18.29) (n=2) | H(2)=1.67 | 0,434 | 0,903 |
| Dietary intake | 25 | Potassium (mg) | Preoperative | 6703.00 ± 1439.69 (n=14) | 6681.83 ± 1454.82 (n=5) | 6861.38 ± 647.78 (n=3) | F(2, 19)=0.02 | 0,982 | 0,982 |
| Dietary intake | 26 | Potassium (mg) | 3 months after discharge | 5444.64 (4879.30–6898.60) (n=13) | 5463.24 (5013.89–6640.84) (n=3) | 4835.20 (4685.92–4984.49) (n=2) | H(2)=1.32 | 0,517 | 0,903 |
| Dietary intake | 27 | Potassium (mg) | 6 months after discharge | 5350.54 (4481.17–6624.25) (n=13) | 6372.40 (6372.40–6372.40) (n=1) | 4770.86 (4677.58–4864.15) (n=2) | H(2)=1.10 | 0,577 | 0,903 |
| Dietary intake | 28 | Selenium (µg) | Preoperative | 66.69 ± 19.10 (n=14) | 43.29 ± 10.06 (n=5) | 40.61 ± 14.25 (n=3) | F(2, 19)=5.21 | 0,016 | 0,720 |
| Dietary intake | 29 | Selenium (µg) | 3 months after discharge | 52.03 (39.32–67.98) (n=13) | 34.76 (24.02–40.81) (n=3) | 28.44 (22.91–33.97) (n=2) | H(2)=4.50 | 0,106 | 0,903 |
| Dietary intake | 30 | Selenium (µg) | 6 months after discharge | 52.03 (38.83–63.32) (n=13) | 15.48 (15.48–15.48) (n=1) | 27.86 (22.71–33.00) (n=2) | H(2)=5.73 | 0,057 | 0,903 |
| Dietary intake | 31 | Zinc (mg) | Preoperative | 18.18 ± 4.96 (n=14) | 18.64 ± 8.74 (n=5) | 19.39 ± 5.00 (n=3) | F(2, 19)=0.05 | 0,947 | 0,982 |
| Dietary intake | 32 | Zinc (mg) | 3 months after discharge | 14.76 (13.07–16.79) (n=13) | 11.11 (9.96–18.77) (n=3) | 11.54 (11.26–11.83) (n=2) | H(2)=2.150.989 | 0,341 | 0,903 |
| Dietary intake | 33 | Zinc (mg) | 6 months after discharge | 13.93 (12.51–16.03) (n=13) | 10.26 (10.26–10.26) (n=1) | 11.39 (11.24–11.55) (n=2) | H(2)=3.360.920 | 0,186 | 0,903 |
| Dietary intake | 34 | Sodium (mg) | Preoperative | 1653.31 (1293.47–2522.85) (n=14) | 1443.00 (1068.23–1675.20) (n=5) | 2002.26 (1820.04–2355.93) (n=3) | H(2)=2.22 | 0,329 | 0,903 |
| Dietary intake | 35 | Sodium (mg) | 3 months after discharge | 1203.71 (1109.13–1668.49) (n=13) | 1287.54 (1031.90–1510.22) (n=3) | 1485.97 (1438.06–1533.88) (n=2) | H(2)=0.32 | 0,854 | 0,937 |
| Dietary intake | 36 | Sodium (mg) | 6 months after discharge | 1150.44 (1008.44–1601.63) (n=13) | 905.24 (905.24–905.24) (n=1) | 1470.76 (1406.94–1534.59) (n=2) | H(2)=1.89 | 0,389 | 0,903 |
| Dietary intake | 37 | Vitamin A (mg) | Preoperative | 4.36 (3.58–9.12) (n=14) | 5.69 (5.44–6.06) (n=5) | 1.76 (1.75–3.96) (n=3) | H(2)=2.66 | 0,265 | 0,903 |
| Dietary intake | 38 | Vitamin A (mg) | 3 months after discharge | 4.78 (2.89–7.34) (n=13) | 3.92 (3.91–7.99) (n=3) | 3.30 (2.92–3.69) (n=2) | H(2)=1.65 | 0,439 | 0,903 |
| Dietary intake | 39 | Vitamin A (mg) | 6 months after discharge | 4.75 (2.86–6.66) (n=13) | 4.54 (4.54–4.54) (n=1) | 3.25 (2.90–3.59) (n=2) | H(2)=1.67 | 0,434 | 0,903 |
| Dietary intake | 40 | Vitamin C (mg) | Preoperative | 401.72 ± 216.67 (n=14) | 377.24 ± 156.55 (n=5) | 311.45 ± 206.91 (n=3) | F(2, 19)=0.24 | 0,786 | 0,921 |
| Dietary intake | 41 | Vitamin C (mg) | 3 months after discharge | 312.79 (200.57–504.52) (n=13) | 349.88 (280.05–530.65) (n=3) | 280.33 (246.33–314.32) (n=2) | H(2)=0.81 | 0,667 | 0,910 |
| Dietary intake | 42 | Vitamin C (mg) | 6 months after discharge | 304.74 (200.58–457.98) (n=13) | 406.84 (406.84–406.84) (n=1) | 275.37 (244.98–305.76) (n=2) | H(2)=0.58 | 0,747 | 0,921 |
| Dietary intake | 43 | Vitamin E (mg) | Preoperative | 4.91 ± 1.77 (n=14) | 4.57 ± 1.14 (n=5) | 3.13 ± 1.71 (n=3) | F(2, 19)=1.43 | 0,263 | 0,903 |
| Dietary intake | 44 | Vitamin E (mg) | 3 months after discharge | 4.62 (2.66–6.13) (n=13) | 3.51 (3.31–5.59) (n=3) | 2.75 (2.41–3.10) (n=2) | H(2)=2.01 | 0,366 | 0,903 |
| Dietary intake | 45 | Vitamin E (mg) | 6 months after discharge | 3.88 (2.42–5.82) (n=13) | 3.65 (3.65–3.65) (n=1) | 2.71 (2.41–3.01) (n=2) | H(2)=0.91 | 0,635 | 0,910 |
| Clinical–Nutritional Laboratory Analyses | 46 | Total Proteins (g/dL) | Preoperative | 6.94 ± 0.55 (n=14) | 6.83 ± 0.73 (n=5) | 6.69 ± 0.34 (n=3) | F(2, 19)=0.26 | 0,777 | 0,827 |
| Clinical–Nutritional Laboratory Analyses | 47 | Total Proteins (g/dL) | 3 months after discharge after discharge | 6.87 (6.61–7.15) (n=13) | 6.33 (6.26–6.46) (n=3) | 7.28 (7.08–7.47) (n=2) | H(2)=6.17 | 0,046 | 0,484 |
| Clinical–Nutritional Laboratory Analyses | 48 | Total Proteins (g/dL) | 6 months after discharge after discharge | 6.69 (6.42–7.03) (n=13) | 6.49 (6.49–6.49) (n=1) | 6.08 (5.98–6.19) (n=2) | H(2)=3.89 | 0,143 | 0,484 |
| Clinical–Nutritional Laboratory Analyses | 49 | Albumin (g/dL) | Preoperative | 4.01 ± 0.67 (n=14) | 3.74 ± 0.65 (n=5) | 4.28 ± 0.24 (n=3) | F(2, 19)=0.72 | 0,502 | 0,750 |
| Clinical–Nutritional Laboratory Analyses | 50 | Albumin (g/dL) | 3 months after discharge after discharge | 4.28 (4.07–4.59) (n=13) | 3.98 (3.87–4.13) (n=3) | 3.46 (3.20–3.72) (n=2) | H(2)=4.49 | 0,106 | 0,484 |
| Clinical–Nutritional Laboratory Analyses | 51 | Albumin (g/dL) | 6 months after discharge after discharge | 4.28 (3.97–4.72) (n=13) | 4.22 (4.22–4.22) (n=1) | 3.28 (2.71–3.85) (n=2) | H(2)=0.67 | 0,717 | 0,811 |
| Clinical–Nutritional Laboratory Analyses | 52 | C-Reactive Protein (mg/L) | Preoperative | 8.57 ± 7.72 (n=14) | 4.53 ± 4.45 (n=5) | 21.15 ± 17.09 (n=3) | F(2, 19)=3.58 | 0,048 | 0,484 |
| Clinical–Nutritional Laboratory Analyses | 53 | C-Reactive Protein (mg/L) | 3 months after discharge after discharge | 0.40 (0.40–1.96) (n=13) | 4.64 (2.52–4.72) (n=3) | 2.92 (2.34–3.51) (n=2) | H(2)=1.82 | 0,403 | 0,719 |
| Clinical–Nutritional Laboratory Analyses | 54 | C-Reactive Protein (mg/L) | 6 months after discharge after discharge | 0.40 (0.40–0.58) (n=13) | 1.64 (1.64–1.64) (n=1) | 7.51 (4.68–10.35) (n=2) | H(2)=5.50 | 0,064 | 0,484 |
| Clinical–Nutritional Laboratory Analyses | 55 | Blood Glucose (mg/dL) | Preoperative | 121.54 (101.00–148.85) (n=14) | 168.00 (107.00–178.00) (n=5) | 134.00 (110.25–151.00) (n=3) | H(2)=1.41 | 0,495 | 0,750 |
| Clinical–Nutritional Laboratory Analyses | 56 | Blood Glucose (mg/dL) | 3 months after discharge after discharge | 121.61 (84.82–134.93) (n=13) | 102.00 (87.88–115.11) (n=3) | 116.70 (96.56–136.85) (n=2) | H(2)=0.87 | 0,647 | 0,811 |
| Clinical–Nutritional Laboratory Analyses | 57 | Blood Glucose (mg/dL) | 6 months after discharge after discharge | 114.97 (80.55–120.90) (n=13) | 90.53 (90.53–90.53) (n=1) | 91.75 (79.62–103.87) (n=2) | H(2)=1.32 | 0,518 | 0,750 |
| Clinical–Nutritional Laboratory Analyses | 58 | Hemoglobin (g/dL) | Preoperative | 12.94 ± 2.42 (n=14) | 12.36 ± 2.06 (n=5) | 12.80 ± 2.19 (n=3) | F(2, 19)=0.11 | 0,894 | 0,908 |
| Clinical–Nutritional Laboratory Analyses | 59 | Hemoglobin (g/dL) | 3 months after discharge after discharge | 13.10 (12.00–14.10) (n=13) | 10.80 (10.55–11.80) (n=3) | 12.75 (12.58–12.92) (n=2) | H(2)=3.08 | 0,215 | 0,568 |
| Clinical–Nutritional Laboratory Analyses | 60 | Hemoglobin (g/dL) | 6 months after discharge after discharge | 13.10 (12.10–14.40) (n=13) | 10.50 (10.50–10.50) (n=1) | 9.20 (8.55–9.85) (n=2) | H(2)=5.61 | 0,061 | 0,484 |
| Clinical–Nutritional Laboratory Analyses | 61 | Lymphocytes (x10³/mm³) | Preoperative | 2.44 ± 1.34 (n=14) | 1.88 ± 0.97 (n=5) | 2.13 ± 1.63 (n=3) | F(2, 19)=0.35 | 0,708 | 0,811 |
| Clinical–Nutritional Laboratory Analyses | 62 | Lymphocytes (x10³/mm³) | 3 months after discharge after discharge | 2.40 (1.90–3.20) (n=13) | 2.80 (2.40–3.15) (n=3) | 2.10 (2.00–2.20) (n=2) | H(2)=1.12 | 0,572 | 0,781 |
| Clinical–Nutritional Laboratory Analyses | 63 | Lymphocytes (x10³/mm³) | 6 months after discharge after discharge | 2.30 (1.90–3.20) (n=13) | 3.20 (3.20–3.20) (n=1) | 0.90 (0.75–1.05) (n=2) | H(2)=5.38 | 0,068 | 0,484 |
| Clinical–Nutritional Laboratory Analyses | 64 | Serum Iron (µg/dL) | Preoperative | 84.39 ± 32.22 (n=14) | 74.87 ± 54.56 (n=5) | 120.01 ± 43.64 (n=3) | F(2, 19)=1.33 | 0,287 | 0,642 |
| Clinical–Nutritional Laboratory Analyses | 65 | Serum Iron (µg/dL) | 3 months after discharge after discharge | 101.74 (94.54–151.77) (n=13) | 38.68 (37.14–44.16) (n=3) | 56.55 (47.00–66.11) (n=2) | H(2)=6.46 | 0,040 | 0,484 |
| Clinical–Nutritional Laboratory Analyses | 66 | Serum Iron (µg/dL) | 6 months after discharge after discharge | 94.28 (81.26–102.93) (n=13) | 59.69 (59.69–59.69) (n=1) | 49.61 (36.61–62.60) (n=2) | H(2)=3.81 | 0,149 | 0,484 |
| Clinical–Nutritional Laboratory Analyses | 67 | Ferritin (ng/mL) | Preoperative | 466.15 (219.35–838.80) (n=14) | 250.10 (80.30–529.40) (n=5) | 770.30 (489.70–1059.40) (n=3) | H(2)=1.29 | 0,523 | 0,750 |
| Clinical–Nutritional Laboratory Analyses | 68 | Ferritin (ng/mL) | 3 months after discharge after discharge | 254.60 (183.60–447.90) (n=13) | 221.40 (120.55–739.75) (n=3) | 498.90 (308.35–689.45) (n=2) | H(2)=0.10 | 0,953 | 0,953 |
| Clinical–Nutritional Laboratory Analyses | 69 | Ferritin (ng/mL) | 6 months after discharge after discharge | 195.30 (128.00–401.50) (n=13) | 78.70 (78.70–78.70) (n=1) | 493.65 (355.32–631.97) (n=2) | H(2)=2.77 | 0,251 | 0,614 |
| Clinical–Nutritional Laboratory Analyses | 70 | UIBC (µg/dL) | Preoperative | 302.03 (269.45–323.41) (n=14) | 259.21 (258.70–304.89) (n=5) | 216.04 (216.04–241.09) (n=3) | H(2)=3.74 | 0,154 | 0,484 |
| Clinical–Nutritional Laboratory Analyses | 71 | UIBC (µg/dL) | 3 months after discharge after discharge | 301.64 (288.72–308.35) (n=13) | 269.14 (251.91–332.49) (n=3) | 273.13 (258.11–288.15) (n=2) | H(2)=1.09 | 0,580 | 0,781 |
| Clinical–Nutritional Laboratory Analyses | 72 | UIBC (µg/dL) | 6 months after discharge after discharge | 297.48 (286.07–321.65) (n=13) | 309.55 (309.55–309.55) (n=1) | 258.77 (250.93–266.61) (n=2) | H(2)=3.76 | 0,153 | 0,484 |
| Clinical–Nutritional Laboratory Analyses | 73 | TIBC (µg/dL) | Preoperative | 377.18 ± 85.62 (n=14) | 362.98 ± 85.04 (n=5) | 352.76 ± 71.47 (n=3) | F(2, 19)=0.13 | 0,878 | 0,905 |
| Clinical–Nutritional Laboratory Analyses | 74 | TIBC (µg/dL) | 3 months after discharge after discharge | 415.72 (403.45–431.09) (n=13) | 307.82 (289.06–376.64) (n=3) | 329.69 (324.22–335.15) (n=2) | H(2)=3.09 | 0,213 | 0,568 |
| Clinical–Nutritional Laboratory Analyses | 75 | TIBC (µg/dL) | 6 months after discharge after discharge | 412.19 (381.53–419.64) (n=13) | 369.24 (369.24–369.24) (n=1) | 308.38 (303.23–313.53) (n=2) | H(2)=4.17 | 0,125 | 0,484 |
| Clinical–Nutritional Laboratory Analyses | 76 | Transferrin (mg/dL) | Preoperative | 241.62 (215.56–258.73) (n=14) | 207.37 (206.96–243.91) (n=5) | 172.83 (172.83–192.88) (n=3) | H(2)=3.74 | 0,154 | 0,484 |
| Clinical–Nutritional Laboratory Analyses | 77 | Transferrin (mg/dL) | 3 months after discharge after discharge | 241.31 (230.98–246.68) (n=13) | 215.31 (201.53–265.99) (n=3) | 218.50 (206.49–230.52) (n=2) | H(2)=1.09 | 0,580 | 0,781 |
| Clinical–Nutritional Laboratory Analyses | 78 | Transferrin (mg/dL) | 6 months after discharge after discharge | 237.98 (228.86–257.32) (n=13) | 247.64 (247.64–247.64) (n=1) | 207.02 (200.74–213.29) (n=2) | H(2)=3.76 | 0,153 | 0,484 |
| Clinical–Nutritional Laboratory Analyses | 79 | Transferrin Saturation (%) | Preoperative | 22.17 ± 6.82 (n=14) | 19.69 ± 10.27 (n=5) | 33.32 ± 5.68 (n=3) | F(2, 19)=3.34 | 0,057 | 0,484 |
| Clinical–Nutritional Laboratory Analyses | 80 | Transferrin Saturation (%) | 3 months after discharge after discharge | 24.10 (22.72–31.97) (n=13) | 12.57 (11.85–12.87) (n=3) | 17.37 (14.18–20.55) (n=2) | H(2)=7.61 | 0,022 | 0,484 |
| Clinical–Nutritional Laboratory Analyses | 81 | Transferrin Saturation (%) | 6 months after discharge after discharge | 21.97 (18.78–25.50) (n=13) | 16.17 (16.17–16.17) (n=1) | 15.82 (11.87–19.77) (n=2) | H(2)=1.75 | 0,417 | 0,724 |
| Clinical–Nutritional Laboratory Analyses | 82 | Urea (mg/dL) | Preoperative | 33.58 (27.55–36.35) (n=14) | 21.59 (14.87–34.56) (n=5) | 40.51 (33.23–42.28) (n=3) | H(2)=2.27 | 0,321 | 0,642 |
| Clinical–Nutritional Laboratory Analyses | 83 | Urea (mg/dL) | 3 months after discharge after discharge | 35.01 (31.03–51.77) (n=13) | 26.43 (24.58–32.70) (n=3) | 46.69 (35.59–57.78) (n=2) | H(2)=1.03 | 0,598 | 0,789 |
| Clinical–Nutritional Laboratory Analyses | 84 | Urea (mg/dL) | 6 months after discharge after discharge | 25.49 (22.30–33.25) (n=13) | 39.31 (39.31–39.31) (n=1) | 83.85 (59.26–108.45) (n=2) | H(2)=3.36 | 0,186 | 0,534 |
| Clinical–Nutritional Laboratory Analyses | 85 | Creatinine (mg/dL) | Preoperative | 0.94 (0.82–1.14) (n=14) | 0.74 (0.71–0.85) (n=5) | 0.94 (0.89–1.15) (n=3) | H(2)=2.38 | 0,304 | 0,642 |
| Clinical–Nutritional Laboratory Analyses | 86 | Creatinine (mg/dL) | 3 months after discharge after discharge | 0.82 (0.75–0.94) (n=13) | 0.73 (0.73–0.84) (n=3) | 1.08 (1.00–1.16) (n=2) | H(2)=2.43 | 0,296 | 0,642 |
| Clinical–Nutritional Laboratory Analyses | 87 | Creatinine (mg/dL) | 6 months after discharge after discharge | 0.87 (0.73–0.97) (n=13) | 1.16 (1.16–1.16) (n=1) | 1.79 (1.31–2.28) (n=2) | H(2)=2.28 | 0,320 | 0,642 |
| Clinical–Nutritional Laboratory Analyses | 88 | Aspartate Aminotransferase (AST) (U/L) | Preoperative | 132.49 ± 97.26 (n=14) | 178.86 ± 99.81 (n=5) | 305.91 ± 289.71 (n=3) | F(2, 19)=2.16 | 0,143 | 0,484 |
| Clinical–Nutritional Laboratory Analyses | 89 | Aspartate Aminotransferase (AST) (U/L) | 3 months after discharge after discharge | 27.47 (24.29–35.43) (n=13) | 36.91 (27.19–74.42) (n=3) | 43.45 (32.88–54.02) (n=2) | H(2)=0.58 | 0,750 | 0,811 |
| Clinical–Nutritional Laboratory Analyses | 90 | Aspartate Aminotransferase (AST) (U/L) | 6 months after discharge after discharge | 29.22 (27.32–37.05) (n=13) | 39.88 (39.88–39.88) (n=1) | 32.70 (25.21–40.20) (n=2) | H(2)=1.43 | 0,490 | 0,750 |
| Clinical–Nutritional Laboratory Analyses | 91 | Aminotransferase (ALT) (U/L) | Preoperative | 145.00 (54.47–234.49) (n=14) | 148.88 (122.00–204.28) (n=5) | 144.48 (84.31–467.45) (n=3) | H(2)=0.38 | 0,828 | 0,867 |
| Clinical–Nutritional Laboratory Analyses | 92 | Aminotransferase (ALT) (U/L) | 3 months after discharge after discharge | 33.04 (28.37–38.72) (n=13) | 48.57 (32.55–58.57) (n=3) | 39.23 (33.95–44.52) (n=2) | H(2)=1.30 | 0,523 | 0,750 |
| Clinical–Nutritional Laboratory Analyses | 93 | Aminotransferase (ALT) (U/L) | 6 months after discharge after discharge | 34.21 (26.44–39.35) (n=13) | 42.54 (42.54–42.54) (n=1) | 50.02 (31.61–68.43) (n=2) | H(2)=0.58 | 0,748 | 0,811 |
| Clinical–Nutritional Laboratory Analyses | 94 | Folic Acid (ng/mL) | Preoperative | 15.67 ± 5.44 (n=14) | 15.71 ± 9.16 (n=5) | 10.05 ± 0.44 (n=3) | F(2, 19)=1.08 | 0,359 | 0,697 |
| Clinical–Nutritional Laboratory Analyses | 95 | Folic Acid (ng/mL) | 3 months after discharge after discharge | 14.74 (10.62–24.00) (n=13) | 24.00 (24.00–24.00) (n=3) | 12.34 (10.97–13.71) (n=2) | H(2)=4.37 | 0,113 | 0,484 |
| Clinical–Nutritional Laboratory Analyses | 96 | Folic Acid (ng/mL) | 6 months after discharge after discharge | 21.47 (14.52–23.66) (n=13) | 24.00 (24.00–24.00) (n=1) | 16.80 (13.20–20.40) (n=2) | H(2)=1.47 | 0,480 | 0,750 |
| Clinical–Nutritional Laboratory Analyses | 97 | Vitamin B12 (pg/mL) | Preoperative | 478.00 (425.00–784.00) (n=14) | 455.00 (407.00–625.00) (n=5) | 589.00 (499.50–1294.50) (n=3) | H(2)=0.73 | 0,693 | 0,811 |
| Clinical–Nutritional Laboratory Analyses | 98 | Vitamin B12 (pg/mL) | 3 months after discharge after discharge | 487.00 (372.00–597.00) (n=13) | 1092.00 (702.50–1546.00) (n=3) | 485.50 (435.75–535.25) (n=2) | H(2)=0.79 | 0,674 | 0,811 |
| Clinical–Nutritional Laboratory Analyses | 99 | Vitamin B12 (pg/mL) | 6 months after discharge after discharge | 379.00 (313.00–531.00) (n=13) | 515.00 (515.00–515.00) (n=1) | 462.50 (401.25–523.75) (n=2) | H(2)=0.58 | 0,749 | 0,811 |
| Clinical–Nutritional Laboratory Analyses | 100 | Vitamin A (µg/dL) | Preoperative | 0.28 (0.19–0.37) (n=14) | 0.31 (0.30–0.34) (n=5) | 0.31 (0.24–0.31) (n=3) | H(2)=0.63 | 0,729 | 0,811 |
| Clinical–Nutritional Laboratory Analyses | 101 | Vitamin A (µg/dL) | 3 months after discharge after discharge | 0.33 (0.29–0.44) (n=13) | 0.21 (0.21–0.21) (n=3) | 0.32 (0.31–0.34) (n=2) | H(2)=4.15 | 0,125 | 0,484 |
| Clinical–Nutritional Laboratory Analyses | 102 | Vitamin A (µg/dL) | 6 months after discharge after discharge | 0.32 (0.26–0.36) (n=13) | 0.61 (0.61–0.61) (n=1) | 0.29 (0.28–0.29) (n=2) | H(2)=2.77 | 0,250 | 0,614 |
| Clinical–Nutritional Laboratory Analyses | 103 | Vitamin E (mg/dL) | Preoperative | 8.30 (6.49–9.63) (n=14) | 7.28 (7.28–7.46) (n=5) | 7.28 (6.62–11.15) (n=3) | H(2)=1.56 | 0,459 | 0,750 |
| Clinical–Nutritional Laboratory Analyses | 104 | Vitamin E (mg/dL) | 3 months after discharge after discharge | 6.65 (5.72–8.29) (n=13) | 4.57 (4.40–6.22) (n=3) | 6.30 (6.15–6.45) (n=2) | H(2)=0.58 | 0,750 | 0,811 |
| Clinical–Nutritional Laboratory Analyses | 105 | Vitamin E (mg/dL) | 6 months after discharge after discharge | 5.09 (4.68–6.52) (n=13) | 10.40 (10.40–10.40) (n=1) | 5.82 (5.73–5.91) (n=2) | H(2)=2.37 | 0,305 | 0,642 |
| Clinical–Nutritional Laboratory Analyses | 106 | Copper (µg/dL) | Preoperative | 142.21 ± 52.72 (n=14) | 159.80 ± 70.83 (n=5) | 128.33 ± 21.36 (n=3) | F(2, 19)=0.34 | 0,718 | 0,811 |
| Clinical–Nutritional Laboratory Analyses | 107 | Copper (µg/dL) | 3 months after discharge after discharge | 99.40 (91.00–112.20) (n=13) | 118.80 (93.50–136.50) (n=3) | 145.20 (120.60–169.80) (n=2) | H(2)=1.85 | 0,397 | 0,719 |
| Clinical–Nutritional Laboratory Analyses | 108 | Copper (µg/dL) | 6 months after discharge after discharge | 92.60 (86.80–103.40) (n=13) | 140.60 (140.60–140.60) (n=1) | 133.10 (114.55–151.65) (n=2) | H(2)=3.83 | 0,147 | 0,484 |
| Clinical–Nutritional Laboratory Analyses | 109 | Zinc (µg/dL) | Preoperative | 82.25 (60.75–96.75) (n=14) | 93.00 (93.00–96.00) (n=5) | 54.00 (54.00–72.00) (n=3) | H(2)=3.97 | 0,137 | 0,484 |
| Clinical–Nutritional Laboratory Analyses | 110 | Zinc (µg/dL) | 3 months after discharge after discharge | 79.00 (76.50–92.00) (n=13) | 85.00 (79.75–86.75) (n=3) | 72.25 (70.12–74.38) (n=2) | H(2)=1.99 | 0,370 | 0,698 |
| Clinical–Nutritional Laboratory Analyses | 111 | Zinc (µg/dL) | 6 months after discharge after discharge | 80.50 (74.26–89.50) (n=13) | 87.50 (87.50–87.50) (n=1) | 63.50 (61.25–65.75) (n=2) | H(2)=3.40 | 0,183 | 0,534 |
| Derived inflammatory–nutritional indices | 112 | Prognostic Nutritional Index | Preoperative | 52.31 ± 11.12 (n=14) | 46.80 ± 5.63 (n=5) | 53.47 ± 9.95 (n=3) | F(2, 19)=0.64 | 0,540 | 0,762 |
| Derived inflammatory–nutritional indices | 113 | Prognostic Nutritional Index | 3 months after discharge | 57.80 (49.20–59.30) (n=13) | 55.00 (52.40–55.90) (n=3) | 45.10 (42.00–48.20) (n=2) | H(2)=2.25 | 0,325 | 0,683 |
| Derived inflammatory–nutritional indices | 114 | Prognostic Nutritional Index | 6 months after discharge | 58.06 (49.62–60.80) (n=13) | 58.20 (58.20–58.20) (n=1) | 37.30 (32.35–42.25) (n=2) | H(2)=4.28 | 0,118 | 0,429 |
| Derived inflammatory–nutritional indices | 115 | Glasgow Prognostic Score (GPS) | Preoperative | 0: 8 (57.1%); 1: 4 (28.6%); 2: 2 (14.3%) | 0: 2 (40.0%); 1: 3 (60.0%) | 0: 1 (33.3%); 1: 2 (66.7%) | H(2)=0.23 | 0,890 | 0,908 |
| Derived inflammatory–nutritional indices | 116 | Glasgow Prognostic Score (GPS) | 3 months after discharge | 0: 11 (84.6%); 1: 2 (15.4%) | 0: 3 (100.0%) | 0: 1 (50.0%); 1: 1 (50.0%) | H(2)=2.09 | 0,351 | 0,683 |
| Derived inflammatory–nutritional indices | 117 | Glasgow Prognostic Score (GPS) | 6 months after discharge | 0: 13 (100.0%) | 0: 1 (100.0%) | 0: 1 (50.0%); 2: 1 (50.0%) | H(2)=7.00 | 0,030 | 0,416 |
| Derived inflammatory–nutritional indices | 118 | Albumin/CRP ratio | Preoperative | 0.64 (0.29–1.34) (n=14) | 0.80 (0.80–2.10) (n=5) | 0.15 (0.14–1.31) (n=3) | H(2)=1.57 | 0,456 | 0,762 |
| Derived inflammatory–nutritional indices | 119 | Albumin/CRP ratio | 3 months after discharge | 10.45 (2.05–10.80) (n=13) | 0.83 (0.82–5.76) (n=3) | 1.49 (1.10–1.88) (n=2) | H(2)=2.08 | 0,354 | 0,683 |
| Derived inflammatory–nutritional indices | 120 | Albumin/CRP ratio | 6 months after discharge | 10.32 (6.60–10.97) (n=13) | 2.57 (2.57–2.57) (n=1) | 1.28 (0.72–1.84) (n=2) | H(2)=4.53 | 0,104 | 0,429 |
| Derived inflammatory–nutritional indices | 121 | CRP/Albumin ratio | Preoperative | 2.30 ± 2.12 (n=14) | 1.14 ± 0.94 (n=5) | 4.80 ± 3.84 (n=3) | F(2, 19)=2.63 | 0,098 | 0,429 |
| Derived inflammatory–nutritional indices | 122 | CRP/Albumin ratio | 3 months after discharge | 836.82 (0.75–925.93) (n=13) | 1.24 (1.22–467.91) (n=3) | 0.92 (0.68–1.15) (n=2) | H(2)=1.30 | 0,523 | 0,762 |
| Derived inflammatory–nutritional indices | 123 | CRP/Albumin ratio | 6 months after discharge | 811.36 (0.18–911.16) (n=13) | 0.39 (0.39–0.39) (n=1) | 3.29 (1.85–4.73) (n=2) | H(2)=0.73 | 0,694 | 0,876 |
| Derived inflammatory–nutritional indices | 124 | CRP/Lymphocytes ratio | Preoperative | 2.77 (1.24–8.23) (n=14) | 2.29 (0.93–2.64) (n=5) | 7.31 (4.81–12.91) (n=3) | H(2)=2.31 | 0,316 | 0,683 |
| Derived inflammatory–nutritional indices | 125 | CRP/Lymphocytes ratio | 3 months after discharge | 0.29 (0.20–2.27) (n=13) | 1.33 (0.73–1.87) (n=3) | 1.46 (1.11–1.81) (n=2) | H(2)=0.39 | 0,823 | 0,908 |
| Derived inflammatory–nutritional indices | 126 | CRP/Lymphocytes ratio | 6 months after discharge | 0.21 (0.15–0.39) (n=13) | 0.51 (0.51–0.51) (n=1) | 7.03 (5.05–9.01) (n=2) | H(2)=4.17 | 0,125 | 0,429 |
| Derived inflammatory–nutritional indices | 127 | Ferritin/Albumin ratio | Preoperative | 123.45 (50.64–254.44) (n=14) | 54.25 (25.74–139.32) (n=5) | 192.57 (119.78–249.88) (n=3) | H(2)=1.06 | 0,588 | 0,784 |
| Derived inflammatory–nutritional indices | 128 | Ferritin/Albumin ratio | 3 months after discharge | 58.94 (41.82–111.64) (n=13) | 59.04 (31.82–187.57) (n=3) | 130.59 (85.33–175.85) (n=2) | H(2)=0.19 | 0,908 | 0,908 |
| Derived inflammatory–nutritional indices | 129 | Ferritin/Albumin ratio | 6 months after discharge | 50.73 (30.08–105.12) (n=13) | 18.65 (18.65–18.65) (n=1) | 204.52 (126.81–282.24) (n=2) | H(2)=2.42 | 0,298 | 0,683 |
| Derived inflammatory–nutritional indices | 130 | Ferritin/Lymphocytes ratio | Preoperative | 389.16 (70.03–486.31) (n=14) | 138.94 (47.24–294.11) (n=5) | 345.77 (230.97–723.10) (n=3) | H(2)=0.63 | 0,730 | 0,876 |
| Derived inflammatory–nutritional indices | 131 | Ferritin/Lymphocytes ratio | 3 months after discharge | 103.87 (78.10–153.69) (n=13) | 63.26 (35.15–346.15) (n=3) | 222.30 (142.15–302.46) (n=2) | H(2)=0.29 | 0,866 | 0,908 |
| Derived inflammatory–nutritional indices | 132 | Ferritin/Lymphocytes ratio | 6 months after discharge | 93.00 (61.30–131.14) (n=13) | 24.59 (24.59–24.59) (n=1) | 501.79 (431.73–571.85) (n=2) | H(2)=5.90 | 0,052 | 0,416 |
| Derived inflammatory–nutritional indices | 133 | Albumin-Lymphocytes-CRP index | Preoperative | 1.45 (0.45–3.78) (n=14) | 1.44 (1.36–4.39) (n=5) | 0.60 (0.42–1.16) (n=3) | H(2)=1.48 | 0,477 | 0,762 |
| Derived inflammatory–nutritional indices | 134 | Albumin-Lymphocytes-CRP index | 3 months after discharge | 19.14 (3.20–34.56) (n=13) | 2.83 (2.24–16.39) (n=3) | 3.28 (2.32–4.24) (n=2) | H(2)=1.99 | 0,370 | 0,683 |
| Derived inflammatory–nutritional indices | 135 | Albumin-Lymphocytes-CRP index | 6 months after discharge | 20.85 (10.33–34.36) (n=13) | 8.23 (8.23–8.23) (n=1) | 0.82 (0.51–1.13) (n=2) | H(2)=5.90 | 0,052 | 0,416 |
| Notes: Values are presented as mean +/- standard deviation or median (P25-P75), with the valid n shown for each group and comparison. The analysis used observed available data only; no multiple imputation was performed. The number of patients evaluated was preoperative n = 24, 3 months after discharge n = 18, and 6 months after discharge n = 16. Raw p-values correspond to the statistical test reported in each row. BH-FDR adjusted p-values were calculated with the Benjamini-Hochberg false discovery rate procedure within each Exposure x Domain family: Dietary intake (45 tests), Clinical-nutritional laboratory analyses (66 tests), and Derived inflammatory-nutritional indices (24 tests). The BH-FDR audit found 945 inferential comparisons, 43 raw p-values < 0.05, and 0 BH-FDR adjusted p-values < 0.05. For binary exposures, two-sample t-test/Welch t-test or Mann-Whitney/Wilcoxon rank-sum tests were used as reported. For multicategorical or ordinal exposures, one-way ANOVA or Kruskal-Wallis tests were used; these are omnibus tests and do not establish pairwise differences or dose-response trends without post-hoc or contrast testing. Because this was an exploratory supplementary analysis with multiple comparisons, small subgroup sizes, and no covariate adjustment, the results should be interpreted as hypothesis-generating rather than confirmatory. Abbreviations: BH-FDR, Benjamini-Hochberg false discovery rate; DGE, delayed gastric emptying; ERCP, endoscopic retrograde cholangiopancreatography; TIBC, total iron-binding capacity; UIBC, unsaturated iron-binding capacity. | | | | | | | | | |

| **Supplementary Table S7.** Exploratory associations between clinically relevant postoperative pancreatic fistula and dietary intake, clinical-nutritional laboratory variables, and inflammatory-nutritional indices after pancreatoduodenectomy. | | | | | | | | |
| --- | --- | --- | --- | --- | --- | --- | --- | --- |
| **Outcome** | | | | **Clinically relevant postoperative pancreatic fistula** | | | | |
| **Domain** | **Source row** | **Variable** | **Time** | **Yes** | **No** | **Statistical result** | **Raw p-value** | **BH-FDR adjusted p-value** |
|  |  |  |  | **Mean ± SD or Median (P25–P75), n** | **Mean ± SD or Median (P25–P75), n** |  |  |  |
| Dietary intake | 1 | Energy (kcal) | Preoperative | 2669.05 ± 618.50 (n=16) | 2799.83 ± 505.75 (n=8) | t(22)=-0.52; Δ_mean=-130.78; 95% IC [-656.11 ; 394.54] | 0,611 | 0,917 |
| Dietary intake | 2 | Energy (kcal) | 3 months after discharge | 2073.14 ± 369.01 (n=13) | 1954.82 ± 234.54 (n=5) | t(16)=0.66; Δ_mean=118.32; 95% IC [-261.43 ; 498.07] | 0,518 | 0,917 |
| Dietary intake | 3 | Energy (kcal) | 6 months after discharge | 2038.00 ± 368.32 (n=12) | 1922.90 ± 224.47 (n=4) | t(14)=0.58; Δ_mean=115.10; 95% IC [-309.17 ; 539.36] | 0,570 | 0,917 |
| Dietary intake | 4 | Protein (g) | Preoperative | 116.48 ± 26.12 (n=16) | 118.78 ± 17.09 (n=8) | t(22)=-0.23; Δ_mean=-2.30; 95% IC [-23.52 ; 18.91] | 0,824 | 0,917 |
| Dietary intake | 5 | Protein (g) | 3 months after discharge | 98.45 ± 16.05 (n=13) | 91.77 ± 7.73 (n=5) | t(16)=0.88; Δ_mean=6.67; 95% IC [-9.42 ; 22.76] | 0,392 | 0,917 |
| Dietary intake | 6 | Protein (g) | 6 months after discharge | 101.68 ± 17.89 (n=12) | 99.15 ± 6.18 (n=4) | t(14)=0.27; Δ_mean=2.53; 95% IC [-17.43 ; 22.48] | 0,790 | 0,917 |
| Dietary intake | 7 | Relative energy balance (kcal) | Preoperative | 1135.35 ± 325.88 (n=16) | 1070.97 ± 325.62 (n=8) | t(22)=0.46; Δ_mean=64.38; 95% IC [-228.19 ; 356.95] | 0,653 | 0,917 |
| Dietary intake | 8 | Relative energy balance (kcal) | 3 months after discharge | 578.90 ± 275.25 (n=13) | 498.98 ± 151.56 (n=5) | t(16)=0.61; Δ_mean=79.93; 95% IC [-199.11 ; 358.96] | 0,552 | 0,917 |
| Dietary intake | 9 | Relative energy balance (kcal) | 6 months after discharge | 665.22 ± 266.16 (n=12) | 639.58 ± 52.66 (n=4) | t(14)=0.19; Δ_mean=25.64; 95% IC [-268.05 ; 319.34] | 0,854 | 0,917 |
| Dietary intake | 10 | Carbohydrate (g) | Preoperative | 367.25 ± 82.90 (n=16) | 392.33 ± 73.19 (n=8) | t(22)=-0.72; Δ_mean=-25.08; 95% IC [-96.87 ; 46.70] | 0,476 | 0,917 |
| Dietary intake | 11 | Carbohydrate (g) | 3 months after discharge | 265.58 ± 43.79 (n=13) | 258.00 ± 31.54 (n=5) | t(16)=0.35; Δ_mean=7.58; 95% IC [-38.24 ; 53.39] | 0,730 | 0,917 |
| Dietary intake | 12 | Carbohydrate (g) | 6 months after discharge | 263.23 ± 43.73 (n=12) | 258.46 ± 25.49 (n=4) | t(14)=0.20; Δ_mean=4.77; 95% IC [-45.41 ; 54.94] | 0,841 | 0,917 |
| Dietary intake | 13 | Lipid (g) | Preoperative | 78.25 (62.63–96.20) (n=16) | 82.07 (75.03–84.50) (n=8) | W=52.00; Δ_HL=-2.51; 95% IC [-20.62 ; 17.64] | 0,481 | 0,917 |
| Dietary intake | 14 | Lipid (g) | 3 months after discharge | 68.56 ± 18.05 (n=13) | 61.75 ± 12.24 (n=5) | t(16)=0.77; Δ_mean=6.81; 95% IC [-11.91 ; 25.54] | 0,452 | 0,917 |
| Dietary intake | 15 | Lipid (g) | 6 months after discharge | 64.26 ± 17.34 (n=12) | 54.72 ± 17.09 (n=4) | t(14)=0.96; Δ_mean=9.54; 95% IC [-11.87 ; 30.95] | 0,355 | 0,917 |
| Dietary intake | 16 | Fiber (g) | Preoperative | 44.81 ± 11.71 (n=16) | 48.48 ± 10.76 (n=8) | t(22)=-0.74; Δ_mean=-3.67; 95% IC [-13.92 ; 6.58] | 0,466 | 0,917 |
| Dietary intake | 17 | Fiber (g) | 3 months after discharge | 39.30 ± 8.69 (n=13) | 35.13 ± 10.84 (n=5) | t(16)=0.85; Δ_mean=4.17; 95% IC [-6.17 ; 14.52] | 0,405 | 0,917 |
| Dietary intake | 18 | Fiber (g) | 6 months after discharge | 37.78 ± 7.65 (n=12) | 34.52 ± 12.60 (n=4) | t(14)=0.63; Δ_mean=3.26; 95% IC [-7.81 ; 14.34] | 0,538 | 0,917 |
| Dietary intake | 19 | Calcium (mg) | Preoperative | 1411.33 ± 319.57 (n=16) | 1603.31 ± 270.14 (n=8) | t(22)=-1.45; Δ_mean=-191.98; 95% IC [-465.62 ; 81.66] | 0,160 | 0,917 |
| Dietary intake | 20 | Calcium (mg) | 3 months after discharge | 1328.61 ± 427.13 (n=13) | 1262.64 ± 279.62 (n=5) | t(16)=0.32; Δ_mean=65.97; 95% IC [-375.18 ; 507.11] | 0,755 | 0,917 |
| Dietary intake | 21 | Calcium (mg) | 6 months after discharge | 1268.26 ± 370.22 (n=12) | 1216.69 ± 325.26 (n=4) | t(14)=0.25; Δ_mean=51.57; 95% IC [-395.52 ; 498.66] | 0,808 | 0,917 |
| Dietary intake | 22 | Iron (mg) | Preoperative | 26.61 ± 7.27 (n=16) | 28.02 ± 5.55 (n=8) | t(22)=-0.48; Δ_mean=-1.42; 95% IC [-7.49 ; 4.66] | 0,634 | 0,917 |
| Dietary intake | 23 | Iron (mg) | 3 months after discharge | 22.24 ± 6.39 (n=13) | 28.20 ± 11.98 (n=5) | t(16)=-1.39; Δ_mean=-5.96; 95% IC [-15.06 ; 3.14] | 0,184 | 0,917 |
| Dietary intake | 24 | Iron (mg) | 6 months after discharge | 20.97 ± 4.96 (n=12) | 29.63 ± 14.31 (n=4) | t(14)=-1.89; Δ_mean=-8.66; 95% IC [-18.50 ; 1.18] | 0,080 | 0,917 |
| Dietary intake | 25 | Potassium (mg) | Preoperative | 6439.17 ± 1286.74 (n=16) | 7213.94 ± 1295.74 (n=8) | t(22)=-1.39; Δ_mean=-774.77; 95% IC [-1932.85 ; 383.31] | 0,179 | 0,917 |
| Dietary intake | 26 | Potassium (mg) | 3 months after discharge | 5146.09 (4743.40–7020.88) (n=13) | 5584.76 (5039.31–6858.33) (n=5) | W=30.00; Δ_HL=-140.12; 95% IC [-1724.55 ; 1198.24] | 0,844 | 0,917 |
| Dietary intake | 27 | Potassium (mg) | 6 months after discharge | 5495.19 ± 1233.77 (n=12) | 5745.89 ± 1036.11 (n=4) | t(14)=-0.36; Δ_mean=-250.69; 95% IC [-1729.43 ; 1228.04] | 0,722 | 0,917 |
| Dietary intake | 28 | Selenium (µg) | Preoperative | 55.06 ± 16.61 (n=16) | 62.36 ± 24.62 (n=8) | t(22)=-0.86; Δ_mean=-7.30; 95% IC [-24.83 ; 10.23] | 0,397 | 0,917 |
| Dietary intake | 29 | Selenium (µg) | 3 months after discharge | 44.78 (37.19–55.74) (n=13) | 39.32 (35.14–67.98) (n=5) | W=33.00; Δ_HL=0.18; 95% IC [-26.04 ; 18.95] | 1,000 | 1,000 |
| Dietary intake | 30 | Selenium (µg) | 6 months after discharge | 46.43 ± 21.14 (n=12) | 49.96 ± 17.41 (n=4) | t(14)=-0.30; Δ_mean=-3.53; 95% IC [-28.79 ; 21.73] | 0,769 | 0,917 |
| Dietary intake | 31 | Zinc (mg) | Preoperative | 17.24 (14.53–18.26) (n=16) | 17.95 (16.96–19.92) (n=8) | W=50.00; Δ_HL=-1.46; 95% IC [-6.35 ; 2.41] | 0,408 | 0,917 |
| Dietary intake | 32 | Zinc (mg) | 3 months after discharge | 14.81 ± 5.36 (n=13) | 14.27 ± 2.15 (n=5) | t(16)=0.22; Δ_mean=0.54; 95% IC [-4.77 ; 5.85] | 0,832 | 0,917 |
| Dietary intake | 33 | Zinc (mg) | 6 months after discharge | 13.69 ± 3.98 (n=12) | 14.12 ± 1.20 (n=4) | t(14)=-0.21; Δ_mean=-0.43; 95% IC [-4.85 ; 4.00] | 0,839 | 0,917 |
| Dietary intake | 34 | Sodium (mg) | Preoperative | 1656.51 (1267.89–2440.80) (n=16) | 1604.93 (1369.66–2476.06) (n=8) | W=66.00; Δ_HL=29.62; 95% IC [-715.44 ; 807.67] | 0,927 | 0,948 |
| Dietary intake | 35 | Sodium (mg) | 3 months after discharge | 1450.99 ± 435.37 (n=13) | 1151.39 ± 331.27 (n=5) | t(16)=1.38; Δ_mean=299.60; 95% IC [-159.81 ; 759.01] | 0,186 | 0,917 |
| Dietary intake | 36 | Sodium (mg) | 6 months after discharge | 1413.17 ± 428.28 (n=12) | 1043.45 ± 346.70 (n=4) | t(14)=1.55; Δ_mean=369.72; 95% IC [-140.66 ; 880.09] | 0,143 | 0,917 |
| Dietary intake | 37 | Vitamin A (mg) | Preoperative | 5.22 ± 2.94 (n=16) | 6.26 ± 2.65 (n=8) | t(22)=-0.85; Δ_mean=-1.05; 95% IC [-3.61 ; 1.52] | 0,407 | 0,917 |
| Dietary intake | 38 | Vitamin A (mg) | 3 months after discharge | 4.08 (2.89–6.93) (n=13) | 4.56 (3.92–7.34) (n=5) | W=30.00; Δ_HL=-0.13; 95% IC [-3.45 ; 4.27] | 0,844 | 0,917 |
| Dietary intake | 39 | Vitamin A (mg) | 6 months after discharge | 4.24 (2.84–5.23) (n=12) | 5.71 (4.13–7.17) (n=4) | W=19.50; Δ_HL=-0.87; 95% IC [-4.11 ; 2.99] | 0,627 | 0,917 |
| Dietary intake | 40 | Vitamin C (mg) | Preoperative | 371.59 ± 205.96 (n=16) | 407.28 ± 182.87 (n=8) | t(22)=-0.41; Δ_mean=-35.69; 95% IC [-214.31 ; 142.93] | 0,683 | 0,917 |
| Dietary intake | 41 | Vitamin C (mg) | 3 months after discharge | 337.47 (212.34–349.88) (n=13) | 273.36 (210.21–504.52) (n=5) | W=35.00; Δ_HL=12.44; 95% IC [-275.81 ; 149.98] | 0,844 | 0,917 |
| Dietary intake | 42 | Vitamin C (mg) | 6 months after discharge | 314.27 (211.08–353.83) (n=12) | 371.56 (256.24–494.24) (n=4) | W=22.00; Δ_HL=-45.28; 95% IC [-279.23 ; 166.00] | 0,856 | 0,917 |
| Dietary intake | 43 | Vitamin E (mg) | Preoperative | 4.32 ± 1.64 (n=16) | 5.07 ± 1.75 (n=8) | t(22)=-1.03; Δ_mean=-0.75; 95% IC [-2.26 ; 0.76] | 0,314 | 0,917 |
| Dietary intake | 44 | Vitamin E (mg) | 3 months after discharge | 4.52 ± 2.48 (n=13) | 4.67 ± 1.82 (n=5) | t(16)=-0.12; Δ_mean=-0.15; 95% IC [-2.76 ; 2.45] | 0,903 | 0,945 |
| Dietary intake | 45 | Vitamin E (mg) | 6 months after discharge | 4.17 ± 2.15 (n=12) | 3.61 ± 2.08 (n=4) | t(14)=0.45; Δ_mean=0.56; 95% IC [-2.09 ; 3.21] | 0,658 | 0,917 |
| Clinical–Nutritional Laboratory Analyses | 46 | Total Proteins (g/dL) | Preoperative | 6.86 (6.63–7.02) (n=16) | 6.90 (6.83–7.54) (n=8) | W=46.00; Δ_HL=-0.15; 95% IC [-0.90 ; 0.11] | 0,283 | 0,812 |
| Clinical–Nutritional Laboratory Analyses | 47 | Total Proteins (g/dL) | 3 months after discharge after discharge | 6.88 ± 0.53 (n=13) | 6.88 ± 0.33 (n=5) | t(16)=-0.01; Δ_mean=-0.00; 95% IC [-0.55 ; 0.54] | 0,994 | 1,000 |
| Clinical–Nutritional Laboratory Analyses | 48 | Total Proteins (g/dL) | 6 months after discharge after discharge | 6.60 ± 0.50 (n=12) | 6.82 ± 0.56 (n=4) | t(14)=-0.73; Δ_mean=-0.22; 95% IC [-0.86 ; 0.42] | 0,477 | 0,838 |
| Clinical–Nutritional Laboratory Analyses | 49 | Albumin (g/dL) | Preoperative | 3.74 ± 0.60 (n=16) | 4.37 ± 0.40 (n=8) | t(22)=-2.68; Δ_mean=-0.63; 95% IC [-1.12 ; -0.14] | 0,014 | 0,462 |
| Clinical–Nutritional Laboratory Analyses | 50 | Albumin (g/dL) | 3 months after discharge after discharge | 4.10 ± 0.53 (n=13) | 4.12 ± 0.55 (n=5) | t(16)=-0.06; Δ_mean=-0.02; 95% IC [-0.62 ; 0.58] | 0,949 | 0,996 |
| Clinical–Nutritional Laboratory Analyses | 51 | Albumin (g/dL) | 6 months after discharge after discharge | 4.25 (4.09–4.72) (n=12) | 4.12 (3.80–4.42) (n=4) | W=30.00; Δ_HL=0.29; 95% IC [-0.42 ; 0.87] | 0,505 | 0,838 |
| Clinical–Nutritional Laboratory Analyses | 52 | C-Reactive Protein (mg/L) | Preoperative | 7.75 (3.65–20.92) (n=16) | 5.00 (1.45–13.09) (n=8) | W=80.00; Δ_HL=2.54; 95% IC [-4.10 ; 11.84] | 0,342 | 0,818 |
| Clinical–Nutritional Laboratory Analyses | 53 | C-Reactive Protein (mg/L) | 3 months after discharge after discharge | 1.76 (0.40–4.09) (n=13) | 0.40 (0.40–0.40) (n=5) | W=44.00; Δ_HL=0.36; 95% IC [-0.49 ; 3.69] | 0,257 | 0,792 |
| Clinical–Nutritional Laboratory Analyses | 54 | C-Reactive Protein (mg/L) | 6 months after discharge after discharge | 0.66 (0.40–2.24) (n=12) | 0.40 (0.40–0.40) (n=4) | W=38.00; Δ_HL=0.33; 95% IC [0.00 ; 3.53] | 0,071 | 0,586 |
| Clinical–Nutritional Laboratory Analyses | 55 | Blood Glucose (mg/dL) | Preoperative | 116.54 (98.00–152.60) (n=16) | 123.22 (95.13–159.00) (n=8) | W=65.50; Δ_HL=1.20; 95% IC [-37.45 ; 55.94] | 0,951 | 0,996 |
| Clinical–Nutritional Laboratory Analyses | 56 | Blood Glucose (mg/dL) | 3 months after discharge after discharge | 118.00 (84.26–133.95) (n=13) | 128.23 (91.13–134.93) (n=5) | W=24.00; Δ_HL=-8.41; 95% IC [-50.67 ; 36.79] | 0,430 | 0,838 |
| Clinical–Nutritional Laboratory Analyses | 57 | Blood Glucose (mg/dL) | 6 months after discharge after discharge | 100.92 ± 22.38 (n=12) | 125.03 ± 54.65 (n=4) | t(14)=-1.30; Δ_mean=-24.11; 95% IC [-63.93 ; 15.70] | 0,215 | 0,739 |
| Clinical–Nutritional Laboratory Analyses | 58 | Hemoglobin (g/dL) | Preoperative | 12.13 ± 1.94 (n=16) | 13.60 ± 2.65 (n=8) | t(22)=-1.55; Δ_mean=-1.47; 95% IC [-3.43 ; 0.50] | 0,135 | 0,655 |
| Clinical–Nutritional Laboratory Analyses | 59 | Hemoglobin (g/dL) | 3 months after discharge after discharge | 12.20 ± 1.73 (n=13) | 13.51 ± 0.70 (n=5) | t(16)=-1.62; Δ_mean=-1.31; 95% IC [-3.03 ; 0.41] | 0,125 | 0,655 |
| Clinical–Nutritional Laboratory Analyses | 60 | Hemoglobin (g/dL) | 6 months after discharge after discharge | 12.11 ± 2.38 (n=12) | 12.75 ± 1.57 (n=4) | t(14)=-0.50; Δ_mean=-0.64; 95% IC [-3.40 ; 2.12] | 0,627 | 0,844 |
| Clinical–Nutritional Laboratory Analyses | 61 | Lymphocytes (x10³/mm³) | Preoperative | 2.21 ± 1.42 (n=16) | 2.27 ± 0.85 (n=8) | t(22)=-0.11; Δ_mean=-0.06; 95% IC [-1.20 ; 1.08] | 0,910 | 0,996 |
| Clinical–Nutritional Laboratory Analyses | 62 | Lymphocytes (x10³/mm³) | 3 months after discharge after discharge | 2.37 ± 1.03 (n=13) | 2.82 ± 0.58 (n=5) | t(16)=-0.92; Δ_mean=-0.45; 95% IC [-1.49 ; 0.59] | 0,372 | 0,818 |
| Clinical–Nutritional Laboratory Analyses | 63 | Lymphocytes (x10³/mm³) | 6 months after discharge after discharge | 2.33 ± 1.11 (n=12) | 2.62 ± 0.85 (n=4) | t(14)=-0.47; Δ_mean=-0.29; 95% IC [-1.60 ; 1.02] | 0,642 | 0,844 |
| Clinical–Nutritional Laboratory Analyses | 64 | Serum Iron (µg/dL) | Preoperative | 84.24 ± 37.26 (n=16) | 93.72 ± 41.79 (n=8) | t(22)=-0.56; Δ_mean=-9.48; 95% IC [-44.29 ; 25.33] | 0,578 | 0,844 |
| Clinical–Nutritional Laboratory Analyses | 65 | Serum Iron (µg/dL) | 3 months after discharge after discharge | 101.51 ± 62.52 (n=13) | 81.57 ± 28.27 (n=5) | t(16)=0.68; Δ_mean=19.94; 95% IC [-42.48 ; 82.37] | 0,508 | 0,838 |
| Clinical–Nutritional Laboratory Analyses | 66 | Serum Iron (µg/dL) | 6 months after discharge after discharge | 86.35 ± 49.02 (n=12) | 95.47 ± 7.00 (n=4) | t(14)=-0.36; Δ_mean=-9.12; 95% IC [-63.07 ; 44.83] | 0,722 | 0,851 |
| Clinical–Nutritional Laboratory Analyses | 67 | Ferritin (ng/mL) | Preoperative | 556.75 (81.05–969.97) (n=16) | 259.60 (181.27–529.40) (n=8) | W=70.50; Δ_HL=119.49; 95% IC [-198.40 ; 726.00] | 0,713 | 0,851 |
| Clinical–Nutritional Laboratory Analyses | 68 | Ferritin (ng/mL) | 3 months after discharge after discharge | 234.30 (183.60–477.80) (n=13) | 254.60 (45.10–399.60) (n=5) | W=40.00; Δ_HL=128.00; 95% IC [-215.70 ; 432.70] | 0,490 | 0,838 |
| Clinical–Nutritional Laboratory Analyses | 69 | Ferritin (ng/mL) | 6 months after discharge after discharge | 295.61 ± 207.21 (n=12) | 191.91 ± 153.02 (n=4) | t(14)=0.91; Δ_mean=103.70; 95% IC [-140.06 ; 347.47] | 0,377 | 0,818 |
| Clinical–Nutritional Laboratory Analyses | 70 | UIBC (µg/dL) | Preoperative | 283.89 ± 84.07 (n=16) | 295.30 ± 34.84 (n=8) | t(22)=-0.37; Δ_mean=-11.41; 95% IC [-76.20 ; 53.38] | 0,718 | 0,851 |
| Clinical–Nutritional Laboratory Analyses | 71 | UIBC (µg/dL) | 3 months after discharge after discharge | 288.95 ± 54.79 (n=13) | 323.65 ± 43.37 (n=5) | t(16)=-1.26; Δ_mean=-34.70; 95% IC [-92.90 ; 23.51] | 0,224 | 0,739 |
| Clinical–Nutritional Laboratory Analyses | 72 | UIBC (µg/dL) | 6 months after discharge after discharge | 298.62 ± 44.28 (n=12) | 309.48 ± 22.95 (n=4) | t(14)=-0.46; Δ_mean=-10.86; 95% IC [-61.22 ; 39.49] | 0,651 | 0,844 |
| Clinical–Nutritional Laboratory Analyses | 73 | TIBC (µg/dL) | Preoperative | 368.13 ± 94.97 (n=16) | 389.02 ± 65.24 (n=8) | t(22)=-0.56; Δ_mean=-20.89; 95% IC [-98.67 ; 56.90] | 0,583 | 0,844 |
| Clinical–Nutritional Laboratory Analyses | 74 | TIBC (µg/dL) | 3 months after discharge after discharge | 390.46 ± 106.81 (n=13) | 405.21 ± 41.63 (n=5) | t(16)=-0.30; Δ_mean=-14.75; 95% IC [-120.52 ; 91.02] | 0,771 | 0,877 |
| Clinical–Nutritional Laboratory Analyses | 75 | TIBC (µg/dL) | 6 months after discharge after discharge | 384.97 ± 70.27 (n=12) | 404.95 ± 18.19 (n=4) | t(13.82)=-0.90; Δ_mean=-19.98; 95% IC [-67.72 ; 27.76] | 0,384 | 0,818 |
| Clinical–Nutritional Laboratory Analyses | 76 | Transferrin (mg/dL) | Preoperative | 227.11 ± 67.26 (n=16) | 236.24 ± 27.87 (n=8) | t(22)=-0.37; Δ_mean=-9.13; 95% IC [-60.96 ; 42.70] | 0,718 | 0,851 |
| Clinical–Nutritional Laboratory Analyses | 77 | Transferrin (mg/dL) | 3 months after discharge after discharge | 231.16 ± 43.83 (n=13) | 258.92 ± 34.70 (n=5) | t(16)=-1.26; Δ_mean=-27.76; 95% IC [-74.32 ; 18.80] | 0,224 | 0,739 |
| Clinical–Nutritional Laboratory Analyses | 78 | Transferrin (mg/dL) | 6 months after discharge after discharge | 238.90 ± 35.43 (n=12) | 247.59 ± 18.36 (n=4) | t(14)=-0.46; Δ_mean=-8.69; 95% IC [-48.98 ; 31.59] | 0,651 | 0,844 |
| Clinical–Nutritional Laboratory Analyses | 79 | Transferrin Saturation (%) | Preoperative | 22.94 ± 8.63 (n=16) | 23.32 ± 7.42 (n=8) | t(22)=-0.11; Δ_mean=-0.38; 95% IC [-7.80 ; 7.04] | 0,917 | 0,996 |
| Clinical–Nutritional Laboratory Analyses | 80 | Transferrin Saturation (%) | 3 months after discharge after discharge | 24.00 ± 9.95 (n=13) | 20.10 ± 6.48 (n=5) | t(16)=0.80; Δ_mean=3.90; 95% IC [-6.37 ; 14.17] | 0,433 | 0,838 |
| Clinical–Nutritional Laboratory Analyses | 81 | Transferrin Saturation (%) | 6 months after discharge after discharge | 21.42 ± 9.40 (n=12) | 23.64 ± 2.44 (n=4) | t(14)=-0.46; Δ_mean=-2.22; 95% IC [-12.64 ; 8.20] | 0,655 | 0,844 |
| Clinical–Nutritional Laboratory Analyses | 82 | Urea (mg/dL) | Preoperative | 28.88 (23.79–41.40) (n=16) | 35.25 (33.99–40.85) (n=8) | W=52.00; Δ_HL=-6.01; 95% IC [-17.32 ; 10.35] | 0,481 | 0,838 |
| Clinical–Nutritional Laboratory Analyses | 83 | Urea (mg/dL) | 3 months after discharge after discharge | 32.46 (26.43–51.96) (n=13) | 35.59 (35.01–36.42) (n=5) | W=33.00; Δ_HL=8.82; 95% IC [-11.09 ; 29.90] | 1,000 | 1,000 |
| Clinical–Nutritional Laboratory Analyses | 84 | Urea (mg/dL) | 6 months after discharge after discharge | 33.70 (25.40–45.01) (n=12) | 20.96 (18.24–25.04) (n=4) | W=41.00; Δ_HL=12.08; 95% IC [0.47 ; 34.62] | 0,045 | 0,583 |
| Clinical–Nutritional Laboratory Analyses | 85 | Creatinine (mg/dL) | Preoperative | 0.91 (0.83–1.26) (n=16) | 0.94 (0.73–1.02) (n=8) | W=76.50; Δ_HL=0.11; 95% IC [-0.15 ; 0.52] | 0,462 | 0,838 |
| Clinical–Nutritional Laboratory Analyses | 86 | Creatinine (mg/dL) | 3 months after discharge after discharge | 0.87 (0.75–0.96) (n=13) | 0.77 (0.73–0.87) (n=5) | W=42.00; Δ_HL=0.06; 95% IC [-0.13 ; 0.28] | 0,375 | 0,818 |
| Clinical–Nutritional Laboratory Analyses | 87 | Creatinine (mg/dL) | 6 months after discharge after discharge | 0.92 (0.78–1.11) (n=12) | 0.86 (0.83–0.89) (n=4) | W=29.50; Δ_HL=0.10; 95% IC [-0.14 ; 1.14] | 0,543 | 0,844 |
| Clinical–Nutritional Laboratory Analyses | 88 | Aspartate Aminotransferase (AST) (U/L) | Preoperative | 217.78 ± 151.54 (n=16) | 85.21 ± 56.88 (n=8) | t(22)=2.37; Δ_mean=132.57; 95% IC [16.57 ; 248.57] | 0,027 | 0,583 |
| Clinical–Nutritional Laboratory Analyses | 89 | Aspartate Aminotransferase (AST) (U/L) | 3 months after discharge after discharge | 25.69 (22.31–35.43) (n=13) | 36.91 (29.79–36.98) (n=5) | W=17.00; Δ_HL=-9.44; 95% IC [-14.67 ; 6.01] | 0,139 | 0,655 |
| Clinical–Nutritional Laboratory Analyses | 90 | Aspartate Aminotransferase (AST) (U/L) | 6 months after discharge after discharge | 31.61 ± 8.46 (n=12) | 40.43 ± 18.90 (n=4) | t(14)=-1.33; Δ_mean=-8.83; 95% IC [-23.09 ; 5.44] | 0,206 | 0,739 |
| Clinical–Nutritional Laboratory Analyses | 91 | Aminotransferase (ALT) (U/L) | Preoperative | 173.27 (89.81–253.93) (n=16) | 102.81 (51.60–224.45) (n=8) | W=81.00; Δ_HL=52.13; 95% IC [-72.71 ; 183.37] | 0,312 | 0,818 |
| Clinical–Nutritional Laboratory Analyses | 92 | Aminotransferase (ALT) (U/L) | 3 months after discharge after discharge | 31.88 ± 15.07 (n=13) | 45.67 ± 11.22 (n=5) | t(16)=-1.84; Δ_mean=-13.78; 95% IC [-29.63 ; 2.06] | 0,084 | 0,616 |
| Clinical–Nutritional Laboratory Analyses | 93 | Aminotransferase (ALT) (U/L) | 6 months after discharge after discharge | 34.82 (25.84–40.15) (n=12) | 39.30 (29.00–48.99) (n=4) | W=21.00; Δ_HL=-4.76; 95% IC [-20.79 ; 17.50] | 0,762 | 0,877 |
| Clinical–Nutritional Laboratory Analyses | 94 | Folic Acid (ng/mL) | Preoperative | 10.55 (9.70–15.53) (n=16) | 17.59 (15.40–19.50) (n=8) | W=32.00; Δ_HL=-5.61; 95% IC [-9.62 ; 0.00] | 0,053 | 0,583 |
| Clinical–Nutritional Laboratory Analyses | 95 | Folic Acid (ng/mL) | 3 months after discharge after discharge | 14.74 (10.62–24.00) (n=13) | 24.00 (22.61–24.00) (n=5) | W=21.00; Δ_HL=-5.27; 95% IC [-13.38 ; 1.39] | 0,264 | 0,792 |
| Clinical–Nutritional Laboratory Analyses | 96 | Folic Acid (ng/mL) | 6 months after discharge after discharge | 22.47 (13.54–24.00) (n=12) | 21.17 (18.57–22.10) (n=4) | W=25.00; Δ_HL=0.00; 95% IC [-10.88 ; 6.22] | 0,951 | 0,996 |
| Clinical–Nutritional Laboratory Analyses | 97 | Vitamin B12 (pg/mL) | Preoperative | 625.00 (455.00–832.75) (n=16) | 412.50 (323.25–455.00) (n=8) | W=106.50; Δ_HL=233.00; 95% IC [88.00 ; 468.00] | 0,010 | 0,462 |
| Clinical–Nutritional Laboratory Analyses | 98 | Vitamin B12 (pg/mL) | 3 months after discharge after discharge | 538.00 (416.00–1047.00) (n=13) | 372.00 (333.00–487.00) (n=5) | W=48.00; Δ_HL=162.00; 95% IC [-71.00 ; 734.00] | 0,139 | 0,655 |
| Clinical–Nutritional Laboratory Analyses | 99 | Vitamin B12 (pg/mL) | 6 months after discharge after discharge | 451.47 (338.76–544.50) (n=12) | 314.62 (313.00–397.44) (n=4) | W=32.00; Δ_HL=66.00; 95% IC [-126.00 ; 368.73] | 0,363 | 0,818 |
| Clinical–Nutritional Laboratory Analyses | 100 | Vitamin A (µg/dL) | Preoperative | 0.30 (0.19–0.33) (n=16) | 0.33 (0.21–0.37) (n=8) | W=47.00; Δ_HL=-0.03; 95% IC [-0.15 ; 0.09] | 0,309 | 0,818 |
| Clinical–Nutritional Laboratory Analyses | 101 | Vitamin A (µg/dL) | 3 months after discharge after discharge | 0.32 ± 0.11 (n=13) | 0.34 ± 0.10 (n=5) | t(16)=-0.44; Δ_mean=-0.03; 95% IC [-0.15 ; 0.10] | 0,665 | 0,844 |
| Clinical–Nutritional Laboratory Analyses | 102 | Vitamin A (µg/dL) | 6 months after discharge after discharge | 0.33 (0.28–0.38) (n=12) | 0.27 (0.25–0.30) (n=4) | W=35.50; Δ_HL=0.06; 95% IC [-0.02 ; 0.20] | 0,180 | 0,739 |
| Clinical–Nutritional Laboratory Analyses | 103 | Vitamin E (mg/dL) | Preoperative | 7.79 (6.95–9.03) (n=16) | 7.37 (6.11–8.22) (n=8) | W=72.00; Δ_HL=0.30; 95% IC [-1.28 ; 2.47] | 0,645 | 0,844 |
| Clinical–Nutritional Laboratory Analyses | 104 | Vitamin E (mg/dL) | 3 months after discharge after discharge | 6.46 ± 2.21 (n=13) | 7.13 ± 2.42 (n=5) | t(16)=-0.56; Δ_mean=-0.67; 95% IC [-3.20 ; 1.86] | 0,583 | 0,844 |
| Clinical–Nutritional Laboratory Analyses | 105 | Vitamin E (mg/dL) | 6 months after discharge after discharge | 5.82 (4.69–7.29) (n=12) | 5.26 (4.99–5.53) (n=4) | W=29.00; Δ_HL=0.56; 95% IC [-1.33 ; 4.57] | 0,585 | 0,844 |
| Clinical–Nutritional Laboratory Analyses | 106 | Copper (µg/dL) | Preoperative | 139.00 (110.15–152.45) (n=16) | 144.20 (100.30–154.15) (n=8) | W=65.00; Δ_HL=0.00; 95% IC [-39.20 ; 44.40] | 0,976 | 1,000 |
| Clinical–Nutritional Laboratory Analyses | 107 | Copper (µg/dL) | 3 months after discharge after discharge | 115.63 ± 32.63 (n=13) | 80.76 ± 18.30 (n=5) | t(16)=2.23; Δ_mean=34.87; 95% IC [1.73 ; 68.01] | 0,040 | 0,583 |
| Clinical–Nutritional Laboratory Analyses | 108 | Copper (µg/dL) | 6 months after discharge after discharge | 109.28 ± 29.09 (n=12) | 78.17 ± 15.95 (n=4) | t(14)=2.01; Δ_mean=31.11; 95% IC [-2.09 ; 64.32] | 0,064 | 0,586 |
| Clinical–Nutritional Laboratory Analyses | 109 | Zinc (µg/dL) | Preoperative | 84.75 (58.25–93.75) (n=16) | 89.00 (73.62–97.88) (n=8) | W=51.00; Δ_HL=-5.78; 95% IC [-27.00 ; 10.50] | 0,442 | 0,838 |
| Clinical–Nutritional Laboratory Analyses | 110 | Zinc (µg/dL) | 3 months after discharge after discharge | 81.88 ± 9.66 (n=13) | 96.70 ± 37.15 (n=5) | t(16)=-1.38; Δ_mean=-14.82; 95% IC [-37.54 ; 7.91] | 0,186 | 0,739 |
| Clinical–Nutritional Laboratory Analyses | 111 | Zinc (µg/dL) | 6 months after discharge after discharge | 81.40 ± 11.60 (n=12) | 68.36 ± 19.44 (n=4) | t(14)=1.65; Δ_mean=13.05; 95% IC [-3.88 ; 29.97] | 0,120 | 0,655 |
| Derived inflammatory–nutritional indices | 112 | Prognostic Nutritional Index | Preoperative | 48.48 ± 10.09 (n=16) | 55.10 ± 7.36 (n=8) | t(22)=-1.64; Δ_mean=-6.62; 95% IC [-14.99 ; 1.74] | 0,115 | 0,543 |
| Derived inflammatory–nutritional indices | 113 | Prognostic Nutritional Index | 3 months after discharge | 51.30 (49.20–57.90) (n=13) | 56.80 (56.20–59.20) (n=5) | W=26.00; Δ_HL=-1.50; 95% IC [-10.10 ; 6.50] | 0,554 | 0,665 |
| Derived inflammatory–nutritional indices | 114 | Prognostic Nutritional Index | 6 months after discharge | 58.13 (48.70–61.17) (n=12) | 54.45 (52.30–56.33) (n=4) | W=27.00; Δ_HL=2.17; 95% IC [-9.60 ; 9.20] | 0,762 | 0,795 |
| Derived inflammatory–nutritional indices | 115 | Glasgow Prognostic Score (GPS) | Preoperative | 0: 6 (37.5%); 1: 7 (43.8%); 2: 3 (18.8%) | 0: 5 (62.5%); 1: 3 (37.5%) | W=84.50; Δ_HL=0.00; 95% IC [-0.00 ; 1.00] | 0,179 | 0,543 |
| Derived inflammatory–nutritional indices | 116 | Glasgow Prognostic Score (GPS) | 3 months after discharge | 0: 11 (84.6%); 1: 2 (15.4%) | 0: 4 (80.0%); 1: 1 (20.0%) | W=31.00; Δ_HL=-0.00; 95% IC [-0.00 ; 0.00] | 0,879 | 0,879 |
| Derived inflammatory–nutritional indices | 117 | Glasgow Prognostic Score (GPS) | 6 months after discharge | 0: 11 (91.7%); 2: 1 (8.3%) | 0: 4 (100.0%) | W=26.00; Δ_HL=0.00; 95% IC [0.00 ; 0.00] | 0,665 | 0,725 |
| Derived inflammatory–nutritional indices | 118 | Albumin/CRP ratio | Preoperative | 0.46 (0.20–1.03) (n=16) | 0.83 (0.35–4.73) (n=8) | W=44.00; Δ_HL=-0.31; 95% IC [-4.37 ; 0.23] | 0,232 | 0,543 |
| Derived inflammatory–nutritional indices | 119 | Albumin/CRP ratio | 3 months after discharge | 2.26 (0.83–10.65) (n=13) | 10.70 (10.45–10.80) (n=5) | W=22.50; Δ_HL=-5.17; 95% IC [-9.87 ; 0.53] | 0,349 | 0,627 |
| Derived inflammatory–nutritional indices | 120 | Albumin/CRP ratio | 6 months after discharge | 6.28 ± 4.63 (n=12) | 10.26 ± 1.05 (n=4) | t(13.49)=-2.77; Δ_mean=-3.99; 95% IC [-7.08 ; -0.89] | 0,015 | 0,360 |
| Derived inflammatory–nutritional indices | 121 | CRP/Albumin ratio | Preoperative | 2.16 (1.02–5.08) (n=16) | 1.21 (0.30–2.94) (n=8) | W=84.50; Δ_HL=0.89; 95% IC [-0.76 ; 3.45] | 0,221 | 0,543 |
| Derived inflammatory–nutritional indices | 122 | CRP/Albumin ratio | 3 months after discharge | 1.24 (0.49–836.82) (n=13) | 925.93 (865.80–934.58) (n=5) | W=10.50; Δ_HL=-864.59; 95% IC [-934.09 ; -0.46] | 0,034 | 0,360 |
| Derived inflammatory–nutritional indices | 123 | CRP/Albumin ratio | 6 months after discharge | 3.49 (0.41–869.24) (n=12) | 441.56 (0.11–890.04) (n=4) | W=30.00; Δ_HL=0.29; 95% IC [-882.85 ; 847.35] | 0,505 | 0,638 |
| Derived inflammatory–nutritional indices | 124 | CRP/Lymphocytes ratio | Preoperative | 3.58 (1.31–9.76) (n=16) | 2.77 (0.44–7.12) (n=8) | W=77.00; Δ_HL=1.12; 95% IC [-2.48 ; 6.89] | 0,444 | 0,627 |
| Derived inflammatory–nutritional indices | 125 | CRP/Lymphocytes ratio | 3 months after discharge | 1.33 (0.29–2.40) (n=13) | 0.14 (0.12–0.20) (n=5) | W=52.50; Δ_HL=0.58; 95% IC [-0.00 ; 2.60] | 0,054 | 0,360 |
| Derived inflammatory–nutritional indices | 126 | CRP/Lymphocytes ratio | 6 months after discharge | 0.45 (0.26–2.24) (n=12) | 0.17 (0.14–0.20) (n=4) | W=40.00; Δ_HL=0.29; 95% IC [-0.02 ; 10.78] | 0,060 | 0,360 |
| Derived inflammatory–nutritional indices | 127 | Ferritin/Albumin ratio | Preoperative | 156.01 (25.41–282.74) (n=16) | 58.71 (40.25–114.72) (n=8) | W=78.00; Δ_HL=33.24; 95% IC [-38.96 ; 191.20] | 0,408 | 0,627 |
| Derived inflammatory–nutritional indices | 128 | Ferritin/Albumin ratio | 3 months after discharge | 59.04 (41.82–111.64) (n=13) | 58.94 (9.76–125.66) (n=5) | W=38.00; Δ_HL=32.77; 95% IC [-73.57 ; 101.87] | 0,622 | 0,711 |
| Derived inflammatory–nutritional indices | 129 | Ferritin/Albumin ratio | 6 months after discharge | 55.01 (36.68–106.73) (n=12) | 39.49 (23.63–65.55) (n=4) | W=30.00; Δ_HL=12.93; 95% IC [-43.83 ; 83.88] | 0,505 | 0,638 |
| Derived inflammatory–nutritional indices | 130 | Ferritin/Lymphocytes ratio | Preoperative | 389.16 (44.73–512.45) (n=16) | 111.52 (76.26–193.85) (n=8) | W=77.00; Δ_HL=206.24; 95% IC [-69.04 ; 398.05] | 0,444 | 0,627 |
| Derived inflammatory–nutritional indices | 131 | Ferritin/Lymphocytes ratio | 3 months after discharge | 103.87 (76.62–330.16) (n=13) | 79.56 (22.55–153.69) (n=5) | W=41.00; Δ_HL=54.96; 95% IC [-54.50 ; 323.12] | 0,430 | 0,627 |
| Derived inflammatory–nutritional indices | 132 | Ferritin/Lymphocytes ratio | 6 months after discharge | 117.51 (62.46–203.21) (n=12) | 70.20 (41.19–96.16) (n=4) | W=34.00; Δ_HL=40.39; 95% IC [-31.70 ; 263.78] | 0,249 | 0,543 |
| Derived inflammatory–nutritional indices | 133 | Albumin-Lymphocytes-CRP index | Preoperative | 1.10 (0.37–2.78) (n=16) | 1.49 (0.65–15.35) (n=8) | W=46.00; Δ_HL=-0.63; 95% IC [-26.93 ; 0.84] | 0,284 | 0,568 |
| Derived inflammatory–nutritional indices | 134 | Albumin-Lymphocytes-CRP index | 3 months after discharge | 5.20 (1.65–19.14) (n=13) | 29.96 (23.10–34.56) (n=5) | W=19.00; Δ_HL=-17.90; 95% IC [-31.36 ; 3.80] | 0,200 | 0,543 |
| Derived inflammatory–nutritional indices | 135 | Albumin-Lymphocytes-CRP index | 6 months after discharge | 16.46 ± 15.86 (n=12) | 26.58 ± 7.19 (n=4) | t(14)=-1.21; Δ_mean=-10.11; 95% IC [-28.00 ; 7.77] | 0,245 | 0,543 |
| Notes: Values are presented as mean +/- standard deviation or median (P25-P75), with the valid n shown for each group and comparison. The analysis used observed available data only; no multiple imputation was performed. The number of patients evaluated was preoperative n = 24, 3 months after discharge n = 18, and 6 months after discharge n = 16. Raw p-values correspond to the statistical test reported in each row. BH-FDR adjusted p-values were calculated with the Benjamini-Hochberg false discovery rate procedure within each Exposure x Domain family: Dietary intake (45 tests), Clinical-nutritional laboratory analyses (66 tests), and Derived inflammatory-nutritional indices (24 tests). The BH-FDR audit found 945 inferential comparisons, 43 raw p-values < 0.05, and 0 BH-FDR adjusted p-values < 0.05. For binary exposures, two-sample t-test/Welch t-test or Mann-Whitney/Wilcoxon rank-sum tests were used as reported. For multicategorical or ordinal exposures, one-way ANOVA or Kruskal-Wallis tests were used; these are omnibus tests and do not establish pairwise differences or dose-response trends without post-hoc or contrast testing. Because this was an exploratory supplementary analysis with multiple comparisons, small subgroup sizes, and no covariate adjustment, the results should be interpreted as hypothesis-generating rather than confirmatory. Clinically relevant POPF was defined as ISGPS grade B or C. Abbreviations: BH-FDR, Benjamini-Hochberg false discovery rate; POPF, postoperative pancreatic fistula; ISGPS, International Study Group of Pancreatic Surgery; PERT, pancreatic enzyme replacement therapy; POPF, postoperative pancreatic fistula; TIBC, total iron-binding capacity; UIBC, unsaturated iron-binding capacity. | | | | | | | | |

| **Supplementary Table S8.** Exploratory associations between POPF classification according to ISGPS and dietary intake, clinical-nutritional laboratory variables, and inflammatory-nutritional indices after pancreatoduodenectomy. | | | | | | | | | |
| --- | --- | --- | --- | --- | --- | --- | --- | --- | --- |
| **Outcome** | | | | **POPF classification according to ISGPS** | | | | |  |
| **Domain** | **Source row** | **Variable** | **Time** | **Biochemical leak** | **Grade B** | **Grade C** | **Statistical result** | **Raw p-value** | **BH-FDR adjusted p-value** |
|  |  |  |  | **Mean ± SD or Median (P25–P75), n** | **Mean ± SD or Median (P25–P75), n** | **Mean ± SD or Median (P25–P75), n** |  |  |  |
| Dietary intake | 1 | Energy (kcal) | Preoperative | 2825.35 ± 540.68 (n=7) | 2592.60 ± 623.65 (n=14) | 3009.85 ± 356.38 (n=3) | F(2, 21)=0.83 | 0,452 | 0,914 |
| Dietary intake | 2 | Energy (kcal) | 3 months after discharge | 1954.82 ± 234.54 (n=5) | 2073.14 ± 369.01 (n=13) | NA | t(16)=-0.66; Δ_mean=-118.32; 95% IC [-498.07 ; 261.43] | 0,518 | 0,914 |
| Dietary intake | 3 | Energy (kcal) | 6 months after discharge | 1922.90 ± 224.47 (n=4) | 2038.00 ± 368.32 (n=12) | NA | t(14)=-0.58; Δ_mean=-115.10; 95% IC [-539.36 ; 309.17] | 0,570 | 0,914 |
| Dietary intake | 4 | Protein (g) | Preoperative | 119.66 ± 18.27 (n=7) | 115.13 ± 27.70 (n=14) | 121.50 ± 9.24 (n=3) | F(2, 21)=0.14 | 0,873 | 0,914 |
| Dietary intake | 5 | Protein (g) | 3 months after discharge | 91.77 ± 7.73 (n=5) | 98.45 ± 16.05 (n=13) | NA | t(16)=-0.88; Δ_mean=-6.67; 95% IC [-22.76 ; 9.42] | 0,392 | 0,914 |
| Dietary intake | 6 | Protein (g) | 6 months after discharge | 99.15 ± 6.18 (n=4) | 101.68 ± 17.89 (n=12) | NA | t(14)=-0.27; Δ_mean=-2.53; 95% IC [-22.48 ; 17.43] | 0,790 | 0,914 |
| Dietary intake | 7 | Relative energy balance (kcal) | Preoperative | 1044.67 ± 342.41 (n=7) | 1108.04 ± 339.24 (n=14) | 1302.68 ± 91.65 (n=3) | F(2, 21)=0.67 | 0,523 | 0,914 |
| Dietary intake | 8 | Relative energy balance (kcal) | 3 months after discharge | 498.98 ± 151.56 (n=5) | 578.90 ± 275.25 (n=13) | NA | t(16)=-0.61; Δ_mean=-79.93; 95% IC [-358.96 ; 199.11] | 0,552 | 0,914 |
| Dietary intake | 9 | Relative energy balance (kcal) | 6 months after discharge | 639.58 ± 52.66 (n=4) | 665.22 ± 266.16 (n=12) | NA | t(14)=-0.19; Δ_mean=-25.64; 95% IC [-319.34 ; 268.05] | 0,854 | 0,914 |
| Dietary intake | 10 | Carbohydrate (g) | Preoperative | 395.75 ± 78.36 (n=7) | 356.92 ± 83.61 (n=14) | 415.85 ± 42.56 (n=3) | F(2, 21)=1.01 | 0,382 | 0,914 |
| Dietary intake | 11 | Carbohydrate (g) | 3 months after discharge | 258.00 ± 31.54 (n=5) | 265.58 ± 43.79 (n=13) | NA | t(16)=-0.35; Δ_mean=-7.58; 95% IC [-53.39 ; 38.24] | 0,730 | 0,914 |
| Dietary intake | 12 | Carbohydrate (g) | 6 months after discharge | 258.46 ± 25.49 (n=4) | 263.23 ± 43.73 (n=12) | NA | t(14)=-0.20; Δ_mean=-4.77; 95% IC [-54.94 ; 45.41] | 0,841 | 0,914 |
| Dietary intake | 13 | Lipid (g) | Preoperative | 84.86 ± 19.87 (n=7) | 78.27 ± 26.52 (n=14) | 95.61 ± 22.20 (n=3) | F(2, 21)=0.68 | 0,519 | 0,914 |
| Dietary intake | 14 | Lipid (g) | 3 months after discharge | 61.75 ± 12.24 (n=5) | 68.56 ± 18.05 (n=13) | NA | t(16)=-0.77; Δ_mean=-6.81; 95% IC [-25.54 ; 11.91] | 0,452 | 0,914 |
| Dietary intake | 15 | Lipid (g) | 6 months after discharge | 54.72 ± 17.09 (n=4) | 64.26 ± 17.34 (n=12) | NA | t(14)=-0.96; Δ_mean=-9.54; 95% IC [-30.95 ; 11.87] | 0,355 | 0,914 |
| Dietary intake | 16 | Fiber (g) | Preoperative | 48.07 ± 11.56 (n=7) | 42.75 ± 10.86 (n=14) | 56.59 ± 6.70 (n=3) | F(2, 21)=2.23 | 0,133 | 0,914 |
| Dietary intake | 17 | Fiber (g) | 3 months after discharge | 35.13 ± 10.84 (n=5) | 39.30 ± 8.69 (n=13) | NA | t(16)=-0.85; Δ_mean=-4.17; 95% IC [-14.52 ; 6.17] | 0,405 | 0,914 |
| Dietary intake | 18 | Fiber (g) | 6 months after discharge | 34.52 ± 12.60 (n=4) | 37.78 ± 7.65 (n=12) | NA | t(14)=-0.63; Δ_mean=-3.26; 95% IC [-14.34 ; 7.81] | 0,538 | 0,914 |
| Dietary intake | 19 | Calcium (mg) | Preoperative | 1630.88 ± 279.37 (n=7) | 1418.72 ± 340.40 (n=14) | 1376.52 ± 102.95 (n=3) | F(2, 21)=1.28 | 0,299 | 0,914 |
| Dietary intake | 20 | Calcium (mg) | 3 months after discharge | 1262.64 ± 279.62 (n=5) | 1328.61 ± 427.13 (n=13) | NA | t(16)=-0.32; Δ_mean=-65.97; 95% IC [-507.11 ; 375.18] | 0,755 | 0,914 |
| Dietary intake | 21 | Calcium (mg) | 6 months after discharge | 1216.69 ± 325.26 (n=4) | 1268.26 ± 370.22 (n=12) | NA | t(14)=-0.25; Δ_mean=-51.57; 95% IC [-498.66 ; 395.52] | 0,808 | 0,914 |
| Dietary intake | 22 | Iron (mg) | Preoperative | 28.39 (26.40–28.95) (n=7) | 24.09 (21.21–27.73) (n=14) | 29.76 (29.20–34.45) (n=3) | H(2)=5.100.741 | 0,078 | 0,914 |
| Dietary intake | 23 | Iron (mg) | 3 months after discharge | 28.20 ± 11.98 (n=5) | 22.24 ± 6.39 (n=13) | NA | t(16)=1.39; Δ_mean=5.96; 95% IC [-3.14 ; 15.06]0.933 | 0,184 | 0,914 |
| Dietary intake | 24 | Iron (mg) | 6 months after discharge | 29.63 ± 14.31 (n=4) | 20.97 ± 4.96 (n=12) | NA | t(14)=1.89; Δ_mean=8.66; 95% IC [-1.18 ; 18.50]0.741 | 0,080 | 0,914 |
| Dietary intake | 25 | Potassium (mg) | Preoperative | 7268.41 ± 1389.62 (n=7) | 6291.80 ± 1308.40 (n=14) | 7258.04 ± 458.03 (n=3) | F(2, 21)=1.690.948 | 0,208 | 0,914 |
| Dietary intake | 26 | Potassium (mg) | 3 months after discharge | 5584.76 (5039.31–6858.33) (n=5) | 5146.09 (4743.40–7020.88) (n=13) | NA | W=35.00; Δ_HL=140.12; 95% IC [-1198.24 ; 1724.55] | 0,844 | 0,914 |
| Dietary intake | 27 | Potassium (mg) | 6 months after discharge | 5745.89 ± 1036.11 (n=4) | 5495.19 ± 1233.77 (n=12) | NA | t(14)=0.36; Δ_mean=250.69; 95% IC [-1228.04 ; 1729.43] | 0,722 | 0,914 |
| Dietary intake | 28 | Selenium (µg) | Preoperative | 66.54 ± 23.33 (n=7) | 55.47 ± 17.63 (n=14) | 45.80 ± 12.62 (n=3) | F(2, 21)=1.43 | 0,261 | 0,914 |
| Dietary intake | 29 | Selenium (µg) | 3 months after discharge | 39.32 (35.14–67.98) (n=5) | 44.78 (37.19–55.74) (n=13) | NA | W=32.00; Δ_HL=-0.18; 95% IC [-18.95 ; 26.04] | 1,000 | 1,000 |
| Dietary intake | 30 | Selenium (µg) | 6 months after discharge | 49.96 ± 17.41 (n=4) | 46.43 ± 21.14 (n=12) | NA | t(14)=0.30; Δ_mean=3.53; 95% IC [-21.73 ; 28.79] | 0,769 | 0,914 |
| Dietary intake | 31 | Zinc (mg) | Preoperative | 17.57 (16.95–20.01) (n=7) | 16.79 (13.33–17.87) (n=14) | 19.34 (18.43–22.16) (n=3) | H(2)=2.36 | 0,307 | 0,914 |
| Dietary intake | 32 | Zinc (mg) | 3 months after discharge | 14.27 ± 2.15 (n=5) | 14.81 ± 5.36 (n=13) | NA | t(16)=-0.22; Δ_mean=-0.54; 95% IC [-5.85 ; 4.77] | 0,832 | 0,914 |
| Dietary intake | 33 | Zinc (mg) | 6 months after discharge | 14.12 ± 1.20 (n=4) | 13.69 ± 3.98 (n=12) | NA | t(14)=0.21; Δ_mean=0.43; 95% IC [-4.00 ; 4.85] | 0,839 | 0,914 |
| Dietary intake | 34 | Sodium (mg) | Preoperative | 1759.41 (1446.72–2561.47) (n=7) | 1656.51 (1293.47–2299.64) (n=14) | 1241.65 (1035.90–1975.62) (n=3) | H(2)=0.99 | 0,608 | 0,914 |
| Dietary intake | 35 | Sodium (mg) | 3 months after discharge | 1151.39 ± 331.27 (n=5) | 1450.99 ± 435.37 (n=13) | NA | t(16)=-1.38; Δ_mean=-299.60; 95% IC [-759.01 ; 159.81]0.933 | 0,186 | 0,914 |
| Dietary intake | 36 | Sodium (mg) | 6 months after discharge | 1043.45 ± 346.70 (n=4) | 1413.17 ± 428.28 (n=12) | NA | t(14)=-1.55; Δ_mean=-369.72; 95% IC [-880.09 ; 140.66]0.881 | 0,143 | 0,914 |
| Dietary intake | 37 | Vitamin A (mg) | Preoperative | 6.29 ± 2.86 (n=7) | 5.27 ± 2.91 (n=14) | 5.27 ± 3.22 (n=3) | F(2, 21)=0.30 | 0,741 | 0,914 |
| Dietary intake | 38 | Vitamin A (mg) | 3 months after discharge | 4.56 (3.92–7.34) (n=5) | 4.08 (2.89–6.93) (n=13) | NA | W=35.00; Δ_HL=0.13; 95% IC [-4.27 ; 3.45] | 0,844 | 0,914 |
| Dietary intake | 39 | Vitamin A (mg) | 6 months after discharge | 5.71 (4.13–7.17) (n=4) | 4.24 (2.84–5.23) (n=12) | NA | W=28.50; Δ_HL=0.86; 95% IC [-2.99 ; 4.11] | 0,627 | 0,914 |
| Dietary intake | 40 | Vitamin C (mg) | Preoperative | 419.76 ± 193.81 (n=7) | 375.72 ± 204.34 (n=14) | 335.13 ± 214.37 (n=3) | F(2, 21)=0.21 | 0,814 | 0,914 |
| Dietary intake | 41 | Vitamin C (mg) | 3 months after discharge | 273.36 (210.21–504.52) (n=5) | 337.47 (212.34–349.88) (n=13) | NA | W=30.00; Δ_HL=-12.44; 95% IC [-149.98 ; 275.81] | 0,844 | 0,914 |
| Dietary intake | 42 | Vitamin C (mg) | 6 months after discharge | 371.56 (256.24–494.24) (n=4) | 314.27 (211.08–353.83) (n=12) | NA | W=26.00; Δ_HL=45.28; 95% IC [-166.00 ; 279.23] | 0,856 | 0,914 |
| Dietary intake | 43 | Vitamin E (mg) | Preoperative | 5.17 ± 1.86 (n=7) | 4.38 ± 1.57 (n=14) | 4.06 ± 2.02 (n=3) | F(2, 21)=0.65 | 0,533 | 0,914 |
| Dietary intake | 44 | Vitamin E (mg) | 3 months after discharge | 4.67 ± 1.82 (n=5) | 4.52 ± 2.48 (n=13) | NA | t(16)=0.12; Δ_mean=0.15; 95% IC [-2.45 ; 2.76] | 0,903 | 0,924 |
| Dietary intake | 45 | Vitamin E (mg) | 6 months after discharge | 3.61 ± 2.08 (n=4) | 4.17 ± 2.15 (n=12) | NA | t(14)=-0.45; Δ_mean=-0.56; 95% IC [-3.21 ; 2.09] | 0,658 | 0,914 |
| Clinical–Nutritional Laboratory Analyses | 46 | Total Proteins (g/dL) | Preoperative | 7.20 ± 0.46 (n=7) | 6.71 ± 0.56 (n=14) | 7.14 ± 0.54 (n=3) | F(2, 21)=2.34 | 0,121 | 0,704 |
| Clinical–Nutritional Laboratory Analyses | 47 | Total Proteins (g/dL) | 3 months after discharge after discharge | 6.88 ± 0.33 (n=5) | 6.88 ± 0.53 (n=13) | NA | t(16)=0.01; Δ_mean=0.00; 95% IC [-0.54 ; 0.55] | 0,994 | 1,000 |
| Clinical–Nutritional Laboratory Analyses | 48 | Total Proteins (g/dL) | 6 months after discharge after discharge | 6.82 ± 0.56 (n=4) | 6.60 ± 0.50 (n=12) | NA | t(14)=0.73; Δ_mean=0.22; 95% IC [-0.42 ; 0.86] | 0,477 | 0,813 |
| Clinical–Nutritional Laboratory Analyses | 49 | Albumin (g/dL) | Preoperative | 4.45 ± 0.35 (n=7) | 3.72 ± 0.60 (n=14) | 3.85 ± 0.58 (n=3) | F(2, 21)=4.37 | 0,026 | 0,594 |
| Clinical–Nutritional Laboratory Analyses | 50 | Albumin (g/dL) | 3 months after discharge after discharge | 4.12 ± 0.55 (n=5) | 4.10 ± 0.53 (n=13) | NA | t(16)=0.06; Δ_mean=0.02; 95% IC [-0.58 ; 0.62] | 0,949 | 0,986 |
| Clinical–Nutritional Laboratory Analyses | 51 | Albumin (g/dL) | 6 months after discharge after discharge | 4.12 (3.80–4.42) (n=4) | 4.25 (4.09–4.72) (n=12) | NA | W=18.00; Δ_HL=-0.29; 95% IC [-0.87 ; 0.42] | 0,505 | 0,813 |
| Clinical–Nutritional Laboratory Analyses | 52 | C-Reactive Protein (mg/L) | Preoperative | 5.24 (1.10–14.28) (n=7) | 6.19 (3.17–10.31) (n=14) | 27.00 (15.88–30.17) (n=3) | H(2)=2.84 | 0,242 | 0,726 |
| Clinical–Nutritional Laboratory Analyses | 53 | C-Reactive Protein (mg/L) | 3 months after discharge after discharge | 0.40 (0.40–0.40) (n=5) | 1.76 (0.40–4.09) (n=13) | NA | W=21.00; Δ_HL=-0.36; 95% IC [-3.69 ; 0.49] | 0,257 | 0,726 |
| Clinical–Nutritional Laboratory Analyses | 54 | C-Reactive Protein (mg/L) | 6 months after discharge after discharge | 0.40 (0.40–0.40) (n=4) | 0.66 (0.40–2.24) (n=12) | NA | W=10.00; Δ_HL=-0.21; 95% IC [-3.53 ; 0.00] | 0,071 | 0,669 |
| Clinical–Nutritional Laboratory Analyses | 55 | Blood Glucose (mg/dL) | Preoperative | 117.00 (91.11–142.72) (n=7) | 116.54 (101.00–148.85) (n=14) | 168.00 (124.61–173.00) (n=3) | H(2)=0.70 | 0,706 | 0,847 |
| Clinical–Nutritional Laboratory Analyses | 56 | Blood Glucose (mg/dL) | 3 months after discharge after discharge | 128.23 (91.13–134.93) (n=5) | 118.00 (84.26–133.95) (n=13) | NA | W=41.00; Δ_HL=8.41; 95% IC [-36.79 ; 50.67] | 0,430 | 0,813 |
| Clinical–Nutritional Laboratory Analyses | 57 | Blood Glucose (mg/dL) | 6 months after discharge after discharge | 125.03 ± 54.65 (n=4) | 100.92 ± 22.38 (n=12) | NA | t(14)=1.30; Δ_mean=24.11; 95% IC [-15.70 ; 63.93] | 0,215 | 0,704 |
| Clinical–Nutritional Laboratory Analyses | 58 | Hemoglobin (g/dL) | Preoperative | 13.80 ± 2.79 (n=7) | 11.88 ± 1.87 (n=14) | 13.33 ± 1.71 (n=3) | F(2, 21)=2.03 | 0,156 | 0,704 |
| Clinical–Nutritional Laboratory Analyses | 59 | Hemoglobin (g/dL) | 3 months after discharge after discharge | 13.51 ± 0.70 (n=5) | 12.20 ± 1.73 (n=13) | NA | t(16)=1.62; Δ_mean=1.31; 95% IC [-0.41 ; 3.03] | 0,125 | 0,704 |
| Clinical–Nutritional Laboratory Analyses | 60 | Hemoglobin (g/dL) | 6 months after discharge after discharge | 12.75 ± 1.57 (n=4) | 12.11 ± 2.38 (n=12) | NA | t(14)=0.50; Δ_mean=0.64; 95% IC [-2.12 ; 3.40] | 0,627 | 0,813 |
| Clinical–Nutritional Laboratory Analyses | 61 | Lymphocytes (x10³/mm³) | Preoperative | 2.10 (1.80–3.20) (n=7) | 1.75 (1.12–3.10) (n=14) | 1.80 (1.80–2.25) (n=3) | H(2)=0.59 | 0,744 | 0,847 |
| Clinical–Nutritional Laboratory Analyses | 62 | Lymphocytes (x10³/mm³) | 3 months after discharge after discharge | 2.82 ± 0.58 (n=5) | 2.37 ± 1.03 (n=13) | NA | t(16)=0.92; Δ_mean=0.45; 95% IC [-0.59 ; 1.49] | 0,372 | 0,768 |
| Clinical–Nutritional Laboratory Analyses | 63 | Lymphocytes (x10³/mm³) | 6 months after discharge after discharge | 2.62 ± 0.85 (n=4) | 2.33 ± 1.11 (n=12) | NA | t(14)=0.47; Δ_mean=0.29; 95% IC [-1.02 ; 1.60] | 0,642 | 0,813 |
| Clinical–Nutritional Laboratory Analyses | 64 | Serum Iron (µg/dL) | Preoperative | 83.22 ± 31.75 (n=7) | 81.11 ± 38.90 (n=14) | 126.51 ± 35.69 (n=3) | F(2, 21)=1.96 | 0,166 | 0,704 |
| Clinical–Nutritional Laboratory Analyses | 65 | Serum Iron (µg/dL) | 3 months after discharge after discharge | 81.57 ± 28.27 (n=5) | 101.51 ± 62.52 (n=13) | NA | t(16)=-0.68; Δ_mean=-19.94; 95% IC [-82.37 ; 42.48] | 0,508 | 0,813 |
| Clinical–Nutritional Laboratory Analyses | 66 | Serum Iron (µg/dL) | 6 months after discharge after discharge | 95.47 ± 7.00 (n=4) | 86.35 ± 49.02 (n=12) | NA | t(14)=0.36; Δ_mean=9.12; 95% IC [-44.83 ; 63.07] | 0,722 | 0,847 |
| Clinical–Nutritional Laboratory Analyses | 67 | Ferritin (ng/mL) | Preoperative | 250.10 (153.45–399.25) (n=7) | 740.45 (123.50–1222.33) (n=14) | 209.10 (144.60–369.25) (n=3) | H(2)=1.46 | 0,482 | 0,813 |
| Clinical–Nutritional Laboratory Analyses | 68 | Ferritin (ng/mL) | 3 months after discharge after discharge | 254.60 (45.10–399.60) (n=5) | 234.30 (183.60–477.80) (n=13) | NA | W=25.00; Δ_HL=-128.00; 95% IC [-432.70 ; 215.70] | 0,490 | 0,813 |
| Clinical–Nutritional Laboratory Analyses | 69 | Ferritin (ng/mL) | 6 months after discharge after discharge | 191.91 ± 153.02 (n=4) | 295.61 ± 207.21 (n=12) | NA | t(14)=-0.91; Δ_mean=-103.70; 95% IC [-347.47 ; 140.06] | 0,377 | 0,768 |
| Clinical–Nutritional Laboratory Analyses | 70 | UIBC (µg/dL) | Preoperative | 293.93 ± 37.40 (n=7) | 277.78 ± 77.14 (n=14) | 319.41 ± 111.34 (n=3) | F(2, 21)=0.44 | 0,648 | 0,813 |
| Clinical–Nutritional Laboratory Analyses | 71 | UIBC (µg/dL) | 3 months after discharge after discharge | 323.65 ± 43.37 (n=5) | 288.95 ± 54.79 (n=13) | NA | t(16)=1.26; Δ_mean=34.70; 95% IC [-23.51 ; 92.90] | 0,224 | 0,704 |
| Clinical–Nutritional Laboratory Analyses | 72 | UIBC (µg/dL) | 6 months after discharge after discharge | 309.48 ± 22.95 (n=4) | 298.62 ± 44.28 (n=12) | NA | t(14)=0.46; Δ_mean=10.86; 95% IC [-39.49 ; 61.22] | 0,651 | 0,813 |
| Clinical–Nutritional Laboratory Analyses | 73 | TIBC (µg/dL) | Preoperative | 377.15 ± 60.42 (n=7) | 358.89 ± 89.29 (n=14) | 445.92 ± 107.51 (n=3) | F(2, 21)=1.32 | 0,287 | 0,758 |
| Clinical–Nutritional Laboratory Analyses | 74 | TIBC (µg/dL) | 3 months after discharge after discharge | 405.21 ± 41.63 (n=5) | 390.46 ± 106.81 (n=13) | NA | t(16)=0.30; Δ_mean=14.75; 95% IC [-91.02 ; 120.52] | 0,771 | 0,848 |
| Clinical–Nutritional Laboratory Analyses | 75 | TIBC (µg/dL) | 6 months after discharge after discharge | 404.95 ± 18.19 (n=4) | 384.97 ± 70.27 (n=12) | NA | t(13.82)=0.90; Δ_mean=19.98; 95% IC [-27.76 ; 67.72] | 0,384 | 0,768 |
| Clinical–Nutritional Laboratory Analyses | 76 | Transferrin (mg/dL) | Preoperative | 235.14 ± 29.92 (n=7) | 222.22 ± 61.71 (n=14) | 255.53 ± 89.07 (n=3) | F(2, 21)=0.44 | 0,648 | 0,813 |
| Clinical–Nutritional Laboratory Analyses | 77 | Transferrin (mg/dL) | 3 months after discharge after discharge | 258.92 ± 34.70 (n=5) | 231.16 ± 43.83 (n=13) | NA | t(16)=1.26; Δ_mean=27.76; 95% IC [-18.80 ; 74.32] | 0,224 | 0,704 |
| Clinical–Nutritional Laboratory Analyses | 78 | Transferrin (mg/dL) | 6 months after discharge after discharge | 247.59 ± 18.36 (n=4) | 238.90 ± 35.43 (n=12) | NA | t(14)=0.46; Δ_mean=8.69; 95% IC [-31.59 ; 48.98] | 0,651 | 0,813 |
| Clinical–Nutritional Laboratory Analyses | 79 | Transferrin Saturation (%) | Preoperative | 21.59 ± 6.03 (n=7) | 22.45 ± 8.65 (n=14) | 29.40 ± 9.29 (n=3) | F(2, 21)=1.09 | 0,356 | 0,768 |
| Clinical–Nutritional Laboratory Analyses | 80 | Transferrin Saturation (%) | 3 months after discharge after discharge | 20.10 ± 6.48 (n=5) | 24.00 ± 9.95 (n=13) | NA | t(16)=-0.80; Δ_mean=-3.90; 95% IC [-14.17 ; 6.37] | 0,433 | 0,813 |
| Clinical–Nutritional Laboratory Analyses | 81 | Transferrin Saturation (%) | 6 months after discharge after discharge | 23.64 ± 2.44 (n=4) | 21.42 ± 9.40 (n=12) | NA | t(14)=0.46; Δ_mean=2.22; 95% IC [-8.20 ; 12.64] | 0,655 | 0,813 |
| Clinical–Nutritional Laboratory Analyses | 82 | Urea (mg/dL) | Preoperative | 35.61 (33.58–42.79) (n=7) | 28.88 (22.32–39.49) (n=14) | 34.56 (30.26–55.28) (n=3) | H(2)=0.89 | 0,640 | 0,813 |
| Clinical–Nutritional Laboratory Analyses | 83 | Urea (mg/dL) | 3 months after discharge after discharge | 35.59 (35.01–36.42) (n=5) | 32.46 (26.43–51.96) (n=13) | NA | W=32.00; Δ_HL=-8.82; 95% IC [-29.90 ; 11.09] | 1,000 | 1,000 |
| Clinical–Nutritional Laboratory Analyses | 84 | Urea (mg/dL) | 6 months after discharge after discharge | 20.96 (18.24–25.04) (n=4) | 33.70 (25.40–45.01) (n=12) | NA | W=7.00; Δ_HL=-12.33; 95% IC [-34.62 ; -0.47] | 0,045 | 0,594 |
| Clinical–Nutritional Laboratory Analyses | 85 | Creatinine (mg/dL) | Preoperative | 0.97 (0.82–1.07) (n=7) | 0.86 (0.82–1.21) (n=14) | 0.94 (0.82–1.45) (n=3) | H(2)=0.09 | 0,956 | 0,986 |
| Clinical–Nutritional Laboratory Analyses | 86 | Creatinine (mg/dL) | 3 months after discharge after discharge | 0.77 (0.73–0.87) (n=5) | 0.87 (0.75–0.96) (n=13) | NA | W=23.00; Δ_HL=-0.06; 95% IC [-0.28 ; 0.13] | 0,375 | 0,768 |
| Clinical–Nutritional Laboratory Analyses | 87 | Creatinine (mg/dL) | 6 months after discharge after discharge | 0.86 (0.83–0.89) (n=4) | 0.92 (0.78–1.11) (n=12) | NA | W=18.50; Δ_HL=-0.10; 95% IC [-1.14 ; 0.14] | 0,543 | 0,813 |
| Clinical–Nutritional Laboratory Analyses | 88 | Aspartate Aminotransferase (AST) (U/L) | Preoperative | 81.67 ± 60.48 (n=7) | 177.75 ± 105.19 (n=14) | 368.63 ± 246.51 (n=3) | F(2, 21)=6.34 | 0,007 | 0,462 |
| Clinical–Nutritional Laboratory Analyses | 89 | Aspartate Aminotransferase (AST) (U/L) | 3 months after discharge after discharge | 36.91 (29.79–36.98) (n=5) | 25.69 (22.31–35.43) (n=13) | NA | W=48.00; Δ_HL=9.44; 95% IC [-6.01 ; 14.67] | 0,139 | 0,704 |
| Clinical–Nutritional Laboratory Analyses | 90 | Aspartate Aminotransferase (AST) (U/L) | 6 months after discharge after discharge | 40.43 ± 18.90 (n=4) | 31.61 ± 8.46 (n=12) | NA | t(14)=1.33; Δ_mean=8.83; 95% IC [-5.44 ; 23.09] | 0,206 | 0,704 |
| Clinical–Nutritional Laboratory Analyses | 91 | Aminotransferase (ALT) (U/L) | Preoperative | 120.63 (48.27–252.91) (n=7) | 146.68 (81.45–204.49) (n=14) | 603.00 (344.00–696.71) (n=3) | H(2)=2.28 | 0,319 | 0,768 |
| Clinical–Nutritional Laboratory Analyses | 92 | Aminotransferase (ALT) (U/L) | 3 months after discharge after discharge | 45.67 ± 11.22 (n=5) | 31.88 ± 15.07 (n=13) | NA | t(16)=1.84; Δ_mean=13.78; 95% IC [-2.06 ; 29.63] | 0,084 | 0,693 |
| Clinical–Nutritional Laboratory Analyses | 93 | Aminotransferase (ALT) (U/L) | 6 months after discharge after discharge | 39.30 (29.00–48.99) (n=4) | 34.82 (25.84–40.15) (n=12) | NA | W=27.00; Δ_HL=4.83; 95% IC [-17.50 ; 20.79] | 0,762 | 0,848 |
| Clinical–Nutritional Laboratory Analyses | 94 | Folic Acid (ng/mL) | Preoperative | 16.63 ± 3.34 (n=7) | 13.45 ± 6.77 (n=14) | 14.82 ± 7.96 (n=3) | F(2, 21)=0.63 | 0,541 | 0,813 |
| Clinical–Nutritional Laboratory Analyses | 95 | Folic Acid (ng/mL) | 3 months after discharge after discharge | 24.00 (22.61–24.00) (n=5) | 14.74 (10.62–24.00) (n=13) | NA | W=44.00; Δ_HL=5.27; 95% IC [-1.39 ; 13.38] | 0,264 | 0,726 |
| Clinical–Nutritional Laboratory Analyses | 96 | Folic Acid (ng/mL) | 6 months after discharge after discharge | 21.17 (18.57–22.10) (n=4) | 22.47 (13.54–24.00) (n=12) | NA | W=23.00; Δ_HL=-0.00; 95% IC [-6.22 ; 10.88] | 0,951 | 0,986 |
| Clinical–Nutritional Laboratory Analyses | 97 | Vitamin B12 (pg/mL) | Preoperative | 410.00 (321.50–435.00) (n=7) | 656.50 (455.00–866.25) (n=14) | 589.00 (522.00–607.00) (n=3) | H(2)=6.74 | 0,034 | 0,594 |
| Clinical–Nutritional Laboratory Analyses | 98 | Vitamin B12 (pg/mL) | 3 months after discharge after discharge | 372.00 (333.00–487.00) (n=5) | 538.00 (416.00–1047.00) (n=13) | NA | W=17.00; Δ_HL=-162.00; 95% IC [-734.00 ; 71.00] | 0,139 | 0,704 |
| Clinical–Nutritional Laboratory Analyses | 99 | Vitamin B12 (pg/mL) | 6 months after discharge after discharge | 314.62 (313.00–397.44) (n=4) | 451.47 (338.76–544.50) (n=12) | NA | W=16.00; Δ_HL=-66.00; 95% IC [-368.73 ; 126.00] | 0,363 | 0,768 |
| Clinical–Nutritional Laboratory Analyses | 100 | Vitamin A (µg/dL) | Preoperative | 0.37 (0.26–0.37) (n=7) | 0.30 (0.17–0.34) (n=14) | 0.30 (0.24–0.30) (n=3) | H(2)=2.31 | 0,316 | 0,768 |
| Clinical–Nutritional Laboratory Analyses | 101 | Vitamin A (µg/dL) | 3 months after discharge after discharge | 0.34 ± 0.10 (n=5) | 0.32 ± 0.11 (n=13) | NA | t(16)=0.44; Δ_mean=0.03; 95% IC [-0.10 ; 0.15] | 0,665 | 0,813 |
| Clinical–Nutritional Laboratory Analyses | 102 | Vitamin A (µg/dL) | 6 months after discharge after discharge | 0.27 (0.25–0.30) (n=4) | 0.33 (0.28–0.38) (n=12) | NA | W=12.50; Δ_HL=-0.06; 95% IC [-0.20 ; 0.02] | 0,180 | 0,704 |
| Clinical–Nutritional Laboratory Analyses | 103 | Vitamin E (mg/dL) | Preoperative | 7.46 (6.06–8.34) (n=7) | 8.11 (6.30–9.63) (n=14) | 7.28 (7.28–7.28) (n=3) | H(2)=0.90 | 0,638 | 0,813 |
| Clinical–Nutritional Laboratory Analyses | 104 | Vitamin E (mg/dL) | 3 months after discharge after discharge | 7.13 ± 2.42 (n=5) | 6.46 ± 2.21 (n=13) | NA | t(16)=0.56; Δ_mean=0.67; 95% IC [-1.86 ; 3.20] | 0,583 | 0,813 |
| Clinical–Nutritional Laboratory Analyses | 105 | Vitamin E (mg/dL) | 6 months after discharge after discharge | 5.26 (4.99–5.53) (n=4) | 5.82 (4.69–7.29) (n=12) | NA | W=19.00; Δ_HL=-0.56; 95% IC [-4.57 ; 1.33] | 0,585 | 0,813 |
| Clinical–Nutritional Laboratory Analyses | 106 | Copper (µg/dL) | Preoperative | 137.00 (94.80–151.40) (n=7) | 142.50 (115.15–154.55) (n=14) | 137.00 (117.00–209.80) (n=3) | H(2)=0.61 | 0,739 | 0,847 |
| Clinical–Nutritional Laboratory Analyses | 107 | Copper (µg/dL) | 3 months after discharge after discharge | 80.76 ± 18.30 (n=5) | 115.63 ± 32.63 (n=13) | NA | t(16)=-2.23; Δ_mean=-34.87; 95% IC [-68.01 ; -1.73] | 0,040 | 0,594 |
| Clinical–Nutritional Laboratory Analyses | 108 | Copper (µg/dL) | 6 months after discharge after discharge | 78.17 ± 15.95 (n=4) | 109.28 ± 29.09 (n=12) | NA | t(14)=-2.01; Δ_mean=-31.11; 95% IC [-64.32 ; 2.09] | 0,064 | 0,669 |
| Clinical–Nutritional Laboratory Analyses | 109 | Zinc (µg/dL) | Preoperative | 85.00 (67.75–98.25) (n=7) | 90.00 (60.75–95.25) (n=14) | 79.50 (66.75–87.75) (n=3) | H(2)=0.33 | 0,849 | 0,919 |
| Clinical–Nutritional Laboratory Analyses | 110 | Zinc (µg/dL) | 3 months after discharge after discharge | 96.70 ± 37.15 (n=5) | 81.88 ± 9.66 (n=13) | NA | t(16)=1.38; Δ_mean=14.82; 95% IC [-7.91 ; 37.54] | 0,186 | 0,704 |
| Clinical–Nutritional Laboratory Analyses | 111 | Zinc (µg/dL) | 6 months after discharge after discharge | 68.36 ± 19.44 (n=4) | 81.40 ± 11.60 (n=12) | NA | t(14)=-1.65; Δ_mean=-13.05; 95% IC [-29.97 ; 3.88] | 0,120 | 0,704 |
| Derived inflammatory–nutritional indices | 112 | Prognostic Nutritional Index | Preoperative | 56.26 ± 7.12 (n=7) | 48.26 ± 10.74 (n=14) | 49.00 ± 3.91 (n=3) | F(2, 21)=1.77 | 0,195 | 0,558 |
| Derived inflammatory–nutritional indices | 113 | Prognostic Nutritional Index | 3 months after discharge | 56.80 (56.20–59.20) (n=5) | 51.30 (49.20–57.90) (n=13) | N/A | W=39.00; Δ_HL=1.50; 95% IC [-6.50 ; 10.10] | 0,554 | 0,665 |
| Derived inflammatory–nutritional indices | 114 | Prognostic Nutritional Index | 6 months after discharge | 54.45 (52.30–56.33) (n=4) | 58.13 (48.70–61.17) (n=12) | N/A | W=21.00; Δ_HL=-2.11; 95% IC [-9.20 ; 9.60] | 0,762 | 0,795 |
| Derived inflammatory–nutritional indices | 115 | Glasgow Prognostic Score (GPS) | Preoperative | 0: 4 (57.1%); 1: 3 (42.9%) | 0: 6 (42.9%); 1: 6 (42.9%); 2: 2 (14.3%) | 0: 1 (33.3%); 1: 1 (33.3%); 2: 1 (33.3%) | H(2)=1.25 | 0,535 | 0,665 |
| Derived inflammatory–nutritional indices | 116 | Glasgow Prognostic Score (GPS) | 3 months after discharge | 0: 4 (80.0%); 1: 1 (20.0%) | 0: 11 (84.6%); 1: 2 (15.4%) | N/A | W=34.00; Δ_HL=0.00; 95% IC [-0.00 ; 0.00] | 0,879 | 0,879 |
| Derived inflammatory–nutritional indices | 117 | Glasgow Prognostic Score (GPS) | 6 months after discharge | 0: 4 (100.0%) | 0: 11 (91.7%); 2: 1 (8.3%) | N/A | W=22.00; Δ_HL=0.00; 95% IC [-0.00 ; 0.00] | 0,665 | 0,725 |
| Derived inflammatory–nutritional indices | 118 | Albumin/CRP ratio | Preoperative | 0.86 (0.32–6.71) (n=7) | 0.64 (0.29–1.34) (n=14) | 0.13 (0.13–0.47) (n=3) | H(2)=3.89 | 0,143 | 0,558 |
| Derived inflammatory–nutritional indices | 119 | Albumin/CRP ratio | 3 months after discharge | 10.70 (10.45–10.80) (n=5) | 2.26 (0.83–10.65) (n=13) | N/A | W=42.50; Δ_HL=5.17; 95% IC [-0.53 ; 9.87] | 0,349 | 0,558 |
| Derived inflammatory–nutritional indices | 120 | Albumin/CRP ratio | 6 months after discharge | 10.26 ± 1.05 (n=4) | 6.28 ± 4.63 (n=12) | N/A | t(13.49)=2.77; Δ_mean=3.99; 95% IC [0.89 ; 7.08] | 0,015 | 0,360 |
| Derived inflammatory–nutritional indices | 121 | CRP/Albumin ratio | Preoperative | 1.17 (0.23–3.29) (n=7) | 1.66 (0.79–3.47) (n=14) | 7.49 (4.37–7.84) (n=3) | H(2)=3.76 | 0,153 | 0,558 |
| Derived inflammatory–nutritional indices | 122 | CRP/Albumin ratio | 3 months after discharge | 925.93 (865.80–934.58) (n=5) | 1.24 (0.49–836.82) (n=13) | N/A | W=54.50; Δ_HL=864.59; 95% IC [0.46 ; 934.09] | 0,034 | 0,360 |
| Derived inflammatory–nutritional indices | 123 | CRP/Albumin ratio | 6 months after discharge | 441.56 (0.11–890.04) (n=4) | 3.49 (0.41–869.24) (n=12) | N/A | W=18.00; Δ_HL=-0.29; 95% IC [-847.35 ; 882.85] | 0,505 | 0,665 |
| Derived inflammatory–nutritional indices | 124 | CRP/Lymphocytes ratio | Preoperative | 2.91 (0.34–7.62) (n=7) | 2.47 (1.29–7.23) (n=14) | 10.00 (6.32–14.26) (n=3) | H(2)=2.52 | 0,283 | 0,558 |
| Derived inflammatory–nutritional indices | 125 | CRP/Lymphocytes ratio | 3 months after discharge | 0.14 (0.12–0.20) (n=5) | 1.33 (0.29–2.40) (n=13) | N/A | W=12.50; Δ_HL=-0.58; 95% IC [-2.60 ; 0.00] | 0,054 | 0,360 |
| Derived inflammatory–nutritional indices | 126 | CRP/Lymphocytes ratio | 6 months after discharge | 0.17 (0.14–0.20) (n=4) | 0.45 (0.26–2.24) (n=12) | N/A | W=8.00; Δ_HL=-0.29; 95% IC [-10.78 ; 0.02] | 0,060 | 0,360 |
| Derived inflammatory–nutritional indices | 127 | Ferritin/Albumin ratio | Preoperative | 54.25 (34.03–84.84) (n=7) | 182.11 (37.22–299.03) (n=14) | 46.99 (35.63–93.15) (n=3) | H(2)=2.18 | 0,336 | 0,558 |
| Derived inflammatory–nutritional indices | 128 | Ferritin/Albumin ratio | 3 months after discharge | 58.94 (9.76–125.66) (n=5) | 59.04 (41.82–111.64) (n=13) | N/A | W=27.00; Δ_HL=-32.77; 95% IC [-101.87 ; 73.57] | 0,622 | 0,711 |
| Derived inflammatory–nutritional indices | 129 | Ferritin/Albumin ratio | 6 months after discharge | 39.49 (23.63–65.55) (n=4) | 55.01 (36.68–106.73) (n=12) | N/A | W=18.00; Δ_HL=-11.52; 95% IC [-83.88 ; 43.83] | 0,505 | 0,665 |
| Derived inflammatory–nutritional indices | 130 | Ferritin/Lymphocytes ratio | Preoperative | 84.09 (72.62–149.68) (n=7) | 398.84 (121.87–536.95) (n=14) | 116.17 (72.92–205.14) (n=3) | H(2)=2.30 | 0,317 | 0,558 |
| Derived inflammatory–nutritional indices | 131 | Ferritin/Lymphocytes ratio | 3 months after discharge | 79.56 (22.55–153.69) (n=5) | 103.87 (76.62–330.16) (n=13) | N/A | W=24.00; Δ_HL=-54.96; 95% IC [-323.12 ; 54.50] | 0,430 | 0,645 |
| Derived inflammatory–nutritional indices | 132 | Ferritin/Lymphocytes ratio | 6 months after discharge | 70.20 (41.19–96.16) (n=4) | 117.51 (62.46–203.21) (n=12) | N/A | W=14.00; Δ_HL=-41.86; 95% IC [-263.78 ; 31.70] | 0,249 | 0,558 |
| Derived inflammatory–nutritional indices | 133 | Albumin-Lymphocytes-CRP index | Preoperative | 1.54 (0.61–21.60) (n=7) | 1.36 (0.47–3.11) (n=14) | 0.33 (0.29–0.89) (n=3) | H(2)=2.38 | 0,304 | 0,558 |
| Derived inflammatory–nutritional indices | 134 | Albumin-Lymphocytes-CRP index | 3 months after discharge | 29.96 (23.10–34.56) (n=5) | 5.20 (1.65–19.14) (n=13) | N/A | W=46.00; Δ_HL=17.90; 95% IC [-3.80 ; 31.36] | 0,200 | 0,558 |
| Derived inflammatory–nutritional indices | 135 | Albumin-Lymphocytes-CRP index | 6 months after discharge | 26.58 ± 7.19 (n=4) | 16.46 ± 15.86 (n=12) | N/A | t(14)=1.21; Δ_mean=10.11; 95% IC [-7.77 ; 28.00] | 0,245 | 0,558 |
| Notes: Values are presented as mean +/- standard deviation or median (P25-P75), with the valid n shown for each group and comparison. The analysis used observed available data only; no multiple imputation was performed. The number of patients evaluated was preoperative n = 24, 3 months after discharge n = 18, and 6 months after discharge n = 16. Raw p-values correspond to the statistical test reported in each row. BH-FDR adjusted p-values were calculated with the Benjamini-Hochberg false discovery rate procedure within each Exposure x Domain family: Dietary intake (45 tests), Clinical-nutritional laboratory analyses (66 tests), and Derived inflammatory-nutritional indices (24 tests). The BH-FDR audit found 945 inferential comparisons, 43 raw p-values < 0.05, and 0 BH-FDR adjusted p-values < 0.05. For binary exposures, two-sample t-test/Welch t-test or Mann-Whitney/Wilcoxon rank-sum tests were used as reported. For multicategorical or ordinal exposures, one-way ANOVA or Kruskal-Wallis tests were used; these are omnibus tests and do not establish pairwise differences or dose-response trends without post-hoc or contrast testing. Because this was an exploratory supplementary analysis with multiple comparisons, small subgroup sizes, and no covariate adjustment, the results should be interpreted as hypothesis-generating rather than confirmatory. Clinically relevant POPF was defined as ISGPS grade B or C. Abbreviations: BH-FDR, Benjamini-Hochberg false discovery rate; POPF, postoperative pancreatic fistula; ISGPS, International Study Group of Pancreatic Surgery; PERT, pancreatic enzyme replacement therapy; POPF, postoperative pancreatic fistula; TIBC, total iron-binding capacity; UIBC, unsaturated iron-binding capacity. | | | | | | | | | |

| **Supplementary Table S9.** Exploratory associations between exocrine pancreatic insufficiency (EPI) and dietary intake, clinical-nutritional laboratory variables, and inflammatory-nutritional indices after pancreatoduodenectomy. | | | | | | | | |
| --- | --- | --- | --- | --- | --- | --- | --- | --- |
| **Outcome** | | | | **Exocrine pancreatic insufficiency** | | | | |
| **Domain** | **Source row** | **Variable** | **Time** | **Yes** | **No** | **Statistical result** | **Raw p-value** | **BH-FDR adjusted p-value** |
|  |  |  |  | **Mean ± SD or Median (P25–P75), n** | **Mean ± SD or Median (P25–P75), n** |  |  |  |
| Dietary intake | 1 | Energy (kcal) | Preoperative | 2642.86 ± 560.27 (n=19) | 3188.56 ± 460.89 (n=4) | t(21)=-1.81; Δ_mean=-545.69; 95% IC [-1171.68 ; 80.30] | 0,084 | 0,483 |
| Dietary intake | 2 | Energy (kcal) | 3 months after discharge | 2029.30 (1850.60–2323.24) (n=17) | 1955.01 (1955.01–1955.01) (n=1) | W=10.00; Δ_HL=74.29; 95% IC [-558.88 ; 711.69] | 0,847 | 0,908 |
| Dietary intake | 3 | Energy (kcal) | 6 months after discharge | 1954.42 (1867.03–2144.14) (n=15) | 1656.00 (1656.00–1656.00) (n=1) | W=14.00; Δ_HL=298.42; 95% IC [-278.05 ; 955.55] | 0,193 | 0,483 |
| Dietary intake | 4 | Protein (g) | Preoperative | 116.34 ± 24.16 (n=19) | 127.97 ± 14.98 (n=4) | t(21)=-0.92; Δ_mean=-11.63; 95% IC [-38.02 ; 14.76] | 0,370 | 0,666 |
| Dietary intake | 5 | Protein (g) | 3 months after discharge | 95.89 (88.50–106.84) (n=17) | 81.37 (81.37–81.37) (n=1) | W=16.00; Δ_HL=14.52; 95% IC [-12.52 ; 45.85] | 0,177 | 0,483 |
| Dietary intake | 6 | Protein (g) | 6 months after discharge | 96.86 (91.59–106.60) (n=15) | 107.49 (107.49–107.49) (n=1) | W=4.00; Δ_HL=-10.63; 95% IC [-35.05 ; 27.96] | 0,515 | 0,799 |
| Dietary intake | 7 | Relative energy balance (kcal) | Preoperative | 1065.74 ± 326.49 (n=19) | 1380.06 ± 171.91 (n=4) | t(21)=-1.85; Δ_mean=-314.32; 95% IC [-668.03 ; 39.39] | 0,079 | 0,483 |
| Dietary intake | 8 | Relative energy balance (kcal) | 3 months after discharge | 514.92 (384.06–693.70) (n=17) | 317.08 (317.08–317.08) (n=1) | W=16.00; Δ_HL=197.84; 95% IC [-140.29 ; 741.50] | 0,177 | 0,483 |
| Dietary intake | 9 | Relative energy balance (kcal) | 6 months after discharge | 614.48 (515.53–686.46) (n=15) | 578.33 (578.33–578.33) (n=1) | W=10.00; Δ_HL=36.15; 95% IC [-237.61 ; 590.76] | 0,664 | 0,908 |
| Dietary intake | 10 | Carbohydrate (g) | Preoperative | 366.77 ± 78.80 (n=19) | 437.36 ± 55.30 (n=4) | t(21)=-1.69; Δ_mean=-70.59; 95% IC [-157.40 ; 16.23] | 0,106 | 0,483 |
| Dietary intake | 11 | Carbohydrate (g) | 3 months after discharge | 266.21 (237.78–281.31) (n=17) | 261.97 (261.97–261.97) (n=1) | W=10.00; Δ_HL=4.24; 95% IC [-76.54 ; 76.29] | 0,847 | 0,908 |
| Dietary intake | 12 | Carbohydrate (g) | 6 months after discharge | 268.49 (242.18–279.41) (n=15) | 223.71 (223.71–223.71) (n=1) | W=14.00; Δ_HL=44.78; 95% IC [-38.63 ; 131.07] | 0,193 | 0,483 |
| Dietary intake | 13 | Lipid (g) | Preoperative | 78.94 ± 22.87 (n=19) | 103.03 ± 23.43 (n=4) | t(21)=-1.91; Δ_mean=-24.09; 95% IC [-50.34 ; 2.16] | 0,070 | 0,483 |
| Dietary intake | 14 | Lipid (g) | 3 months after discharge | 63.29 (56.65–77.74) (n=17) | 64.63 (64.63–64.63) (n=1) | W=8.00; Δ_HL=-1.34; 95% IC [-22.52 ; 33.38] | 1,000 | 1,000 |
| Dietary intake | 15 | Lipid (g) | 6 months after discharge | 58.62 (53.03–77.62) (n=15) | 36.80 (36.80–36.80) (n=1) | W=15.00; Δ_HL=21.82; 95% IC [1.85 ; 57.10] | 0,129 | 0,483 |
| Dietary intake | 16 | Fiber (g) | Preoperative | 44.62 ± 11.40 (n=19) | 55.32 ± 6.03 (n=4) | t(21)=-1.80; Δ_mean=-10.70; 95% IC [-23.05 ; 1.65] | 0,086 | 0,483 |
| Dietary intake | 17 | Fiber (g) | 3 months after discharge | 37.11 (34.74–46.11) (n=17) | 31.76 (31.76–31.76) (n=1) | W=14.00; Δ_HL=5.35; 95% IC [-11.74 ; 24.56] | 0,335 | 0,628 |
| Dietary intake | 18 | Fiber (g) | 6 months after discharge | 37.22 (34.92–43.12) (n=15) | 26.98 (26.98–26.98) (n=1) | W=13.00; Δ_HL=10.24; 95% IC [-6.07 ; 27.00] | 0,278 | 0,544 |
| Dietary intake | 19 | Calcium (mg) | Preoperative | 1461.96 ± 327.64 (n=19) | 1526.39 ± 311.31 (n=4) | t(21)=-0.36; Δ_mean=-64.43; 95% IC [-436.65 ; 307.80] | 0,722 | 0,908 |
| Dietary intake | 20 | Calcium (mg) | 3 months after discharge | 1274.51 (1019.91–1475.83) (n=17) | 1093.02 (1093.02–1093.02) (n=1) | W=10.00; Δ_HL=181.49; 95% IC [-283.12 ; 1128.95] | 0,847 | 0,908 |
| Dietary intake | 21 | Calcium (mg) | 6 months after discharge | 1258.11 (1020.80–1457.99) (n=15) | 926.12 (926.12–926.12) (n=1) | W=14.00; Δ_HL=331.99; 95% IC [-129.35 ; 1207.77] | 0,193 | 0,483 |
| Dietary intake | 22 | Iron (mg) | Preoperative | 26.10 ± 5.94 (n=19) | 33.98 ± 5.56 (n=4) | t(21)=-2.43; Δ_mean=-7.88; 95% IC [-14.62 ; -1.15] | 0,024 | 0,483 |
| Dietary intake | 23 | Iron (mg) | 3 months after discharge | 21.33 (17.75–27.88) (n=17) | 22.11 (22.11–22.11) (n=1) | W=8.00; Δ_HL=-0.78; 95% IC [-6.98 ; 26.48] | 1,000 | 1,000 |
| Dietary intake | 24 | Iron (mg) | 6 months after discharge | 21.33 (18.05–25.80) (n=15) | 18.72 (18.72–18.72) (n=1) | W=11.00; Δ_HL=2.61; 95% IC [-3.79 ; 31.95] | 0,515 | 0,799 |
| Dietary intake | 25 | Potassium (mg) | Preoperative | 6551.32 ± 1263.44 (n=19) | 7775.76 ± 1100.90 (n=4) | t(21)=-1.79; Δ_mean=-1224.44; 95% IC [-2644.78 ; 195.91] | 0,087 | 0,483 |
| Dietary intake | 26 | Potassium (mg) | 3 months after discharge | 5444.64 (4743.40–6898.60) (n=17) | 5039.31 (5039.31–5039.31) (n=1) | W=11.00; Δ_HL=405.33; 95% IC [-949.37 ; 3017.26] | 0,700 | 0,908 |
| Dietary intake | 27 | Potassium (mg) | 6 months after discharge | 5350.54 (4731.80–6498.32) (n=15) | 4270.76 (4270.76–4270.76) (n=1) | W=14.00; Δ_HL=1079.78; 95% IC [-234.07 ; 3454.32] | 0,193 | 0,483 |
| Dietary intake | 28 | Selenium (µg) | Preoperative | 57.22 ± 17.55 (n=19) | 60.78 ± 31.69 (n=4) | t(21)=-0.32; Δ_mean=-3.56; 95% IC [-26.66 ; 19.53] | 0,751 | 0,908 |
| Dietary intake | 29 | Selenium (µg) | 3 months after discharge | 44.78 (37.19–64.08) (n=17) | 35.14 (35.14–35.14) (n=1) | W=13.00; Δ_HL=9.64; 95% IC [-21.86 ; 52.71] | 0,441 | 0,763 |
| Dietary intake | 30 | Selenium (µg) | 6 months after discharge | 46.44 (38.41–61.93) (n=15) | 29.84 (29.84–29.84) (n=1) | W=13.00; Δ_HL=16.60; 95% IC [-14.36 ; 54.52] | 0,278 | 0,544 |
| Dietary intake | 31 | Zinc (mg) | Preoperative | 17.22 (15.21–18.12) (n=19) | 22.16 (18.88–26.06) (n=4) | W=15.00; Δ_HL=-6.49; 95% IC [-12.12 ; 0.05] | 0,068 | 0,483 |
| Dietary intake | 32 | Zinc (mg) | 3 months after discharge | 13.36 (11.11–16.79) (n=17) | 14.76 (14.76–14.76) (n=1) | W=7.00; Δ_HL=-1.40; 95% IC [-5.96 ; 11.66] | 0,847 | 0,908 |
| Dietary intake | 33 | Zinc (mg) | 6 months after discharge | 13.22 (11.39–15.64) (n=15) | 12.51 (12.51–12.51) (n=1) | W=9.00; Δ_HL=0.71; 95% IC [-3.37 ; 7.93] | 0,828 | 0,908 |
| Dietary intake | 34 | Sodium (mg) | Preoperative | 1637.82 (1310.32–2200.51) (n=19) | 1816.14 (1138.78–2470.38) (n=4) | W=42.00; Δ_HL=108.69; 95% IC [-1114.01 ; 1146.76] | 0,776 | 0,908 |
| Dietary intake | 35 | Sodium (mg) | 3 months after discharge | 1287.54 (1109.13–1668.49) (n=17) | 1189.32 (1189.32–1189.32) (n=1) | W=11.00; Δ_HL=98.22; 95% IC [-483.54 ; 1192.76] | 0,700 | 0,908 |
| Dietary intake | 36 | Sodium (mg) | 6 months after discharge | 1169.09 (1034.21–1600.03) (n=15) | 1008.44 (1008.44–1008.44) (n=1) | W=11.00; Δ_HL=160.65; 95% IC [-272.30 ; 1274.14] | 0,515 | 0,799 |
| Dietary intake | 37 | Vitamin A (mg) | Preoperative | 4.64 (3.59–7.58) (n=19) | 7.04 (4.98–8.35) (n=4) | W=33.00; Δ_HL=-1.37; 95% IC [-4.69 ; 2.94] | 0,715 | 0,908 |
| Dietary intake | 38 | Vitamin A (mg) | 3 months after discharge | 4.56 (3.61–7.34) (n=17) | 2.66 (2.66–2.66) (n=1) | W=15.00; Δ_HL=1.90; 95% IC [-0.13 ; 10.63] | 0,247 | 0,529 |
| Dietary intake | 39 | Vitamin A (mg) | 6 months after discharge | 4.59 (3.13–6.66) (n=15) | 2.25 (2.25–2.25) (n=1) | W=15.00; Δ_HL=2.34; 95% IC [0.30 ; 9.81] | 0,129 | 0,483 |
| Dietary intake | 40 | Vitamin C (mg) | Preoperative | 367.46 (233.55–590.44) (n=19) | 394.53 (272.15–491.01) (n=4) | W=40.00; Δ_HL=49.86; 95% IC [-236.96 ; 246.20] | 0,903 | 0,945 |
| Dietary intake | 41 | Vitamin C (mg) | 3 months after discharge | 337.47 (212.34–504.52) (n=17) | 199.90 (199.90–199.90) (n=1) | W=15.00; Δ_HL=137.57; 95% IC [-50.58 ; 511.53] | 0,247 | 0,529 |
| Dietary intake | 42 | Vitamin C (mg) | 6 months after discharge | 323.80 (227.23–432.41) (n=15) | 169.56 (169.56–169.56) (n=1) | W=14.00; Δ_HL=154.24; 95% IC [-14.30 ; 517.06] | 0,193 | 0,483 |
| Dietary intake | 43 | Vitamin E (mg) | Preoperative | 4.59 ± 1.55 (n=19) | 4.94 ± 2.41 (n=4) | t(21)=-0.38; Δ_mean=-0.35; 95% IC [-2.30 ; 1.60] | 0,711 | 0,908 |
| Dietary intake | 44 | Vitamin E (mg) | 3 months after discharge | 3.98 (3.12–6.13) (n=17) | 2.34 (2.34–2.34) (n=1) | W=15.00; Δ_HL=1.64; 95% IC [-0.27 ; 7.77] | 0,247 | 0,529 |
| Dietary intake | 45 | Vitamin E (mg) | 6 months after discharge | 3.65 (2.52–5.32) (n=15) | 1.99 (1.99–1.99) (n=1) | W=14.00; Δ_HL=1.66; 95% IC [-0.28 ; 7.17] | 0,193 | 0,483 |
| Clinical–Nutritional Laboratory Analyses | 46 | Total Proteins (g/dL) | Preoperative | 6.84 ± 0.55 (n=19) | 7.31 ± 0.55 (n=4) | t(21)=-1.55; Δ_mean=-0.47; 95% IC [-1.11 ; 0.16] | 0,136 | 0,784 |
| Clinical–Nutritional Laboratory Analyses | 47 | Total Proteins (g/dL) | 3 months after discharge after discharge | 6.80 (6.47–7.13) (n=17) | 7.15 (7.15–7.15) (n=1) | W=4.00; Δ_HL=-0.35; 95% IC [-0.96 ; 0.79] | 0,441 | 0,784 |
| Clinical–Nutritional Laboratory Analyses | 48 | Total Proteins (g/dL) | 6 months after discharge after discharge | 6.56 (6.34–6.86) (n=15) | 7.37 (7.37–7.37) (n=1) | W=2.00; Δ_HL=-0.81; 95% IC [-1.50 ; 0.23] | 0,278 | 0,784 |
| Clinical–Nutritional Laboratory Analyses | 49 | Albumin (g/dL) | Preoperative | 3.95 ± 0.65 (n=19) | 4.01 ± 0.57 (n=4) | t(21)=-0.19; Δ_mean=-0.07; 95% IC [-0.80 ; 0.67] | 0,852 | 0,937 |
| Clinical–Nutritional Laboratory Analyses | 50 | Albumin (g/dL) | 3 months after discharge after discharge | 4.18 (3.98–4.39) (n=17) | 4.32 (4.32–4.32) (n=1) | W=5.00; Δ_HL=-0.14; 95% IC [-1.38 ; 0.53] | 0,563 | 0,784 |
| Clinical–Nutritional Laboratory Analyses | 51 | Albumin (g/dL) | 6 months after discharge after discharge | 4.22 (3.91–4.57) (n=15) | 4.53 (4.53–4.53) (n=1) | W=4.00; Δ_HL=-0.31; 95% IC [-2.39 ; 0.40] | 0,515 | 0,784 |
| Clinical–Nutritional Laboratory Analyses | 52 | C-Reactive Protein (mg/L) | Preoperative | 5.50 (2.36–11.21) (n=19) | 15.88 (3.66–28.59) (n=4) | W=30.50; Δ_HL=-3.74; 95% IC [-25.53 ; 6.48] | 0,570 | 0,784 |
| Clinical–Nutritional Laboratory Analyses | 53 | C-Reactive Protein (mg/L) | 3 months after discharge after discharge | 0.78 (0.40–4.09) (n=17) | 0.40 (0.40–0.40) (n=1) | W=13.50; Δ_HL=0.38; 95% IC [0.00 ; 5.49] | 0,364 | 0,784 |
| Clinical–Nutritional Laboratory Analyses | 54 | C-Reactive Protein (mg/L) | 6 months after discharge after discharge | 0.40 (0.40–1.74) (n=15) | 0.40 (0.40–0.40) (n=1) | W=11.00; Δ_HL=0.00; 95% IC [0.00 ; 12.79] | 0,473 | 0,784 |
| Clinical–Nutritional Laboratory Analyses | 55 | Blood Glucose (mg/dL) | Preoperative | 117.00 (99.58–153.06) (n=19) | 148.72 (117.39–170.50) (n=4) | W=31.50; Δ_HL=-16.26; 95% IC [-68.85 ; 42.00] | 0,626 | 0,810 |
| Clinical–Nutritional Laboratory Analyses | 56 | Blood Glucose (mg/dL) | 3 months after discharge after discharge | 118.00 (84.51–133.95) (n=17) | 134.93 (134.93–134.93) (n=1) | W=4.00; Δ_HL=-16.93; 95% IC [-61.18 ; 137.07] | 0,441 | 0,784 |
| Clinical–Nutritional Laboratory Analyses | 57 | Blood Glucose (mg/dL) | 6 months after discharge after discharge | 106.64 (79.07–118.59) (n=15) | 201.69 (201.69–201.69) (n=1) | W=0.00; Δ_HL=-95.05; 95% IC [-134.20 ; -71.22] | 0,129 | 0,784 |
| Clinical–Nutritional Laboratory Analyses | 58 | Hemoglobin (g/dL) | Preoperative | 12.46 ± 2.06 (n=19) | 14.28 ± 2.34 (n=4) | t(21)=-1.57; Δ_mean=-1.82; 95% IC [-4.22 ; 0.59] | 0,131 | 0,784 |
| Clinical–Nutritional Laboratory Analyses | 59 | Hemoglobin (g/dL) | 3 months after discharge after discharge | 12.80 (11.70–13.16) (n=17) | 14.10 (14.10–14.10) (n=1) | W=3.00; Δ_HL=-1.30; 95% IC [-5.40 ; 0.50] | 0,335 | 0,784 |
| Clinical–Nutritional Laboratory Analyses | 60 | Hemoglobin (g/dL) | 6 months after discharge after discharge | 12.28 (10.50–13.66) (n=15) | 14.60 (14.60–14.60) (n=1) | W=1.00; Δ_HL=-2.32; 95% IC [-6.70 ; 1.20] | 0,192 | 0,784 |
| Clinical–Nutritional Laboratory Analyses | 61 | Lymphocytes (x10³/mm³) | Preoperative | 2.27 ± 1.34 (n=19) | 2.38 ± 0.69 (n=4) | t(21)=-0.15; Δ_mean=-0.11; 95% IC [-1.56 ; 1.35] | 0,880 | 0,952 |
| Clinical–Nutritional Laboratory Analyses | 62 | Lymphocytes (x10³/mm³) | 3 months after discharge after discharge | 2.30 (1.90–3.00) (n=17) | 3.20 (3.20–3.20) (n=1) | W=4.00; Δ_HL=-0.90; 95% IC [-2.50 ; 1.40] | 0,439 | 0,784 |
| Clinical–Nutritional Laboratory Analyses | 63 | Lymphocytes (x10³/mm³) | 6 months after discharge after discharge | 2.10 (1.70–3.20) (n=15) | 2.70 (2.70–2.70) (n=1) | W=6.00; Δ_HL=-0.60; 95% IC [-2.10 ; 1.74] | 0,828 | 0,926 |
| Clinical–Nutritional Laboratory Analyses | 64 | Serum Iron (µg/dL) | Preoperative | 81.79 ± 37.81 (n=19) | 115.63 ± 36.37 (n=4) | t(21)=-1.64; Δ_mean=-33.84; 95% IC [-76.87 ; 9.19] | 0,117 | 0,784 |
| Clinical–Nutritional Laboratory Analyses | 65 | Serum Iron (µg/dL) | 3 months after discharge after discharge | 95.86 (49.63–127.00) (n=17) | 51.97 (51.97–51.97) (n=1) | W=12.00; Δ_HL=43.89; 95% IC [-22.56 ; 147.14] | 0,563 | 0,784 |
| Clinical–Nutritional Laboratory Analyses | 66 | Serum Iron (µg/dL) | 6 months after discharge after discharge | 86.01 (60.85–96.47) (n=15) | 102.93 (102.93–102.93) (n=1) | W=3.00; Δ_HL=-16.92; 95% IC [-79.31 ; 91.00] | 0,386 | 0,784 |
| Clinical–Nutritional Laboratory Analyses | 67 | Ferritin (ng/mL) | Preoperative | 529.40 (173.95–1096.15) (n=19) | 209.10 (176.85–289.17) (n=4) | W=54.50; Δ_HL=248.86; 95% IC [-128.80 ; 1268.40] | 0,194 | 0,784 |
| Clinical–Nutritional Laboratory Analyses | 68 | Ferritin (ng/mL) | 3 months after discharge after discharge | 234.30 (173.10–477.80) (n=17) | 254.60 (254.60–254.60) (n=1) | W=8.00; Δ_HL=-20.30; 95% IC [-234.90 ; 1003.50] | 1,000 | 1,000 |
| Clinical–Nutritional Laboratory Analyses | 69 | Ferritin (ng/mL) | 6 months after discharge after discharge | 217.00 (149.14–415.50) (n=15) | 128.00 (128.00–128.00) (n=1) | W=11.00; Δ_HL=89.00; 95% IC [-94.00 ; 642.30] | 0,515 | 0,784 |
| Clinical–Nutritional Laboratory Analyses | 70 | UIBC (µg/dL) | Preoperative | 285.15 ± 68.21 (n=19) | 314.31 ± 91.47 (n=4) | t(21)=-0.74; Δ_mean=-29.16; 95% IC [-111.53 ; 53.21] | 0,470 | 0,784 |
| Clinical–Nutritional Laboratory Analyses | 71 | UIBC (µg/dL) | 3 months after discharge after discharge | 301.64 (279.32–308.35) (n=17) | 285.00 (285.00–285.00) (n=1) | W=12.00; Δ_HL=16.64; 95% IC [-99.08 ; 137.39] | 0,563 | 0,784 |
| Clinical–Nutritional Laboratory Analyses | 72 | UIBC (µg/dL) | 6 months after discharge after discharge | 296.22 (279.83–317.03) (n=15) | 300.73 (300.73–300.73) (n=1) | W=6.00; Δ_HL=-4.51; 95% IC [-57.64 ; 107.13] | 0,828 | 0,926 |
| Clinical–Nutritional Laboratory Analyses | 73 | TIBC (µg/dL) | Preoperative | 366.94 ± 82.92 (n=19) | 429.94 ± 93.41 (n=4) | t(21)=-1.36; Δ_mean=-63.00; 95% IC [-159.67 ; 33.67] | 0,190 | 0,784 |
| Clinical–Nutritional Laboratory Analyses | 74 | TIBC (µg/dL) | 3 months after discharge after discharge | 406.66 (340.62–431.09) (n=17) | 336.97 (336.97–336.97) (n=1) | W=13.00; Δ_HL=69.69; 95% IC [-121.64 ; 283.93] | 0,441 | 0,784 |
| Clinical–Nutritional Laboratory Analyses | 75 | TIBC (µg/dL) | 6 months after discharge after discharge | 409.04 (341.98–419.61) (n=15) | 403.66 (403.66–403.66) (n=1) | W=8.00; Δ_HL=5.38; 95% IC [-114.68 ; 98.48] | 1,000 | 1,000 |
| Clinical–Nutritional Laboratory Analyses | 76 | Transferrin (mg/dL) | Preoperative | 228.12 ± 54.57 (n=19) | 251.45 ± 73.18 (n=4) | t(21)=-0.74; Δ_mean=-23.33; 95% IC [-89.23 ; 42.56] | 0,470 | 0,784 |
| Clinical–Nutritional Laboratory Analyses | 77 | Transferrin (mg/dL) | 3 months after discharge after discharge | 241.31 (223.46–246.68) (n=17) | 228.00 (228.00–228.00) (n=1) | W=12.00; Δ_HL=13.31; 95% IC [-79.26 ; 109.91] | 0,563 | 0,784 |
| Clinical–Nutritional Laboratory Analyses | 78 | Transferrin (mg/dL) | 6 months after discharge after discharge | 236.98 (223.86–253.63) (n=15) | 240.58 (240.58–240.58) (n=1) | W=6.00; Δ_HL=-3.61; 95% IC [-46.11 ; 85.70] | 0,828 | 0,926 |
| Clinical–Nutritional Laboratory Analyses | 79 | Transferrin Saturation (%) | Preoperative | 21.98 ± 8.09 (n=19) | 27.48 ± 8.50 (n=4) | t(21)=-1.23; Δ_mean=-5.50; 95% IC [-14.82 ; 3.82] | 0,233 | 0,784 |
| Clinical–Nutritional Laboratory Analyses | 80 | Transferrin Saturation (%) | 3 months after discharge after discharge | 23.74 (13.66–30.55) (n=17) | 15.42 (15.42–15.42) (n=1) | W=12.00; Δ_HL=8.31; 95% IC [-4.43 ; 24.04] | 0,563 | 0,784 |
| Clinical–Nutritional Laboratory Analyses | 81 | Transferrin Saturation (%) | 6 months after discharge after discharge | 20.21 (16.99–24.48) (n=15) | 25.50 (25.50–25.50) (n=1) | W=3.00; Δ_HL=-5.29; 95% IC [-17.57 ; 13.96] | 0,386 | 0,784 |
| Clinical–Nutritional Laboratory Analyses | 82 | Urea (mg/dL) | Preoperative | 32.27 (23.05–39.71) (n=19) | 34.72 (32.41–45.16) (n=4) | W=33.00; Δ_HL=-5.11; 95% IC [-37.09 ; 10.49] | 0,715 | 0,874 |
| Clinical–Nutritional Laboratory Analyses | 83 | Urea (mg/dL) | 3 months after discharge after discharge | 35.01 (27.71–51.77) (n=17) | 13.91 (13.91–13.91) (n=1) | W=17.00; Δ_HL=21.10; 95% IC [8.82 ; 56.47] | 0,123 | 0,784 |
| Clinical–Nutritional Laboratory Analyses | 84 | Urea (mg/dL) | 6 months after discharge after discharge | 32.73 (23.95–41.57) (n=15) | 19.62 (19.62–19.62) (n=1) | W=14.00; Δ_HL=13.11; 95% IC [-5.52 ; 113.42] | 0,193 | 0,784 |
| Clinical–Nutritional Laboratory Analyses | 85 | Creatinine (mg/dL) | Preoperative | 0.87 (0.78–1.16) (n=19) | 0.96 (0.88–1.23) (n=4) | W=32.00; Δ_HL=-0.10; 95% IC [-0.80 ; 0.37] | 0,655 | 0,831 |
| Clinical–Nutritional Laboratory Analyses | 86 | Creatinine (mg/dL) | 3 months after discharge after discharge | 0.87 (0.75–0.96) (n=17) | 0.69 (0.69–0.69) (n=1) | W=16.00; Δ_HL=0.18; 95% IC [-0.04 ; 1.48] | 0,177 | 0,784 |
| Clinical–Nutritional Laboratory Analyses | 87 | Creatinine (mg/dL) | 6 months after discharge after discharge | 0.87 (0.76–1.10) (n=15) | 0.95 (0.95–0.95) (n=1) | W=6.00; Δ_HL=-0.08; 95% IC [-0.27 ; 1.82] | 0,828 | 0,926 |
| Clinical–Nutritional Laboratory Analyses | 88 | Aspartate Aminotransferase (AST) (U/L) | Preoperative | 154.30 ± 103.01 (n=19) | 282.54 ± 264.88 (n=4) | t(3.19)=-0.95; Δ_mean=-128.24; 95% IC [-542.06 ; 285.58] | 0,407 | 0,784 |
| Clinical–Nutritional Laboratory Analyses | 89 | Aspartate Aminotransferase (AST) (U/L) | 3 months after discharge after discharge | 27.47 (23.74–36.91) (n=17) | 36.98 (36.98–36.98) (n=1) | W=4.00; Δ_HL=-9.51; 95% IC [-22.16 ; 74.96] | 0,441 | 0,784 |
| Clinical–Nutritional Laboratory Analyses | 90 | Aspartate Aminotransferase (AST) (U/L) | 6 months after discharge after discharge | 29.22 (26.46–38.42) (n=15) | 38.81 (38.81–38.81) (n=1) | W=4.00; Δ_HL=-9.59; 95% IC [-21.10 ; 28.85] | 0,515 | 0,784 |
| Clinical–Nutritional Laboratory Analyses | 91 | Aminotransferase (ALT) (U/L) | Preoperative | 148.88 (65.79–224.51) (n=19) | 344.00 (77.48–649.86) (n=4) | W=28.00; Δ_HL=-62.65; 95% IC [-585.86 ; 141.07] | 0,441 | 0,784 |
| Clinical–Nutritional Laboratory Analyses | 92 | Aminotransferase (ALT) (U/L) | 3 months after discharge after discharge | 33.04 (28.37–40.39) (n=17) | 45.37 (45.37–45.37) (n=1) | W=4.00; Δ_HL=-12.33; 95% IC [-30.90 ; 23.20] | 0,441 | 0,784 |
| Clinical–Nutritional Laboratory Analyses | 93 | Aminotransferase (ALT) (U/L) | 6 months after discharge after discharge | 34.21 (25.25–40.95) (n=15) | 54.27 (54.27–54.27) (n=1) | W=1.00; Δ_HL=-20.06; 95% IC [-41.07 ; 32.57] | 0,193 | 0,784 |
| Clinical–Nutritional Laboratory Analyses | 94 | Folic Acid (ng/mL) | Preoperative | 14.67 ± 6.21 (n=19) | 14.98 ± 6.50 (n=4) | t(21)=-0.09; Δ_mean=-0.32; 95% IC [-7.47 ; 6.83] | 0,928 | 0,988 |
| Clinical–Nutritional Laboratory Analyses | 95 | Folic Acid (ng/mL) | 3 months after discharge after discharge | 18.73 (10.87–24.00) (n=17) | 9.10 (9.10–9.10) (n=1) | W=15.00; Δ_HL=9.63; 95% IC [-1.81 ; 14.90] | 0,233 | 0,784 |
| Clinical–Nutritional Laboratory Analyses | 96 | Folic Acid (ng/mL) | 6 months after discharge after discharge | 21.56 (16.20–24.00) (n=15) | 11.66 (11.66–11.66) (n=1) | W=12.00; Δ_HL=9.90; 95% IC [-4.63 ; 12.34] | 0,378 | 0,784 |
| Clinical–Nutritional Laboratory Analyses | 97 | Vitamin B12 (pg/mL) | Preoperative | 501.00 (412.50–752.00) (n=19) | 522.00 (420.75–598.00) (n=4) | W=45.00; Δ_HL=63.00; 95% IC [-170.00 ; 498.00] | 0,597 | 0,788 |
| Clinical–Nutritional Laboratory Analyses | 98 | Vitamin B12 (pg/mL) | 3 months after discharge after discharge | 495.00 (386.00–761.00) (n=17) | 372.00 (372.00–372.00) (n=1) | W=13.00; Δ_HL=123.00; 95% IC [-134.00 ; 1628.00] | 0,441 | 0,784 |
| Clinical–Nutritional Laboratory Analyses | 99 | Vitamin B12 (pg/mL) | 6 months after discharge after discharge | 392.00 (325.65–558.00) (n=15) | 313.00 (313.00–313.00) (n=1) | W=12.50; Δ_HL=79.00; 95% IC [-19.00 ; 1456.33] | 0,329 | 0,784 |
| Clinical–Nutritional Laboratory Analyses | 100 | Vitamin A (µg/dL) | Preoperative | 0.31 (0.19–0.35) (n=19) | 0.30 (0.27–0.32) (n=4) | W=37.00; Δ_HL=-0.00; 95% IC [-0.13 ; 0.14] | 0,967 | 1,000 |
| Clinical–Nutritional Laboratory Analyses | 101 | Vitamin A (µg/dL) | 3 months after discharge after discharge | 0.33 (0.22–0.40) (n=17) | 0.32 (0.32–0.32) (n=1) | W=9.00; Δ_HL=0.01; 95% IC [-0.19 ; 0.23] | 1,000 | 1,000 |
| Clinical–Nutritional Laboratory Analyses | 102 | Vitamin A (µg/dL) | 6 months after discharge after discharge | 0.32 (0.28–0.37) (n=15) | 0.21 (0.21–0.21) (n=1) | W=15.00; Δ_HL=0.11; 95% IC [0.03 ; 0.40] | 0,127 | 0,784 |
| Clinical–Nutritional Laboratory Analyses | 103 | Vitamin E (mg/dL) | Preoperative | 8.11 (6.06–9.33) (n=19) | 7.28 (7.28–7.60) (n=4) | W=43.50; Δ_HL=0.18; 95% IC [-1.48 ; 5.08] | 0,684 | 0,852 |
| Clinical–Nutritional Laboratory Analyses | 104 | Vitamin E (mg/dL) | 3 months after discharge after discharge | 6.59 (4.57–7.08) (n=17) | 8.29 (8.29–8.29) (n=1) | W=3.00; Δ_HL=-1.70; 95% IC [-4.70 ; 3.00] | 0,335 | 0,784 |
| Clinical–Nutritional Laboratory Analyses | 105 | Vitamin E (mg/dL) | 6 months after discharge after discharge | 5.64 (4.78–6.62) (n=15) | 5.09 (5.09–5.09) (n=1) | W=9.00; Δ_HL=0.55; 95% IC [-1.61 ; 13.20] | 0,828 | 0,926 |
| Clinical–Nutritional Laboratory Analyses | 106 | Copper (µg/dL) | Preoperative | 141.00 (109.00–153.50) (n=19) | 117.00 (89.70–173.40) (n=4) | W=48.00; Δ_HL=14.42; 95% IC [-131.20 ; 69.20] | 0,440 | 0,784 |
| Clinical–Nutritional Laboratory Analyses | 107 | Copper (µg/dL) | 3 months after discharge after discharge | 106.60 (91.40–116.20) (n=17) | 61.20 (61.20–61.20) (n=1) | W=17.00; Δ_HL=45.40; 95% IC [3.80 ; 133.20] | 0,123 | 0,784 |
| Clinical–Nutritional Laboratory Analyses | 108 | Copper (µg/dL) | 6 months after discharge after discharge | 95.00 (90.81–114.25) (n=15) | 59.08 (59.08–59.08) (n=1) | W=15.00; Δ_HL=35.92; 95% IC [3.61 ; 111.12] | 0,129 | 0,784 |
| Clinical–Nutritional Laboratory Analyses | 109 | Zinc (µg/dL) | Preoperative | 90.00 (62.50–96.00) (n=19) | 82.25 (73.12–87.75) (n=4) | W=45.00; Δ_HL=5.00; 95% IC [-25.50 ; 25.50] | 0,596 | 0,788 |
| Clinical–Nutritional Laboratory Analyses | 110 | Zinc (µg/dL) | 3 months after discharge after discharge | 77.00 (74.50–86.00) (n=17) | 121.50 (121.50–121.50) (n=1) | W=1.00; Δ_HL=-44.50; 95% IC [-55.00 ; 28.50] | 0,176 | 0,784 |
| Clinical–Nutritional Laboratory Analyses | 111 | Zinc (µg/dL) | 6 months after discharge after discharge | 80.50 (71.13–88.50) (n=15) | 70.50 (70.50–70.50) (n=1) | W=11.00; Δ_HL=10.00; 95% IC [-26.50 ; 34.00] | 0,515 | 0,784 |
| Derived inflammatory–nutritional indices | 112 | Prognostic Nutritional Index | Preoperative | 50.80 ± 10.38 (n=19) | 52.00 ± 6.79 (n=4) | t(21)=-0.22; Δ_mean=-1.20; 95% IC [-12.58 ; 10.18] | 0,829 | 0,968 |
| Derived inflammatory–nutritional indices | 113 | Prognostic Nutritional Index | 3 months after discharge | 55.00 (49.20–57.90) (n=17) | 59.20 (59.20–59.20) (n=1) | W=4.00; Δ_HL=-4.20; 95% IC [-20.30 ; 6.60] | 0,441 | 0,774 |
| Derived inflammatory–nutritional indices | 114 | Prognostic Nutritional Index | 6 months after discharge | 55.50 (49.10–59.75) (n=15) | 58.80 (58.80–58.80) (n=1) | W=4.00; Δ_HL=-3.30; 95% IC [-31.40 ; 4.68] | 0,515 | 0,774 |
| Derived inflammatory–nutritional indices | 115 | Glasgow Prognostic Score (GPS) | Preoperative | 0: 9 (47.4%); 1: 8 (42.1%); 2: 2 (10.5%) | 0: 2 (50.0%); 1: 1 (25.0%); 2: 1 (25.0%) | W=36.00; Δ_HL=-0.00; 95% IC [-1.00 ; 1.00] | 0,894 | 0,975 |
| Derived inflammatory–nutritional indices | 116 | Glasgow Prognostic Score (GPS) | 3 months after discharge | 0: 14 (82.4%); 1: 3 (17.6%) | 0: 1 (100.0%) | W=10.00; Δ_HL=0.00; 95% IC [0.00 ; 1.00] | 0,766 | 0,968 |
| Derived inflammatory–nutritional indices | 117 | Glasgow Prognostic Score (GPS) | 6 months after discharge | 0: 14 (93.3%); 2: 1 (6.7%) | 0: 1 (100.0%) | W=8.00; Δ_HL=0.00; 95% IC [0.00 ; 0.00] | 1,000 | 1,000 |
| Derived inflammatory–nutritional indices | 118 | Albumin/CRP ratio | Preoperative | 0.80 (0.29–1.80) (n=19) | 0.47 (0.13–3.41) (n=4) | W=47.00; Δ_HL=0.10; 95% IC [-9.76 ; 1.67] | 0,491 | 0,774 |
| Derived inflammatory–nutritional indices | 119 | Albumin/CRP ratio | 3 months after discharge | 5.22 (0.83–10.70) (n=17) | 10.80 (10.80–10.80) (n=1) | W=3.00; Δ_HL=-5.58; 95% IC [-10.26 ; 1.15] | 0,335 | 0,774 |
| Derived inflammatory–nutritional indices | 120 | Albumin/CRP ratio | 6 months after discharge | 9.12 (2.49–10.54) (n=15) | 11.32 (11.32–11.32) (n=1) | W=2.00; Δ_HL=-2.20; 95% IC [-11.16 ; 1.00] | 0,278 | 0,774 |
| Derived inflammatory–nutritional indices | 121 | CRP/Albumin ratio | Preoperative | 1.25 (0.57–3.44) (n=19) | 4.37 (0.96–7.66) (n=4) | W=29.50; Δ_HL=-0.93; 95% IC [-6.94 ; 2.12] | 0,516 | 0,774 |
| Derived inflammatory–nutritional indices | 122 | CRP/Albumin ratio | 3 months after discharge | 1.59 (0.75–911.16) (n=17) | 925.93 (925.93–925.93) (n=1) | W=4.00; Δ_HL=-924.33; 95% IC [-925.77 ; 31.01] | 0,440 | 0,774 |
| Derived inflammatory–nutritional indices | 123 | CRP/Albumin ratio | 6 months after discharge | 0.81 (0.29–879.31) (n=15) | 883.00 (883.00–883.00) (n=1) | W=4.00; Δ_HL=-882.20; 95% IC [-882.90 ; 85.52] | 0,515 | 0,774 |
| Derived inflammatory–nutritional indices | 124 | CRP/Lymphocytes ratio | Preoperative | 2.63 (1.25–7.16) (n=19) | 6.32 (2.01–12.13) (n=4) | W=30.50; Δ_HL=-1.41; 95% IC [-11.51 ; 4.67] | 0,570 | 0,805 |
| Derived inflammatory–nutritional indices | 125 | CRP/Lymphocytes ratio | 3 months after discharge | 0.77 (0.20–2.27) (n=17) | 0.12 (0.12–0.12) (n=1) | W=15.00; Δ_HL=0.64; 95% IC [-0.01 ; 869.44] | 0,247 | 0,774 |
| Derived inflammatory–nutritional indices | 126 | CRP/Lymphocytes ratio | 6 months after discharge | 0.38 (0.18–1.56) (n=15) | 0.15 (0.15–0.15) (n=1) | W=12.00; Δ_HL=0.24; 95% IC [-0.04 ; 901.52] | 0,386 | 0,774 |
| Derived inflammatory–nutritional indices | 127 | Ferritin/Albumin ratio | Preoperative | 140.38 (39.99–290.89) (n=19) | 46.73 (40.92–70.07) (n=4) | W=54.00; Δ_HL=59.87; 95% IC [-25.40 ; 283.53] | 0,209 | 0,774 |
| Derived inflammatory–nutritional indices | 128 | Ferritin/Albumin ratio | 3 months after discharge | 59.04 (40.07–125.66) (n=17) | 58.94 (58.94–58.94) (n=1) | W=9.00; Δ_HL=0.10; 95% IC [-54.33 ; 257.17] | 1,000 | 1,000 |
| Derived inflammatory–nutritional indices | 129 | Ferritin/Albumin ratio | 6 months after discharge | 50.73 (34.48–107.56) (n=15) | 28.26 (28.26–28.26) (n=1) | W=12.00; Δ_HL=22.47; 95% IC [-21.36 ; 331.70] | 0,386 | 0,774 |
| Derived inflammatory–nutritional indices | 130 | Ferritin/Lymphocytes ratio | Preoperative | 383.55 (65.66–524.70) (n=19) | 90.76 (56.42–160.65) (n=4) | W=55.00; Δ_HL=216.06; 95% IC [-62.90 ; 902.89] | 0,181 | 0,774 |
| Derived inflammatory–nutritional indices | 131 | Ferritin/Lymphocytes ratio | 3 months after discharge | 103.87 (63.26–158.37) (n=17) | 79.56 (79.56–79.56) (n=1) | W=10.00; Δ_HL=24.31; 95% IC [-72.53 ; 549.49] | 0,847 | 0,968 |
| Derived inflammatory–nutritional indices | 132 | Ferritin/Lymphocytes ratio | 6 months after discharge | 103.87 (62.07–153.62) (n=15) | 47.41 (47.41–47.41) (n=1) | W=12.00; Δ_HL=56.46; 95% IC [-32.62 ; 594.51] | 0,386 | 0,774 |
| Derived inflammatory–nutritional indices | 133 | Albumin-Lymphocytes-CRP index | Preoperative | 1.37 (0.56–3.61) (n=19) | 0.89 (0.31–10.08) (n=4) | W=44.00; Δ_HL=0.19; 95% IC [-32.72 ; 3.04] | 0,656 | 0,875 |
| Derived inflammatory–nutritional indices | 134 | Albumin-Lymphocytes-CRP index | 3 months after discharge | 8.87 (1.65–23.90) (n=17) | 34.56 (34.56–34.56) (n=1) | W=3.00; Δ_HL=-25.69; 95% IC [-33.37 ; 14.66] | 0,335 | 0,774 |
| Derived inflammatory–nutritional indices | 135 | Albumin-Lymphocytes-CRP index | 6 months after discharge | 14.60 (6.18–31.35) (n=15) | 30.58 (30.58–30.58) (n=1) | W=4.00; Δ_HL=-15.98; 95% IC [-30.38 ; 15.23] | 0,515 | 0,774 |
| Notes: Values are presented as mean +/- standard deviation or median (P25-P75), with the valid n shown for each group and comparison. The analysis used observed available data only; no multiple imputation was performed. The number of patients evaluated was preoperative n = 24, 3 months after discharge n = 18, and 6 months after discharge n = 16. Raw p-values correspond to the statistical test reported in each row. BH-FDR adjusted p-values were calculated with the Benjamini-Hochberg false discovery rate procedure within each Exposure x Domain family: Dietary intake (45 tests), Clinical-nutritional laboratory analyses (66 tests), and Derived inflammatory-nutritional indices (24 tests). The BH-FDR audit found 945 inferential comparisons, 43 raw p-values < 0.05, and 0 BH-FDR adjusted p-values < 0.05. For binary exposures, two-sample t-test/Welch t-test or Mann-Whitney/Wilcoxon rank-sum tests were used as reported. For multicategorical or ordinal exposures, one-way ANOVA or Kruskal-Wallis tests were used; these are omnibus tests and do not establish pairwise differences or dose-response trends without post-hoc or contrast testing. Because this was an exploratory supplementary analysis with multiple comparisons, small subgroup sizes, and no covariate adjustment, the results should be interpreted as hypothesis-generating rather than confirmatory. Abbreviations: BH-FDR, Benjamini-Hochberg false discovery rate; EPI, Exocrine pancreatic insufficiency; TIBC, total iron-binding capacity; UIBC, unsaturated iron-binding capacity. | | | | | | | | |

| **Supplementary Table S10.** Exploratory associations between EPI grade and dietary intake, clinical-nutritional laboratory variables, and inflammatory-nutritional indices after pancreatoduodenectomy. | | | | | | | | | | |
| --- | --- | --- | --- | --- | --- | --- | --- | --- | --- | --- |
| **Outcome** | | | | **EPI grade** | | | | | | |
| **Domain** | **Source row** | **Variable** | **Time** | **Normal** | **Mild** | **Moderate** | **Severe** | **Statistical result** | **Raw p-value** | **BH-FDR adjusted p-value** |
|  |  |  |  | **Mean ± SD or Median (P25–P75), n** | **Mean ± SD or Median (P25–P75), n** | **Mean ± SD or Median (P25–P75), n** | **Mean ± SD or Median (P25–P75), n** |  |  |  |
| Dietary intake | 1 | Energy (kcal) | Preoperative | 3188.56 ± 460.89 (n=4) | NA | 2552.51 ± 496.91 (n=11) | 2767.11 ± 651.12 (n=8) | F(2, 20)=1.97 | 0,165 | 0,749 |
| Dietary intake | 2 | Energy (kcal) | 3 months after discharge | 1955.01 (1955.01–1955.01) (n=1) | NA | 1983.44 (1695.87–2306.26) (n=10) | 2029.30 (1957.90–2203.31) (n=7) | H(2)=0.29 | 0,866 | 0,957 |
| Dietary intake | 3 | Energy (kcal) | 6 months after discharge | 1656.00 (1656.00–1656.00) (n=1) | NA | 2014.08 (1917.77–2178.34) (n=9) | 1912.90 (1853.25–1996.08) (n=6) | H(2)=2.89 | 0,235 | 0,749 |
| Dietary intake | 4 | Protein (g) | Preoperative | 127.97 ± 14.98 (n=4) | NA | 118.90 ± 22.02 (n=11) | 112.81 ± 27.98 (n=8) | F(2, 20)=0.56 | 0,579 | 0,957 |
| Dietary intake | 5 | Protein (g) | 3 months after discharge | 81.37 (81.37–81.37) (n=1) | NA | 95.19 (88.50–106.07) (n=10) | 95.89 (89.45–103.03) (n=7) | H(2)=2.14 | 0,343 | 0,749 |
| Dietary intake | 6 | Protein (g) | 6 months after discharge | 107.49 (107.49–107.49) (n=1) | NA | 96.86 (96.22–102.68) (n=9) | 94.87 (89.80–107.74) (n=6) | H(2)=0.66 | 0,719 | 0,957 |
| Dietary intake | 7 | Relative energy balance (kcal) | Preoperative | 1380.06 ± 171.91 (n=4) | NA | 1098.69 ± 288.78 (n=11) | 1020.43 ± 388.48 (n=8) | F(2, 20)=1.79 | 0,192 | 0,749 |
| Dietary intake | 8 | Relative energy balance (kcal) | 3 months after discharge | 317.08 (317.08–317.08) (n=1) | NA | 491.32 (444.01–578.55) (n=10) | 574.58 (376.99–820.64) (n=7) | H(2)=2.11 | 0,349 | 0,749 |
| Dietary intake | 9 | Relative energy balance (kcal) | 6 months after discharge | 578.33 (578.33–578.33) (n=1) | NA | 614.48 (501.44–679.91) (n=9) | 635.08 (546.64–817.15) (n=6) | H(2)=0.40 | 0,817 | 0,957 |
| Dietary intake | 10 | Carbohydrate (g) | Preoperative | 437.36 ± 55.30 (n=4) | NA | 352.02 ± 74.25 (n=11) | 387.05 ± 85.30 (n=8) | F(2, 20)=1.92 | 0,172 | 0,749 |
| Dietary intake | 11 | Carbohydrate (g) | 3 months after discharge | 261.97 (261.97–261.97) (n=1) | NA | 257.10 (228.73–278.84) (n=10) | 271.36 (254.83–280.61) (n=7) | H(2)=0.76 | 0,685 | 0,957 |
| Dietary intake | 12 | Carbohydrate (g) | 6 months after discharge | 223.71 (223.71–223.71) (n=1) | NA | 268.49 (249.84–279.88) (n=9) | 263.61 (238.44–277.19) (n=6) | H(2)=2.07 | 0,355 | 0,749 |
| Dietary intake | 13 | Lipid (g) | Preoperative | 103.03 ± 23.43 (n=4) | NA | 74.31 ± 19.97 (n=11) | 85.29 ± 26.36 (n=8) | F(2, 20)=2.36 | 0,120 | 0,749 |
| Dietary intake | 14 | Lipid (g) | 3 months after discharge | 64.63 (64.63–64.63) (n=1) | NA | 66.33 (50.42–80.21) (n=10) | 62.92 (57.41–71.28) (n=7) | H(2)=0.01 | 0,995 | 0,995 |
| Dietary intake | 15 | Lipid (g) | 6 months after discharge | 36.80 (36.80–36.80) (n=1) | NA | 60.48 (58.13–78.45) (n=9) | 55.91 (50.14–58.91) (n=6) | H(2)=3.43 | 0,180 | 0,749 |
| Dietary intake | 16 | Fiber (g) | Preoperative | 55.32 ± 6.03 (n=4) | NA | 42.27 ± 10.98 (n=11) | 47.86 ± 11.88 (n=8) | F(2, 20)=2.27 | 0,129 | 0,749 |
| Dietary intake | 17 | Fiber (g) | 3 months after discharge | 31.76 (31.76–31.76) (n=1) | NA | 37.16 (33.55–45.64) (n=10) | 35.99 (34.84–43.13) (n=7) | H(2)=1.12 | 0,570 | 0,957 |
| Dietary intake | 18 | Fiber (g) | 6 months after discharge | 26.98 (26.98–26.98) (n=1) | NA | 41.87 (34.69–44.42) (n=9) | 35.83 (35.16–37.63) (n=6) | H(2)=2.25 | 0,325 | 0,749 |
| Dietary intake | 19 | Calcium (mg) | Preoperative | 1434.32 (1372.96–1587.74) (n=4) | NA | 1499.59 (1197.08–1660.60) (n=11) | 1656.26 (1252.08–1726.38) (n=8) | H(2)=0.24 | 0,885 | 0,957 |
| Dietary intake | 20 | Calcium (mg) | 3 months after discharge | 1093.02 (1093.02–1093.02) (n=1) | NA | 1165.10 (985.34–1642.23) (n=10) | 1331.73 (1162.84–1439.84) (n=7) | H(2)=0.49 | 0,784 | 0,957 |
| Dietary intake | 21 | Calcium (mg) | 6 months after discharge | 926.12 (926.12–926.12) (n=1) | NA | 1068.66 (1006.62–1638.94) (n=9) | 1265.51 (1111.18–1344.02) (n=6) | H(2)=2.01 | 0,366 | 0,749 |
| Dietary intake | 22 | Iron (mg) | Preoperative | 33.98 ± 5.56 (n=4) | NA | 24.98 ± 3.59 (n=11) | 27.64 ± 8.23 (n=8) | F(2, 20)=3.420.833 | 0,053 | 0,749 |
| Dietary intake | 23 | Iron (mg) | 3 months after discharge | 22.11 (22.11–22.11) (n=1) | NA | 22.99 (18.26–27.44) (n=10) | 19.92 (17.17–27.05) (n=7) | H(2)=0.25 | 0,883 | 0,957 |
| Dietary intake | 24 | Iron (mg) | 6 months after discharge | 18.72 (18.72–18.72) (n=1) | NA | 23.82 (20.71–26.01) (n=9) | 18.20 (16.14–23.79) (n=6) | H(2)=2.17 | 0,338 | 0,749 |
| Dietary intake | 25 | Potassium (mg) | Preoperative | 7775.76 ± 1100.90 (n=4) | NA | 6430.19 ± 1187.40 (n=11) | 6717.88 ± 1426.80 (n=8) | F(2, 20)=1.67 | 0,214 | 0,749 |
| Dietary intake | 26 | Potassium (mg) | 3 months after discharge | 5039.31 (5039.31–5039.31) (n=1) | NA | 5524.00 (5020.64–6980.24) (n=10) | 5133.78 (4640.01–6022.35) (n=7) | H(2)=1.01 | 0,605 | 0,957 |
| Dietary intake | 27 | Potassium (mg) | 6 months after discharge | 4270.76 (4270.76–4270.76) (n=1) | NA | 6372.40 (5226.42–6728.03) (n=9) | 4770.86 (4506.95–5252.26) (n=6) | H(2)=4.600.961 | 0,100 | 0,749 |
| Dietary intake | 28 | Selenium (µg) | Preoperative | 60.78 ± 31.69 (n=4) | NA | 60.25 ± 17.83 (n=11) | 53.05 ± 17.42 (n=8) | F(2, 20)=0.34 | 0,716 | 0,957 |
| Dietary intake | 29 | Selenium (µg) | 3 months after discharge | 35.14 (35.14–35.14) (n=1) | NA | 48.41 (35.90–64.92) (n=10) | 39.85 (38.34–55.47) (n=7) | H(2)=0.80 | 0,670 | 0,957 |
| Dietary intake | 30 | Selenium (µg) | 6 months after discharge | 29.84 (29.84–29.84) (n=1) | NA | 52.03 (41.03–65.65) (n=9) | 38.75 (38.28–55.11) (n=6) | H(2)=2.25 | 0,325 | 0,749 |
| Dietary intake | 31 | Zinc (mg) | Preoperative | 22.79 ± 5.40 (n=4) | NA | 16.65 ± 3.01 (n=11) | 18.63 ± 7.47 (n=8) | F(2, 20)=1.96 | 0,167 | 0,749 |
| Dietary intake | 32 | Zinc (mg) | 3 months after discharge | 14.76 (14.76–14.76) (n=1) | NA | 13.56 (11.60–15.41) (n=10) | 12.73 (11.54–18.13) (n=7) | H(2)=0.12 | 0,941 | 0,962 |
| Dietary intake | 33 | Zinc (mg) | 6 months after discharge | 12.51 (12.51–12.51) (n=1) | NA | 13.93 (13.07–16.03) (n=9) | 11.87 (11.24–14.43) (n=6) | H(2)=0.81 | 0,666 | 0,957 |
| Dietary intake | 34 | Sodium (mg) | Preoperative | 1816.14 (1138.78–2470.38) (n=4) | NA | 1675.20 (1310.32–2354.61) (n=11) | 1592.51 (1381.33–2101.39) (n=8) | H(2)=0.17 | 0,919 | 0,962 |
| Dietary intake | 35 | Sodium (mg) | 3 months after discharge | 1189.32 (1189.32–1189.32) (n=1) | NA | 1258.16 (1127.98–1648.89) (n=10) | 1390.15 (1093.95–1657.34) (n=7) | H(2)=0.33 | 0,846 | 0,957 |
| Dietary intake | 36 | Sodium (mg) | 6 months after discharge | 1008.44 (1008.44–1008.44) (n=1) | NA | 1169.09 (1114.30–1601.63) (n=9) | 1240.13 (999.88–1534.59) (n=6) | H(2)=0.65 | 0,724 | 0,957 |
| Dietary intake | 37 | Vitamin A (mg) | Preoperative | 6.29 ± 3.32 (n=4) | NA | 5.45 ± 2.88 (n=11) | 5.67 ± 2.95 (n=8) | F(2, 20)=0.12 | 0,891 | 0,957 |
| Dietary intake | 38 | Vitamin A (mg) | 3 months after discharge | 2.66 (2.66–2.66) (n=1) | NA | 4.67 (3.90–7.83) (n=10) | 4.08 (3.14–6.11) (n=7) | H(2)=1.89 | 0,388 | 0,759 |
| Dietary intake | 39 | Vitamin A (mg) | 6 months after discharge | 2.25 (2.25–2.25) (n=1) | NA | 4.76 (4.54–8.69) (n=9) | 3.67 (2.94–4.55) (n=6) | H(2)=4.570.961 | 0,102 | 0,749 |
| Dietary intake | 40 | Vitamin C (mg) | Preoperative | 394.53 (272.15–491.01) (n=4) | NA | 315.77 (220.72–518.03) (n=11) | 411.59 (261.67–563.29) (n=8) | H(2)=0.42 | 0,811 | 0,957 |
| Dietary intake | 41 | Vitamin C (mg) | 3 months after discharge | 199.90 (199.90–199.90) (n=1) | NA | 305.42 (220.12–555.88) (n=10) | 348.32 (262.56–426.62) (n=7) | H(2)=1.60 | 0,450 | 0,844 |
| Dietary intake | 42 | Vitamin C (mg) | 6 months after discharge | 169.56 (169.56–169.56) (n=1) | NA | 323.80 (239.87–603.03) (n=9) | 317.09 (237.12–334.48) (n=6) | H(2)=2.37 | 0,305 | 0,749 |
| Dietary intake | 43 | Vitamin E (mg) | Preoperative | 4.94 ± 2.41 (n=4) | NA | 4.69 ± 1.59 (n=11) | 4.45 ± 1.59 (n=8) | F(2, 20)=0.11 | 0,893 | 0,957 |
| Dietary intake | 44 | Vitamin E (mg) | 3 months after discharge | 2.34 (2.34–2.34) (n=1) | NA | 4.79 (3.22–6.00) (n=10) | 3.44 (2.75–5.37) (n=7) | H(2)=2.29 | 0,319 | 0,749 |
| Dietary intake | 45 | Vitamin E (mg) | 6 months after discharge | 1.99 (1.99–1.99) (n=1) | NA | 4.82 (3.65–5.92) (n=9) | 2.70 (2.13–3.28) (n=6) | H(2)=7.190.833 | 0,027 | 0,749 |
| Clinical–Nutritional Laboratory Analyses | 46 | Total Proteins (g/dL) | Preoperative | 7.31 ± 0.55 (n=4) | NA | 7.02 ± 0.43 (n=11) | 6.58 ± 0.63 (n=8) | F(2, 20)=3.02 | 0,071 | 0,675 |
| Clinical–Nutritional Laboratory Analyses | 47 | Total Proteins (g/dL) | 3 months after discharge after discharge | 7.15 (7.15–7.15) (n=1) | NA | 6.71 (6.48–7.14) (n=10) | 6.87 (6.62–7.01) (n=7) | H(2)=0.75 | 0,686 | 0,816 |
| Clinical–Nutritional Laboratory Analyses | 48 | Total Proteins (g/dL) | 6 months after discharge after discharge | 7.37 (7.37–7.37) (n=1) | NA | 6.56 (6.42–7.03) (n=9) | 6.45 (6.11–6.67) (n=6) | H(2)=2.60 | 0,272 | 0,675 |
| Clinical–Nutritional Laboratory Analyses | 49 | Albumin (g/dL) | Preoperative | 4.01 ± 0.57 (n=4) | NA | 4.05 ± 0.72 (n=11) | 3.81 ± 0.55 (n=8) | F(2, 20)=0.33 | 0,724 | 0,836 |
| Clinical–Nutritional Laboratory Analyses | 50 | Albumin (g/dL) | 3 months after discharge after discharge | 4.32 (4.32–4.32) (n=1) | NA | 4.23 (4.03–4.61) (n=10) | 3.98 (3.58–4.27) (n=7) | H(2)=2.52 | 0,283 | 0,675 |
| Clinical–Nutritional Laboratory Analyses | 51 | Albumin (g/dL) | 6 months after discharge after discharge | 4.53 (4.53–4.53) (n=1) | NA | 4.39 (3.97–4.73) (n=9) | 4.14 (3.92–4.25) (n=6) | H(2)=2.23 | 0,329 | 0,675 |
| Clinical–Nutritional Laboratory Analyses | 52 | C-Reactive Protein (mg/L) | Preoperative | 15.88 (3.66–28.59) (n=4) | NA | 6.88 (3.41–15.02) (n=11) | 4.86 (1.58–9.09) (n=8) | H(2)=0.74 | 0,692 | 0,816 |
| Clinical–Nutritional Laboratory Analyses | 53 | C-Reactive Protein (mg/L) | 3 months after discharge after discharge | 0.40 (0.40–0.40) (n=1) | NA | 0.77 (0.40–3.07) (n=10) | 1.76 (0.40–4.45) (n=7) | H(2)=1.16 | 0,559 | 0,802 |
| Clinical–Nutritional Laboratory Analyses | 54 | C-Reactive Protein (mg/L) | 6 months after discharge after discharge | 0.40 (0.40–0.40) (n=1) | NA | 0.58 (0.40–1.64) (n=9) | 0.40 (0.40–1.48) (n=6) | H(2)=0.87 | 0,647 | 0,816 |
| Clinical–Nutritional Laboratory Analyses | 55 | Blood Glucose (mg/dL) | Preoperative | 148.72 (117.39–170.50) (n=4) | NA | 104.00 (95.58–135.50) (n=11) | 136.50 (121.31–153.10) (n=8) | H(2)=1.59 | 0,451 | 0,726 |
| Clinical–Nutritional Laboratory Analyses | 56 | Blood Glucose (mg/dL) | 3 months after discharge after discharge | 134.93 (134.93–134.93) (n=1) | NA | 87.97 (84.32–126.57) (n=10) | 122.55 (110.00–145.47) (n=7) | H(2)=1.85 | 0,396 | 0,675 |
| Clinical–Nutritional Laboratory Analyses | 57 | Blood Glucose (mg/dL) | 6 months after discharge after discharge | 201.69 (201.69–201.69) (n=1) | NA | 90.53 (77.60–106.64) (n=9) | 117.09 (115.23–124.11) (n=6) | H(2)=4.41 | 0,110 | 0,675 |
| Clinical–Nutritional Laboratory Analyses | 58 | Hemoglobin (g/dL) | Preoperative | 14.28 ± 2.34 (n=4) | NA | 12.54 ± 2.28 (n=11) | 12.35 ± 1.87 (n=8) | F(2, 20)=1.19 | 0,324 | 0,675 |
| Clinical–Nutritional Laboratory Analyses | 59 | Hemoglobin (g/dL) | 3 months after discharge after discharge | 14.10 (14.10–14.10) (n=1) | NA | 12.85 (11.77–13.79) (n=10) | 12.60 (11.65–13.10) (n=7) | H(2)=1.19 | 0,552 | 0,802 |
| Clinical–Nutritional Laboratory Analyses | 60 | Hemoglobin (g/dL) | 6 months after discharge after discharge | 14.60 (14.60–14.60) (n=1) | NA | 12.50 (10.80–14.40) (n=9) | 11.39 (10.50–12.89) (n=6) | H(2)=2.86 | 0,239 | 0,675 |
| Clinical–Nutritional Laboratory Analyses | 61 | Lymphocytes (x10³/mm³) | Preoperative | 2.38 ± 0.69 (n=4) | NA | 2.50 ± 1.47 (n=11) | 1.95 ± 1.15 (n=8) | F(2, 20)=0.44 | 0,648 | 0,816 |
| Clinical–Nutritional Laboratory Analyses | 62 | Lymphocytes (x10³/mm³) | 3 months after discharge after discharge | 3.20 (3.20–3.20) (n=1) | NA | 2.20 (1.92–2.95) (n=10) | 2.30 (1.95–3.00) (n=7) | H(2)=0.79 | 0,675 | 0,816 |
| Clinical–Nutritional Laboratory Analyses | 63 | Lymphocytes (x10³/mm³) | 6 months after discharge after discharge | 2.70 (2.70–2.70) (n=1) | NA | 2.30 (1.90–3.20) (n=9) | 1.73 (1.24–3.01) (n=6) | H(2)=0.61 | 0,738 | 0,836 |
| Clinical–Nutritional Laboratory Analyses | 64 | Serum Iron (µg/dL) | Preoperative | 115.63 ± 36.37 (n=4) | NA | 80.01 ± 29.33 (n=11) | 84.24 ± 49.35 (n=8) | F(2, 20)=1.31 | 0,293 | 0,675 |
| Clinical–Nutritional Laboratory Analyses | 65 | Serum Iron (µg/dL) | 3 months after discharge after discharge | 51.97 (51.97–51.97) (n=1) | NA | 96.88 (60.86–120.69) (n=10) | 75.66 (51.16–129.18) (n=7) | H(2)=0.46 | 0,796 | 0,847 |
| Clinical–Nutritional Laboratory Analyses | 66 | Serum Iron (µg/dL) | 6 months after discharge after discharge | 102.93 (102.93–102.93) (n=1) | NA | 90.54 (59.69–96.32) (n=9) | 80.81 (65.41–131.28) (n=6) | H(2)=0.99 | 0,609 | 0,816 |
| Clinical–Nutritional Laboratory Analyses | 67 | Ferritin (ng/mL) | Preoperative | 209.10 (176.85–289.17) (n=4) | NA | 269.10 (173.95–620.00) (n=11) | 1086.15 (595.40–1520.10) (n=8) | H(2)=3.16 | 0,206 | 0,675 |
| Clinical–Nutritional Laboratory Analyses | 68 | Ferritin (ng/mL) | 3 months after discharge after discharge | 254.60 (254.60–254.60) (n=1) | NA | 202.65 (77.10–362.40) (n=10) | 447.90 (291.60–678.90) (n=7) | H(2)=2.39 | 0,303 | 0,675 |
| Clinical–Nutritional Laboratory Analyses | 69 | Ferritin (ng/mL) | 6 months after discharge after discharge | 128.00 (128.00–128.00) (n=1) | NA | 183.90 (78.70–351.00) (n=9) | 326.62 (200.73–454.66) (n=6) | H(2)=3.56 | 0,169 | 0,675 |
| Clinical–Nutritional Laboratory Analyses | 70 | UIBC (µg/dL) | Preoperative | 314.31 ± 91.47 (n=4) | NA | 303.79 ± 57.51 (n=11) | 259.52 ± 77.10 (n=8) | F(2, 20)=1.19 | 0,325 | 0,675 |
| Clinical–Nutritional Laboratory Analyses | 71 | UIBC (µg/dL) | 3 months after discharge after discharge | 285.00 (285.00–285.00) (n=1) | NA | 307.97 (291.95–327.14) (n=10) | 293.62 (274.23–301.62) (n=7) | H(2)=2.62 | 0,270 | 0,675 |
| Clinical–Nutritional Laboratory Analyses | 72 | UIBC (µg/dL) | 6 months after discharge after discharge | 300.73 (300.73–300.73) (n=1) | NA | 309.55 (292.48–321.65) (n=9) | 280.26 (270.65–294.63) (n=6) | H(2)=1.99 | 0,370 | 0,675 |
| Clinical–Nutritional Laboratory Analyses | 73 | TIBC (µg/dL) | Preoperative | 429.94 ± 93.41 (n=4) | NA | 383.80 ± 78.07 (n=11) | 343.76 ± 88.96 (n=8) | F(2, 20)=1.44 | 0,260 | 0,675 |
| Clinical–Nutritional Laboratory Analyses | 74 | TIBC (µg/dL) | 3 months after discharge after discharge | 336.97 (336.97–336.97) (n=1) | NA | 415.96 (404.15–441.78) (n=10) | 357.27 (329.69–418.88) (n=7) | H(2)=1.21 | 0,545 | 0,802 |
| Clinical–Nutritional Laboratory Analyses | 75 | TIBC (µg/dL) | 6 months after discharge after discharge | 403.66 (403.66–403.66) (n=1) | NA | 409.04 (369.24–419.58) (n=9) | 381.91 (326.04–423.12) (n=6) | H(2)=0.03 | 0,985 | 0,985 |
| Clinical–Nutritional Laboratory Analyses | 76 | Transferrin (mg/dL) | Preoperative | 251.45 ± 73.18 (n=4) | NA | 243.03 ± 46.01 (n=11) | 207.62 ± 61.68 (n=8) | F(2, 20)=1.19 | 0,325 | 0,675 |
| Clinical–Nutritional Laboratory Analyses | 77 | Transferrin (mg/dL) | 3 months after discharge after discharge | 228.00 (228.00–228.00) (n=1) | NA | 246.38 (233.56–261.71) (n=10) | 234.90 (219.38–241.30) (n=7) | H(2)=2.62 | 0,270 | 0,675 |
| Clinical–Nutritional Laboratory Analyses | 78 | Transferrin (mg/dL) | 6 months after discharge after discharge | 240.58 (240.58–240.58) (n=1) | NA | 247.64 (233.98–257.32) (n=9) | 224.21 (216.52–235.70) (n=6) | H(2)=1.99 | 0,370 | 0,675 |
| Clinical–Nutritional Laboratory Analyses | 79 | Transferrin Saturation (%) | Preoperative | 27.48 ± 8.50 (n=4) | NA | 20.40 ± 5.08 (n=11) | 24.15 ± 11.05 (n=8) | F(2, 20)=1.24 | 0,311 | 0,675 |
| Clinical–Nutritional Laboratory Analyses | 80 | Transferrin Saturation (%) | 3 months after discharge after discharge | 15.42 (15.42–15.42) (n=1) | NA | 23.69 (15.92–28.94) (n=10) | 23.74 (15.19–30.71) (n=7) | H(2)=0.46 | 0,796 | 0,847 |
| Clinical–Nutritional Laboratory Analyses | 81 | Transferrin Saturation (%) | 6 months after discharge after discharge | 25.50 (25.50–25.50) (n=1) | NA | 19.36 (16.17–23.62) (n=9) | 21.97 (18.41–32.34) (n=6) | H(2)=1.52 | 0,468 | 0,735 |
| Clinical–Nutritional Laboratory Analyses | 82 | Urea (mg/dL) | Preoperative | 42.85 ± 22.49 (n=4) | NA | 41.38 ± 26.45 (n=11) | 28.89 ± 10.76 (n=8) | F(2, 20)=0.94 | 0,409 | 0,675 |
| Clinical–Nutritional Laboratory Analyses | 83 | Urea (mg/dL) | 3 months after discharge after discharge | 13.91 (13.91–13.91) (n=1) | NA | 35.72 (31.39–48.57) (n=10) | 33.60 (23.94–52.23) (n=7) | H(2)=2.99 | 0,224 | 0,675 |
| Clinical–Nutritional Laboratory Analyses | 84 | Urea (mg/dL) | 6 months after discharge after discharge | 19.62 (19.62–19.62) (n=1) | NA | 29.77 (25.14–39.31) (n=9) | 33.70 (25.26–59.57) (n=6) | H(2)=2.49 | 0,288 | 0,675 |
| Clinical–Nutritional Laboratory Analyses | 85 | Creatinine (mg/dL) | Preoperative | 0.96 (0.88–1.23) (n=4) | NA | 0.87 (0.74–1.16) (n=11) | 0.88 (0.83–1.12) (n=8) | H(2)=0.25 | 0,883 | 0,907 |
| Clinical–Nutritional Laboratory Analyses | 86 | Creatinine (mg/dL) | 3 months after discharge after discharge | 0.69 (0.69–0.69) (n=1) | NA | 0.84 (0.75–0.95) (n=10) | 0.87 (0.78–1.08) (n=7) | H(2)=2.34 | 0,310 | 0,675 |
| Clinical–Nutritional Laboratory Analyses | 87 | Creatinine (mg/dL) | 6 months after discharge after discharge | 0.95 (0.95–0.95) (n=1) | NA | 0.87 (0.73–1.09) (n=9) | 0.84 (0.80–1.78) (n=6) | H(2)=0.23 | 0,893 | 0,907 |
| Clinical–Nutritional Laboratory Analyses | 88 | Aspartate Aminotransferase (AST) (U/L) | Preoperative | 282.54 ± 264.88 (n=4) | NA | 162.33 ± 108.95 (n=11) | 143.25 ± 100.43 (n=8) | F(2, 7.04)=0.49 | 0,632 | 0,816 |
| Clinical–Nutritional Laboratory Analyses | 89 | Aspartate Aminotransferase (AST) (U/L) | 3 months after discharge after discharge | 36.98 (36.98–36.98) (n=1) | NA | 28.45 (24.39–36.54) (n=10) | 25.69 (23.30–35.53) (n=7) | H(2)=0.76 | 0,685 | 0,816 |
| Clinical–Nutritional Laboratory Analyses | 90 | Aspartate Aminotransferase (AST) (U/L) | 6 months after discharge after discharge | 38.81 (38.81–38.81) (n=1) | NA | 31.72 (27.94–37.05) (n=9) | 26.18 (24.00–36.68) (n=6) | H(2)=1.95 | 0,377 | 0,675 |
| Clinical–Nutritional Laboratory Analyses | 91 | Aminotransferase (ALT) (U/L) | Preoperative | 383.34 ± 370.06 (n=4) | NA | 213.68 ± 202.69 (n=11) | 147.62 ± 78.64 (n=8) | F(2, 6.75)=1.10 | 0,387 | 0,675 |
| Clinical–Nutritional Laboratory Analyses | 92 | Aminotransferase (ALT) (U/L) | 3 months after discharge after discharge | 45.37 (45.37–45.37) (n=1) | NA | 31.91 (28.38–45.30) (n=10) | 36.38 (24.48–39.55) (n=7) | H(2)=0.77 | 0,681 | 0,816 |
| Clinical–Nutritional Laboratory Analyses | 93 | Aminotransferase (ALT) (U/L) | 6 months after discharge after discharge | 54.27 (54.27–54.27) (n=1) | NA | 34.21 (31.38–42.54) (n=9) | 28.64 (20.16–38.37) (n=6) | H(2)=2.49 | 0,288 | 0,675 |
| Clinical–Nutritional Laboratory Analyses | 94 | Folic Acid (ng/mL) | Preoperative | 14.98 ± 6.50 (n=4) | NA | 15.58 ± 4.85 (n=11) | 13.41 ± 7.90 (n=8) | F(2, 20)=0.28 | 0,760 | 0,836 |
| Clinical–Nutritional Laboratory Analyses | 95 | Folic Acid (ng/mL) | 3 months after discharge after discharge | 9.10 (9.10–9.10) (n=1) | NA | 20.67 (14.44–24.00) (n=10) | 15.08 (10.23–24.00) (n=7) | H(2)=1.82 | 0,402 | 0,675 |
| Clinical–Nutritional Laboratory Analyses | 96 | Folic Acid (ng/mL) | 6 months after discharge after discharge | 11.66 (11.66–11.66) (n=1) | NA | 21.56 (20.87–24.00) (n=9) | 16.98 (9.85–23.84) (n=6) | H(2)=1.96 | 0,375 | 0,675 |
| Clinical–Nutritional Laboratory Analyses | 97 | Vitamin B12 (pg/mL) | Preoperative | 522.00 (420.75–598.00) (n=4) | NA | 455.00 (370.00–638.50) (n=11) | 720.50 (443.75–1365.50) (n=8) | H(2)=2.62 | 0,269 | 0,675 |
| Clinical–Nutritional Laboratory Analyses | 98 | Vitamin B12 (pg/mL) | 3 months after discharge after discharge | 372.00 (372.00–372.00) (n=1) | NA | 460.00 (353.75–582.25) (n=10) | 585.00 (436.50–1431.50) (n=7) | H(2)=2.12 | 0,347 | 0,675 |
| Clinical–Nutritional Laboratory Analyses | 99 | Vitamin B12 (pg/mL) | 6 months after discharge after discharge | 313.00 (313.00–313.00) (n=1) | NA | 392.00 (316.25–515.00) (n=9) | 462.50 (336.30–903.55) (n=6) | H(2)=1.87 | 0,393 | 0,675 |
| Clinical–Nutritional Laboratory Analyses | 100 | Vitamin A (µg/dL) | Preoperative | 0.30 (0.27–0.32) (n=4) | NA | 0.33 (0.22–0.37) (n=11) | 0.24 (0.17–0.32) (n=8) | H(2)=2.44 | 0,296 | 0,675 |
| Clinical–Nutritional Laboratory Analyses | 101 | Vitamin A (µg/dL) | 3 months after discharge after discharge | 0.32 (0.32–0.32) (n=1) | NA | 0.31 (0.20–0.42) (n=10) | 0.33 (0.29–0.38) (n=7) | H(2)=0.30 | 0,862 | 0,903 |
| Clinical–Nutritional Laboratory Analyses | 102 | Vitamin A (µg/dL) | 6 months after discharge after discharge | 0.21 (0.21–0.21) (n=1) | NA | 0.34 (0.28–0.38) (n=9) | 0.29 (0.28–0.31) (n=6) | H(2)=3.37 | 0,186 | 0,675 |
| Clinical–Nutritional Laboratory Analyses | 103 | Vitamin E (mg/dL) | Preoperative | 7.28 (7.28–7.60) (n=4) | NA | 8.11 (6.81–9.33) (n=11) | 7.37 (5.97–9.46) (n=8) | H(2)=0.57 | 0,752 | 0,836 |
| Clinical–Nutritional Laboratory Analyses | 104 | Vitamin E (mg/dL) | 3 months after discharge after discharge | 8.29 (8.29–8.29) (n=1) | NA | 6.62 (5.92–7.67) (n=10) | 6.00 (4.39–6.74) (n=7) | H(2)=1.85 | 0,396 | 0,675 |
| Clinical–Nutritional Laboratory Analyses | 105 | Vitamin E (mg/dL) | 6 months after discharge after discharge | 5.09 (5.09–5.09) (n=1) | NA | 5.83 (4.88–6.71) (n=9) | 5.16 (4.24–5.91) (n=6) | H(2)=1.01 | 0,602 | 0,816 |
| Clinical–Nutritional Laboratory Analyses | 106 | Copper (µg/dL) | Preoperative | 146.10 ± 95.32 (n=4) | NA | 153.49 ± 52.70 (n=11) | 124.90 ± 18.52 (n=8) | F(2, 20)=0.67 | 0,522 | 0,801 |
| Clinical–Nutritional Laboratory Analyses | 107 | Copper (µg/dL) | 3 months after discharge after discharge | 61.20 (61.20–61.20) (n=1) | NA | 105.10 (91.10–112.35) (n=10) | 106.60 (93.90–117.50) (n=7) | H(2)=2.82 | 0,244 | 0,675 |
| Clinical–Nutritional Laboratory Analyses | 108 | Copper (µg/dL) | 6 months after discharge after discharge | 59.08 (59.08–59.08) (n=1) | NA | 95.00 (92.60–115.30) (n=9) | 92.70 (76.20–108.91) (n=6) | H(2)=3.09 | 0,214 | 0,675 |
| Clinical–Nutritional Laboratory Analyses | 109 | Zinc (µg/dL) | Preoperative | 82.25 (73.12–87.75) (n=4) | NA | 96.00 (85.50–97.25) (n=11) | 61.00 (54.00–90.00) (n=8) | H(2)=7.33 | 0,026 | 0,675 |
| Clinical–Nutritional Laboratory Analyses | 110 | Zinc (µg/dL) | 3 months after discharge after discharge | 121.50 (121.50–121.50) (n=1) | NA | 80.25 (75.00–87.88) (n=10) | 77.00 (72.25–82.00) (n=7) | H(2)=2.57 | 0,276 | 0,675 |
| Clinical–Nutritional Laboratory Analyses | 111 | Zinc (µg/dL) | 6 months after discharge after discharge | 70.50 (70.50–70.50) (n=1) | NA | 86.00 (80.50–89.50) (n=9) | 71.13 (61.25–76.28) (n=6) | H(2)=4.73 | 0,094 | 0,675 |
| Derived inflammatory–nutritional indices | 112 | Prognostic Nutritional Index | Preoperative | 52.00 ± 6.79 (n=4) | N/A | 52.95 ± 11.79 (n=11) | 47.84 ± 7.83 (n=8) | F(2, 20)=0.64 | 0,536 | 0,788 |
| Derived inflammatory–nutritional indices | 113 | Prognostic Nutritional Index | 3 months after discharge | 59.20 (59.20–59.20) (n=1) | N/A | 56.50 (50.65–57.88) (n=10) | 50.90 (47.30–55.45) (n=7) | H(2)=0.94 | 0,626 | 0,791 |
| Derived inflammatory–nutritional indices | 114 | Prognostic Nutritional Index | 6 months after discharge | 58.80 (58.80–58.80) (n=1) | N/A | 58.20 (53.40–60.80) (n=9) | 49.31 (47.65–55.95) (n=6) | H(2)=1.95 | 0,377 | 0,788 |
| Derived inflammatory–nutritional indices | 115 | Glasgow Prognostic Score (GPS) | Preoperative | 0: 2 (50.0%); 1: 1 (25.0%); 2: 1 (25.0%) | N/A | 0: 4 (36.4%); 1: 6 (54.5%); 2: 1 (9.1%) | 0: 5 (62.5%); 1: 2 (25.0%); 2: 1 (12.5%) | H(2)=0.74 | 0,692 | 0,791 |
| Derived inflammatory–nutritional indices | 116 | Glasgow Prognostic Score (GPS) | 3 months after discharge | 0: 1 (100.0%) | N/A | 0: 9 (90.0%); 1: 1 (10.0%) | 0: 5 (71.4%); 1: 2 (28.6%) | H(2)=1.17 | 0,558 | 0,788 |
| Derived inflammatory–nutritional indices | 117 | Glasgow Prognostic Score (GPS) | 6 months after discharge | 0: 1 (100.0%) | N/A | 0: 9 (100.0%) | 0: 5 (83.3%); 2: 1 (16.7%) | H(2)=1.67 | 0,435 | 0,788 |
| Derived inflammatory–nutritional indices | 118 | Albumin/CRP ratio | Preoperative | 0.47 (0.13–3.41) (n=4) | N/A | 0.48 (0.27–1.18) (n=11) | 0.83 (0.40–2.19) (n=8) | H(2)=0.85 | 0,654 | 0,791 |
| Derived inflammatory–nutritional indices | 119 | Albumin/CRP ratio | 3 months after discharge | 10.80 (10.80–10.80) (n=1) | N/A | 5.80 (1.51–10.64) (n=10) | 2.26 (0.77–10.68) (n=7) | H(2)=1.41 | 0,495 | 0,788 |
| Derived inflammatory–nutritional indices | 120 | Albumin/CRP ratio | 6 months after discharge | 11.32 (11.32–11.32) (n=1) | N/A | 6.60 (2.57–10.97) (n=9) | 9.97 (4.21–10.36) (n=6) | H(2)=1.46 | 0,481 | 0,788 |
| Derived inflammatory–nutritional indices | 121 | CRP/Albumin ratio | Preoperative | 4.37 (0.96–7.66) (n=4) | N/A | 2.07 (0.90–3.69) (n=11) | 1.21 (0.46–2.56) (n=8) | H(2)=0.81 | 0,667 | 0,791 |
| Derived inflammatory–nutritional indices | 122 | CRP/Albumin ratio | 3 months after discharge | 925.93 (925.93–925.93) (n=1) | N/A | 1.42 (0.55–858.56) (n=10) | 1.85 (1.30–922.87) (n=7) | H(2)=1.28 | 0,528 | 0,788 |
| Derived inflammatory–nutritional indices | 123 | CRP/Albumin ratio | 6 months after discharge | 883.00 (883.00–883.00) (n=1) | N/A | 0.73 (0.18–811.36) (n=9) | 470.37 (1.85–956.54) (n=6) | H(2)=2.17 | 0,338 | 0,788 |
| Derived inflammatory–nutritional indices | 124 | CRP/Lymphocytes ratio | Preoperative | 6.32 (2.01–12.13) (n=4) | N/A | 2.29 (1.25–9.16) (n=11) | 2.77 (1.97–5.16) (n=8) | H(2)=0.44 | 0,803 | 0,828 |
| Derived inflammatory–nutritional indices | 125 | CRP/Lymphocytes ratio | 3 months after discharge | 0.12 (0.12–0.12) (n=1) | N/A | 0.36 (0.20–1.41) (n=10) | 2.15 (0.53–2.34) (n=7) | H(2)=2.57 | 0,277 | 0,788 |
| Derived inflammatory–nutritional indices | 126 | CRP/Lymphocytes ratio | 6 months after discharge | 0.15 (0.15–0.15) (n=1) | N/A | 0.38 (0.17–0.51) (n=9) | 1.68 (0.22–9.01) (n=6) | H(2)=1.78 | 0,411 | 0,788 |
| Derived inflammatory–nutritional indices | 127 | Ferritin/Albumin ratio | Preoperative | 46.73 (40.92–70.07) (n=4) | N/A | 71.66 (39.99–156.01) (n=11) | 290.89 (149.05–361.19) (n=8) | H(2)=3.25 | 0,197 | 0,788 |
| Derived inflammatory–nutritional indices | 128 | Ferritin/Albumin ratio | 3 months after discharge | 58.94 (58.94–58.94) (n=1) | N/A | 45.42 (17.34–90.53) (n=10) | 111.64 (73.48–173.38) (n=7) | H(2)=2.86 | 0,239 | 0,788 |
| Derived inflammatory–nutritional indices | 129 | Ferritin/Albumin ratio | 6 months after discharge | 28.26 (28.26–28.26) (n=1) | N/A | 38.88 (18.65–72.07) (n=9) | 77.92 (49.50–109.96) (n=6) | H(2)=3.09 | 0,214 | 0,788 |
| Derived inflammatory–nutritional indices | 130 | Ferritin/Lymphocytes ratio | Preoperative | 126.32 ± 117.36 (n=4) | N/A | 238.03 ± 186.19 (n=11) | 786.12 ± 666.72 (n=8) | F(2, 10.47)=3.86 | 0,055 | 0,788 |
| Derived inflammatory–nutritional indices | 131 | Ferritin/Lymphocytes ratio | 3 months after discharge | 79.56 (79.56–79.56) (n=1) | N/A | 77.36 (32.73–144.23) (n=10) | 131.74 (117.51–268.15) (n=7) | H(2)=2.51 | 0,285 | 0,788 |
| Derived inflammatory–nutritional indices | 132 | Ferritin/Lymphocytes ratio | 6 months after discharge | 47.41 (47.41–47.41) (n=1) | N/A | 62.84 (24.59–105.66) (n=9) | 131.44 (110.69–304.18) (n=6) | H(2)=5.01 | 0,082 | 0,788 |
| Derived inflammatory–nutritional indices | 133 | Albumin-Lymphocytes-CRP index | Preoperative | 0.89 (0.31–10.08) (n=4) | N/A | 1.36 (0.46–3.61) (n=11) | 1.45 (0.78–2.39) (n=8) | H(2)=0.38 | 0,828 | 0,828 |
| Derived inflammatory–nutritional indices | 134 | Albumin-Lymphocytes-CRP index | 3 months after discharge | 34.56 (34.56–34.56) (n=1) | N/A | 14.01 (2.92–23.70) (n=10) | 5.20 (1.53–25.79) (n=7) | H(2)=1.17 | 0,558 | 0,788 |
| Derived inflammatory–nutritional indices | 135 | Albumin-Lymphocytes-CRP index | 6 months after discharge | 30.58 (30.58–30.58) (n=1) | N/A | 10.33 (8.23–28.35) (n=9) | 17.40 (4.73–30.82) (n=6) | H(2)=0.58 | 0,748 | 0,816 |
| Notes: Values are presented as mean +/- standard deviation or median (P25-P75), with the valid n shown for each group and comparison. The analysis used observed available data only; no multiple imputation was performed. The number of patients evaluated was preoperative n = 24, 3 months after discharge n = 18, and 6 months after discharge n = 16. Raw p-values correspond to the statistical test reported in each row. BH-FDR adjusted p-values were calculated with the Benjamini-Hochberg false discovery rate procedure within each Exposure x Domain family: Dietary intake (45 tests), Clinical-nutritional laboratory analyses (66 tests), and Derived inflammatory-nutritional indices (24 tests). The BH-FDR audit found 945 inferential comparisons, 43 raw p-values < 0.05, and 0 BH-FDR adjusted p-values < 0.05. For binary exposures, two-sample t-test/Welch t-test or Mann-Whitney/Wilcoxon rank-sum tests were used as reported. For multicategorical or ordinal exposures, one-way ANOVA or Kruskal-Wallis tests were used; these are omnibus tests and do not establish pairwise differences or dose-response trends without post-hoc or contrast testing. Because this was an exploratory supplementary analysis with multiple comparisons, small subgroup sizes, and no covariate adjustment, the results should be interpreted as hypothesis-generating rather than confirmatory. Abbreviations: BH-FDR, Benjamini-Hochberg false discovery rate; EPI, exocrine pancreatic insufficiency; TIBC, total iron-binding capacity; UIBC, unsaturated iron-binding capacity. | | | | | | | | | | |

| **Supplementary Table S11.** Exploratory associations between pancreatic enzyme replacement therapy (PERT) and dietary intake, clinical-nutritional laboratory variables, and inflammatory-nutritional indices after pancreatoduodenectomy. | | | | | | | | |
| --- | --- | --- | --- | --- | --- | --- | --- | --- |
| **Outcome** | | | | **Pancreatic enzyme replacement therapy (PERT)** | | | | |
| **Domain** | **Source row** | **Variable** | **Time** | **Yes** | **No** | **Statistical result** | **Raw p-value** | **BH-FDR adjusted p-value** |
|  |  |  |  | **Mean ± SD or Median (P25–P75), n** | **Mean ± SD or Median (P25–P75), n** |  |  |  |
| Dietary intake | 1 | Energy (kcal) | Preoperative | 2485.70 ± 661.28 (n=10) | 2874.74 ± 464.22 (n=14) | t(22)=-1.70; Δ_mean=-389.04; 95% IC [-864.21 ; 86.12] | 0,104 | 0,391 |
| Dietary intake | 2 | Energy (kcal) | 3 months after discharge | 1863.37 ± 314.44 (n=9) | 2217.18 ± 262.27 (n=9) | t(16)=-2.59; Δ_mean=-353.81; 95% IC [-643.15 ; -64.46] | 0,020 | 0,300 |
| Dietary intake | 3 | Energy (kcal) | 6 months after discharge | 1844.71 ± 242.43 (n=7) | 2137.17 ± 352.45 (n=9) | t(14)=-1.87; Δ_mean=-292.46; 95% IC [-627.66 ; 42.73] | 0,082 | 0,391 |
| Dietary intake | 4 | Protein (g) | Preoperative | 109.66 ± 28.65 (n=10) | 122.67 ± 17.40 (n=14) | t(22)=-1.39; Δ_mean=-13.01; 95% IC [-32.49 ; 6.47] | 0,180 | 0,405 |
| Dietary intake | 5 | Protein (g) | 3 months after discharge | 88.53 ± 10.01 (n=9) | 104.66 ± 13.77 (n=9) | t(16)=-2.84; Δ_mean=-16.12; 95% IC [-28.16 ; -4.09] | 0,012 | 0,270 |
| Dietary intake | 6 | Protein (g) | 6 months after discharge | 90.14 ± 9.38 (n=7) | 109.54 ± 14.34 (n=9) | t(14)=-3.09; Δ_mean=-19.40; 95% IC [-32.87 ; -5.94] | 0,008 | 0,270 |
| Dietary intake | 7 | Relative energy balance (kcal) | Preoperative | 988.64 ± 346.59 (n=10) | 1203.35 ± 278.35 (n=14) | t(22)=-1.68; Δ_mean=-214.70; 95% IC [-479.26 ; 49.85] | 0,107 | 0,391 |
| Dietary intake | 8 | Relative energy balance (kcal) | 3 months after discharge | 470.39 ± 155.14 (n=9) | 643.01 ± 295.27 (n=9) | t(16)=-1.55; Δ_mean=-172.62; 95% IC [-408.32 ; 63.07] | 0,140 | 0,391 |
| Dietary intake | 9 | Relative energy balance (kcal) | 6 months after discharge | 551.72 ± 93.42 (n=7) | 742.10 ± 272.59 (n=9) | t(14)=-1.76; Δ_mean=-190.37; 95% IC [-422.70 ; 41.95] | 0,101 | 0,391 |
| Dietary intake | 10 | Carbohydrate (g) | Preoperative | 350.21 ± 92.97 (n=10) | 393.75 ± 65.09 (n=14) | t(22)=-1.35; Δ_mean=-43.54; 95% IC [-110.27 ; 23.19] | 0,190 | 0,407 |
| Dietary intake | 11 | Carbohydrate (g) | 3 months after discharge | 244.97 ± 39.29 (n=9) | 281.97 ± 32.95 (n=9) | t(16)=-2.16; Δ_mean=-37.00; 95% IC [-73.23 ; -0.76] | 0,046 | 0,345 |
| Dietary intake | 12 | Carbohydrate (g) | 6 months after discharge | 245.99 ± 32.59 (n=7) | 274.52 ± 41.05 (n=9) | t(14)=-1.50; Δ_mean=-28.52; 95% IC [-69.23 ; 12.18] | 0,155 | 0,391 |
| Dietary intake | 13 | Lipid (g) | Preoperative | 67.43 (62.30–82.49) (n=10) | 82.59 (78.40–110.53) (n=14) | W=40.00; Δ_HL=-16.44; 95% IC [-37.96 ; 2.03] | 0,084 | 0,391 |
| Dietary intake | 14 | Lipid (g) | 3 months after discharge | 58.82 ± 15.81 (n=9) | 74.52 ± 13.99 (n=9) | t(16)=-2.23; Δ_mean=-15.70; 95% IC [-30.62 ; -0.78] | 0,040 | 0,345 |
| Dietary intake | 15 | Lipid (g) | 6 months after discharge | 55.58 ± 12.43 (n=7) | 66.77 ± 19.49 (n=9) | t(14)=-1.32; Δ_mean=-11.20; 95% IC [-29.39 ; 6.99] | 0,208 | 0,425 |
| Dietary intake | 16 | Fiber (g) | Preoperative | 46.71 ± 9.45 (n=10) | 45.55 ± 12.80 (n=14) | t(22)=0.24; Δ_mean=1.16; 95% IC [-8.75 ; 11.07] | 0,810 | 0,868 |
| Dietary intake | 17 | Fiber (g) | 3 months after discharge | 36.87 ± 10.29 (n=9) | 39.41 ± 8.39 (n=9) | t(16)=-0.58; Δ_mean=-2.55; 95% IC [-11.93 ; 6.83] | 0,573 | 0,774 |
| Dietary intake | 18 | Fiber (g) | 6 months after discharge | 34.98 ± 7.94 (n=7) | 38.51 ± 9.52 (n=9) | t(14)=-0.79; Δ_mean=-3.53; 95% IC [-13.12 ; 6.06] | 0,443 | 0,736 |
| Dietary intake | 19 | Calcium (mg) | Preoperative | 1398.82 ± 337.52 (n=10) | 1529.96 ± 292.69 (n=14) | t(22)=-1.02; Δ_mean=-131.14; 95% IC [-398.88 ; 136.60] | 0,321 | 0,602 |
| Dietary intake | 20 | Calcium (mg) | 3 months after discharge | 1255.01 ± 369.06 (n=9) | 1365.56 ± 414.29 (n=9) | t(16)=-0.60; Δ_mean=-110.55; 95% IC [-502.61 ; 281.52] | 0,558 | 0,774 |
| Dietary intake | 21 | Calcium (mg) | 6 months after discharge | 1062.21 (1011.21–1246.56) (n=7) | 1272.90 (1025.81–1491.53) (n=9) | W=25.00; Δ_HL=-195.90; 95% IC [-494.95 ; 209.85] | 0,525 | 0,774 |
| Dietary intake | 22 | Iron (mg) | Preoperative | 26.11 ± 7.12 (n=10) | 27.77 ± 6.48 (n=14) | t(22)=-0.60; Δ_mean=-1.67; 95% IC [-7.46 ; 4.13] | 0,557 | 0,774 |
| Dietary intake | 23 | Iron (mg) | 3 months after discharge | 19.92 (17.75–28.23) (n=9) | 24.65 (19.73–26.23) (n=9) | W=36.00; Δ_HL=-1.98; 95% IC [-8.46 ; 9.44] | 0,724 | 0,795 |
| Dietary intake | 24 | Iron (mg) | 6 months after discharge | 20.71 (17.34–23.45) (n=7) | 23.82 (18.72–26.01) (n=9) | W=27.00; Δ_HL=-1.72; 95% IC [-8.03 ; 6.63] | 0,672 | 0,775 |
| Dietary intake | 25 | Potassium (mg) | Preoperative | 6322.39 ± 1215.74 (n=10) | 6965.31 ± 1360.88 (n=14) | t(22)=-1.19; Δ_mean=-642.92; 95% IC [-1762.15 ; 476.32] | 0,246 | 0,481 |
| Dietary intake | 26 | Potassium (mg) | 3 months after discharge | 5133.78 (4564.53–5584.76) (n=9) | 5444.64 (5039.31–6898.60) (n=9) | W=35.00; Δ_HL=-310.86; 95% IC [-1979.03 ; 841.36] | 0,659 | 0,775 |
| Dietary intake | 27 | Potassium (mg) | 6 months after discharge | 5423.58 ± 1137.05 (n=7) | 5662.31 ± 1233.47 (n=9) | t(14)=-0.40; Δ_mean=-238.73; 95% IC [-1528.32 ; 1050.85] | 0,697 | 0,784 |
| Dietary intake | 28 | Selenium (µg) | Preoperative | 47.75 ± 13.30 (n=10) | 64.45 ± 20.47 (n=14) | t(22)=-2.26; Δ_mean=-16.70; 95% IC [-32.06 ; -1.34] | 0,034 | 0,345 |
| Dietary intake | 29 | Selenium (µg) | 3 months after discharge | 40.38 ± 21.48 (n=9) | 55.82 ± 17.99 (n=9) | t(16)=-1.65; Δ_mean=-15.44; 95% IC [-35.24 ; 4.36] | 0,118 | 0,391 |
| Dietary intake | 30 | Selenium (µg) | 6 months after discharge | 39.40 ± 21.58 (n=7) | 53.46 ± 16.92 (n=9) | t(14)=-1.46; Δ_mean=-14.05; 95% IC [-34.66 ; 6.55] | 0,165 | 0,391 |
| Dietary intake | 31 | Zinc (mg) | Preoperative | 16.14 (13.33–17.50) (n=10) | 18.06 (17.01–21.09) (n=14) | W=42.00; Δ_HL=-2.39; 95% IC [-7.19 ; 0.35]0.456 | 0,107 | 0,391 |
| Dietary intake | 32 | Zinc (mg) | 3 months after discharge | 12.12 (10.97–13.36) (n=9) | 15.31 (13.77–16.79) (n=9) | W=23.00; Δ_HL=-2.66; 95% IC [-6.19 ; 2.67] | 0,133 | 0,391 |
| Dietary intake | 33 | Zinc (mg) | 6 months after discharge | 12.40 ± 2.81 (n=7) | 14.88 ± 3.67 (n=9) | t(14)=-1.48; Δ_mean=-2.48; 95% IC [-6.07 ; 1.12] | 0,161 | 0,391 |
| Dietary intake | 34 | Sodium (mg) | Preoperative | 1540.41 (1198.25–1901.48) (n=10) | 2075.03 (1320.09–2726.62) (n=14) | W=45.00; Δ_HL=-415.39; 95% IC [-1222.92 ; 187.37] | 0,151 | 0,391 |
| Dietary intake | 35 | Sodium (mg) | 3 months after discharge | 1288.41 ± 368.46 (n=9) | 1447.12 ± 479.34 (n=9) | t(16)=-0.79; Δ_mean=-158.71; 95% IC [-585.94 ; 268.52] | 0,442 | 0,736 |
| Dietary intake | 36 | Sodium (mg) | 6 months after discharge | 1224.90 ± 351.96 (n=7) | 1395.28 ± 491.27 (n=9) | t(14)=-0.77; Δ_mean=-170.38; 95% IC [-642.76 ; 301.99] | 0,452 | 0,736 |
| Dietary intake | 37 | Vitamin A (mg) | Preoperative | 5.20 ± 2.76 (n=10) | 5.83 ± 2.97 (n=14) | t(22)=-0.52; Δ_mean=-0.62; 95% IC [-3.10 ; 1.85] | 0,606 | 0,774 |
| Dietary intake | 38 | Vitamin A (mg) | 3 months after discharge | 3.92 (2.89–4.56) (n=9) | 4.88 (3.61–7.34) (n=9) | W=32.00; Δ_HL=-0.80; 95% IC [-4.05 ; 2.75] | 0,480 | 0,745 |
| Dietary intake | 39 | Vitamin A (mg) | 6 months after discharge | 3.94 (2.71–4.65) (n=7) | 4.75 (3.41–6.66) (n=9) | W=24.00; Δ_HL=-0.85; 95% IC [-4.11 ; 1.76] | 0,458 | 0,736 |
| Dietary intake | 40 | Vitamin C (mg) | Preoperative | 292.62 (235.76–493.96) (n=10) | 373.09 (200.51–534.79) (n=14) | W=69.00; Δ_HL=-4.00; 95% IC [-199.65 ; 137.14] | 0,977 | 0,999 |
| Dietary intake | 41 | Vitamin C (mg) | 3 months after discharge | 273.36 (210.21–349.88) (n=9) | 337.47 (249.87–504.52) (n=9) | W=39.00; Δ_HL=-15.45; 95% IC [-274.66 ; 148.42] | 0,930 | 0,973 |
| Dietary intake | 42 | Vitamin C (mg) | 6 months after discharge | 321.33 ± 155.89 (n=7) | 363.37 ± 184.78 (n=9) | t(14)=-0.48; Δ_mean=-42.04; 95% IC [-229.02 ; 144.95] | 0,637 | 0,775 |
| Dietary intake | 43 | Vitamin E (mg) | Preoperative | 4.36 ± 1.65 (n=10) | 4.72 ± 1.75 (n=14) | t(22)=-0.50; Δ_mean=-0.36; 95% IC [-1.82 ; 1.11] | 0,619 | 0,774 |
| Dietary intake | 44 | Vitamin E (mg) | 3 months after discharge | 3.44 (2.66–4.62) (n=9) | 4.96 (3.36–6.13) (n=9) | W=34.00; Δ_HL=-0.70; 95% IC [-3.01 ; 1.55] | 0,596 | 0,774 |
| Dietary intake | 45 | Vitamin E (mg) | 6 months after discharge | 3.32 (2.52–4.24) (n=7) | 3.88 (2.23–5.82) (n=9) | W=31.00; Δ_HL=-0.13; 95% IC [-2.60 ; 1.94] | 1,000 | 1,000 |
| Clinical–Nutritional Laboratory Analyses | 46 | Total Proteins (g/dL) | Preoperative | 6.95 ± 0.63 (n=10) | 6.88 ± 0.53 (n=14) | t(22)=0.25; Δ_mean=0.06; 95% IC [-0.43 ; 0.55] | 0,802 | 0,898 |
| Clinical–Nutritional Laboratory Analyses | 47 | Total Proteins (g/dL) | 3 months after discharge after discharge | 6.68 ± 0.45 (n=9) | 7.08 ± 0.43 (n=9) | t(16)=-1.95; Δ_mean=-0.41; 95% IC [-0.85 ; 0.03] | 0,068 | 0,348 |
| Clinical–Nutritional Laboratory Analyses | 48 | Total Proteins (g/dL) | 6 months after discharge after discharge | 6.36 ± 0.37 (n=7) | 6.88 ± 0.49 (n=9) | t(14)=-2.34; Δ_mean=-0.53; 95% IC [-1.01 ; -0.04] | 0,035 | 0,289 |
| Clinical–Nutritional Laboratory Analyses | 49 | Albumin (g/dL) | Preoperative | 4.01 ± 0.64 (n=10) | 3.91 ± 0.61 (n=14) | t(22)=0.37; Δ_mean=0.10; 95% IC [-0.44 ; 0.63] | 0,712 | 0,898 |
| Clinical–Nutritional Laboratory Analyses | 50 | Albumin (g/dL) | 3 months after discharge after discharge | 3.91 ± 0.49 (n=9) | 4.30 ± 0.50 (n=9) | t(16)=-1.68; Δ_mean=-0.39; 95% IC [-0.89 ; 0.10] | 0,113 | 0,393 |
| Clinical–Nutritional Laboratory Analyses | 51 | Albumin (g/dL) | 6 months after discharge after discharge | 3.85 ± 0.84 (n=7) | 4.43 ± 0.37 (n=9) | t(14)=-1.86; Δ_mean=-0.58; 95% IC [-1.24 ; 0.09] | 0,084 | 0,348 |
| Clinical–Nutritional Laboratory Analyses | 52 | C-Reactive Protein (mg/L) | Preoperative | 3.41 (1.67–10.65) (n=10) | 9.15 (4.87–17.77) (n=14) | W=51.00; Δ_HL=-3.69; 95% IC [-12.76 ; 2.14] | 0,279 | 0,658 |
| Clinical–Nutritional Laboratory Analyses | 53 | C-Reactive Protein (mg/L) | 3 months after discharge after discharge | 3.44 (0.78–4.64) (n=9) | 0.40 (0.40–0.76) (n=9) | W=60.00; Δ_HL=1.36; 95% IC [-0.00 ; 4.24] | 0,079 | 0,348 |
| Clinical–Nutritional Laboratory Analyses | 54 | C-Reactive Protein (mg/L) | 6 months after discharge after discharge | 1.64 (0.66–2.64) (n=7) | 0.40 (0.40–0.40) (n=9) | W=53.00; Δ_HL=1.24; 95% IC [0.00 ; 3.04] | 0,014 | 0,251 |
| Clinical–Nutritional Laboratory Analyses | 55 | Blood Glucose (mg/dL) | Preoperative | 112.00 (101.19–159.50) (n=10) | 127.76 (90.04–153.53) (n=14) | W=78.50; Δ_HL=7.68; 95% IC [-35.13 ; 57.87] | 0,639 | 0,897 |
| Clinical–Nutritional Laboratory Analyses | 56 | Blood Glucose (mg/dL) | 3 months after discharge after discharge | 91.13 (84.26–128.23) (n=9) | 122.55 (118.00–134.93) (n=9) | W=28.00; Δ_HL=-20.55; 95% IC [-49.44 ; 22.07] | 0,289 | 0,658 |
| Clinical–Nutritional Laboratory Analyses | 57 | Blood Glucose (mg/dL) | 6 months after discharge after discharge | 90.96 ± 20.09 (n=7) | 119.38 ± 36.42 (n=9) | t(14)=-1.85; Δ_mean=-28.42; 95% IC [-61.40 ; 4.56] | 0,086 | 0,348 |
| Clinical–Nutritional Laboratory Analyses | 58 | Hemoglobin (g/dL) | Preoperative | 11.81 ± 1.76 (n=10) | 13.20 ± 2.44 (n=14) | t(22)=-1.53; Δ_mean=-1.39; 95% IC [-3.27 ; 0.49] | 0,140 | 0,462 |
| Clinical–Nutritional Laboratory Analyses | 59 | Hemoglobin (g/dL) | 3 months after discharge after discharge | 12.06 ± 1.84 (n=9) | 13.07 ± 1.25 (n=9) | t(16)=-1.37; Δ_mean=-1.02; 95% IC [-2.59 ; 0.55] | 0,189 | 0,567 |
| Clinical–Nutritional Laboratory Analyses | 60 | Hemoglobin (g/dL) | 6 months after discharge after discharge | 10.81 ± 2.04 (n=7) | 13.40 ± 1.57 (n=9) | t(14)=-2.88; Δ_mean=-2.59; 95% IC [-4.52 ; -0.66] | 0,012 | 0,251 |
| Clinical–Nutritional Laboratory Analyses | 61 | Lymphocytes (x10³/mm³) | Preoperative | 2.49 ± 1.49 (n=10) | 2.05 ± 1.05 (n=14) | t(22)=0.85; Δ_mean=0.44; 95% IC [-0.63 ; 1.51] | 0,404 | 0,726 |
| Clinical–Nutritional Laboratory Analyses | 62 | Lymphocytes (x10³/mm³) | 3 months after discharge after discharge | 2.44 ± 0.68 (n=9) | 2.54 ± 1.17 (n=9) | t(16)=-0.22; Δ_mean=-0.10; 95% IC [-1.06 ; 0.86] | 0,827 | 0,910 |
| Clinical–Nutritional Laboratory Analyses | 63 | Lymphocytes (x10³/mm³) | 6 months after discharge after discharge | 2.17 ± 1.18 (n=7) | 2.59 ± 0.93 (n=9) | t(14)=-0.79; Δ_mean=-0.42; 95% IC [-1.55 ; 0.71] | 0,440 | 0,726 |
| Clinical–Nutritional Laboratory Analyses | 64 | Serum Iron (µg/dL) | Preoperative | 81.54 ± 39.98 (n=10) | 91.59 ± 37.78 (n=14) | t(22)=-0.63; Δ_mean=-10.05; 95% IC [-43.28 ; 23.18] | 0,537 | 0,844 |
| Clinical–Nutritional Laboratory Analyses | 65 | Serum Iron (µg/dL) | 3 months after discharge after discharge | 65.10 ± 34.88 (n=9) | 126.84 ± 55.51 (n=9) | t(16)=-2.83; Δ_mean=-61.75; 95% IC [-108.08 ; -15.42] | 0,012 | 0,251 |
| Clinical–Nutritional Laboratory Analyses | 66 | Serum Iron (µg/dL) | 6 months after discharge after discharge | 73.58 ± 35.00 (n=7) | 100.34 ± 45.63 (n=9) | t(14)=-1.28; Δ_mean=-26.77; 95% IC [-71.52 ; 17.99] | 0,220 | 0,631 |
| Clinical–Nutritional Laboratory Analyses | 67 | Ferritin (ng/mL) | Preoperative | 620.00 (123.50–825.42) (n=10) | 259.60 (125.62–750.20) (n=14) | W=75.00; Δ_HL=4.19; 95% IC [-448.10 ; 561.20] | 0,792 | 0,898 |
| Clinical–Nutritional Laboratory Analyses | 68 | Ferritin (ng/mL) | 3 months after discharge after discharge | 448.40 ± 414.65 (n=9) | 275.52 ± 168.06 (n=9) | t(16)=1.16; Δ_mean=172.88; 95% IC [-143.28 ; 489.03] | 0,263 | 0,648 |
| Clinical–Nutritional Laboratory Analyses | 69 | Ferritin (ng/mL) | 6 months after discharge after discharge | 314.33 ± 241.10 (n=7) | 234.96 ± 158.59 (n=9) | t(14)=0.79; Δ_mean=79.37; 95% IC [-134.87 ; 293.60] | 0,440 | 0,726 |
| Clinical–Nutritional Laboratory Analyses | 70 | UIBC (µg/dL) | Preoperative | 283.29 ± 54.21 (n=10) | 290.84 ± 82.47 (n=14) | t(22)=-0.25; Δ_mean=-7.55; 95% IC [-69.60 ; 54.50] | 0,803 | 0,898 |
| Clinical–Nutritional Laboratory Analyses | 71 | UIBC (µg/dL) | 3 months after discharge after discharge | 288.72 (243.09–308.35) (n=9) | 301.64 (293.62–307.59) (n=9) | W=29.00; Δ_HL=-24.48; 95% IC [-85.88 ; 16.23] | 0,331 | 0,685 |
| Clinical–Nutritional Laboratory Analyses | 72 | UIBC (µg/dL) | 6 months after discharge after discharge | 281.89 ± 29.61 (n=7) | 316.46 ± 41.14 (n=9) | t(14)=-1.87; Δ_mean=-34.56; 95% IC [-74.17 ; 5.05] | 0,082 | 0,348 |
| Clinical–Nutritional Laboratory Analyses | 73 | TIBC (µg/dL) | Preoperative | 364.83 ± 66.82 (n=10) | 382.43 ± 98.22 (n=14) | t(22)=-0.49; Δ_mean=-17.60; 95% IC [-92.09 ; 56.90] | 0,629 | 0,897 |
| Clinical–Nutritional Laboratory Analyses | 74 | TIBC (µg/dL) | 3 months after discharge after discharge | 348.49 ± 77.69 (n=9) | 440.62 ± 85.23 (n=9) | t(16)=-2.40; Δ_mean=-92.13; 95% IC [-173.63 ; -10.63] | 0,029 | 0,273 |
| Clinical–Nutritional Laboratory Analyses | 75 | TIBC (µg/dL) | 6 months after discharge after discharge | 355.47 ± 53.64 (n=7) | 416.80 ± 55.27 (n=9) | t(14)=-2.23; Δ_mean=-61.33; 95% IC [-120.32 ; -2.34] | 0,043 | 0,315 |
| Clinical–Nutritional Laboratory Analyses | 76 | Transferrin (mg/dL) | Preoperative | 226.63 ± 43.37 (n=10) | 232.67 ± 65.98 (n=14) | t(22)=-0.25; Δ_mean=-6.04; 95% IC [-55.68 ; 43.60] | 0,803 | 0,898 |
| Clinical–Nutritional Laboratory Analyses | 77 | Transferrin (mg/dL) | 3 months after discharge after discharge | 230.98 (194.47–246.68) (n=9) | 241.31 (234.90–246.07) (n=9) | W=29.00; Δ_HL=-19.58; 95% IC [-68.70 ; 12.98] | 0,331 | 0,685 |
| Clinical–Nutritional Laboratory Analyses | 78 | Transferrin (mg/dL) | 6 months after discharge after discharge | 225.51 ± 23.69 (n=7) | 253.17 ± 32.91 (n=9) | t(14)=-1.87; Δ_mean=-27.65; 95% IC [-59.34 ; 4.04] | 0,082 | 0,348 |
| Clinical–Nutritional Laboratory Analyses | 79 | Transferrin Saturation (%) | Preoperative | 21.93 ± 8.44 (n=10) | 23.88 ± 8.04 (n=14) | t(22)=-0.58; Δ_mean=-1.96; 95% IC [-9.00 ; 5.09] | 0,571 | 0,857 |
| Clinical–Nutritional Laboratory Analyses | 80 | Transferrin Saturation (%) | 3 months after discharge after discharge | 18.07 ± 7.22 (n=9) | 27.76 ± 8.45 (n=9) | t(16)=-2.62; Δ_mean=-9.69; 95% IC [-17.54 ; -1.84] | 0,019 | 0,251 |
| Clinical–Nutritional Laboratory Analyses | 81 | Transferrin Saturation (%) | 6 months after discharge after discharge | 19.99 ± 7.54 (n=7) | 23.53 ± 8.77 (n=9) | t(14)=-0.85; Δ_mean=-3.54; 95% IC [-12.48 ; 5.39] | 0,409 | 0,726 |
| Clinical–Nutritional Laboratory Analyses | 82 | Urea (mg/dL) | Preoperative | 34.36 (22.32–43.17) (n=10) | 34.72 (27.55–38.20) (n=14) | W=68.00; Δ_HL=-1.84; 95% IC [-14.75 ; 14.44] | 0,930 | 0,959 |
| Clinical–Nutritional Laboratory Analyses | 83 | Urea (mg/dL) | 3 months after discharge after discharge | 38.86 ± 15.81 (n=9) | 36.35 ± 16.28 (n=9) | t(16)=0.33; Δ_mean=2.51; 95% IC [-13.53 ; 18.55] | 0,744 | 0,898 |
| Clinical–Nutritional Laboratory Analyses | 84 | Urea (mg/dL) | 6 months after discharge after discharge | 34.67 (23.22–43.92) (n=7) | 29.77 (22.77–33.25) (n=9) | W=36.00; Δ_HL=2.72; 95% IC [-12.30 ; 23.40] | 0,672 | 0,898 |
| Clinical–Nutritional Laboratory Analyses | 85 | Creatinine (mg/dL) | Preoperative | 0.86 (0.77–1.11) (n=10) | 0.96 (0.82–1.18) (n=14) | W=64.50; Δ_HL=-0.06; 95% IC [-0.33 ; 0.26] | 0,770 | 0,898 |
| Clinical–Nutritional Laboratory Analyses | 86 | Creatinine (mg/dL) | 3 months after discharge after discharge | 0.92 (0.73–1.14) (n=9) | 0.82 (0.77–0.87) (n=9) | W=42.00; Δ_HL=0.04; 95% IC [-0.14 ; 0.30] | 0,930 | 0,959 |
| Clinical–Nutritional Laboratory Analyses | 87 | Creatinine (mg/dL) | 6 months after discharge after discharge | 0.86 (0.77–1.13) (n=7) | 0.87 (0.79–0.97) (n=9) | W=32.00; Δ_HL=0.00; 95% IC [-0.23 ; 0.37] | 1,000 | 1,000 |
| Clinical–Nutritional Laboratory Analyses | 88 | Aspartate Aminotransferase (AST) (U/L) | Preoperative | 174.92 (137.75–287.81) (n=10) | 107.13 (44.84–157.38) (n=14) | W=94.00; Δ_HL=67.50; 95% IC [-29.02 ; 159.23] | 0,169 | 0,531 |
| Clinical–Nutritional Laboratory Analyses | 89 | Aspartate Aminotransferase (AST) (U/L) | 3 months after discharge after discharge | 26.33 (22.31–37.64) (n=9) | 29.42 (25.69–35.43) (n=9) | W=40.00; Δ_HL=-0.07; 95% IC [-11.69 ; 23.32] | 1,000 | 1,000 |
| Clinical–Nutritional Laboratory Analyses | 90 | Aspartate Aminotransferase (AST) (U/L) | 6 months after discharge after discharge | 36.51 ± 16.88 (n=7) | 31.72 ± 6.05 (n=9) | t(14)=0.79; Δ_mean=4.79; 95% IC [-8.14 ; 17.71] | 0,440 | 0,726 |
| Clinical–Nutritional Laboratory Analyses | 91 | Aminotransferase (ALT) (U/L) | Preoperative | 204.42 (145.58–302.94) (n=10) | 89.50 (54.47–197.25) (n=14) | W=99.00; Δ_HL=90.12; 95% IC [-39.91 ; 205.05] | 0,095 | 0,348 |
| Clinical–Nutritional Laboratory Analyses | 92 | Aminotransferase (ALT) (U/L) | 3 months after discharge after discharge | 38.65 ± 19.34 (n=9) | 32.77 ± 9.79 (n=9) | t(16)=0.81; Δ_mean=5.88; 95% IC [-9.44 ; 21.19] | 0,428 | 0,726 |
| Clinical–Nutritional Laboratory Analyses | 93 | Aminotransferase (ALT) (U/L) | 6 months after discharge after discharge | 39.22 ± 23.94 (n=7) | 35.92 ± 11.50 (n=9) | t(14)=0.36; Δ_mean=3.29; 95% IC [-16.08 ; 22.66] | 0,721 | 0,898 |
| Clinical–Nutritional Laboratory Analyses | 94 | Folic Acid (ng/mL) | Preoperative | 15.21 ± 7.00 (n=10) | 14.07 ± 5.47 (n=14) | t(22)=0.45; Δ_mean=1.14; 95% IC [-4.14 ; 6.41] | 0,659 | 0,898 |
| Clinical–Nutritional Laboratory Analyses | 95 | Folic Acid (ng/mL) | 3 months after discharge after discharge | 24.00 (10.62–24.00) (n=9) | 14.74 (10.87–22.61) (n=9) | W=50.00; Δ_HL=1.39; 95% IC [-4.74 ; 9.66] | 0,413 | 0,726 |
| Clinical–Nutritional Laboratory Analyses | 96 | Folic Acid (ng/mL) | 6 months after discharge after discharge | 24.00 (19.38–24.00) (n=7) | 21.47 (11.66–23.38) (n=9) | W=43.00; Δ_HL=2.44; 95% IC [-2.79 ; 12.34] | 0,237 | 0,644 |
| Clinical–Nutritional Laboratory Analyses | 97 | Vitamin B12 (pg/mL) | Preoperative | 607.00 (411.25–688.00) (n=10) | 455.00 (421.25–616.00) (n=14) | W=79.50; Δ_HL=84.00; 95% IC [-137.00 ; 264.00] | 0,597 | 0,876 |
| Clinical–Nutritional Laboratory Analyses | 98 | Vitamin B12 (pg/mL) | 3 months after discharge after discharge | 538.00 (425.00–597.00) (n=9) | 416.00 (344.00–761.00) (n=9) | W=51.00; Δ_HL=81.00; 95% IC [-336.00 ; 300.00] | 0,377 | 0,726 |
| Clinical–Nutritional Laboratory Analyses | 99 | Vitamin B12 (pg/mL) | 6 months after discharge after discharge | 510.94 (366.00–550.00) (n=7) | 335.06 (313.00–531.00) (n=9) | W=38.00; Δ_HL=46.00; 95% IC [-424.73 ; 216.94] | 0,525 | 0,844 |
| Clinical–Nutritional Laboratory Analyses | 100 | Vitamin A (µg/dL) | Preoperative | 0.30 (0.20–0.33) (n=10) | 0.30 (0.20–0.36) (n=14) | W=64.50; Δ_HL=-0.00; 95% IC [-0.11 ; 0.09] | 0,768 | 0,898 |
| Clinical–Nutritional Laboratory Analyses | 101 | Vitamin A (µg/dL) | 3 months after discharge after discharge | 0.27 ± 0.10 (n=9) | 0.38 ± 0.09 (n=9) | t(16)=-2.48; Δ_mean=-0.11; 95% IC [-0.21 ; -0.02] | 0,025 | 0,273 |
| Clinical–Nutritional Laboratory Analyses | 102 | Vitamin A (µg/dL) | 6 months after discharge after discharge | 0.29 (0.27–0.35) (n=7) | 0.32 (0.28–0.38) (n=9) | W=30.00; Δ_HL=-0.00; 95% IC [-0.10 ; 0.08] | 0,915 | 0,959 |
| Clinical–Nutritional Laboratory Analyses | 103 | Vitamin E (mg/dL) | Preoperative | 7.37 (5.84–8.11) (n=10) | 7.79 (7.28–8.69) (n=14) | W=53.00; Δ_HL=-0.53; 95% IC [-2.59 ; 0.83] | 0,332 | 0,685 |
| Clinical–Nutritional Laboratory Analyses | 104 | Vitamin E (mg/dL) | 3 months after discharge after discharge | 6.33 ± 2.12 (n=9) | 6.96 ± 2.40 (n=9) | t(16)=-0.59; Δ_mean=-0.63; 95% IC [-2.89 ; 1.63] | 0,563 | 0,857 |
| Clinical–Nutritional Laboratory Analyses | 105 | Vitamin E (mg/dL) | 6 months after discharge after discharge | 6.00 (5.73–6.62) (n=7) | 4.88 (4.68–5.43) (n=9) | W=43.00; Δ_HL=1.09; 95% IC [-2.30 ; 2.24] | 0,244 | 0,644 |
| Clinical–Nutritional Laboratory Analyses | 106 | Copper (µg/dL) | Preoperative | 147.70 (127.25–160.70) (n=10) | 137.00 (99.20–149.55) (n=14) | W=89.50; Δ_HL=12.41; 95% IC [-14.40 ; 47.00] | 0,265 | 0,648 |
| Clinical–Nutritional Laboratory Analyses | 107 | Copper (µg/dL) | 3 months after discharge after discharge | 119.13 ± 39.44 (n=9) | 92.76 ± 19.16 (n=9) | t(16)=1.80; Δ_mean=26.38; 95% IC [-4.60 ; 57.36] | 0,090 | 0,348 |
| Clinical–Nutritional Laboratory Analyses | 108 | Copper (µg/dL) | 6 months after discharge after discharge | 120.14 ± 30.27 (n=7) | 87.01 ± 19.85 (n=9) | t(14)=2.64; Δ_mean=33.13; 95% IC [6.27 ; 60.00] | 0,019 | 0,251 |
| Clinical–Nutritional Laboratory Analyses | 109 | Zinc (µg/dL) | Preoperative | 93.00 (90.00–96.75) (n=10) | 72.75 (56.00–89.88) (n=14) | W=102.00; Δ_HL=13.50; 95% IC [-0.00 ; 36.00] | 0,064 | 0,348 |
| Clinical–Nutritional Laboratory Analyses | 110 | Zinc (µg/dL) | 3 months after discharge after discharge | 84.00 (76.50–86.00) (n=9) | 77.00 (71.00–92.00) (n=9) | W=44.00; Δ_HL=3.50; 95% IC [-16.00 ; 14.00] | 0,791 | 0,898 |
| Clinical–Nutritional Laboratory Analyses | 111 | Zinc (µg/dL) | 6 months after discharge after discharge | 78.93 ± 11.56 (n=7) | 77.53 ± 17.01 (n=9) | t(14)=0.19; Δ_mean=1.40; 95% IC [-14.73 ; 17.53] | 0,855 | 0,925 |
| Derived inflammatory–nutritional indices | 112 | Prognostic Nutritional Index | Preoperative | 52.53 ± 8.92 (n=10) | 49.36 ± 10.26 (n=14) | t(22)=0.79; Δ_mean=3.17; 95% IC [-5.19 ; 11.52] | 0,440 | 0,621 |
| Derived inflammatory–nutritional indices | 113 | Prognostic Nutritional Index | 3 months after discharge | 51.29 ± 6.84 (n=9) | 55.71 ± 7.77 (n=9) | t(16)=-1.28; Δ_mean=-4.42; 95% IC [-11.74 ; 2.89] | 0,218 | 0,452 |
| Derived inflammatory–nutritional indices | 114 | Prognostic Nutritional Index | 6 months after discharge | 49.37 ± 11.42 (n=7) | 57.23 ± 5.40 (n=9) | t(14)=-1.83; Δ_mean=-7.86; 95% IC [-17.06 ; 1.35] | 0,089 | 0,405 |
| Derived inflammatory–nutritional indices | 115 | Glasgow Prognostic Score (GPS) | Preoperative | 0: 4 (40.0%); 1: 6 (60.0%) | 0: 7 (50.0%); 1: 4 (28.6%); 2: 3 (21.4%) | W=68.00; Δ_HL=-0.00; 95% IC [-1.00 ; 1.00] | 0,923 | 0,923 |
| Derived inflammatory–nutritional indices | 116 | Glasgow Prognostic Score (GPS) | 3 months after discharge | 0: 7 (77.8%); 1: 2 (22.2%) | 0: 8 (88.9%); 1: 1 (11.1%) | W=45.00; Δ_HL=0.00; 95% IC [-0.00 ; 0.00] | 0,585 | 0,715 |
| Derived inflammatory–nutritional indices | 117 | Glasgow Prognostic Score (GPS) | 6 months after discharge | 0: 6 (85.7%); 2: 1 (14.3%) | 0: 9 (100.0%) | W=36.00; Δ_HL=0.00; 95% IC [0.00 ; 0.00] | 0,313 | 0,470 |
| Derived inflammatory–nutritional indices | 118 | Albumin/CRP ratio | Preoperative | 1.14 (0.41–2.38) (n=10) | 0.37 (0.23–0.84) (n=14) | W=90.00; Δ_HL=0.35; 95% IC [-0.29 ; 1.85] | 0,254 | 0,456 |
| Derived inflammatory–nutritional indices | 119 | Albumin/CRP ratio | 3 months after discharge | 1.33 (0.81–5.22) (n=9) | 10.70 (6.38–10.97) (n=9) | W=16.50; Δ_HL=-5.66; 95% IC [-10.02 ; -0.10] | 0,038 | 0,228 |
| Derived inflammatory–nutritional indices | 120 | Albumin/CRP ratio | 6 months after discharge | 2.57 (1.89–6.02) (n=7) | 10.70 (10.32–11.32) (n=9) | W=6.00; Δ_HL=-7.05; 95% IC [-9.60 ; -2.20] | 0,008 | 0,180 |
| Derived inflammatory–nutritional indices | 121 | CRP/Albumin ratio | Preoperative | 0.96 (0.42–2.45) (n=10) | 2.81 (1.19–4.27) (n=14) | W=50.50; Δ_HL=-0.94; 95% IC [-3.21 ; 0.58] | 0,266 | 0,456 |
| Derived inflammatory–nutritional indices | 122 | CRP/Albumin ratio | 3 months after discharge | 1.24 (0.75–1.59) (n=9) | 865.80 (1.85–925.93) (n=9) | W=28.50; Δ_HL=-835.43; 95% IC [-924.72 ; 1.23] | 0,310 | 0,470 |
| Derived inflammatory–nutritional indices | 123 | CRP/Albumin ratio | 6 months after discharge | 0.39 (0.17–0.57) (n=7) | 883.00 (811.36–934.58) (n=9) | W=8.00; Δ_HL=-882.61; 95% IC [-962.36 ; -0.66] | 0,015 | 0,180 |
| Derived inflammatory–nutritional indices | 124 | CRP/Lymphocytes ratio | Preoperative | 1.81 (1.01–5.54) (n=10) | 5.78 (2.63–9.92) (n=14) | W=44.00; Δ_HL=-2.24; 95% IC [-8.41 ; 0.62] | 0,135 | 0,405 |
| Derived inflammatory–nutritional indices | 125 | CRP/Lymphocytes ratio | 3 months after discharge | 1.33 (0.46–2.15) (n=9) | 0.25 (0.20–2.27) (n=9) | W=47.00; Δ_HL=0.26; 95% IC [-1.47 ; 1.87] | 0,596 | 0,715 |
| Derived inflammatory–nutritional indices | 126 | CRP/Lymphocytes ratio | 6 months after discharge | 0.51 (0.39–2.11) (n=7) | 0.19 (0.15–0.29) (n=9) | W=44.00; Δ_HL=0.26; 95% IC [-0.19 ; 2.88] | 0,204 | 0,452 |
| Derived inflammatory–nutritional indices | 127 | Ferritin/Albumin ratio | Preoperative | 139.08 (32.87–193.63) (n=10) | 67.42 (29.82–241.05) (n=14) | W=74.00; Δ_HL=3.89; 95% IC [-114.90 ; 130.81] | 0,838 | 0,922 |
| Derived inflammatory–nutritional indices | 128 | Ferritin/Albumin ratio | 3 months after discharge | 115.69 ± 105.31 (n=9) | 67.66 ± 44.74 (n=9) | t(16)=1.26; Δ_mean=48.03; 95% IC [-32.82 ; 128.88] | 0,226 | 0,452 |
| Derived inflammatory–nutritional indices | 129 | Ferritin/Albumin ratio | 6 months after discharge | 49.10 (34.48–111.07) (n=7) | 50.73 (28.26–72.07) (n=9) | W=38.00; Δ_HL=10.62; 95% IC [-32.08 ; 100.24] | 0,525 | 0,700 |
| Derived inflammatory–nutritional indices | 130 | Ferritin/Lymphocytes ratio | Preoperative | 253.10 (70.16–391.97) (n=10) | 205.14 (68.98–486.31) (n=14) | W=67.00; Δ_HL=-19.29; 95% IC [-263.96 ; 310.68] | 0,884 | 0,922 |
| Derived inflammatory–nutritional indices | 131 | Ferritin/Lymphocytes ratio | 3 months after discharge | 101.82 (63.26–330.16) (n=9) | 103.87 (78.10–131.74) (n=9) | W=43.00; Δ_HL=23.72; 95% IC [-69.74 ; 250.87] | 0,860 | 0,922 |
| Derived inflammatory–nutritional indices | 132 | Ferritin/Lymphocytes ratio | 6 months after discharge | 220.62 ± 224.95 (n=7) | 89.98 ± 53.58 (n=9) | t(14)=1.70; Δ_mean=130.63; 95% IC [-34.45 ; 295.72] | 0,112 | 0,405 |
| Derived inflammatory–nutritional indices | 133 | Albumin-Lymphocytes-CRP index | Preoperative | 2.17 (0.86–4.11) (n=10) | 0.68 (0.30–1.52) (n=14) | W=96.00; Δ_HL=0.92; 95% IC [-0.45 ; 2.98] | 0,135 | 0,405 |
| Derived inflammatory–nutritional indices | 134 | Albumin-Lymphocytes-CRP index | 3 months after discharge | 3.20 (1.65–8.87) (n=9) | 23.10 (15.36–34.56) (n=9) | W=24.00; Δ_HL=-14.23; 95% IC [-31.01 ; 1.80] | 0,158 | 0,421 |
| Derived inflammatory–nutritional indices | 135 | Albumin-Lymphocytes-CRP index | 6 months after discharge | 8.23 (2.78–10.12) (n=7) | 28.35 (20.21–34.36) (n=9) | W=11.00; Δ_HL=-18.77; 95% IC [-30.23 ; -2.28] | 0,034 | 0,228 |
| Notes: Values are presented as mean +/- standard deviation or median (P25-P75), with the valid n shown for each group and comparison. The analysis used observed available data only; no multiple imputation was performed. The number of patients evaluated was preoperative n = 24, 3 months after discharge n = 18, and 6 months after discharge n = 16. Raw p-values correspond to the statistical test reported in each row. BH-FDR adjusted p-values were calculated with the Benjamini-Hochberg false discovery rate procedure within each Exposure x Domain family: Dietary intake (45 tests), Clinical-nutritional laboratory analyses (66 tests), and Derived inflammatory-nutritional indices (24 tests). The BH-FDR audit found 945 inferential comparisons, 43 raw p-values < 0.05, and 0 BH-FDR adjusted p-values < 0.05. For binary exposures, two-sample t-test/Welch t-test or Mann-Whitney/Wilcoxon rank-sum tests were used as reported. For multicategorical or ordinal exposures, one-way ANOVA or Kruskal-Wallis tests were used; these are omnibus tests and do not establish pairwise differences or dose-response trends without post-hoc or contrast testing. Because this was an exploratory supplementary analysis with multiple comparisons, small subgroup sizes, and no covariate adjustment, the results should be interpreted as hypothesis-generating rather than confirmatory. Abbreviations: BH-FDR, Benjamini-Hochberg false discovery rate; PERT, pancreatic enzyme replacement therapy; TIBC, total iron-binding capacity; UIBC, unsaturated iron-binding capacity. | | | | | | | | |

| **Supplementary Table S12. Informative dropout and death-related attrition sensitivity analyses** | | | | | | | | |  |
| --- | --- | --- | --- | --- | --- | --- | --- | --- | --- |
| **Analytical structure and model completion.** | | | | | | | | |  |
| Diagnostic item | Result | | | | | | | |  |
| Patients in PD cohort | 24 | | | | | | | |  |
| Deaths before 3-month assessment | 6 (25.0%) | | | | | | | |  |
| Deaths before 6-month assessment | 8 (33.3%) | | | | | | | |  |
| Longitudinal variables available at T1/T2/T3 | 46/46 | | | | | | | |  |
| Observed LMM contrasts | 138 contrasts; 0 model errors | | | | | | | |  |
| IPW LMM contrasts | 138 contrasts; 0 model errors | | | | | | | |  |
| Death-sensitivity contrasts | 276 contrasts; 0 model errors | | | | | | | |  |
| Joint/shared-parameter models attempted | 11 priority models; 11 completed | | | | | | | |  |
| **Baseline comparison between participants surviving to 6 months and participants dying before 6 months.** | | | | | | | | |  |
| **Domain** | **Variables, n** | **p<0.05, n** | **Lowest p** | **Variables with lowest p-values** | | | | |  |
| Composite indices | 9 | 0 | 0.118 | Ferritin/Albumin ratio (p=0.118); Ferritin/Lymphocytes ratio (p=0.209); Modified Glasgow Prognostic Score (p=0.476) | | | | |  |
| Dietary intake | 15 | 0 | 0.134 | Selenium intake (mcg/day) (p=0.134); Vitamin E intake (mg/day) (p=0.284); Iron intake (mg/day) (p=0.343) | | | | |  |
| Laboratory biomarkers | 22 | 0 | 0.118 | Ferritin (ng/mL) (p=0.118); Serum vitamin E (p=0.207); Serum vitamin A (p=0.371) | | | | |  |
| **Agreement between observed-data LMM and IPW-weighted LMM across all variables.** | | | | | | | | |  |
| Contrast | Contrasts, n | Observed p<0.05 | IPW p<0.05 | Status changes | ≥30%  relative difference | Median  (relative difference), % | Maximum  (relative difference), % | |  |
| T1 - T2 | 46 | 27 | 27 | 0 | 4 | 1.883 | 3 838.3 | |  |
| T2 - T3 | 46 | 0 | 0 | 0 | 10 | 10.46 | 165.0 | |  |
| T1 - T3 | 46 | 29 | 29 | 0 | 3 | 2.887 | 185.7 | |  |
| Overall | 138 | 56 | 56 | 0 | 17 | 3.609 | 3 838.3 | |  |
| **Panel D. Pattern-mixture and joint/shared-parameter diagnostics.** | | | | | | | | |  |
| **Diagnostic component** | **Terms/models, n** | | | | **Significant/completed, n** | | | |  |
| Death pattern: died before 3 months | 46 | | | | 3 | | | |  |
| Death pattern: died between 3 and 6 months | 46 | | | | 0 | | | |  |
| Time effect: T2 vs T1 | 46 | | | | 27 | | | |  |
| Time effect: T3 vs T1 | 46 | | | | 28 | | | |  |
| Joint/shared-parameter priority models | 11 | | | | 11 | | | |  |
| Models flagged as singular across observed/IPW/pattern/sensitivity blocks | 21 | | | | Diagnostic only | | | |  |
| **Panel E. Detailed robustness results for priority variables.** | | | | | | | | |  |
| **Domain** | **Variable** | **Contrast** | **Observed LMM estimate [95% CI], p** | **IPW LMM estimate [95% CI], p** | **IPW relative difference, %** | **Adverse quantile**  **estimate, p** | **Adverse extreme estimate, p** | **IPW material-change flag** |  |
| Dietary intake | Energy intake (kcal/day) | T1 - T2 | 694.7 [506.5; 882.9], p=<0.001 | 695.2 [506.0; 884.4], p=<0.001 | 0.079 | 774.4, p=<0.001 | 833.4, p=<0.001 | No |  |
|  |  | T2 - T3 | 19.41 [-182.2; 221.1], p=0.846 | 22.12 [-180.6; 224.9], p=0.826 | 13.95 | 39.33, p=0.671 | 80.44, p=0.427 | No |  |
|  |  | T1 - T3 | 714.1 [517.6; 910.5], p=<0.001 | 717.3 [519.9; 914.8], p=<0.001 | 0.457 | 813.8, p=<0.001 | 913.8, p=<0.001 | No |  |
|  | Energy relative to requirement (kcal/day) | T1 - T2 | 139.0 [69.28; 208.7], p=<0.001 | 139.1 [69.31; 208.8], p=<0.001 | 0.066 | 172.2, p=0.002 | 207.1, p=<0.001 | No |  |
|  |  | T2 - T3 | 28.61 [-44.62; 101.8], p=0.433 | 30.66 [-42.62; 103.9], p=0.401 | 7.182 | 94.77, p=0.073 | 92.40, p=0.108 | No |  |
|  |  | T1 - T3 | 167.6 [94.70; 240.5], p=<0.001 | 169.7 [96.82; 242.6], p=<0.001 | 1.281 | 267.0, p=<0.001 | 299.5, p=<0.001 | No |  |
|  | Protein intake (g/day) | T1 - T2 | 23.02 [15.77; 30.27], p=<0.001 | 23.13 [15.86; 30.41], p=<0.001 | 0.507 | 23.92, p=<0.001 | 27.59, p=<0.001 | No |  |
|  |  | T2 - T3 | -4.69 [-12.43; 3.05], p=0.226 | -4.58 [-12.35; 3.19], p=0.239 | 2.328 | -2.94, p=0.394 | -1.85, p=0.622 | No |  |
|  |  | T1 - T3 | 18.33 [10.76; 25.90], p=<0.001 | 18.55 [10.96; 26.15], p=<0.001 | 1.232 | 20.98, p=<0.001 | 25.73, p=<0.001 | No |  |
| Laboratory biomarkers | Albumin (g/dL) | T1 - T2 | -0.14 [-0.49; 0.20], p=0.407 | -0.13 [-0.47; 0.22], p=0.459 | 11.73 | 0.04, p=0.774 | 0.14, p=0.490 | No |  |
|  |  | T2 - T3 | -0.08 [-0.46; 0.30], p=0.682 | -0.10 [-0.48; 0.28], p=0.590 | -30.46 | -0.12, p=0.417 | 0.32, p=0.123 | Yes |  |
|  |  | T1 - T3 | -0.22 [-0.58; 0.14], p=0.221 | -0.23 [-0.59; 0.13], p=0.202 | -3.044 | -0.08, p=0.599 | 0.45, p=0.028 | No |  |
|  | CRP (mg/L) | T1 - T2 | 1.10 [0.61; 1.60], p=<0.001 | 1.09 [0.60; 1.59], p=<0.001 | -1.081 | 0.88, p=<0.001 | 0.85, p=0.002 | No |  |
|  |  | T2 - T3 | 0.17 [-0.38; 0.72], p=0.535 | 0.22 [-0.32; 0.76], p=0.423 | 28.53 | 0.12, p=0.587 | -0.21, p=0.404 | No |  |
|  |  | T1 - T3 | 1.27 [0.76; 1.79], p=<0.001 | 1.31 [0.80; 1.82], p=<0.001 | 2.879 | 1.00, p=<0.001 | 0.64, p=0.015 | No |  |
|  | Ferritin (ng/mL) | T1 - T2 | 0.54 [0.06; 1.03], p=0.029 | 0.53 [0.04; 1.02], p=0.036 | -2.716 | 0.04, p=0.872 | -0.10, p=0.745 | No |  |
|  |  | T2 - T3 | 0.15 [-0.37; 0.67], p=0.561 | 0.16 [-0.37; 0.69], p=0.538 | 7.458 | 0.15, p=0.583 | 0.12, p=0.701 | No |  |
|  |  | T1 - T3 | 0.69 [0.19; 1.20], p=0.009 | 0.69 [0.18; 1.20], p=0.010 | -0.524 | 0.20, p=0.479 | 0.02, p=0.954 | No |  |
|  | Serum vitamin A | T1 - T2 | -0.03 [-0.09; 0.04], p=0.447 | -0.02 [-0.09; 0.05], p=0.507 | 12.31 | 0.01, p=0.857 | 0.02, p=0.476 | No |  |
|  |  | T2 - T3 | -0.01 [-0.09; 0.06], p=0.703 | -0.01 [-0.09; 0.06], p=0.689 | -5.451 | -0.02, p=0.598 | -0.02, p=0.534 | No |  |
|  |  | T1 - T3 | -0.04 [-0.11; 0.03], p=0.256 | -0.04 [-0.11; 0.03], p=0.288 | 6.003 | -0.01, p=0.728 | 0.00, p=0.928 | No |  |
|  | Serum vitamin E | T1 - T2 | 1.94 [0.70; 3.18], p=0.003 | 1.92 [0.69; 3.15], p=0.003 | -0.650 | 2.54, p=<0.001 | 2.65, p=<0.001 | No |  |
|  |  | T2 - T3 | 0.31 [-1.01; 1.64], p=0.634 | 0.38 [-0.94; 1.70], p=0.561 | 21.24 | 0.36, p=0.512 | 0.37, p=0.513 | No |  |
|  |  | T1 - T3 | 2.25 [0.96; 3.54], p=0.001 | 2.30 [1.02; 3.59], p=<0.001 | 2.403 | 2.90, p=<0.001 | 3.02, p=<0.001 | No |  |
| Composite indices | PNI | T1 - T2 | -2.79 [-8.06; 2.48], p=0.292 | -2.56 [-7.83; 2.72], p=0.333 | 8.247 | -0.33, p=0.884 | 0.83, p=0.768 | No |  |
|  |  | T2 - T3 | -0.28 [-6.08; 5.52], p=0.923 | -0.51 [-6.32; 5.30], p=0.860 | -83.40 | -0.35, p=0.876 | 4.86, p=0.090 | Yes |  |
|  |  | T1 - T3 | -3.06 [-8.52; 2.39], p=0.264 | -3.07 [-8.52; 2.39], p=0.264 | -0.053 | -0.68, p=0.763 | 5.69, p=0.048 | No |  |
|  | CRP/Albumin ratio | T1 - T2 | 0.70 [0.37; 1.04], p=<0.001 | 0.69 [0.36; 1.02], p=<0.001 | -1.300 | 0.57, p=<0.001 | 0.53, p=0.006 | No |  |
|  |  | T2 - T3 | 0.07 [-0.30; 0.44], p=0.696 | 0.10 [-0.26; 0.47], p=0.570 | 44.27 | 0.11, p=0.434 | -0.31, p=0.097 | Yes |  |
|  |  | T1 - T3 | 0.77 [0.43; 1.12], p=<0.001 | 0.80 [0.45; 1.14], p=<0.001 | 2.953 | 0.69, p=<0.001 | 0.22, p=0.243 | No |  |
|  | Albumin-Lymphocytes-CRP index | T1 - T2 | -1.24 [-1.84; -0.65], p=<0.001 | -1.22 [-1.81; -0.63], p=<0.001 | 1.671 | -0.87, p=0.003 | -0.85, p=0.008 | No |  |
|  |  | T2 - T3 | -0.27 [-0.92; 0.38], p=0.403 | -0.33 [-0.98; 0.31], p=0.301 | -23.90 | -0.13, p=0.638 | 0.15, p=0.630 | No |  |
|  |  | T1 - T3 | -1.51 [-2.13; -0.90], p=<0.001 | -1.56 [-2.17; -0.94], p=<0.001 | -2.894 | -1.01, p=<0.001 | -0.70, p=0.026 | No |  |
| Notes: PD, pancreatoduodenectomy; LMM, linear mixed-effects model; IPW, inverse probability weighting. T1=preoperative assessment; T2=3 months after discharge; T3=6 months after discharge. All comparisons used baseline values only. p-values are from Wilcoxon rank-sum tests comparing participants surviving to 6 months with participants dying before 6 months. None of the 46 variables differed significantly at p<0.05. The IPW denominator model was alive ~ time + z_age + sexo + z_base_albumin + z_base_log_crp + z_base_energy; the numerator model was alive ~ time. Stabilized weights were truncated at the 5th and 95th percentiles and remained stable (minimum=0.861, median=1.000, maximum=1.217). A status change indicates a shift across the p<0.05 threshold after IPW weighting. The full pattern-mixture model including the time × death-pattern interaction was structurally non-identifiable because participants who died before a given follow-up time had no observations at that time. Therefore, the model was simplified to main effects for time and death pattern. Joint/shared-parameter models were fitted only for priority variables and are exploratory because only 8 deaths were observed. Priority variables were selected a priori because they directly represent the main dietary trajectory, inflammatory/nutritional biomarker profile, fat-soluble vitamin status, and integrated inflammatory–nutritional indices. Estimates are expressed on the analysis scale used for each variable. The adverse quantile and adverse extreme scenarios are deliberately conservative counterfactual death-sensitivity scenarios and should not be interpreted as observed patient values. | | | | | | | | |  |
|  |  |  |  |  |  |  |  |  |  |
|  |  |  |  |  |  |  |  |  |  |
|  |  |  |  |  |  |  |  |  |  |
|  |  |  |  |  |  |  |  |  |  |
|  |  |  |  |  |  |  |  |  |  |
|  |  |  |  |  |  |  |  |  |  |
